# A heme-activatable probe and its application in the high-throughput screening of *Plasmodium falciparum* ring-stage inhibitors

**DOI:** 10.1038/s41392-022-00961-9

**Published:** 2022-05-18

**Authors:** Sheng Liu, Chunyan Wei, Tian Liu, Shuang-Gang Ma, Chen Chen, Hao Lin, Lianhui Zhang, Heng Wang, Chong-Jing Zhang, Shi-Shan Yu

**Affiliations:** 1 State Key Laboratory of Bioactive Substance and Function of Natural Medicines and Beijing Key Laboratory of Active Substance Discovery and Druggability Evaluation, Institute of Materia Medica, Chinese Academy of Medical Sciences and Peking Union Medical College, Beijing, 100050 China; 2 Department of Microbiology and Parasitology, Institute of Basic Medical Sciences Chinese Academy of Medical Sciences, School of Basic Medicine Peking Union Medical College, 5# Dong Dan San Tiao, Beijing, 100005 China

**Keywords:** Infection, Infectious diseases

**Dear Editor**,

The resistance of *Plasmodium falciparum* to artemisinin derivatives (ARTs) in Asian, African and South American countries is a pressing global concern. Heme is an important biomarker of malaria parasites and is closely related to the mode of action of ARTs, but no heme-activatable fluorescent probe has been developed to study the biology of heme in malaria parasites in a light-up manner. Here, we rationally developed probes from the unique reaction between heme and Yingzhaosu A (YZSA), then we unprecedentedly applied the probe to identify ring-stage inhibitors of *P. falciparum*.

Despite being the first endoperoxide-containing antimalarial with a defined structure during the historic 523 project in the 1970s, YZSA is neglected.^1^ Interestingly, we observed *m/z* values of 728.26 and 840.35 for the reaction between YZSA and heme (Supplementary Figs. S1–S3), suggesting that YZSA was attacked by heme from the less sterically hindered side and broke the endoperoxide bond to generate sterically hindered tertiary oxygen-centered radicals (Supplementary Scheme S1). The product subsequently underwent rearrangement to remove the side chain and YZSA was simultaneously cleaved into two separate parts (Fig. 1a). This simultaneity was not observed for ART or other trioxolane antimalarials. Therefore, YZSA provides a good scaffold for a fluorescence turn-on heme probe.

**Fig. 1:**
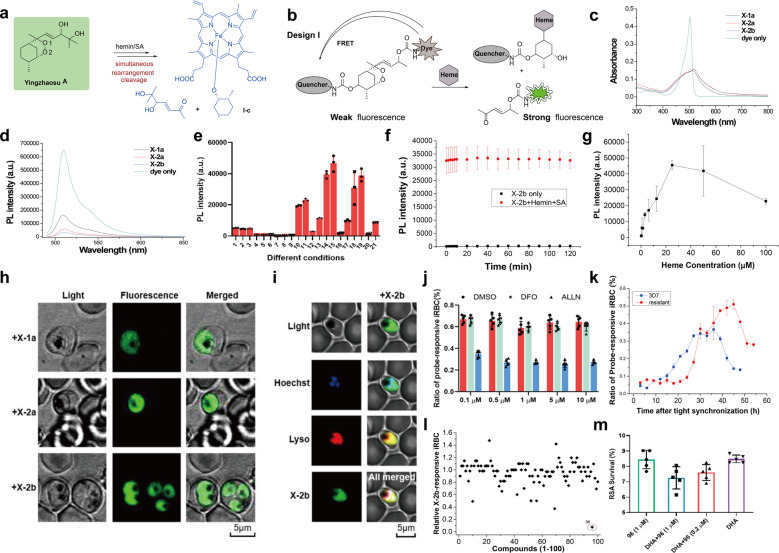
Development of YZSA-based probes to study heme biology and identify ring-stage inhibitors for the most pathogenic parasite, *P. falciparum*. **a** The unique reaction between Yingzhaosu A and heme. **b** Design I for FRET-based heme-reactive probes. **c** Absorption spectra of X-1a, X-2a, X-2b and the BODIPY fluorophore in PBS (5 μM). **d** PL spectra of X-1a, X-2a, X-2b and the BODIPY fluorophore in PBS (5 μM). **e** PL intensity of three probes incubated with/without heme under different conditions for 2 h. The different conditions were as follows: 1, X-1a; 2, X-1a+GSH; 3, X-1a+SA; 4, X-2a; 5, X-2a+GSH; 6, X-2a+SA; 7, X-2b; 8, X-2b+GSH; 9, X-2b+SA; 10, hemin+SA+X-1a; 11, hemin+SA+GSH+X-1a; 12, hemin+X-1a; 13, hemin+GSH+X-1a; 14, hemin+SA+X-2a; 15, hemin+SA+GSH+X-2a; 16, hemin+X-2a; 17, hemin+GSH+X-2a; 18, hemin+SA+X-2b; 19, hemin+SA+GSH+X-2b; 20, hemin+X-2b; and 21, hemin+GSH+X-2b. SA sodium ascorbate. Results are shown as the mean ± SD with *n* = 3 biological replicates. **f** Time-dependent PL intensity of probe X-2b treated with hemin and SA. Results are shown as the mean ± SD with *n* = 3 biological replicates. **g** PL intensity of probe X-2b treated with different concentrations of hemin in the presence of SA. The fluorescence intensity of the probe steadily increased as the concentration of heme increased from 0 to 25 μM. Higher concentrations of heme (50 and 100 μM) did not further increase the fluorescence intensity but instead caused it to decrease greatly, which was ascribed to the quenching effect of heme. Results are shown as the mean ± SD with *n* = 3 biological replicates. **h** Confocal images of *P. falciparum* (3D7) treated with X-1a, X-2a, or X-2b (10 μM) for 3 h. BF bright field, FL fluorescence. **i** Confocal images of *P. falciparum* (3D7) treated with X-2b, Hoechst and lysosome tracker red (LTR). **j** The ratio of probe-responsive iRBCs treated with DFO or ALLN for 1 h and subsequently coincubated with X-2b for 3 h. The ratio of probe-responsive iRBCs denotes the number of probe-imaged RBCs among the examined 100,000 RBCs. Trophozoite-stage parasites were used in the assay. Trophozoite-stage parasites were obtained by allowing the sorbitol-treated parasites to grow further for 16 h. DFO a chelator of ferrous iron, ALL, a cysteine protease inhibitor. Results are shown as the mean ± SD with *n* = 5 biological replicates. **k** Comparing the growth patterns of 3D7 and ART-resistant *P. falciparum* by measuring the ratio of X-2b-imaged RBCs during one life cycle. iRBC infected red blood cell, RBC red blood cell, ALA the precursor of heme biosynthesis, SA the inhibitor of heme biosynthesis. Results are shown as the mean ± SD with *n* = 3 biological replicates. **l** The high-throughput screening results for a library of 100 compounds. The ratio of probe-responsive iRBCs in the presence of the screened compound was normalized to that of the control group without the addition of screened compounds. **m** Ring-stage survival of DHA- and 96-treated 0–3 hpi rings. Results are shown as the mean ± SD with *n* = 5 biological replicates

As shown by fluorescence resonance energy transfer, design I (Fig. 1b) is better than design II (Supplementary Fig. S4) because only the fluorophore in design I could eliminate the quenching effect from both Disperse Red 1 and heme. The designed probes were synthesized from a derivative of (*R*)-carvone (II) using 12 steps (Supplementary Scheme S2). The chemical structures of all intermediates and the final three probes (X-1a/2a/2b) were confirmed by multiple experiments, including X-ray diffraction, electronic circular dichroism, ^1^H NMR, ^13^C NMR, COSY, HMBC, HMQC, NOE and HRMS (Supplementary Tables S1–S7 and Supplementary Figs. S25–S175).

The probes X-1a/2a/2b and their cleaved product had low toxicity toward *P. falciparum* (Supplementary Figs. S5 and S6). Three probes had broader absorption spectra than that of Bodipy (Fig. 1c). The quencher group decreased the fluorescence intensity of X-1a/2a/2b by 3.0-fold, 10-fold and 17.9-fold compared to that of Bodipy dye alone (Fig. 1d). However, in the presence of heme from the in situ reduction in hemin with sodium ascorbate or glutathione (Supplementary Table S8), the fluorescence intensity of X-1a/2a/2b increased by 3.1-fold, 43-fold and 118-fold compared to that of the probe alone (Fig. 1e and Supplementary Fig. S7). The reactions between heme and the probes occurred in the same manner as that of reaction between YZSA and heme (Supplementary Figs. S8–S13). The reactions proceeded so quickly that the fluorescence intensity dramatically increased during the time period between the sample preparation and the first reading of the microplate reader (Fig. 1f and Supplementary Fig. S14). The calculated limits of detection (LODs) for X-1a/X-2a/X-2b were 0.14, 0.062 and 0.041 μM, respectively (Fig. 1g and Supplementary Fig. S15 and S16). Therefore, among the three probes, X-2b with both *R* configurations at C-8 and C-12 positions had the best performance regarding its photophysical properties and LOD. The probes were also fluoresced in the presence of ferrous and cuprous ions in vitro (Supplementary Fig. S17 and Supplementary Table S9), but the following in situ experiments indicated that the probes were mainly activated by heme inside parasites.

The microscopic imaging of blood-stage *P. falciparum* with probes revealed that the probes had complete parasite specificity, and fluorogenic labeling was absent in the cytosol of both noninfected and infected erythrocytes (Fig. 1h). Furthermore, the coimaging experiment with Hoechst and lysosome tracker red indicated that the fluorophore released from probe X-2b lighted the whole parasite except for some parasite pigments (Fig. 1i). These imaging results implied that cuprous ions played a negligible role in fluorescing the probes inside malaria parasites, as normal red blood cells (RBCs) had a higher content of copper than that of infected red blood cells (iRBCs) and parasites.^2^ Second, the coincubation of deferoxamine (chelator of free ferrous iron) had a negligible effect on the ratio of probe-responsive iRBCs while ALLN (inhibitor of hemoglobin digestion) treatment decreased the ratio of probe-responsive iRBCs by 60% (Fig. 1j), indicating that heme, but not ferrous ions, lit up the probe inside parasites. Moreover, the addition of δ-aminolevulinic acid (the precursor of heme biosynthesis) or succinylacetone (an inhibitor of heme biosynthesis) had no effect on the ratio of probe-responsive iRBCs (Supplementary Fig. S18); therefore, the heme that activated the probes was mostly originated from hemoglobin digestion. In line with the time-dependent property of hemoglobin digestion, the ratio of probe-responsive iRBCs did not increase until the synchronized parasites had grown for 9 h post synchronization, and the ratio reached a maximum at 36 h post synchronization (Fig. 1k, blue curve). Then, the ratio decreased steadily from 36 to 45 h, indicating that some of the parasites reached the ring stage in the next growth cycle. In addition, probe X-2b could detect prolonged low-heme levels in ART-resistant *P. falciparum* (6320)^3^ (Supplementary Fig. S19) with a decelerated growth pattern (Fig.1k, red curve); this pattern is consistent with the phenotype of the decelerated ring stage^4^ and the reduced endocytosis of hemoglobin in the food vacuole in ART-resistant strains.^5^ Together, these in situ results indicated that heme played a predominant role in X-2b activation inside parasites.

Finally, the X-2b was employed to set up a high-throughput screening (HTS) assay to identify *P. falciparum* ring-stage inhibitors. The rationale of HTS is that compounds inhibiting the ring-stage parasite generate a lower ratio of probe-responsive iRBCs than that of the corresponding non-inhibiting compounds. After optimizing the screening conditions (Supplementary Fig. S20), we screened a library of 100 natural products with diverse structures (Supplementary Table S10). Triterpene and alkaloid natural products showed high activity among the top 10 hits (Supplementary Fig. S21). Triterpenoid compound 96, also called pristimerin, had the strongest inhibitory effect on the ratio of probe-responsive iRBCs (Fig. 1l). The morphology of the tiny pyknotic spots together with the stage susceptibility profile indicated that pristimerin could potently inhibit ART-sensitive and ART-resistant parasites in the ring stage (Supplementary Figs. S22–S24). The ring-stage survival assay (RSA) demonstrated that the addition of pristimerin significantly increased the ablation of ART-resistant parasites by dihydroartemisinin (DHA) (Fig. 1m), establishing that pristimerin combines effectively with DHA to kill resistant parasites.

In summary, we describe the first light-up probes to study heme biology and identify ring-stage inhibitors for the most pathogenic parasite, *P. falciparum*. The HTS assay revealed that ring-stage inhibition is an overlooked property of pristimerin that can ablate *P. falciparum*. More broadly, the new light-up heme probe could complement current methods, including genetic checking and RSA assays, to monitor the development of ART resistance in regions with high malaria morbidity due to *P. falciparum*. In addition, we believe that pristimerin, which was discovered by heme-mediated HTS, could work well in combination with ARTs to efficiently kill *P. falciparum*. Furthermore, as the initial attempt to find a sensitizing small molecule that could increase the potency of ART toward ART-resistant *P. falciparum* was successful, a larger-scale HTS to identify more sensitizers that can increase the potency of the artemisinin-based combination therapy regimen is justifiable, and this will certainly enhance our confidence in addressing ART resistance in *P. falciparum*.

## Supplementary information

Supplementary material

## Data Availability

The raw data that support the findings of this study are available from the corresponding author upon reasonable request.
