# Supplementary material for: A heme-activatable probe and its application in the high-throughput screening of Plasmodium falciparum ring-stage inhibitors
Source: Signal Transduct Target Ther. 2022 May 18;7:160. doi: 10.1038/s41392-022-00961-9 (PMC9114409; doi:10.1038/s41392-022-00961-9)
Supplement: Supplementary file 1 — Supplementary material [file 41392_2022_961_MOESM1_ESM.pdf]

Supplementary Materials for

A Heme-Activatable Probe and Its Application in the High-Throughput Screening of *Plasmodium Falciparum* Ring-Stage Inhibitors

Sheng Liu[1]‡, Chunyan Wei[2]‡, Tian Liu[1]‡, Shuang-Gang Ma[1]‡, Chen Chen[1], Hao Lin[1], Lianhui Zhang[2], Heng Wang[2]*, Chong-Jing Zhang[1]*, Shi-Shan Yu[1]*

[1] State Key Laboratory of Bioactive Substances and Functions of Natural Medicines, Institute of Materia Medica, Chinese Academy of Medical Sciences and Peking Union Medical College, Beijing, 100050, China.
[2] Department of Microbiology and Parasitology, Institute of Basic Medical Sciences, Chinese Academy of Medical Sciences & School of Basic Medicine, Peking Union Medical College, Beijing, 100005, China.
‡ These authors contributed equally to this work.

* Corresponding authors: yushishan@imm.ac.cn
zhangchongjing@imm.ac.cn
wanghpumc@163.com

This PDF file includes:

Materials and Methods

Figures S1 to S175

Schemes S1 to S2

Tables S1 to S10

Materials and Methods

Reagents and facilities

All commercial chemicals were used as supplied unless otherwise indicated. UV spectra were measured on a JASCO V650 spectrophotometer. Fluorescence spectra were measured on FLS1000 photoluminescence spectrometer. CD spectra were measured on a JASCO J-815 spectropolarimeter. NMR spectra were obtained on an INOVA-500 or a Bruker-600 NMR spectrometer. Chemical shifts are given in δ (ppm) with solvent peaks as references. LC−HRMS analysis was performed on a Thermo FisherExactive Plus mass spectrometer equipped with a ThermoFisher Accela HPLC system (ThermoFisher Scientific, Bremen, Germany). Preparative HPLC was performed on a Shimadazu LC-6AD instrument with SPD-20A and RID-10A detectors, using an Xtimate C18 column (250 × 20 mm, 5 μm). Silica gel (200-300 mesh, Qingdao Marine Chemical Factory, China) were used for column chromatography. TLC was carried out with glass precoated Si gel GF254 plates. The library of screened compounds was purchased from TOPSCIENCE.

General procedure for the reaction of Yingzhaosu A and heme detected by LC-HRMS

To the hemin solution (1.0 mL, 0.01 mmol in 0.1 M NaOH) was added sodium L-ascorbate (19.8 mg, 0.10 mmol, 10 equiv) with or without GSH (30.7 mg, 0.10 mmol, 10 equiv). Then a solution of Yingzhaosu A (0.01 mmol, 1 equiv) in acetonitrile (1.0 mL) was added. The mixture was stirred under argon at room temperature and monitored by LC-HRMS.

LC-HRMS analysis: Compounds were eluted using a binary gradient solvent system consisting of acetonitrile/1% formic acid (solvent A) and H_2O/1% formic acid (solvent B). The gradient used was 5% to 95% of solvent A over 10 min (flow rate: 0.5 mL/min). Retention time was 3.98 min for heme and 4.14 to 5.13 min for the heme-cyclohexane adducts.

General procedure for the reaction of YZSA-based probes and heme detected by LC-HRMS

To the hemin solution (50 μL, 0.001 mmol in 0.1 M NaOH) was added sodium L-ascorbate (1.98 mg, 0.01 mmol, 10 equiv) with or without GSH (3.07 mg, 0.01 mmol, 10 equiv). Then a solution of probe (0.01 mmol, 1 equiv) in acetonitrile (0.1 mL) was added. The mixture was stirred under argon at room temperature and monitored by LC-HRMS.

General procedure for the reaction of YZSA-based probes and heme under different conditions detected by microplate reader

We first prepared the working solution for hemin, sodium ascorbate, GSH, and probes. Preparation of working solution of hemin (1mM): Hemin (2.6 mg) was first dissolved in 1M NaOH (aqueous solution, 0.25 mL), followed by addition of Tris buffer (1M, 2.5 mL). The pH was adjusted to 8 using 1M HCl. Then the volume was topped up to 3.9 mL. Preparation of working solution of GSH (10mM): GSH (2.0 mg) was dissolved in water (0.65 μL). Preparation of working solution of sodium ascorbate (10mM): Sodium ascorbate (10.5 mg) was dissolved in water (0.535 μL), followed by dilution to the working solution. Preparation of working solution of the probe (1mM): The probe (0.942 mg) was dissolved in DMSO (1 mL). Then we checked the fluorescence intensity of probes according to the conditions in the table S8. The fluorescence signal was measured at 37 °C every 2 min for 2 h with excitation and emission wavelength bands centered at 480 and 530 nm, respectively. At least three biological replicates were carried out. Rates of fluorescence increase were determined for each sample using the initial rate method and compared to determine reactivity.

We first prepared the working solution for metal ions, sodium ascorbate and probes. Preparation of working solution of Fe^{2+} (0.55 mM): $FeSO_4·7H_2O$ (15.2 mg) was dissolved in purified water (1mL), followed by dilution to the working solution. Preparation of working solution of other metal ions (1 mM): Solutions of the indicated metal ions were prepared in purified water to 100 mM, followed by dilution to the working solution. Preparation of working solution of sodium ascorbate (10 mM): Sodium ascorbate (19.8 mg) was dissolved in water (1 mL), followed by dilution to the working solution. Preparation of working solution of the probe (10 μM): Dilute to working concentration with 1mM probes. Then we checked the fluorescence intensity of probes according to the conditions in the table S9. The fluorescence signal was measured at rt every 2 min for 0.5-2 h with excitation and emission wavelength bands centered at 480 and 530 nm, respectively. At least two or three biological replicates were carried out.

Measurement of UV–vis and fluorescence spectra

The stock solution of probe X-1a (1.0 mM), X-2a (1.0 mM), X-2b (1.0 mM) and bodipy dye (1.0 mM) were prepared in DMSO of analytical grade. Dilution of the stock solution by phosphate buffer saline (PBS) buffer of pH 7.4 gave rise to the desired concentration at 10.0

μM, and a final volume of 2.0 mL was thereby obtained for spectral investigation. As for the fluorescence experiments, excitation and emission wavelength were set at 480 nm and 480–750 nm respectively. The fluorescence quantum yield was measured with the solution of bodipy dye (2 μM), X-1a (30 μM), X-2a (5 μM) and X-2b (2.5 μM) in PBS buffer.

Calculation of limit of detection for three probes to detect heme

To the PBS buffer (1×, 170 μL) was added 10 μL of hemin solution with stock concentration from 2 mM to 3.9 μM. Then 10 μL sodium ascorbate solution (10 mM) and 10 μL probe (1 mM) was added to the above mixture. The fluorescence of reaction mixture was measured at 37 °C every 2 min for 2 h in the Microplate Reader (Tecan Austria GmbH) with excitation and emission wavelength bands centered at 480 and 530 nm, respectively. The reading at the 10 min was used to calculat the limit of detection (LOD) based on the following on formula: LOD = 3σ/k, where σ is the standard derivation of the fluorescent intensity of mixture with the lowest heme concentration (0.19 μM) and k is the slop of the fluorescent intensity over heme concentration (0.19-3.1 μM).

Plasmodium falciparum cultures, synchronization and morphology observation

P. falciparum ART-sensitive strain 3D7 and ART-resistant PfKelch13:C580Y strain 6320 were cultured in 5% O_2 and 5% CO_2 in N_2 as previously described.[1] Briefly, parasites were incubated in petri dishes at 37°C using 4% haematocrit of human red blood cells with RPMI Medium1640, supplemented with 25 mM HEPES, 10 mg/L Glucose, 0.5% Albumax II, 0.3 g/L L-Glutamine, 0.015% Hypoxanthine, 0.125 g/L gentamicin, and 25 mM sodium bicarbonate.

To test the performance of the three probes X-1a/2a/2b in imaging live *P. falciparum* parasites strain 3D7 with flow cytometry, synchronization of parasite cultures with two rounds of treatment with D-sorbitol (5%, w/v) was performed as previously described[2] and parasites of ring stage with time window 0-8 hour were obtained.

To study the growth pattern of both *P. falciparum* strain 3D7 and PfKelch13: C580Y strain 6320 with probe X-2b, tight synchronization was performed essentially as described[3] with slight modifications. Briefly, parasites were firstly synchronized by two successive treatment of sorbitol (5%). Then 40%/70% Percoll discontinuous gradient was used to enrich the mature schizonts which were further washed in RPMI-1640, and cultured with fresh erythrocytes. Six hours later, purified mature schizonts were treated with D-sorbitol (5%, w/v) again to obtain 0-

6 hour old rings. For the *in vitro* ring-stage survival assay$^{0\text{-}3h}$ (RSA$^{0\text{-}3h}$), the same tight synchronization was performed except the final 5% sorbitol treatment was done at 3 h after mature schizonts enrichment with Percoll discontinuous gradient. Giemsa-stained thin blood smears were made and observed under microscope to measure the parasitaemia and to observe the parasite morphology.

Confocal imaging of P. falciparum parasites treated with probes

The fluorescence images of live *P. falciparum* parasites strain 3D7 with the three probes X-1a/2a/2b were taken on an Olympus FV1000 confocal microscope, equipped with a with 60x (1.35 NA) oil immersion objective (Olympus, Tokyo, Japan). For this, *P. falciparum* 3D7 parasites of mixed asexual stage at 5% parasitaemia were incubated separately with the three probes X-1a/2a/2b of 10 μM for three hours under normal culture condition. Then one drop (10 μL) of culture was placed on the slide and further covered by a cover glass. Then slides were viewed with 488 nm excitation. The range of emitted fluorescence was measured from 510 to 550 nm. To investigate the cellular location of X-2b, Hoechst 33342 (Solarbio, China) was used for labelling nucleus and LysoTracker Red DND-99 (LTR) (ThermoFisher Scientific, USA) was used to stain food vacuole (FV) in parasites. Parasite culture was incubated with X-2b (10 μM), Hoechst (10 μg/ml) and LTR (75 nM) for three hours, 30 minutes, and one hour, respectively. The stained parasites culture was then washed in 20 times the volume of the culture medium, and observed within half an hour using sequential scanning at 405 nm (Hoechst), 488 nm (X-2b), 568 nm (LTR), excitation, and 425±15 nm, 525±25nm, and 575~625 nm emission for Hoechst, X-2b and LTR respectively. Images were produced using the FV10-ASW Fluoview software 3.0 (Olympus), and were not modified beyond the standard adjustment of intensity.

Flow cytometry analysis of the P. falciparum parasites treated by probes

To detect the performance of the probe (X-2b) to image parasites, highly synchronized *P. falciparum* strain 3D7 parasite cultures with a 0-8h time window) with the parasitemia of 2.22% (measured by Giemsa stained smear) at the hematocrit of 4% were pooled into the 96 well plates in triplicates. Parasites of different stages were incubated with each probe (10 μM) for three hours. Then, 1/10 of the culture volume (15 μL) in each well was resuspended in new

complete culture medium (200 μL) and filtered with a cell strainer with the pore diameter of 40 μm before flow cytometry. Data were collected using an Accuri C6 Flow Cytometer® system from the FL1 (488nm Excitation and 530±15nm Emission detection) with acquisition of 100 000 events/samples. Gating was done by uninfected and infected erythrocytes treated with DMSO.

When examining the effects of ferrous ion chelator deferoxamine (DFO), calpain inhibitor I (ALLN), δ-aminolevulinic acid (ALA) and succinylacetone (SA) on activation of X-2b by flow cytometry analysis, *P. falciparum* 3D7 parasite culture was treated with one round synchronization of D-sorbitol (5%, w/v). DFO, ALLN, ALA, and SA of and the control DMSO of different concentrations (0.1 μM, 0.5 μM, 1 μM, 5 μM and 10 μM) was incubated with the synchronized parasite culture separately for one hour and then X-2b was added to each sample and incubated for three hours at the concentration of 10 μM. Quintuplicates was used for each concentration. For and DFO and ALLN, parasite culture of 16 hours after synchronization with the parasitemia of 2.67% (measured by Giemsa stained smear) and a haematocrit of 4% hematocrit was used. For ALA and SA, parasite culture just after the synchronization with the parasitemia of 3.46% (measured by Giemsa stained smear) and the same haematocrit was used.

When using probe X-2b to study the growth pattern of the both *P. falciparum* strain 3D7 and PfKelch13:C580Y strain 6320, the procedures were similar to the previous section except that the parasites were more tightly synchronized with a 0-6 h time window and a lower initial parasitemia of 0.5%-1%/2% haematocrit for both strains. Giemsa-stained thin blood smears were made using pellets from the remaining culture and examined with a microscopy coupled to a CCD camera.

High-throughput screening of small molecules altering the heme level of P. falciparum parasites using the light-up heme probe (X-2b)

Before evaluating the effects of screened compounds on altering the heme level, we first optimized the screening condition in terms of the age of parasites, the concentration of probe X-2b and the time of incubation between the parasites and probe. To do so, the *P. falciparum* strain 3D7 parasites synchronized with two rounds of D-sorbitol (5% w/v) treatment. The parasites at different time periods (7 h, 8 h, 9 h, 10 h) were incubated with the probe X-2b (5

μM) for 2 h, followed by the analysis with flow cytometry. To optimize the concentration and incubation time of probe X-2b, the synchronized *P. falciparum* strain 3D7 parasites (0-8h window) was firstly allowed to grow for 9 h. Then parasites at this stage were incubated with the probe of different concentrations (1 μM, 2.5 μM, 5 μM and 10 μM) for different incubation time (1h, 2h, 3h). Then the probe-responsive iRBCs were further analyzed using the flow cytometry method.

For the high-throughput screening, synchronized *P. falciparum* strain 3D7 parasites (0-8h window) were prepared and pooled to 96-well plates. The screened compounds (5 μM) were incubated with the parasites for 9 h, followed by a further incubation with probe X-2b (10 μM) for 3 h. Then the ratio of probe-responsive iRBC was analyzed by flow cytometry. In the preliminary screening, each compound was screened in duplicate. For further validation, the hit compounds were repeatedly screened with different concentrations (0.1μM, 0.5 μM, 1μM, 2.5 μM, 5 μM and 10 μM) in triplicate. In order to observe morphology of parasites treated with screened compounds, thicker smears stained with Giemsa were also made.

In vitro ring-stage survival assay$^{0\text{-}3h}$ (RSA$^{0\text{-}3h}$)

RSA$^{0\text{-}3h}$ assay was done essentially as described in previous reports.[3,4] Briefly, 0-3 h old rings were obtained as mentioned above (see culturing *Plasmodium falciparum* and its synchronization and morphology observation). Ring cultures adjusted to 1% parasitaemia were pooled to 96-well plates and treated with different compounds for 6 h, followed by further culturing for 66 h. Then parasites were stained with 0.2×SYBR Green I (Sigma) and 0.3 μM MitoTracker Deep Red FM (Invitrogen) as described in the previous report.[5] The viability of parasites was analyzed by flow cytometry with the Accuri C6 Flow Cytometer® system. Data was collected from the FL1(488 nm Excitation and 530±15 nm Emission detection) and FL4 (640 Excitation and 675+12.5 nm Emission detection) with acquisition of 100 000 events/sample. Gating was done with DMSO-treated cells without staining, DMSO-treated cells stained with SYBR Green I only and DMSO-treated cells stained with MitoTracker Deep Red FM only.

For studying the role of DHA and 96#, 0-3 h old rings of the PfKelch13:C580Y strain 6320 parasites were incubated with 96# (1μM) alone, or DHA (700 nM) alone according to the

following incubation time period: 0-6 h, 9-15 h, 18-24 h, 27-33 h, 36-42 h, 45-51 h, 54-60 h, and 61-67 h. After each incubation, the drug-containing media was removed and the treated cells were further washed three times with fresh media. The cells were further cultured till 72 h before viability analysis. Five biological repetitions per experiment were carried out. Then parasites were stained with 0.2×SYBR Green I (Sigma) and 0.3 μM MitoTracker Deep Red FM (Invitrogen) as described in the previous report (33) and the viability of parasites was analyzed by flow cytometry with the Accuri C6 Flow Cytometer® system. Giemsa-stained thin smears were also made to examine the morphology and state of the parasites at the end of assay.

For studying the combination effect of DHA and 96 in the $RSA^{0\text{-}3\ h}$ assay, the 0-3 h old rings of PfKelch13:C580Y strain 6320 parasites incubated both with 96 alone of different concentrations (1μM, 0.2 μM), or co-incubated with DHA for 6 h, followed by further culturing for 66 h. Five biological repetitions per experiment were carried out. Then parasites were stained with 0.2×SYBR Green I (Sigma) and 0.3 μM MitoTracker Deep Red FM (Invitrogen) as described in the previous report[5] and the viability of parasites was analyzed by flow cytometry with the Accuri C6 Flow Cytometer® system. Data were collected from the FL1 (488 nm Excitation and 530±15 nm Emission detection) and FL4 (640 Excitation and 675+12.5 nm Emission detection) with acquisition of 100 000 events/sample. Gating was done with DMSO-treated cells without staining, DMSO-treated cells stained with SYBR Green I only and DMSO-treated cells stained with MitoTracker Deep Red FM only.

Viability of parasites treated with three probes X-1a/2a/2b

The toxicity of three probes X-1a/2a/2b towards parasites was measured by the SYBR Green I-based 96 microplate assay as previously described with slight modifications[1]. In brief, highly synchronized ring-stage *P. falciparum* 3D7 strain parasite cultures were diluted with fresh erythrocytes and complete medium to 0.5% parasitaemia and 2% haematocrit. The cultures were incubated with each probe at different concentrations according to a 2-fold serial dilution for 72h. Then the cultures were stained with SYBR Green I (Invitrogen) in lysis buffer for 30 min at 37℃ in the dark. The fluorescence was measured using Synergy H1 Hybrid Multi-Mode Microplate Reader (BioTek) with excitation and emission wavelength bands centered at 485 and 528 nm, respectively. To eliminate the fluorescence influence of probe

itself on fluorescence measuring, cultures incubated with probes X-1a/2a/2b each but without SYBR Green I staining was carried out as internal control. Each experiment was run in triplicates. The half-maximal inhibitory concentrations (IC_{50}) of probes X-1a/2a/2b against *P. falciparum* 3D7 were calculated and plotting of the sigmoidal dose–response curve was performed using the software GraphPad Prism 5.

Detailed asexual blood stage susceptibility profiles for DHA and pristimerin towards P. falciparum (3D7)

We employed a recently reported approach[7] to profile the blood stage susceptibility for DHA and pristimerin. Briefly, tight synchronized rings of the *P. falciparum* 3D7 (0-3 h old) were incubated with 96#, or DHA with different concentrations according to the following incubation time period: 0-8 h, 8-16 h, 16-24 h, 24-32 h, 32-40 h. After each incubation, the drug-containing media was removed and the treated cells were further washed three times with fresh media. The cells were further cultured till 72 h before viability analysis by flow cytometry with SYBR Green and MitoTracker Deep Red FM staining (Life Technologies).

General procedures for the synthesis of the fluorescent probes

All commercial chemicals were used as supplied unless otherwise indicated. All yields reported refer to the yields of the isolated compounds. UV spectra were measured on a JASCO V650 spectrophotometer. Fluorescence spectra were measured on FLS1000 photoluminescence spectrometer. CD spectra were measured on a JASCO J-815 spectropolarimeter. NMR spectra were obtained on an INOVA-500 or a Bruker-600 NMR spectrometer. Chemical shifts are given in δ (ppm) with solvent peaks as references. LC−HRMS analysis was performed on a Thermo FisherExactive Plus mass spectrometer equipped with a ThermoFisher Accela HPLC system (ThermoFisher Scientific, Bremen, Germany). Preparative HPLC was performed on a Shimadazu LC-6AD instrument with SPD-20A and RID-10A detectors, using an Xtimate C18 column (250 × 20 mm, 5 μm). Silica gel (80-100, 200-300 mesh, Qingdao Marine Chemical Factory, China) were used for column chromatography. TLC was carried out with glass precoated Si gel GF254 plates.

Synthesis of compound II-a

To a photocatalytic reaction vessel under oxygen was added compound II (1.2 g, 7.31 mmol), methylene blue (25 mg, 0.78 mmol), acetonitrile (40 mL). The reaction mixture was stirred at 0 °C for 1 h. Compound II was consumed in total up to 14.4 g and the resulting compound II-a was used for the next step without any treatment.

Synthesis of compound III

The synthetic procedure was same as previous report.[6] To the solution of compound II-a in acetonitrile, *p*-Toluenesulfonic acid (1000 mg, 5.8 mmol) was added, and the reaction mixture was stirred at room temperature for 12 h. Then the solvent was removed under reduced pressure and the residue was purified by flash chromatography on a silica gel column using PE-EA (100:1 to 20:1) as the eluent to provide 8.6 g (50%) of compound III as a colorless oil. A small portion of compound III was separated with preparative HPLC to give III-1 and III-2. The configurations of III-1 and III-2 were assigned based on the previous report.[1] Compound III-1: ^{1}H NMR (500 MHz, $CDCl_3$) δ_H 6.20 (1H, dd, J = 16, 11.5 Hz), 5.21-5.26 (2H, m), 4.34 (1H, s), 2.77 (1H, d, J = 15.5 Hz), 2.59 (1H, d, J = 13.5 Hz), 2.43-2.47 (2H, m), 2.32 (1H, s), 1.81 (1H, d, J = 13.5 Hz), 1.26 (3H, d, J = 6.5 Hz), 1.14 (3H, s); ^{13}C NMR (125 MHz, $CDCl_3$) δ_C 208.0, 141.9, 114.5, 84.0, 81.9, 48.6, 43.0, 36.8, 29.4, 23.6, 10.9; HRESI-MS *m/z*: 197.1164 $[M + H]^+$ (calcd 197.1178 for $C_{11}H_{17}O_3$); 219.0982 $[M + Na]^+$ (calcd 219.0997 for $C_{11}H_{16}O_3Na$). Compound III-2: ^{1}H NMR (500 MHz, $CDCl_3$) δ_H 5.63 (1H, dd, J = 17.5, 12.5 Hz), 5.13 (1H, d, J = 12.5 Hz), 5.04 (1H, d, J = 17.5 Hz), 4.38 (1H, s), 2.75 (1H, d, J = 16.0 Hz), 2.67 (1H, d, J = 13.5 Hz), 2.38-2.42 (1H, m), 2.30 (1H, d, J = 15.5 Hz), 1.87 (1H, d, J = 14.0 Hz), 1.59 (3H, s), 1.28 (3H, d, J = 5.0 Hz); ^{13}C NMR (125 MHz, $CDCl_3$) δ_C 207.8, 139.1, 114.7, 83.0, 81.9, 48.8, 43.6, 37.2, 28.5, 22.0, 11.0; HRESI-MS *m/z*: 197.1167 $[M + H]^+$ (calcd 197.1178 for $C_{11}H_{17}O_3$); 219.0985 $[M + Na]^+$ (calcd 219.0997 for $C_{11}H_{16}O_3Na$).

Synthesis of compound IV

Ozone was bubbled through a cooled (-78 °C) solution of compound III (8.6 g, 43.9 mmol, 1.0 equiv) in dry CH_2Cl_2 (150 mL) until the color of reaction mixture turned from colorless to blue. Then, the ozone generator was turned off and oxygen was bubbled through for a few minutes. Then, the argon flow was turned on to discharge the excessive oxygen. Then the mixture was transferred to the room temperature, followed by addition of dimethylsulfide (8.0 mL, 109.4 mmol, 2.5 equiv). After being stirred for 30 min, the reaction mixture was then

concentrated under reduced pressure and the residue was purified by silica gel column chromatography (PE/EA, 10:1→6:1, v/v) to afford compounds IV-1 (2.2 g, 25.3%) and IV-2 ((1.8 g, 20.7%) as a colorless oil. Compound IV-1: ^{1}H NMR (500 MHz, $CDCl_3$) δ_H 9.80 (1H, s, H-10), 4.39 (1H, m, H-2), 2.70 (1H, dt, J = 16.0, 3.0 Hz, H-5a), 2.63 (1H, m, H-4), 2.51 (1H, m, partial overlap, H-5b), 2.47 (1H, m, partial overlap, H-1), 2.32 (1H, ddd, J = 13.5, 7.0, 3.0 Hz, H-3a), 1.89 (1H, ddd, J = 13.5, 3.0, 2.0 Hz, H-3b), 1.29 (3H, d, J = 6.5 Hz, H-7), 1.08 (3H, s, H-9). ^{13}C NMR (125 MHz, CDCl3) δ_C 207.3 (C-6), 202.6 (C-10), 88.0 (C-8), 82.2 (C-2), 48.5 (C-1), 42.2 (C-5), 32.7 (C-4), 30.1 (C-3), 17.4 (C-9), 10.9 (C-7). HRESI-MS *m/z*: 199.0960 $[M + H]^+$ (calcd 199.0970 for $C_{10}H_{15}O_4$); 221.0775 $[M + Na]^+$ (calcd 221.0790 for $C_{10}H_{14}O_4Na$). Compound IV-2: ^{1}H NMR (500 MHz, $CDCl_3$) δ_H 9.35 (1H, s, H-10), 4.43 (1H, m, H-2), 2.62 (1H, ddd, J = 10.5, 7.0, 3.5 Hz, H-3a), 2.39-2.52 (4H, overlap, H-1, 4, 5), 1.91 (1H, ddd, J = 13.5, 3.0, 2.0 Hz, H-3b), 1.55 (3H, s, H-9), 1.29 (3H, d, J = 7.0 Hz, H-7). ^{13}C NMR (125 MHz, $CDCl_3$) δ_C 206.9 (C-6), 200.6 (C-10), 87.8 (C-8), 82.5 (C-2), 48.8 (C-1), 43.4 (C-5), 35.5 (C-4), 28.2 (C-3), 17.9 (C-9), 11.0 (C-7). HRESI-MS *m/z*: 199.0959 $[M + H]^+$ (calcd 199.0970 for $C_{10}H_{15}O_4$); 221.0777 $[M + Na]^+$ (calcd 221.0790 for $C_{10}H_{14}O_4Na$).

Synthesis of compounds V-1 and V-2

To a solution of IV-1 (2.2 g, 11.1 mmol) in 50 mL of CH_2Cl_2 was added the solution of 1-triphenylphosphonene-2-acetone (4.2 g, 13.2 mmol, dissolved in 15 mL CH_2Cl_2) at 0 °C for 15 min. Then the reaction mixture was stirred at room temperature for 33 h. The reaction mixture was then concentrated under reduced pressure and the residue was purified by silica gel column chromatography (PE/EA, 100:1→3:1, v/v) to afford compound V-1 (650 mg, 24.6%) as a colorless oil. ^{1}H NMR (500 MHz, $CDCl_3$) δ_H 7.02 (1H, d, J = 16.5 Hz, H-10), 6.28 (1H, d, J = 16.5 Hz, H-11), 4.35 (1H, m, H-2), 2.77 (1H, dd, J = 16.0, 3.0 Hz, H-5a), 2.39-2.47 (3H, overlap, H-1, 3a, 5b), 2.34 (1H, m, H-4), 2.30 (3H, s, H-13), 1.87 (1H, ddd, J = 13.5, 3.0, 2.0 Hz, H-3b), 1.26 (3H, d, J = 6.5 Hz, H-7), 1.18 (3H, s, H-9). ^{13}C NMR (125 MHz, $CDCl_3$) δ_C 207.1 (C-6), 198.0 (C-12), 149.1 (C-10), 129.9 (C-11), 83.6 (C-8), 81.9 (C-2), 48.5 (C-1), 42.7 (C-5), 37.0 (C-4), 29.7 (C-3), 27.5 (C-13), 23.2 (C-9), 10.8 (C-7). HRESI-MS *m/z*: 239.1277 $[M + H]^+$ (calcd 239.1283 for $C_{13}H_{19}O_4$); 261.1095 $[M + Na]^+$ (calcd 261.1103 for $C_{13}H_{18}O_4Na$).

To a solution of IV-2 (1.8 g, 9.1 mmol) in 50 mL of CH_2Cl_2 was added the solution of 1-triphenylphosphonene-2-acetone (3.5 g, 11.2 mmol, dissolved in 15 mL CH_2Cl_2) at 0 °C for 15 min. Then the reaction mixture was stirred at room temperature for 7 days. The reaction mixture was then concentrated under reduced pressure and the residue was purified by silica gel column chromatography (PE/EA, 100:1→3:1, v/v) to afford compound V-2 (1.1 g, 50.9%) as a

colorless oil. ^{1}H NMR (500 MHz, $CDCl_3$) δ_H 6.49 (1H, d, *J* = 16.5 Hz, H-10), 6.02 (1H, d, *J* = 16.5 Hz, H-11), 4.39 (1H, m, H-2), 2.69 (1H, m, H-5a), 2.54 (1H, dt, *J* = 16.0, 2.5 Hz, H-3a), 2.41 (1H, qd, *J* = 6.5, 4.0 Hz, H-1), 2.34 (1H, dd, *J* = 16.0, 5.0 Hz, 5b), 2.23 (4H, overlap, H-4, 13), 1.90 (1H, ddd, *J* = 13.5, 3.0, 1.5 Hz, H-3b), 1.61 (3H, s, H-9), 1.27 (3H, d, *J* = 6.5 Hz, H-7). ^{13}C NMR (125 MHz, $CDCl_3$) δ_C 206.8 (C-6), 197.4 (C-12), 145.3 (C-10), 127.8 (C-11), 82.6 (C-8), 82.0 (C-2), 48.8 (C-1), 43.7 (C-5), 36.8 (C-4), 28.6 (C-13), 28.2 (C-3), 21.9 (C-9), 11.0 (C-7). HRESI-MS *m/z*: 239.1276 $[M + H]^+$ (calcd 239.1283 for $C_{13}H_{19}O_4$); 261.1096 $[M + Na]^+$ (calcd 261.1103 for $C_{13}H_{18}O_4Na$).

Synthesis of compounds VI-1a, VI-1b, VI-2a and VI-2b

To a solution of ketone V-1 (650 mg, 2.7 mmol) in anhydrous ether (50 mL) was added $LiBH_4$ (572 mg, 27.0 mmol) at -15 °C. After 3 h at -15 °C, the reaction was quenched by addition of water (30 mL). The mixture was then extracted with CH_2Cl_2 (50 mL × 3), dried over anhydrous sodium sulfate, and concentrated under vacuum. The residue was purified by reverse-phase preparative HPLC eluting with acetonitrile and water (the ratio of acetonitrile ranging from 20% to 95%) as the mobile phase to give compound VI-1a (159.6 mg, 24.6%) and VI-1b (206.5 mg, 31.8%) as a colorless oil. Compound VI-1a: ^{1}H NMR (500 MHz, $CDCl_3$) δ_H 6.02 (1H, dd, *J* = 16.5, 1.5 Hz, H-10), 5.75 (1H, dd, *J* = 16.5, 6.0 Hz, H-11), 4.37 (1H, qd, *J* = 6.5, 1.5 Hz, H-12), 4.33 (1H, m, H-2), 2.76 (1H, dt, *J* = 16.0, 2.5 Hz, H-5a), 2.54 (1H, ddd, *J* = 13.5, 7.0, 3.5 Hz, H-3a), 2.40-2.46 (2H, overlap, H-1, 5b), 2.28 (1H, m, H-4), 1.81 (1H, ddd, *J* = 13.5, 3.0, 2.0 Hz, H-3b), 1.31 (3H, d, *J* = 6.5 Hz, H-13), 1.26 (3H, d, *J* = 6.5 Hz, H-7), 1.14 (3H, s, H-9). ^{13}C NMR (125 MHz, $CDCl_3$) δ_C 207.8 (C-6), 134.2 (C-11), 133.1 (C-10), 83.3 (C-8), 81.9 (C-2), 68.4 (C-12), 48.6 (C-1), 43.0 (C-5), 37.3 (C-4), 29.4 (C-3), 23.8 (C-9), 23.4 (C-13), 10.9 (C-7). HRESI-MS *m/z*: 263.1267 $[M + Na]^+$ (calcd 263.1259 for $C_{13}H_{18}O_4Na$). Compound VI-1b: ^{1}H NMR (500 MHz, $CDCl_3$) δ_H 6.02 (1H, dd, *J* = 16.5, 1.5 Hz, H-10), 5.75 (1H, dd, *J* = 16.5, 6.0 Hz, H-11), 4.38 (1H, qd, *J* = 6.5, 1.5 Hz, H-12), 4.33 (1H, m, H-2), 2.76 (1H, dt, *J* = 16.0, 2.5 Hz, H-5a), 2.54 (1H, ddd, *J* = 13.5, 7.0, 3.5 Hz, H-3a), 2.40-2.46 (2H, overlap, H-1, 5b), 2.28 (1H, m, H-4), 1.81 (1H, ddd, *J* = 13.5, 3.0, 2.0 Hz, H-3b), 1.30 (3H, d, *J* = 6.5 Hz, H-13), 1.26 (3H, d, *J* = 6.5 Hz, H-7), 1.14 (3H, s, H-9). ^{13}C NMR (125 MHz, $CDCl_3$) δ_C 207.8 (C-6), 134.2 (C-11), 133.1 (C-10), 83.3 (C-8), 81.9 (C-2), 68.4 (C-12), 48.6 (C-1), 43.0 (C-5), 37.2 (C-4), 29.4 (C-3), 23.9 (C-9), 23.5 (C-13), 10.9 (C-7). HRESI-MS *m/z*: 263.1246 $[M + Na]^+$ (calcd 263.1259 for $C_{13}H_{18}O_4Na$).

To a solution of ketone V-2 (1.1 g, 4.6 mmol) in anhydrous ether (50 mL) was added $LiBH_4$ (966 mg, 46.0 mmol) at -15 °C. After 3 h at -15 °C, the reaction was quenched by addition of

water (30 mL), and then extracted with CH_2Cl_2 (50 mL × 3), dried over anhydrous sodium sulfate, and concentrated under vacuum. The residue was purified by reversed-phase preparative HPLC eluting with acetonitrile in water (20% to 95%) as the mobile phase to give compound VI-2a (348.1 mg, 31.5%) and VI-2b (313.1 mg, 28.3%) as a colorless oil. Compound VI-2a: ^{1}H NMR (500 MHz, $CDCl_3$) δ_H 5.50 (1H, dd, J = 16.0, 2.0 Hz, H-11), 5.44 (1H, d, J = 16.0 Hz, H-10), 4.37 (1H, m, H-2), 4.25 (1H, m, H-12), 2.71 (1H, dd, J = 15.5, 2.5 Hz, H-5a), 2.65 (1H, dt, J = 13.5, 3.0 Hz, H-3a), 2.42 (1H, qd, J = 7.0, 4.0 Hz, H-1), 2.30 (1H, dd, J = 15.5, 4.5 Hz, H-5b), 2.12 (1H, m, H-4), 1.87 (1H, dt, J = 13.5, 1.5 Hz, H-3b), 1.57 (3H, s, H-9), 1.22-1.25 (6H, overlap, H-7, 13). ^{13}C NMR (125 MHz, $CDCl_3$) δ_C 208.6 (C-6), 134.2 (C-11), 130.2 (C-10), 82.4 (C-8), 81.9 (C-2), 68.2 (C-12), 48.7 (C-1), 43.5 (C-5), 37.3 (C-4), 28.4 (C-3), 23.2 (C-13), 22.3 (C-9), 11.0 (C-7). HRESI-MS *m/z*: 263.1244 $[M + Na]^+$ (calcd 263.1259 for $C_{13}H_{18}O_4Na$). Compound VI-2b: ^{1}H NMR (500 MHz, $CDCl_3$) δ_H 5.53 (1H, dd, J = 16.0, 5.0 Hz, H-11), 5.46 (1H, d, J = 16.0 Hz, H-10), 4.37 (1H, m, H-2), 4.28 (1H, m, H-12), 2.72 (1H, dd, J = 15.5, 3.0 Hz, H-5a), 2.66 (1H, ddd, J = 13.0, 7.0, 3.5 Hz, H-3a), 2.40 (1H, qd, J = 6.5, 4.0 Hz, H-1), 2.30 (1H, dd, J = 15.5, 4.5 Hz, H-5b), 2.14 (1H, m, H-4), 1.87 (1H, ddd, J = 13.0, 2.5, 1.5 Hz, H-3b), 1.58 (3H, s, H-9), 1.23-1.26 (6H, overlap, H-7, 13). ^{13}C NMR (125 MHz, $CDCl_3$) δ_C 208.4 (C-6), 134.2 (C-11), 130.0 (C-10), 82.5 (C-8), 81.9 (C-2), 67.8 (C-12), 48.7 (C-1), 43.6 (C-5), 37.3 (C-4), 28.5 (C-3), 23.1 (C-13), 22.4 (C-9), 11.0 (C-7). HRESI-MS *m/z*: 263.1246 $[M + Na]^+$ (calcd 263.1259 for $C_{13}H_{18}O_4Na$).

Synthesis of compounds VII-1a, VII-2a and VII-2b

To a cold solution (0 °C) of compound VI-1a (159.6 mg, 0.66 mmol) in CH_2Cl_2 (20 mL) was added pyridine (261 mg, 3.30 mmol), then 4-nitrophenyl chloroformate (401.67 mg, 1.99 mmol) and the resulting mixture was then stirred for 5 h at room temperature. Then, N-Boc-ethylenediamine (528 mg, 3.30 mmol) and DMAP (241.6 mg, 1.98 mmol) were added and the resulting mixture was stirred at room temperature for additional 5 h. The reaction mixture was then concentrated under reduced pressure and the residue was purified by silica gel column chromatography (PE/EA, 10:1→1:1, v/v) to afford compound VII-1a (210.6 mg, 74.3%) as a colorless oil. Compounds VII-2a and VII-2b were obtained using the same procedures from VI-2a and VI-2b, respectively.

Compound VII-1a, colorless oil, 210.6 mg, 74.3% yield, ^{1}H NMR (500 MHz, $CDCl_3$) δ_H 6.02 (1H, d, J = 16.5 Hz, H-10), 5.70 (1H, dd, J = 16.5, 5.5 Hz, H-11), 5.29 (1H, m, H-12), 4.33 (1H, m, H-2), 3.27 (4H, overlap, H-15, 16), 2.75 (1H, dd, J = 16.0, 2.5 Hz, H-5a), 2.53

(1H, ddd, J = 13.5, 6.0, 3.0 Hz, H-3a), 2.41-2.46 (2H, overlap, H-1, 5b), 2.27 (1H, m, H-4), 1.80 (1H, ddd, J = 13.5, 2.0, 2.0 Hz, H-3b), 1.44 (9H, s, H-19, 20, 21), 1.32 (3H, d, J = 7.0 Hz, H-13), 1.26 (3H, d, J = 7.0 Hz, H-7), 1.13 (3H, s, H-9). ^{13}C NMR (125 MHz, $CDCl_3$) δ_C 208.1 (C-6), 156.4 (C-17), 156.2 (C-14), 134.5 (C-10), 130.2 (C-11), 83.3 (C-8), 81.9 (C-2), 79.6 (C-18), 70.8 (C-12), 48.6 (C-1), 42.9 (C-5), 41.4 (C-15), 40.6 (C-16), 37.3 (C-4), 29.4 (C-3), 28.3 (C-19, 20, 21), 23.8 (C-9), 20.4 (C-13), 10.9 (C-7). HRESI-MS *m/z*: 427.2478 $[M + H]^+$ (calcd 427.2444 for $C_{21}H_{35}N_2O_7$); 449.2298 $[M + Na]^+$ (calcd 449.2264 for $C_{21}H_{34}N_2O_7Na$).

Compound VII-2a, colorless oil, 549.6 mg, 88.9% yield, ^{1}H NMR (500 MHz, $CDCl_3$) δ_H 5.45 (2H, overlap, H-10, 11), 5.20 (1H, m, H-12), 4.38 (1H, m, H-2), 3.24 (4H, overlap, H-15, 16), 2.65 (2H, overlap, H-3a, 5a), 2.40 (1H, m, H-1), 2.29 (2H, dd, J = 16.0, 4.0 Hz, H-5b), 2.11 (1H, m, H-4), 1.86 (1H, brd, J = 13.5, H-3b), 1.58 (3H, s, H-9), 1.43 (9H, s, H-19, 20, 21), 1.27 (6H, overlap, H-7, 13). ^{13}C NMR (125 MHz, $CDCl_3$) δ_C 207.8 (C-6), 156.3 (C-17), 156.1 (C-14), 131.8 (C-10), 130.1 (C-11), 82.3 (C-8), 81.8 (C-2), 79.5 (C-18), 70.8 (C-12), 48.8 (C-1), 43.6 (C-5), 41.2 (C-15), 40.6 (C-16), 37.3 (C-4), 28.4 (C-3), 28.3 (C-19, 20, 21), 22.1 (C-9), 20.4 (C-13), 11.0 (C-7). HRESI-MS *m/z*: 427.2415 $[M + H]^+$ (calcd 427.2444 for $C_{21}H_{35}N_2O_7$); 449.2230 $[M + Na]^+$ (calcd 449.2264 for $C_{21}H_{34}N_2O_7Na$).

Compound VII-2b, colorless oil, 299.1 mg, 53.8% yield, ^{1}H NMR (500 MHz, $CDCl_3$) δ_H 5.50 (1H, d, J = 16.0 Hz, H-10), 5.30 (1H, dd, J = 16.0, 6.5 Hz, H-11), 5.12 (1H, m, H-12), 4.37 (1H, m, H-2), 3.14-3.39 (4H, overlap, H-15, 16), 2.60-2.71 (2H, overlap, H-3a, 5a), 2.38 (1H, qd, J = 6.5, 3.5 Hz, H-1), 2.26 (1H, dd, J = 15.5, 4.0 Hz, H-5b), 2.10 (1H, m, H-4), 1.85 (1H, brd, J = 13.5, H-3b), 1.55 (3H, s, H-9), 1.42 (9H, s, H-19, 20, 21), 1.25 (6H, overlap, H-7, 13). ^{13}C NMR (125 MHz, $CDCl_3$) δ_C 208.3 (C-6), 156.4 (C-17), 156.2 (C-14), 133.0 (C-10), 129.4 (C-11), 82.0 (C-8), 81.8 (C-2), 79.1 (C-18), 71.2 (C-12), 48.7 (C-1), 43.6 (C-5), 41.1 (C-15), 40.3 (C-16), 37.0 (C-4), 28.4 (C-3, 19, 20, 21), 21.9 (C-9), 20.3 (C-13), 10.9 (C-7). HR ESI-MS m/z: 427.2437 $[M + H]^+$ (calcd 427.2444 for $C_{21}H_{35}N_2O_7$); 449.2254 $[M + Na]^+$ (calcd 449.2264 for $C_{21}H_{34}N_2O_7Na$).

Synthesis of compounds VIII-1a, VIII-2a and VIII-2b

To a solution of compound VII-1a (210.6 mg, 0.49 mmol) in anhydrous ether (30 mL) was added $LiBH_4$ (102.9 mg, 4.90 mmol) at -6 °C. After 6 h at -6 °C, the reaction was quenched

by addition of water (30 mL), and then extracted with CH_2Cl_2 (50 mL × 3), dried over anhydrous sodium sulfate, and concentrated under vacuum to afford compound VIII-1a (201.6 mg, 95.2%) as a colorless oil. Compounds VIII-2a and VIII-2b were obtained using the same procedures from VII-2a and VII-2b, respectively.

Compound VIII-1a, colorless oil, 95.2% yield, ^1H NMR (500 MHz, $CDCl_3$) δ_H 5.91 (1H, d, J = 15.6 Hz, H-10), 5.51 (1H, dd, J = 15.6, 6.0 Hz, H-11), 5.29 (1H, m, H-12), 3.92 (1H, m, H-2), 3.83 (1H, dd, J = 4.5, 4.5 Hz, H-6), 3.26 (4H, overlap, H-15, 16), 2.35 (1H, ddd, J = 13.2, 6.6, 3.0 Hz, H-3a), 2.30 (1H, dt, J = 15.0, 2.4 Hz, H-5a), 1.97 (1H, m, H-1), 1.88 (1H, dt, J = 15.0, 4.8 Hz, H-5b), 1.79 (1H, m, H-4), 1.44 (9H, s, H-19, 20, 21), 1.38 (1H, ddd, J = 13.2, 3.0, 1.2 Hz, H-3b), 1.30-1.35 (6H, overlap, H-9, 13), 1.12 (3H, d, J = 7.2 Hz, H-7). ^{13}C NMR (125 MHz, $CDCl_3$) δ_C 156.3 (C-17), 156.2 (C-14), 134.6 (C-10), 130.1 (C-11), 83.1 (C-8), 79.8 (C-2), 79.6 (C-18), 71.0 (C-12), 67.1 (C-6), 41.3 (C-15), 40.7 (C-16), 39.5 (C-1), 34.0 (C-5), 32.6 (C-4), 29.5 (C-3), 28.4 (C-19, 20, 21), 24.5 (C-9), 20.6 (C-13), 13.9 (C-7). HR ESI-MS m/z: 429.2679 $[M + H]^+$ (calcd 429.2601 for $C_{21}H_{37}N_2O_7$); 451.2496 $[M + Na]^+$ (calcd 451.2420 for $C_{21}H_{36}N_2O_7Na$).

Compound VIII-2a, colorless oil, 500.1 mg, 94.2% yield, ^1H NMR (500 MHz, $CDCl_3$) δ_H 5.81 (1H, d, J = 16.0 Hz, H-10), 5.63 (1H, dd, J = 16.0, 6.0 Hz, H-11), 5.24 (1H, m, H-12), 3.97 (1H, brs, H-2), 3.81 (1H, dd, J = 6.0, 5.5 Hz, H-6), 3.23 (4H, overlap, H-15, 16), 2.51 (1H, ddd, J = 13.0, 7.0, 3.5 Hz, H-3a), 2.19 (1H, brd, J = 15.0 Hz, H-5a), 1.97 (1H, qd, J = 7.0, 3.0 Hz, H-1), 1.82 (1H, dt, J = 15.0, 5.0 Hz, H-5b), 1.72 (1H, m, H-4), 1.61 (3H, s, H-9), 1.42 (10H, overlap, H-3b, 19, 20, 21), 1.28 (3H, d, J = 6.5 Hz, H-13), 1.11 (3H, d, J = 6.5 Hz, H-7). ^{13}C NMR (125 MHz, $CDCl_3$) δ_C 156.4 (C-17), 156.1 (C-14), 133.4 (C-10), 130.6 (C-11), 81.8 (C-8), 79.8 (C-2), 79.4 (C-18), 70.7 (C-12), 67.1 (C-6), 41.2 (C-15), 40.5 (C-16), 39.7 (C-1), 34.8 (C-5), 33.3 (C-4), 28.8 (C-3), 28.4 (C-19, 20, 21), 22.7 (C-9), 20.3 (C-13), 13.9 (C-7). HRESI-MS *m/z*: 429.2659 $[M + H]^+$ (calcd 429.2601 for $C_{21}H_{37}N_2O_7$); 451.2478 $[M + Na]^+$ (calcd 451.2420 for $C_{21}H_{36}N_2O_7Na$).

Compound VIII-2b, colorless oil, 250.0 mg, 87.8% yield, ^1H NMR (500 MHz, $CDCl_3$) δ_H 5.80 (1H, d, J = 16.0 Hz, H-10), 5.51 (1H, dd, J = 16.0, 6.5 Hz, H-11), 5.21 (1H, m, H-12), 3.97 (1H, brs, H-2), 3.74 (1H, dd, J = 5.5, 5.0 Hz, H-6), 3.27 (2H, m, H-15), 3.13 (2H, m, 16), 2.51 (1H, ddd, J = 13.0, 6.5, 3.0 Hz, H-3a), 2.15 (1H, brd, J = 15.0 Hz, H-5a), 1.97 (1H, m, H-

1), 1.80 (1H, dt, J = 15.0, 5.0 Hz, H-5b), 1.70 (1H, m, H-4), 1.59 (3H, s, H-9), 1.43 (10H, overlap, H-3b, 19, 20, 21), 1.27 (3H, d, J = 6.5 Hz, H-13), 1.11 (3H, d, J = 7.0 Hz, H-7). ^{13}C NMR (125 MHz, $CDCl_3$) δ_C 156.6 (C-17), 155.9 (C-14), 135.0 (C-10), 129.6 (C-11), 81.7 (C-8), 79.7 (C-2), 79.0 (C-18), 70.7 (C-12), 66.9 (C-6), 41.2 (C-15), 39.8 (C-16), 39.6 (C-1), 34.5 (C-5), 33.4 (C-4), 28.7 (C-3), 28.4 (C-19, 20, 21), 22.9 (C-9), 20.2 (C-13), 13.8 (C-7). HRESI-MS *m/z*: 429.2649 [M + H]$^+$ (calcd 429.2601 for $C_{21}H_{37}N_2O_7$); 451.2468 [M + Na]$^+$ (calcd 451.2420 for $C_{21}H_{36}N_2O_7Na$).

Synthesis of compounds IX-1a, IX-2a and IX-2b

To a cold solution (0 °C) of compound VIII-1a (150.0 mg, 0.35 mmol) in CH_2Cl_2 (15 mL) was added pyridine (140.97 mg, 1.75 mmol), then 4-nitrophenyl chloroformate (211.64 mg, 1.05 mmol) and the resulting mixture was stirred for 4 days while being allowed to warm to room temperature. The reaction mixture was then concentrated under reduced pressure and the residue was purified by silica gel column chromatography (PE/EA, 50:1→1:1, v/v) to afford the intermediate (220.1 mg). Then, to a solution of the activated intermediate (220.1 mg, 0.37 mmol) was added DMAP (90.28 mg, 0.74 mmol), then disperse red (139.1 mg, 0.44 mmol) and the resulting mixture was stirred at room temperature for 5 h. The reaction mixture was then concentrated under reduced pressure and the residue was purified by silica gel column chromatography (PE/EA, 50:1→1:1, v/v) to afford compound IX-1a (100 mg, 35.1%) as a red solid. Compounds IX-2a and IX-2b were obtained using the same procedures from VIII-2a and VIII-2b, respectively.

Compound IX-1a, red solid, 100 mg, 35.1% yield, ^{1}H NMR (600 MHz, $CDCl_3$) δ_H 8.30 (2H, d, J = 8.4 Hz, H-35, 37), 7.92 (4H, d, J = 8.4 Hz, H-28, 32, 34, 38), 6.82 (2H, d, J = 8.4 Hz, H-29, 31), 5.88 (1H, d, J = 16.2 Hz, H-10), 5.72 (1H, dd, J = 16.2, 5.4 Hz, H-11), 5.27 (1H, m, H-12), 5.16 (1H, dd, J = 6.0 Hz, H-6), 3.84 (1H, m, H-2), 3.55-3.60 (2H, overlap, H-24), 3.48-3.54 (2H, overlap, H-25), 3.40 (2H, m, H-23), 3.26 (4H, overlap, H-15, 16), 2.34 (1H, brd, J = 18.0 Hz, H-3a), 2.21 (1H, brd, J = 15.6 Hz, H-5a), 2.03 (1H, m, H-1), 1.95 (1H, dt, J = 16.2, 4.2 Hz, H-5b), 1.74 (1H, brs, H-4), 1.42 (9H, s, H-19, 20, 21), 1.35 (3H, brd, J = 12.6 Hz, H-3b), 1.30 (3H, brs, H-13), 1.23 (3H, t, J = 6.6 Hz, H-26), 1.13 (3H, s, H-9), 1.06 (3H, d, J = 7.2 Hz, H-7). ^{13}C NMR (150 MHz, $CDCl_3$) δ_C157.1 (C-22), 156.3 (C-17), 156.1 (C-14, 36), 151.6

(C-27), 147.3 (C-33), 143.5 (C-30), 135.5 (C-10), 129.6 (C-11), 126.8 (C-28, 32), 124.7 (C-35, 37), 122.4 (C-34, 38), 111.7 (C-29, 31), 81.8 (C-8), 79.5 (C-18), 77.5 (C-2), 71.1 (C-12), 69.2 (C-6), 49.9 (C-24), 45.8 (C-25), 41.3 (C-15), 40.7 (C-16), 38.4 (C-23), 37.7 (C-1), 31.9 (C-4), 31.5 (C-5), 29.3 (C-3), 28.3 (C-19, 20, 21), 24.4 (C-9), 20.6 (C-13), 13.0 (C-7), 12.3 (C-26). HRESI-MS *m/z*: 768.3905 $[M + H]^+$ (calcd 768.3932 for $C_{38}H_{54}N_7O_{10}$); 790.3701 $[M + Na]^+$ (calcd 790.3752 for $C_{38}H_{53}N_7O_{10}Na$).

Compound IX-2a, red solid, 356.1 mg, 50.7% yield, ^{1}H NMR (600 MHz, $CDCl_3$) δ_H 8.32 (2H, d, *J* = 9.0 Hz, H-35, 37), 7.96 (4H, overlap, H-28, 32, 34, 38), 6.90 (2H, brs, H-29, 31), 5.71 (1H, d, *J* = 16.2 Hz, H-10), 5.57 (1H, dd, *J* = 16.2, 5.4 Hz, H-11), 5.20 (1H, m, H-12), 5.11 (1H, m Hz, H-6), 3.92 (1H, m, H-2), 3.59-3.69 (2H, overlap, H-24), 3.45-3.59 (3H, overlap, H-23a, 25), 3.34 (1H, m, H-23b), 3.22 (4H, overlap, H-15, 16), 2.51 (1H, brd, *J* = 12.6 Hz, H-3a), 2.02-2.15 (2H, overlap, H-1, 5a), 1.86 (1H, dt, *J* = 16.2, 4.8 Hz, H-5b), 1.69 (1H, brs, H-4), 1.55 (3H, s, H-9), 1.42 (10H, overlap, H-3b, 19, 20, 21), 1.24-1.29 (6H, overlap, H-13, 26), 1.06 (3H, d, *J* = 6.6 Hz, H-7). ^{13}C NMR (150 MHz, $CDCl_3$) δ_C 157.3 (C-22), 156.6 (C-17), 156.1 (C-14), 151.7 (C-27), 155.8 (C-36), 147.2 (C-33), 143.4 (C-30), 134.7 (C-10), 129.3 (C-11), 127.1 (C-28, 32), 124.7 (C-35, 37), 122.3 (C-34, 38), 112.1 (C-29, 31), 81.3 (C-8), 79.6 (C-18), 77.7 (C-2), 70.8 (C-12), 69.4 (C-6), 50.2 (C-24), 46.1 (C-25), 41.6 (C-15), 40.5 (C-16), 38.4 (C-23), 37.9 (C-1), 32.9 (C-4), 32.3 (C-5), 28.6 (C-3), 28.4 (C-19, 20, 21), 23.0 (C-9), 20.5 (C-13), 12.9 (C-7), 12.4 (C-26). HRESI-MS *m/z*: 768.3896 $[M + H]^+$ (calcd 768.3932 for $C_{38}H_{54}N_7O_{10}$); 790.3712 $[M + Na]^+$ (calcd 790.3752 for $C_{38}H_{53}N_7O_{10}Na$).

Compound IX-2b, red solid, 214.3 mg, 54.6% yield, ^{1}H NMR (600 MHz, $CDCl_3$) δ_H 8.31 (2H, d, *J* = 8.4 Hz, H-35, 37), 7.96 (4H, overlap, H-28, 32, 34, 38), 6.91 (2H, brd, H-29, 31), 5.66-5.79 (2H, overlap, H-10, 11), 5.14 (2H, overlap, H-6, 12), 3.92 (1H, m, H-2), 3.46-3.70 (5H, overlap, H-23a, 24, 25), 3.40 (1H, brs, H-23b), 3.22 (4H, overlap, H-15, 16), 2.52 (1H, brd, *J* = 12.0 Hz, H-3a), 2.02-2.12 (2H, overlap, H-1, 5a), 1.86 (1H, dt, *J* = 15.6, 5.4 Hz, H-5b), 1.66 (1H, brs, H-4), 1.54 (3H, s, H-9), 1.42 (10H, overlap, H-3b, 19, 20, 21), 1.23-1.30 (6H, overlap, H-13, 26), 1.08 (3H, d, *J* = 6.6 Hz, H-7). ^{13}C NMR (150 MHz, $CDCl_3$) δ_C 157.4 (C-22), 156.5 (C-17), 156.4 (C-14), 155.8 (C-36), 152.3 (C-27), 147.2 (C-33), 143.0 (C-30), 134.4 (C-10), 129.9 (C-11), 127.5 (C-28, 32), 124.7 (C-35, 37), 122.2 (C-34, 38), 112.3 (C-29, 31), 80.7 (C-8), 79.5 (C-18), 77.8 (C-2), 70.7 (C-12), 69.2 (C-6), 50.2 (C-24), 46.1 (C-25), 41.7 (C-

15), 40.3 (C-16), 38.4 (C-23), 38.0 (C-1), 32.6 (C-4), 32.5 (C-5), 28.7 (C-3), 28.4 (C-19, 20, 21), 22.4 (C-9), 19.2 (C-13), 12.9 (C-7), 12.4 (C-26). HRESI-MS *m/z*: 768.3892 $[M + H]^+$ (calcd 768.3932 for $C_{38}H_{54}N_7O_{10}$); 790.3718 $[M + Na]^+$ (calcd 790.3752 for $C_{38}H_{53}N_7O_{10}Na$).

Synthesis of the fluorescent probe X

To a solution of compound IX-1a (80 mg，0.10 mmol) in CH_2Cl_2 (8 mL), 2 mL of TFA was added. The reaction mixture was stirred at room temperature for 20 h. After completion, the solution was concentrated under reduced pressure and the residue was dissolved in CH_2Cl_2. Then dye (38.9 mg，0.1 mmol) and trimethylamine (41.7 μL) were added and the reaction mixture was stirred at room temperature for 6 h. After completion, the solution was concentrated under reduced pressure and the residue was purified by column chromatography (DCM/MeOH, 20:1, v/v) to afford fluorescent probe X-1a as red solid. Fluorescent probes X-2a and X-2b were obtained using the same procedures from IX-2a and IX-2b, respectively.

Fluorescent probe X-1a, red solid, 44.9 mg, 46.7% yield, ^{1}H NMR (600 MHz, $CDCl_3$) δ_H 8.30 (2H, d, *J* = 8.4 Hz, H-44, 46), 7.12 (4H, overlap, H-37, 41, 43, 47), 7.09 (1H, s, H-25), 7.02 (2H, d, *J* = 8.4 Hz, H-38, 40), 6.88 (1H, d, *J* = 3.6 Hz, H-27), 6.28 (1H, d, *J* = 3.6 Hz, H-28), 6.12 (1H, s, H-22), 5.90 (1H, d, *J* = 15.6 Hz, H-10), 5.70 (1H, dd, *J* = 16.2, 4.2 Hz, H-11), 5.26 (1H, m, H-12), 5.14 (1H, m, H-6), 3.83 (1H, m, H-2), 3.66-3.73 (2H, overlap, H-33), 3.58-3.66 (2H, overlap, H-34), 3.47 (2H, m, H-32), 3.32 (2H, dd, *J* = 4.8 Hz, H-16), 3.26 (2H, dd, *J* = 7.2 Hz, H-19), 3.21 (2H, dd, *J* = 4.8 Hz, H-15), 2.65 (2H, dd, *J* = 7.2 Hz, H-18), 2.55 (3H, s, H-29), 2.34 (1H, m, H-3a), 2.25 (3H, s, H-30), 2.18 (1H, d, *J* = 15.6 Hz, H-5a), 2.02 (1H, m, H-1), 1.94 (1H, dt, *J* = 15.6, 4.8 Hz, H-5b), 1.74 (1H, brs, H-4), 1.26-1.36 (7H, overlap, H-3b, 13, 35), 1.13 (3H, s, H-9), 1.03 (3H, d, *J* = 7.2 Hz, H-7). ^{13}C NMR (150 MHz, $CDCl_3$) δ_C 172.6 (C-17), 160.5 (C-21), 157.1 (C-31), 157.0 (C-20), 156.8 (C-45), 156.3 (C-14), 152.5 (C-36), 147.0 (C-42), 144.1 (C-24), 142.2 (C-39), 135.4 (C-10), 135.2 (C-23), 133.2 (C-26), 129.7 (C-11), 128.2 (C-27), 124.9 (C-44, 46), 123.9 (C-25), 121.4 (C-37, 41), 121.1 (C-43, 47), 120.6 (C-22), 117.4 (C-28), 114.0 (C-38, 40), 81.8 (C-8), 78.0 (C-2), 70.9 (C-12), 69.5 (C-6), 50.9 (C-33), 47.2 (C-34), 41.0 (C-15), 39.9 (C-16), 38.4 (C-32), 37.7 (C-1), 35.7 (C-18), 31.9 (C-4), 31.5 (C-5), 29.3 (C-3), 24.8 (C-19), 24.4 (C-9), 20.6 (C-13), 15.0 (C-29), 13.0 (C-7), 12.4

(C-35), 11.3 (C-30). HRESI-MS *m/z*: 942.4488 $[M + H]^+$ (calcd 942.4497 for $C_{47}H_{59}BF_2N_9O_9$); 964.4301 $[M + Na]^+$ (calcd 964.4316 for $C_{47}H_{58}BF_2N_9O_9Na$).

Fluorescent probe X-2a, red solid, 113.1 mg, 30.8% yield, ^{1}H NMR (600 MHz, $CDCl_3$) δ_H 8.30 (2H, d, *J* = 9.0 Hz, H-44, 46), 7.89 (2H, d, *J* = 9.0 Hz, H-43, 47), 7.87 (2H, d, *J* = 9.0 Hz, H-37, 41), 7.06 (1H, s, H-25), 6.86 (1H, d, *J* = 3.6 Hz, H-27), 6.82 (2H, d, *J* = 9.0 Hz, H-38, 40), 6.28 (1H, d, *J* = 3.6 Hz, H-28), 6.10 (1H, s, H-22), 5.70 (1H, d, *J* = 16.2 Hz, H-10), 5.55 (1H, dd, *J* = 16.2, 4.8 Hz, H-11), 5.18 (1H, m, H-12), 5.09 (1H, m, H-6), 3.91 (1H, m, H-2), 3.54-3.60 (2H, overlap, H-33), 3.45-3.54 (3H, overlap, H-34, 32a), 3.29-3.38 (2H, overlap, H-16a, 32b), 3.21-3.29 (3H, overlap, H-16b, 19), 3.09-3.20 (2H, overlap, H-15), 2.64 (2H, dd, *J* = 7.2 Hz, H-18), 2.54 (3H, s, H-29), 2.48 (1H, d, *J* = 12.6 Hz, H-3a), 2.23 (3H, s, H-30), 2.12 (1H, d, *J* = 12.6 Hz, H-5a), 2.05 (1H, m, H-1), 1.84 (1H, m, H-5b), 1.66 (1H, brs, H-4), 1.54 (3H, s, H-9), 1.43 (1H, m, H-3b), 1.21-1.25 (6H, overlap, H-13, 35), 1.06 (3H, d, *J* = 6.0 Hz, H-7). ^{13}C NMR (150 MHz, $CDCl_3$) δ_C 173.0 (C-17), 160.3 (C-21), 157.3 (C-31), 157.0 (C-20), 156.8 (C-45), 156.1 (C-14), 151.4 (C-36), 147.2 (C-42), 144.0 (C-24), 143.6 (C-39), 135.1 (C-23), 134.5 (C-10), 133.3 (C-26), 129.4 (C-11), 128.2 (C-27), 126.4 (C-37, 41), 124.6 (C-44, 46), 123.8 (C-25), 122.5 (C-43, 47), 120.5 (C-22), 117.3 (C-28), 111.4 (C-38, 40), 81.4 (C-8), 77.7 (C-2), 70.6 (C-12), 69.3 (C-6), 49.9 (C-33), 45.6 (C-34), 41.5 (C-15), 39.5 (C-16), 38.3 (C-32), 37.9 (C-1), 35.6 (C-18), 32.9 (C-4), 32.2 (C-5), 28.6 (C-3), 24.8 (C-19), 23.1 (C-9), 20.6 (C-13), 14.9 (C-29), 13.0 (C-7), 12.3 (C-35), 11.3 (C-30). HRESI-MS *m/z*: 942.4464 $[M + H]^+$ (calcd 942.4497 for $C_{47}H_{59}BF_2N_9O_9$); 964.4301 $[M + Na]^+$ (calcd 964.4316 for $C_{47}H_{58}BF_2N_9O_9Na$).

Fluorescent probe X-2b, red solid, 91.2 mg, 42.1% yield, ^{1}H NMR (600 MHz, $CDCl_3$) δ_H 8.30 (2H, d, *J* = 8.4 Hz, H-44, 46), 7.89 (2H, d, *J* = 8.4 Hz, H-43, 47), 7.85 (2H, d, *J* = 8.4 Hz, H-37, 41), 7.06 (1H, s, H-25), 6.86 (1H, d, *J* = 3.6 Hz, H-27), 6.81 (2H, d, *J* = 8.4 Hz, H-38, 40), 6.26 (1H, d, *J* = 3.6 Hz, H-28), 6.09 (1H, s, H-22), 5.70 (1H, d, *J* = 16.2 Hz, H-10), 5.61 (1H, dd, *J* = 16.2, 6.0 Hz, H-11), 5.21 (1H, m, H-12), 5.11 (1H, m, H-6), 3.90 (1H, m, H-2), 3.42-3.61 (5H, overlap, H-33, 34, 32a), 3.31-3.38 (2H, overlap, H-16a, 32b), 3.24-3.28 (2H, dd, *J* = 7.2 Hz, H-19), 3.14-3.23 (2H, overlap, H-15a, 16b), 3.09 (1H, m, H-15b), 2.64 (2H, dd, *J* = 7.2 Hz, H-18), 2.53 (3H, s, H-29), 2.48 (1H, d, *J* = 12.6 Hz, H-3a), 2.22 (3H, s, H-30), 2.07 (1H, overlap, H-5a), 2.00-2.05 (1H, overlap, H-1), 1.81 (1H, m, H-5b), 1.64 (1H, brs, H-4), 1.53 (3H, s, H-9), 1.41 (1H, m, H-3b), 1.20-1.25 (6H, overlap, H-13, 35), 1.06 (3H, d, *J* = 6.6

Hz, H-7). ^{13}C NMR (150 MHz, $CDCl_3$) δ_C 172.6 (C-17), 160.2 (C-21), 157.4 (C-31), 157.3 (C-20), 156.8 (C-45), 156.4 (C-14), 151.5 (C-36), 147.2 (C-42), 143.9 (C-24), 143.6 (C-39), 135.1 (C-23), 134.8 (C-10), 133.3 (C-26), 129.9 (C-11), 128.3 (C-27), 126.3 (C-37, 41), 124.6 (C-44, 46), 123.8 (C-25), 122.5 (C-43, 47), 120.4 (C-22), 117.4 (C-28), 111.3 (C-38, 40), 81.7 (C-8), 77.8 (C-2), 70.8 (C-12), 69.3 (C-6), 49.8 (C-33), 45.6 (C-34), 41.4 (C-15), 39.3 (C-16), 38.4 (C-32), 38.0 (C-1), 35.7 (C-18), 32.6 (C-4), 32.4 (C-5), 28.6 (C-3), 24.8 (C-19), 22.2 (C-9), 19.4 (C-13), 14.9 (C-29), 13.0 (C-7), 12.3 (C-35), 11.3 (C-30). HR ESI-MS m/z: 942.4497 $[M + H]^+$ (calcd 942.4497 for $C_{47}H_{59}BF_2N_9O_9$); 964.4302 $[M + Na]^+$ (calcd 964.4316 for $C_{47}H_{58}BF_2N_9O_9Na$).

Culture of single crystal of IV-1

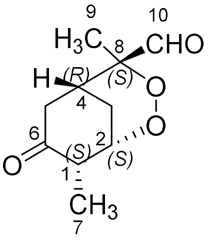

IV-1

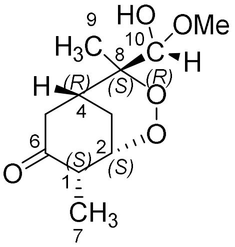

IV-1-hemiacetal

The intermediate of IV-1 (5 mg) was dissolved in methanol, slow evaporation of the solution at room temperature resulted in high-quality, single crystals, and the crystal structure of IV-1-hemiacetal allowed unambiguous assignment of the absolute configuration of IV-1 based on the Flack parameter (0.09(11)) for anomalous dispersion with Cu Kα radiation (Fig. S168, Tables S42 and S43).

Preparation of the (R)- and (S)-MTPA ester derivatives of VI-1a, VI-2a, VI-2b, VIII-2a and VIII-2b

Compound VI-1a (2.4 mg, 0.01 mmol) was transferred into a clean NMR tube and was dried completely under the vacuum of a diaphragm pump. Deuterated pyridine (0.5 mL) and (*S*)-(+)-α-methoxy-α- (trifluoromethyl) phenylacetyl chloride (5.05 mg, 0.02mmol) were added into the NMR tube immediately, and then the NMR tube was shaken carefully to mix the sample and MTPA chloride evenly. The reaction NMR tube was permitted to stand at room temperature, and the reaction was found to be complete after 12 h. The solution was concentrated under reduced pressure, and then purified by reversed-phase preparative HPLC eluting with acetonitrile in water (20% to 95%) as the mobile phase to yield (*R*)-MTPA ester (VI-1aR). In the manner described for VI-1a, another portion of compound VI-1a (2.4 mg, 0.01

mmol) was reacted in a second NMR tube with (*R*)-(-)-α-methoxy-α- (trifluoromethyl) phenylacetyl chloride (5.05 mg, 0.02mmol) at room temperature for 12 h using deuterated pyridine (0.5 mL) as a solvent to afford the (*S*)-MTPA derivative (VI-1aS) of VI-1a. ^{1}H NMR spectra of VI-1aR and VI-1aS in $CDCl_3$ was recorded, and data were assigned on the basis of the correlations of its ^{1}H-^{1}H COSY spectra. (*S*)-MTPA esters (VI-2aS, VI-2bS, VIII-2aS, and VIII-2bS) and (*R*)-MTPA esters (VI-2aR, VI-2bR, VIII-2aR, and VIII-2bR) were obtained using the same procedures for preparing (*S*)- and (*R*)-MTPA esters of VI-1a.

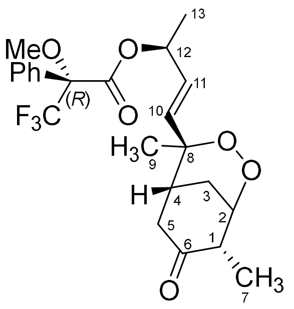

VI-1aR
(*R*)-MTPA ester

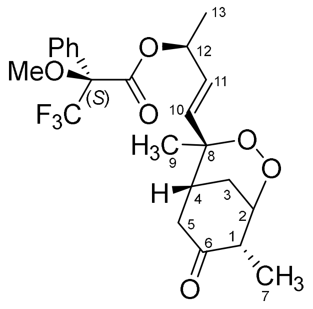

VI-1aS
(*S*)-MTPA ester

(*R*)-MTPA ester of VI-1a (VI-1aR) ^{1}H NMR (500 MHz, $CDCl_3$) δ_H 7.512 (2H, dd, *J* = 7.5, 1.5 Hz, Ar-H), 7.395 (3H, m, Ar-H), 6.008 (1H, d, *J* = 16.0 Hz, H-10), 5.719 (1H, dd, *J* = 16.0, 7.5 Hz, H-11), 5.622 (1H, qd, *J* = 6.5, 1.0 Hz, H-12), 4.295 (1H, m, H-2), 3.575 (3H, s, MTPA-OCH_3), 2.752 (1H, dt, *J* = 15.5, 3.0 Hz, H-5a), 2.406-2.472 (3H, overlap, H-1, 3a, 5b), 2.204 (1H, m, H-4), 1.801 (1H, ddd, *J* = 13.5, 3.5, 2.0 Hz, H-3b), 1.457 (3H, d, *J* = 6.5 Hz, H-13), 1.272 (3H, d, *J* = 7.0 Hz, H-7), 1.107 (3H, s, H-9).

(*S*)-MTPA ester of VI-1a (VI-1aS) ^{1}H NMR (500 MHz, $CDCl_3$) δ_H 7.530 (2H, dd, *J* = 8.0, 1.5 Hz, Ar-H), 7.382 (3H, m, Ar-H), 6.057 (1H, d, *J* = 16.0 Hz, H-10), 5.820 (1H, dd, *J* = 16.0, 7.5 Hz, H-11), 5.598 (1H, qd, *J* = 5.5, 1.0 Hz, H-12), 4.243 (1H, m, H-2), 3.544 (3H, s, MTPA-OCH_3), 2.750 (1H, dt, *J* = 16.0, 2.5 Hz, H-5a), 2.390-2.468 (3H, overlap, H-1, 3a, 5b), 2.232 (1H, m, H-4), 1.791 (1H, ddd, *J* = 13.5, 3.5, 2.0 Hz, H-3b), 1.393 (3H, d, *J* = 6.5 Hz, H-13), 1.258 (3H, d, *J* = 7.0 Hz, H-7), 1.127 (3H, s, H-9).

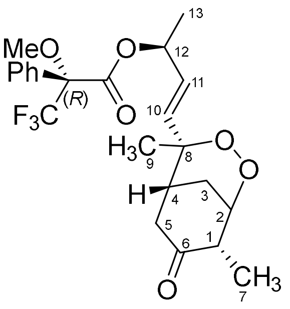

VI-2aR
(R)-MTPA ester

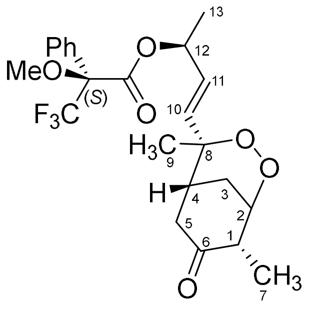

VI-2aS
(S)-MTPA ester

(*R*)-MTPA ester of VI-2a (VI-2aR) ^{1}H NMR (500 MHz, $CDCl_3$) δ_H 7.504 (2H, dd, J = 7.0, 2.5 Hz, Ar-H), 7.383 (3H, m, Ar-H), 5.561 (1H, m, H-12), 5.439 (1H, d, J = 16.5 Hz, H-10), 5.367 (1H, dd, J = 16.5, 6.0 Hz, H-11), 4.371 (1H, m, H-2), 3.556 (3H, s, MTPA-OCH_3), 2.643 (1H, m, H-5a), 2.572 (1H, dt, J = 16.0, 2.5 Hz, H-3a), 2.392 (1H, qd, J = 7.0, 3.5 Hz, H-1), 2.281 (1H, dd, J = 15.5, 4.5 Hz, H-5b), 2.035 (1H, m, H-4), 1.862 (1H, ddd, J = 13.5, 3.0, 1.5 Hz, H-3b), 1.522 (3H, s, H-9), 1.403 (3H, d, J = 6.5 Hz, H-13), 1.260 (3H, d, J = 6.5 Hz, H-7).

(*S*)-MTPA ester of VI-2a (VI-2aS) ^{1}H NMR (500 MHz, $CDCl_3$) δ_H 7.504 (2H, dd, J = 6.5, 3.0 Hz, Ar-H), 7.390 (3H, m, Ar-H), 5.592 (1H, d, J = 16.0 Hz, H-10), 5.547 (1H, m, H-12), 5.489 (1H, dd, J = 16.0, 6.5 Hz, H-11), 4.388 (1H, m, H-2), 3.528 (3H, s, MTPA-OCH_3), 2.657 (1H, m, H-5a), 2.604 (1H, dt, J = 16.0, 2.5 Hz, H-3a), 2.402 (1H, qd, J = 6.5, 3.5 Hz, H-1), 2.300 (1H, dd, J = 15.5, 4.5 Hz, H-5b), 2.093 (1H, m, H-4), 1.873 (1H, ddd, J = 14.0, 3.5, 2.0 Hz, H-3b), 1.564 (3H, s, H-9), 1.354 (3H, d, J = 6.5 Hz, H-13), 1.271 (3H, d, J = 7.0 Hz, H-7).

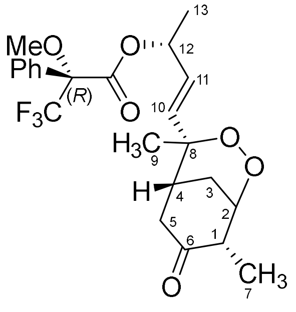

VI-2bR
(R)-MTPA ester

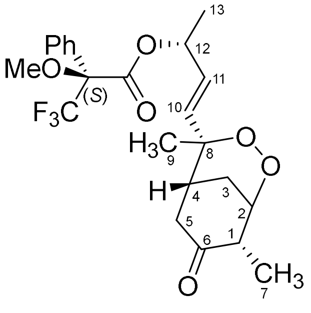

VI-2bR
(S)-MTPA ester

(*R*)-MTPA ester of VI-2b (VI-2bR) ^{1}H NMR (500 MHz, $CDCl_3$) δ_H 7.535 (2H, dd, J = 7.5, 2.0 Hz, Ar-H), 7.406 (3H, m, Ar-H), 5.604 (1H, d, J = 15.5 Hz, H-10), 5.500 (1H, m, H-

12), 5.447 (1H, dd, J = 15.5, 7.0 Hz, H-11), 4.380 (1H, m, H-2), 3.599 (3H, s, MTPA-OCH_3), 2.596-2.682 (2H, overlap, H-5a, 3a), 2.393 (1H, qd, J = 7.0, 3.5 Hz, H-1), 2.282 (1H, dd, J = 15.5, 4.5 Hz, H-5b), 2.119 (1H, m, H-4), 1.877 (1H, ddd, J = 13.5, 3.5, 2.0 Hz, H-3b), 1.564 (3H, s, H-9), 1.317 (3H, d, J = 6.5 Hz, H-13), 1.268 (3H, d, J = 7.0 Hz, H-7).

(*S*)-MTPA ester of VI-2b (VI-2bS) ^{1}H NMR (500 MHz, $CDCl_3$) δ_H 7.544 (2H, dd, J = 7.0, 1.5 Hz, Ar-H), 7.402 (3H, m, Ar-H), 5.523 (1H, m, H-12), 5.396 (1H, overlap, H-10), 5.389 (1H, overlap, H-11), 4.356 (1H, m, H-2), 3.574 (3H, s, MTPA-OCH_3), 2.624 (1H, m, H-5a), 2.562 (1H, m, H-3a), 2.377 (1H, qd, J = 6.5, 3.5 Hz, H-1), 2.232 (1H, dd, J = 16.0, 4.5 Hz, H-5b), 2.024 (1H, m, H-4), 1.844 (1H, ddd, J = 13.5, 3.0, 1.5 Hz, H-3b), 1.499 (3H, s, H-9), 1.401 (3H, d, J = 7.0 Hz, H-13), 1.255 (3H, d, J = 7.0 Hz, H-7).

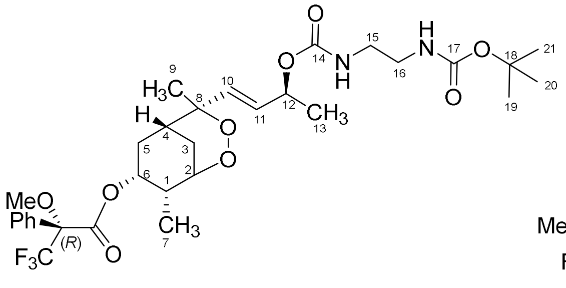
VIII-2aR
(*R*)-MTPA ester

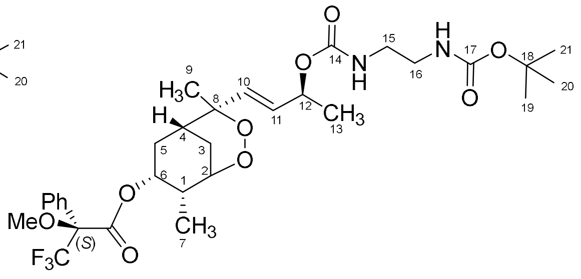
VIII-2aS
(*S*)-MTPA ester

(*R*)-MTPA ester of VIII-2a (VIII-2aR) ^{1}H NMR (500 MHz, $CDCl_3$) δ_H 7.564 (2H, dd, J = 6.5, 3.0 Hz, Ar-H), 7.421 (3H, m, Ar-H), 5.672 (1H, d, J = 16.5 Hz, H-10), 5.541 (1H, overlap, H-6), 5.530 (1H, overlap, H-11), 4.910 (1H, m, H-12), 3.935 (1H, m, H-2), 3.646 (3H, s, MTPA-OCH_3), 3.234 (4H, overlap, H-15, 16), 2.536 (1H, m, H-3a), 2.195 (1H, m, H-1), 2.095 (1H, brd, H-5a), 1.945 (1H, dt, J = 16.0, 6.0 Hz, H-5b), 1.658 (1H, m, H-4), 1.573 (3H, s, H-9), 1.455 (1H, m, H-3b), 1.433 (9H, s, H-19, 20, 21), 1.144 (3H, d, J = 7.0 Hz, H-7), 1.086 (3H, d, J = 6.5 Hz, H-13).

(*S*)-MTPA ester of VIII-2a (VIII-2aS) ^{1}H NMR (500 MHz, $CDCl_3$) δ_H 7.578 (2H, dd, J = 6.0, 2.0 Hz, Ar-H), 7.437 (3H, m, Ar-H), 5.932 (1H, d, J = 16.0 Hz, H-10), 5.672 (1H, dd, J = 7.0 Hz, H-6), 5.589 (1H, dd, J = 16.0, 5.0 Hz, H-11), 5.208 (1H, m, H-12), 3.917 (1H, m, H-2), 3.517 (3H, s, MTPA-OCH_3), 3.254 (4H, overlap, H-15, 16), 2.549 (1H, ddd, J = 13.5, 7.5, 4.0 Hz, H-3a), 2.231 (1H, brd, H-5a), 2.186 (1H, qd, J = 7.0, 3.5 Hz, H-1), 2.007 (1H, ddd, J = 16.5, 7.0, 5.0 Hz, H-5b), 1.706 (1H, m, H-4), 1.601 (3H, s, H-9), 1.471 (1H, m, H-3b), 1.430 (9H, s, H-19, 20, 21), 1.241 (3H, d, J = 6.0 Hz, H-13), 1.037 (3H, d, J = 7.0 Hz, H-7).

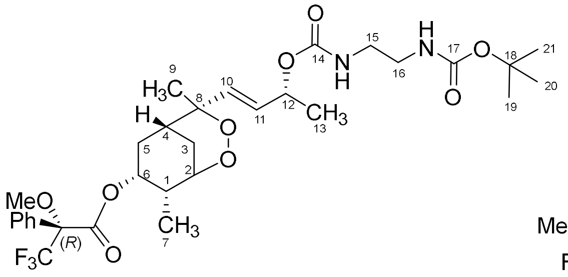

VIII-2bR
(*R*)-MTPA ester

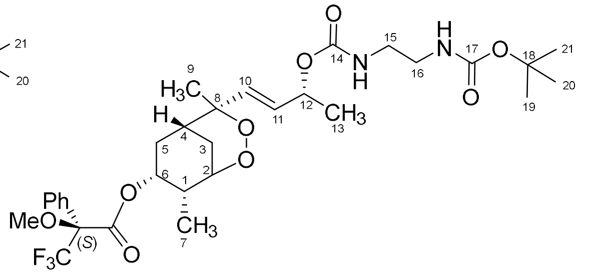

VIII-2bS
(*S*)-MTPA ester

(*R*)-MTPA ester of VIII-2b (VIII-2bR) ^{1}H NMR (500 MHz, $CDCl_3$) δ_H 7.567 (2H, dd, *J* = 6.5, 2.0 Hz, Ar-H), 7.420 (3H, m, Ar-H), 5.671 (1H, d, *J* = 16.5 Hz, H-10), 5.546 (1H, overlap, H-6), 5.522 (1H, overlap, H-11), 4.916 (1H, m, H-12), 3.935 (1H, m, H-2), 3.648 (3H, s, MTPA-OCH_3), 3.230 (4H, overlap, H-15, 16), 2.537 (1H, m, H-3a), 2.197 (1H, qd, *J* = 7.0, 3.5 Hz, H-1), 2.100 (1H, brd, H-5a), 1.947 (1H, dt, *J* = 16.5, 5.5 Hz, H-5b), 1.666 (1H, m, H-4), 1.574 (3H, s, H-9), 1.461 (1H, m, H-3b), 1.438 (9H, s, H-19, 20, 21), 1.146 (3H, d, *J* = 7.0 Hz, H-7), 1.091 (3H, d, *J* = 6.5 Hz, H-13).

(*S*)-MTPA ester of VIII-2b (VIII-2bS) ^{1}H NMR (500 MHz, $CDCl_3$) δ_H 7.578 (2H, dd, *J* = 4.0, 3.0 Hz, Ar-H), 7.435 (3H, m, Ar-H), 5.946 (1H, d, *J* = 16.5 Hz, H-10), 5.665 (1H, dd, *J* = 7.0 Hz, H-6), 5.599 (1H, dd, *J* = 16.5, 4.5 Hz, H-11), 5.209 (1H, m, H-12), 3.916 (1H, m, H-2), 3.520 (3H, s, MTPA-OCH_3), 3.254 (4H, overlap, H-15, 16), 2.550 (1H, m, H-3a), 2.237 (1H, brd, H-5a), 2.187 (1H, qd, *J* = 7.0, 3.5 Hz, H-1), 2.006 (1H, m, H-5b), 1.708 (1H, m, H-4), 1.601 (3H, s, H-9), 1.473 (1H, m, H-3b), 1.435 (9H, s, H-19, 20, 21), 1.251 (3H, d, *J* = 6.5 Hz, H-13), 1.039 (3H, d, *J* = 7.5 Hz, H-7).

The structure elucidation of key intermediates

Compound IV：

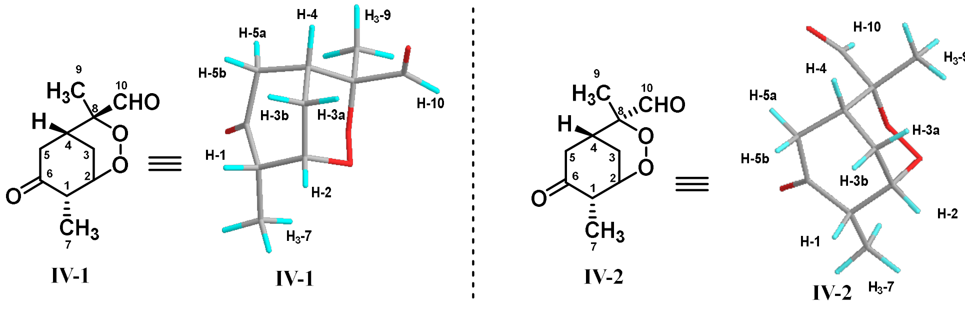

Compounds IV-1 and IV-2, a pair of stereoisomers, were obtained as a colorless oil by oxygen bridge ring formed. Their ^1H and ^{13}C NMR spectra showed two sets of similar resonance signals. They were established to have the same molecular formula of $C_{10}H_{14}O_4$, as determined by the HRESIMS data (m/z 199.0960 $[M + H]^+$, 221.0775 $[M + Na]^+$ for IV-1, and m/z 199.0959 $[M + H]^+$, 221.0777 $[M + Na]^+$ for IV-2, calcd 199.0960 for $C_{10}H_{15}O_4$, 221.0790 for $C_{10}H_{14}O_4Na$). The relative configuration of IV-1 was determined by a series of NOE experiments. H-3a (δ_H 2.32) was irradiated in the NOEs experiment, and the diagnostic NOE for H-10 (δ_H 9.80) was observed. Furthermore, irradiation of H-3b at δ_H 1.89 enhanced the signals for H-1 (δ_H 2.47) and H-5b (δ_H 2.51). On the other hand, the obvious NOE was observed for H-5a (δ_H 2.70) after irradiation of H_3-9 (δ_H1.07). These results revealed that the peroxide bridge and Me-7 oriented on the same side of the cyclohexanone ring, and the aldehyde group and H-2 was on the same side of the peroxide bridge-containing ring. In the NOEs experiment of **IV-2**, the obvious NOE observed for H_3-9 (δ_H1.55) after irradiation of H-2 (δ_H 4.43) suggested that the CH_3-9 and H-2 was on the same side of the peroxide bridge-containing ring. Due to intermidates IV-1 and IV-2 were derived from the common starting material II, the analysis of relative configurations of **IV-1** and **IV-2** indicated that they were a pair of epimer at C8. The absolute configuration of IV-1 was determined by X-ray diffraction experiment. A single crystal of IV-1-hemiacetal was obtained from MeOH. Thus, the crystal structure of IV-1-hemiacetal (Fig. S25a) allowed unambiguous assignment of the absolute configuration of IV-1 (1*S*, 2*S*, 4*R*, 8*S*) based on the Flack parameter (0.00(2)) for anomalous dispersion with Cu Kα radiation. Therefore, IV-2 was determined to be (1*S*, 2*S*, 4*R*, 8*R*).

Compound V：

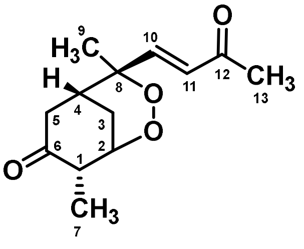

V-1

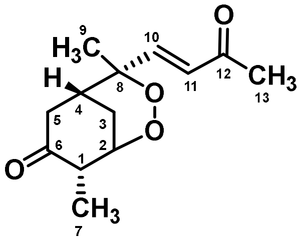

V-2

Compound V was obtained as a colorless oil by Wittig reactions of IV. They were established to have the same molecular formula of $C_{13}H_{19}O_4$, as determined by the HRESIMS data (*m/z* 239.1277 $[M + H]^+$, 261.1095 $[M + Na]^+$ for V-1, and m/z 239.1276 $[M + H]^+$,

261.1096 [M + Na]$^+$ for V-2, calcd 239.1283 for $C_{13}H_{19}O_4$, 261.1103 for $C_{13}H_{18}O_4Na$). The high similarity of the ^{1}H and ^{13}C NMR spectra of V-1 and V-2 suggested that they were a pair of stereoisomers. The geometry of the double bond in V-1 and V-2 were determined to be *E* by the coupling constants ($J_{\text{H-10, H-11}}$ = 16.5 Hz). Similar to IV-1 and IV-2, the relative configuration of V-1 and V-2 were also determined by NOE experiments. In the NOEs experiment of V-1, when H_3-9 was irradiated in the NOEs experiment, the diagnostic NOE for H-5a (δ_H 2.77) was observed. On the other hand, irradiation of H-3b at δ_H 1.87 enhanced the signals for H-1 (δ_H 2.45) and H-5b (δ_H 2.47, *J* = dd, 15.6, 4.2 Hz). These results revealed that the peroxide bridge and Me-7 oriented on the same side of the cyclohexanone ring and the α,β-unsaturated ketone side chain and H-2 were on the same side of the peroxide bridge-containing ring. In the NOEs experiment of V-2, the diagnostic NOE observed for H_3-9 after irradiation of H-2 (δ_H 4.39) suggested that the CH_3-9 and H-2 was on the same side of the peroxide bridge ring. The relative configuration of the peroxide bridge and Me-7 faced on the same side of the cyclohexanone ring was supported by the NOEs observed for H_3-9 after irradiation of H-3a (δ_H 2.68), for H-1 and H-5b after irradiation of H-3b (δ_H 1.90), and for olefin protons H-10 and H-11 after irradiation of H-5a (δ_H 2.54).

The individual absolute configurations of V-1 and V-2 were determined by comparison of the experimental and calculated ECD spectra using the TD-DFT method. The calculated ECD spectrum for V1a (1*S*, 2*S*, 4*R*, 8*S*) agreed well with that measured for V-1 (Fig. S25b), whereas the calculated ECD spectrum for V2a (1*S*, 2*S*, 4*R*, 8*R*) was consistent with that measured for V-2 (Fig. S25c). Thus, the absolute configurations of V-1 and V-2 were deduced to be (1*S*, 2*S*, 4*R*, 8*S*, 10*E*) and (1*S*, 2*S*, 4*R*, 8*R*, 10*E*), respectively.

Compound VI：

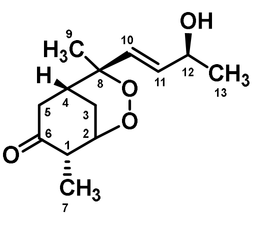

VI-1a

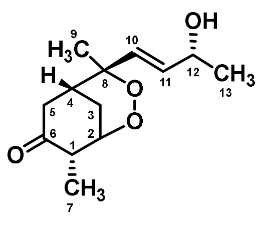

VI-1b

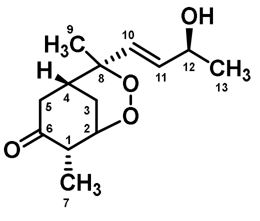

VI-2a

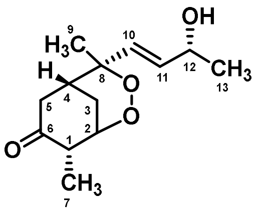

VI-2b

Compound VI-1a was obtained as a colorless oil by the reduction of carbonyl group in C-12 of V-1. The C-12 absolute configuration of VI-1a was unambiguously determined by the modified Mosher's method. The treatment of VI-1a with (*R*)- and (*S*)-MTPA-Cl afforded the (*S*)- and (*R*)-MTPA esters (VI-1aS and VI-1aR), respectively. The difference in chemical shift values ($\Delta\delta^{SR} = \delta_S - \delta_R$) for the diastereomeric esters VI-1aS and VI-1aR was calculated to

assign the absolute configuration of C-12 as *S* (Fig. S25d). Therefore, the absolute configuration of VI-1a was deduced to be (1*S*, 2*S*, 4*R*, 8*S*, 10*E*, 12*S*). As VI-1b was the epimer of VI-1a, the absolute configuration of VI-1b was then assigned as (1*S*, 2*S*, 4*R*, 8*S*, 10*E*, 12*R*).

Compounds VI-2a and VI-2b were obtained as a colorless oil by the reduction of carbonyl group in C-12 of V-2. VI-2a and VI-2b was a pair of epimer at C12. The C-12 absolute configuration of VI-2a was unambiguously determined using the above-mentioned modified Mosher's method. The treatment of VI-2a with (*R*)- and (*S*)-MTPA-Cl afforded the (*S*)- and (*R*)-MTPA esters (VI-2aS and VI-2aR), respectively. The calculation of the $\Delta\delta^{SR}$ (δ_S - δ_R) values for VI-2aS and VI-2aR assigned the absolute configuration of C-12 as *S* (Fig. S25e). Therefore, the absolute configuration of VI-2a was assigned as (1*S*, 2*S*, 4*R*, 8*R*, 10*E*, 12*S*). As VI-2b was the epimer of VI-2a, the absolute configuration of VI-2b was then assigned as (1*S*, 2*S*, 4*R*, 8*R*, 10*E*, 12*R*).

Compounds VIII-1a, VIII-2a, VIII-2b:

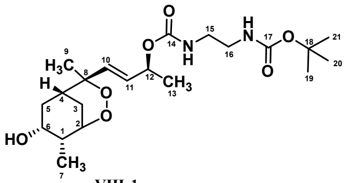
VIII-1a

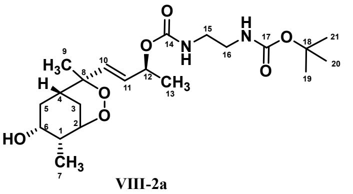
VIII-2a

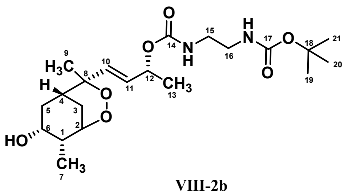
VIII-2b

Compounds VIII-1a, VIII-2a, VIII-2b were obtained as the primary product by the reduction of carbonyl group in C-6 of VII-1a, VII-2a, VII-2b, respectively. Their ^{1}H and ^{13}C NMR spectra showed three sets of similar resonance signals. Briefly, they all showed small coupling constants for H-6 in their ^{1}H NMR spectra [VIII-1a δ_H 3.83 (dd, *J* = 4.5, 4.5 Hz), VIII-2a δ_H 3.81 (dd, *J* =6.0, 5.5 Hz), and VIII-2b δ_H 3.74 (dd, *J* = 5.5, 5.0 Hz)]. These results indicated that H-6 was β-configuration (equatorial orientation) and 6-hydroxyl was α-configuration (axial orientation) in their six-member carbon ring in a chair conformation. Thus, the absolute configuration of C6 was assigned as *R* for VIII-1a, VIII-2a and VIII-2b. Furthermore, the C-6 absolute configuration of VIII-2a and VIII-2b were unambiguously determined using the above-mentioned modified Mosher's method. The calculation of the $\Delta\delta^{SR}$ (δ_S - δ_R) values for VIII-2aS, VIII-2aR and VIII-2bS, VIII-2bR established 6*R* absolute configuration for VIII-2a and VIII-2b (Fig. S25f, 25g). Therefore, the absolute configuration of VIII-1a, VIII-2a, VIII-2b were assigned as (1*S*, 2*S*, 4*R*, 6*R*, 8*S*, 10*E*, 12*S*), (1*S*, 2*S*, 4*R*, 6*R*, 8*R*, 10*E*, 12*S*), (1*S*, 2*S*, 4*R*, 6*R*, 8*R*, 10*E*, 12*R*), respectively.

Supplementary references

1. Wei, C. et al. Activity-based protein profiling reveals that secondary-carbon-centered radicals of synthetic 1,2,4-trioxolanes are predominately responsible for modification of protein targets in malaria parasites. *Chem. Commun.* **55**, 9535-9538 (2019).
2. Lambros, C. & Vanderberg, J. P. Synchronization of *Plasmodium falciparum* erythrocytic stages in culture. *J Parasitol.* **65**, 418-420 (1979).
3. Birnbaum, J. et al. A Kelch13-defined endocytosis pathway mediates artemisinin resistance in malaria parasites. *Science* **367**, 51–59 (2020).
4. Amaratunga, C., Neal, A. T., & Fairhurst, R. M. Flow cytometry-based analysis of artemisinin-resistant *Plasmodium falciparum* in the ring-stage survival assay. *Antimicrob. Agents Chemother.* **58**, 4938-4940 (2014).
5. Klonis, N., et al. Artemisinin activity against *Plasmodium falciparum* requires hemoglobin uptake and digestion. *Proc. Natl. Acad. Sci. U.S.A.* **108**, 11405–11410 (2011).
6. O'Neil P. M. et al. A carbonyl oxide route to antimalarial Yingzhaosu A analogues: synthesis and antimalarial activity. *Tetrahedron Lett.* **1998**, *39*, 6065-6068.
7. Murithi J. M. et al. Combining stage specificity and metabolomics profiling to advance antimalarial drug discovery. *Cell Chem. Bio*. **2020**, *27*, 158-171.

Supplementary figures

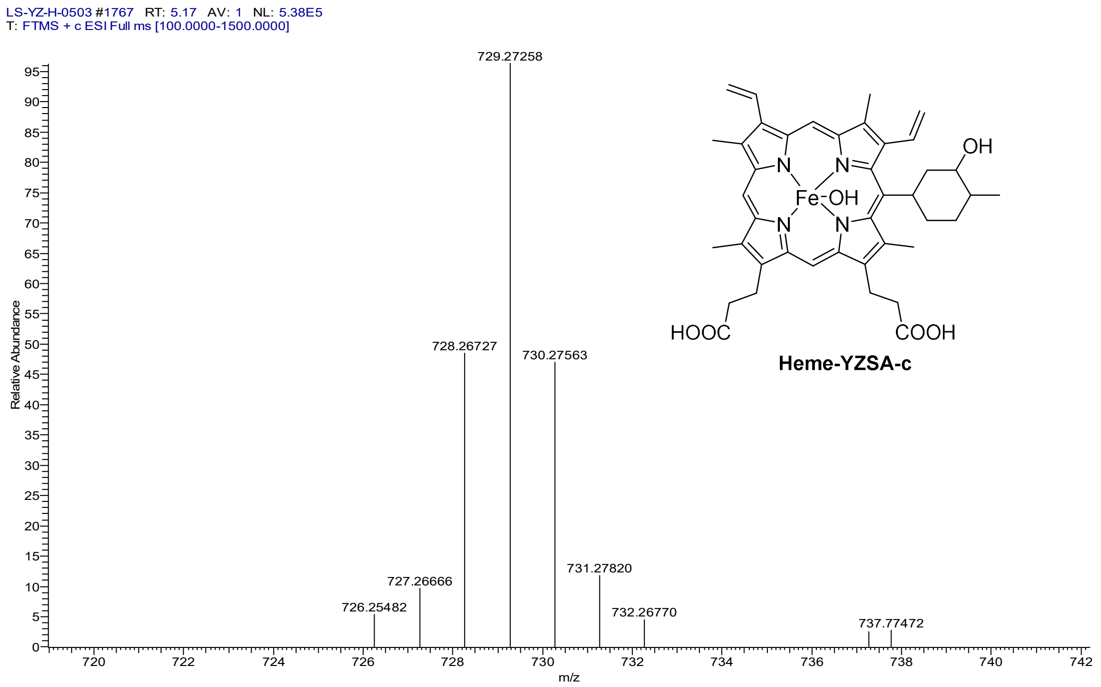

Fig. S1 HRMS data of mono-alkylated heme (Heme-YZSA-c) in the reaction between Yingzhaosu A and heme

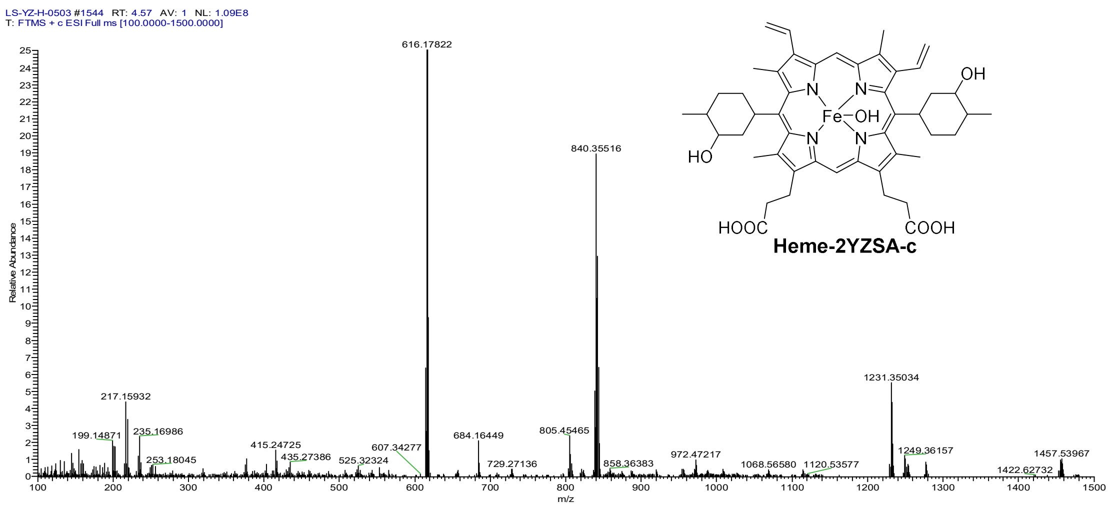

Fig. S2 HRMS data of dual-alkylated heme (Heme-2YZSA-c) in the reaction between Yingzhaosu A and heme

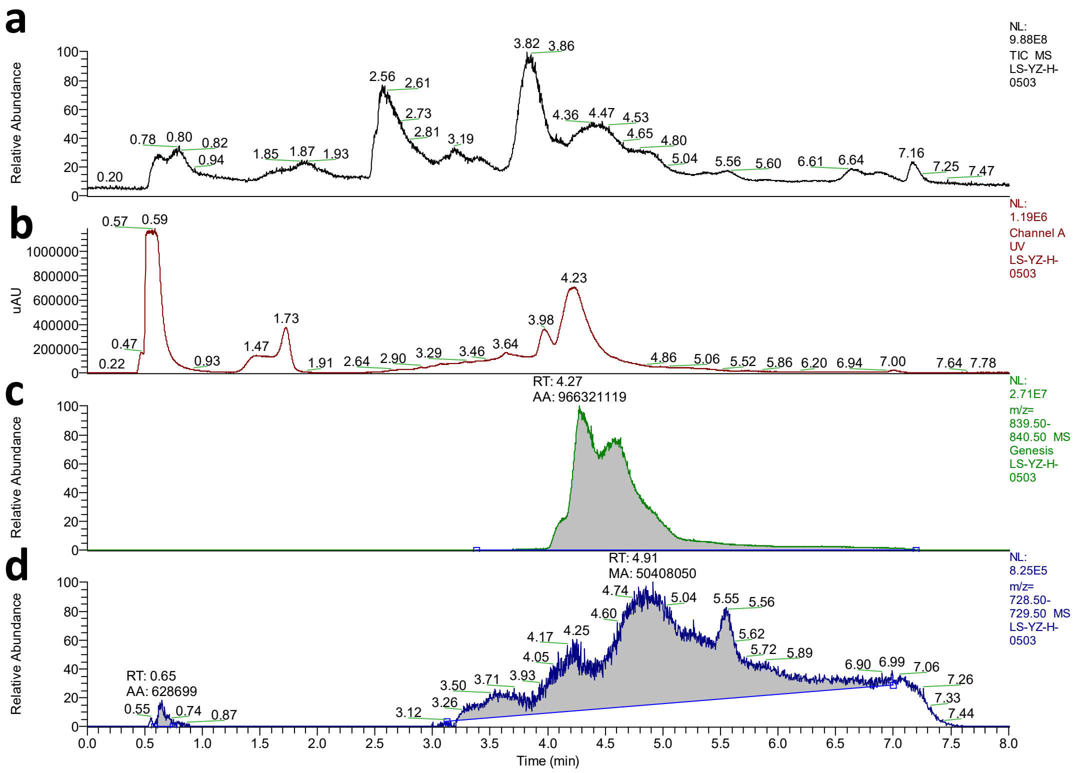

Fig. S3 HPLC/UV/HRMS analyses of dual-alkylated heme and mono-alkylated heme in the reaction between Yingzhaosu A and heme. **a**, Total ion current (TIC) profile. **b**, HPLC/UV chromatogram monitored at 254 nm. **c**, Extracted ion current (EIC) with the m/z value being 839.50-840.50 for dual-alkylated heme. **d,** Extracted ion current (EIC) with the m/z value being 728.50-729.50 for mono-alkylated heme. The ratio of dual-alkylated heme to mono-alkylated heme was calculated with their responding areas of ion current (966321119/50408050 = 19/1)

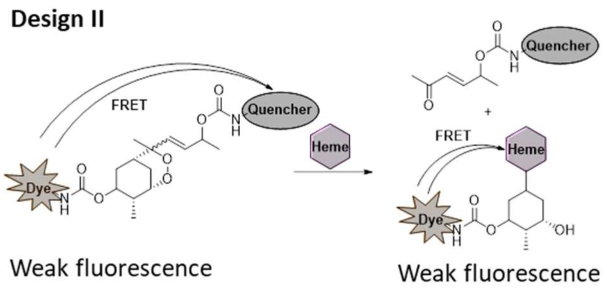

Fig. S4 Design II for FRET-based heme-reactive probe. In principle, this design is worse than design I (Fig. 1b in maintext) as the released dye was not highly fluorescent because of the quenching effect of heme

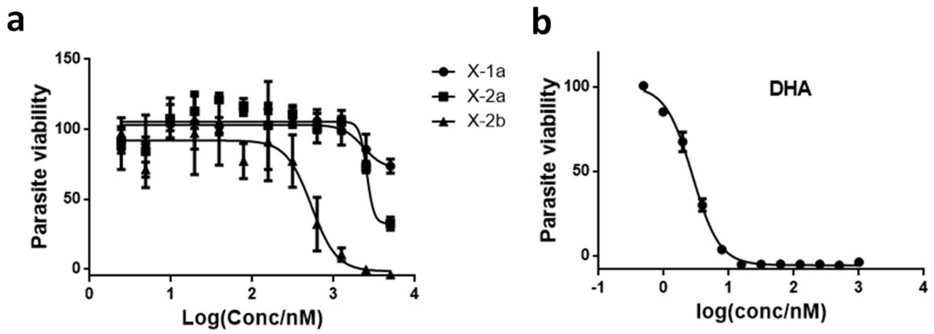

Fig. S5 The viability of *P. falciparum* treated with probes and DHA. a The viability of P. falciparum (3D7) treated with X-1a, X-2a or X-2b. **b** The viability of *P. falciparum* (3D7) treated with DHA. Results are shown as the means ± SD with n = 3 biological replicates

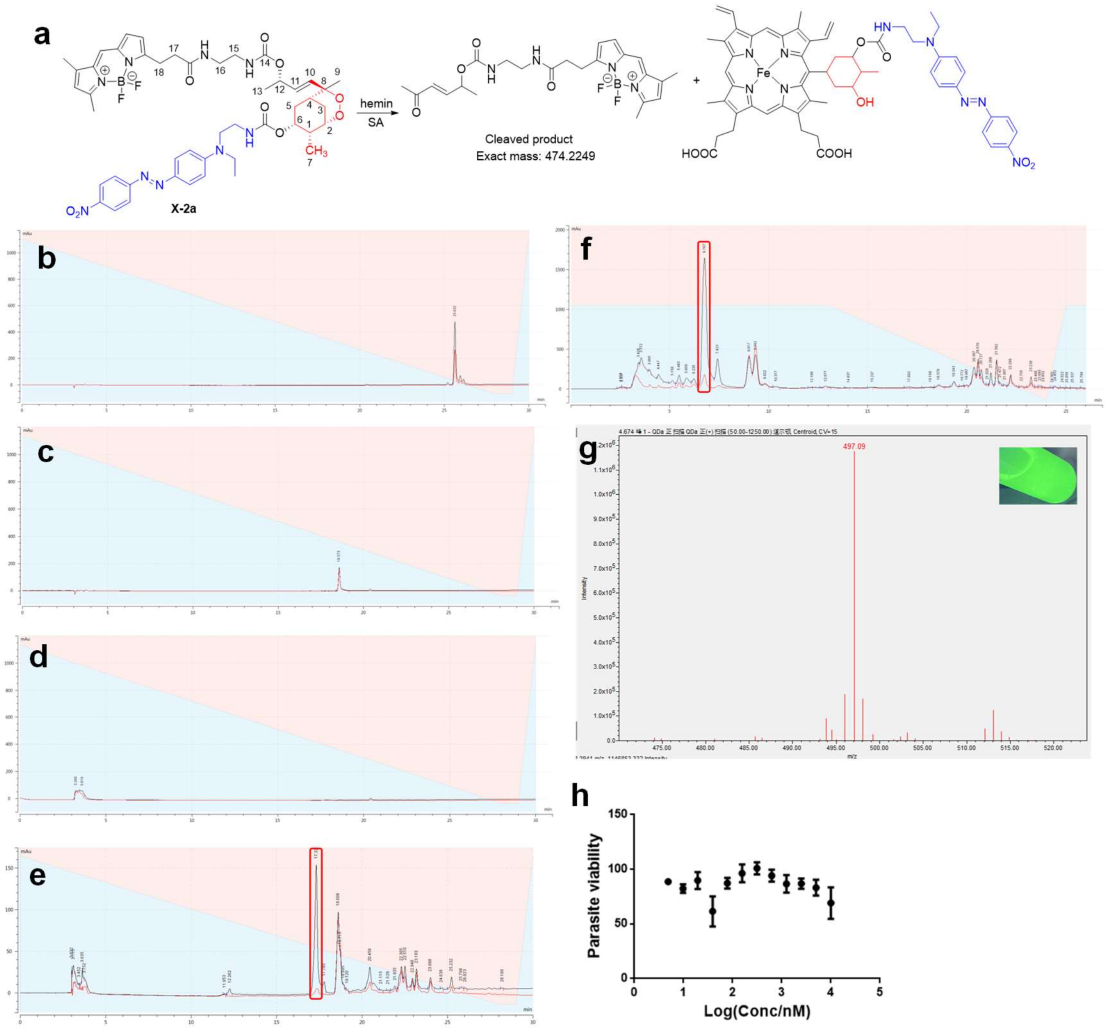

Fig. S6 Identification and isolation of cleavage products generated in the reaction between X-2a and hemin. **a** The reaction scheme between probe X-2a and heme. HPLC analysis: Compounds were eluted using a binary solvent system consisting of acetonitrile/0.1% TFA (solvent A) and H_2O/0.1% TFA (solvent B), flow rate: 3 mL/min, λ = 480/530 nm. (**a-d**), Identification: The gradient used was 95% to 5% of solvent A over 28 min, then the isocratic elution was 5% of solvent A over 1 min. **b** X-2a, t_R = 25.632 min. **c** hemin, t_R = 18.573 min. **d** L-SA, t_R = 3.618 min. **e** reaction solution (30 min), the free fluorophore (red box): t_R = 17.320 min. **f** Isolation: The isocratic elution was 55% of solvent A over 13 min, then the gradient used was 55% to 2% of solvent A over 11 min, the free fluorophore (red box): t_R = 6.767 min. **g** MS data of the free fluorophore generated in the reaction between X-2a and hemin. The inset picture was obtained by irradiation of the solution of cleavage product at 365 nm. **h** The viability of plasmodium falciparum treated with cleaved free fluorophore. Results are shown as the means ± SD with n = 3 biological replicates

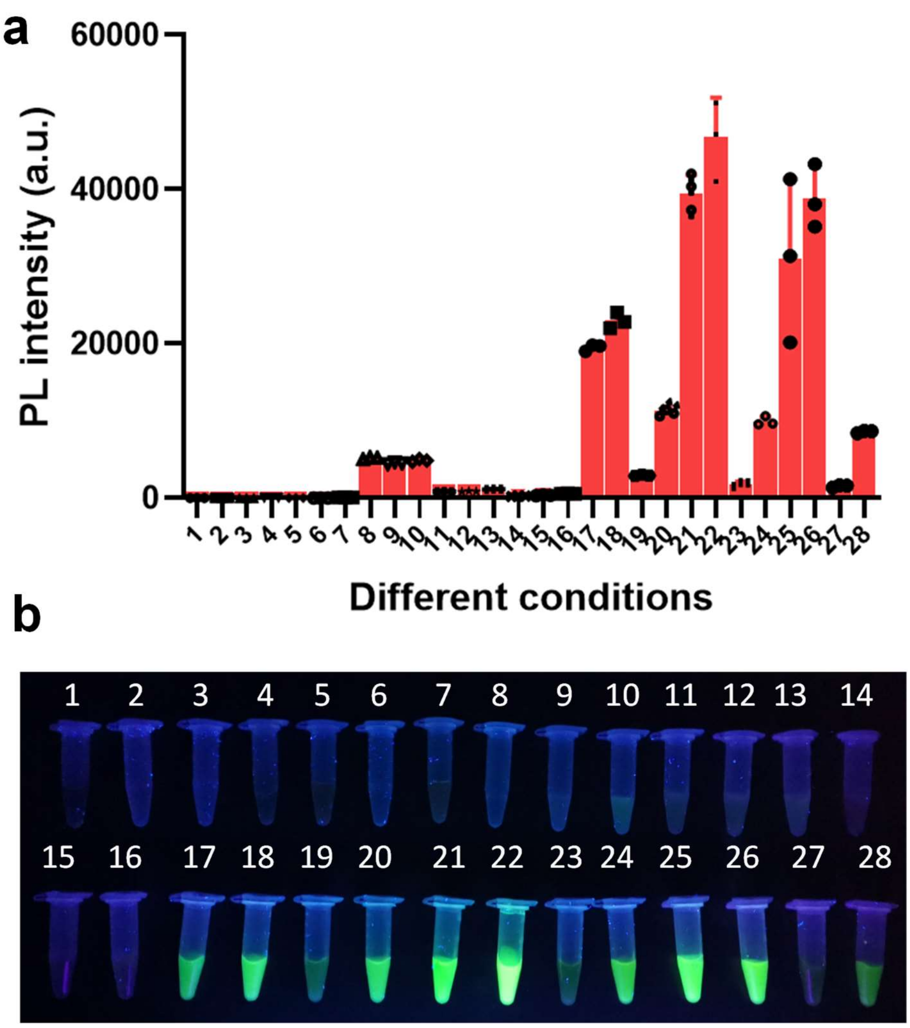

Fig. S7 PL intensity of three probes incubated under different conditions for 2 h. a PL intensity of three probes incubated with/without heme under different conditions for 2 h. Results are shown as the means ± SD with n=3 biological replicates. **b** A photograph was taken with 365 nm excitation. The different conditions were as follows: 1, hemin; 2, SA; 3, GSH; 4, hemin+SA; 5, hemin+GSH; 6, SA+GSH; 7, hemin+SA+GSH; 8, X-1a; 9, X-1a+GSH; 10, X-1a+SA; 11, X-2a; 12, X-2a+GSH; 13, X-2a+SA; 14, X-2b; 15, X-2b+GSH; 16, X-2b+SA; 17, hemin+SA+X-1a; 18, hemin+SA+GSH+X-1a; 19, hemin+X-1a; 20, hemin+GSH+X-1a; 21, hemin+SA+X-2a; 22, hemin+SA+GSH+X-2a; 23, hemin+X-2a; 24, hemin+GSH+X-2a; 25, hemin+SA+X-2b; 26, hemin+SA+GSH+X-2b; 27, hemin+X-2b; and 28, hemin+GSH+X-2b. The results of condtions 8-28 were also shown in the maintext as Fig 1e

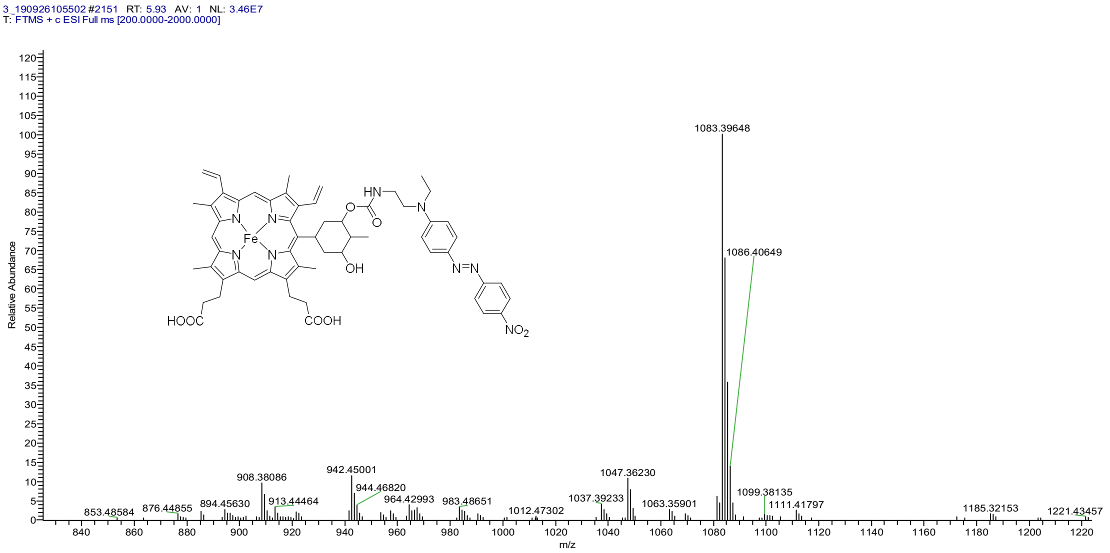

Fig. S8 HRMS data of the alkylated heme generated in the reaction between X-1a and heme

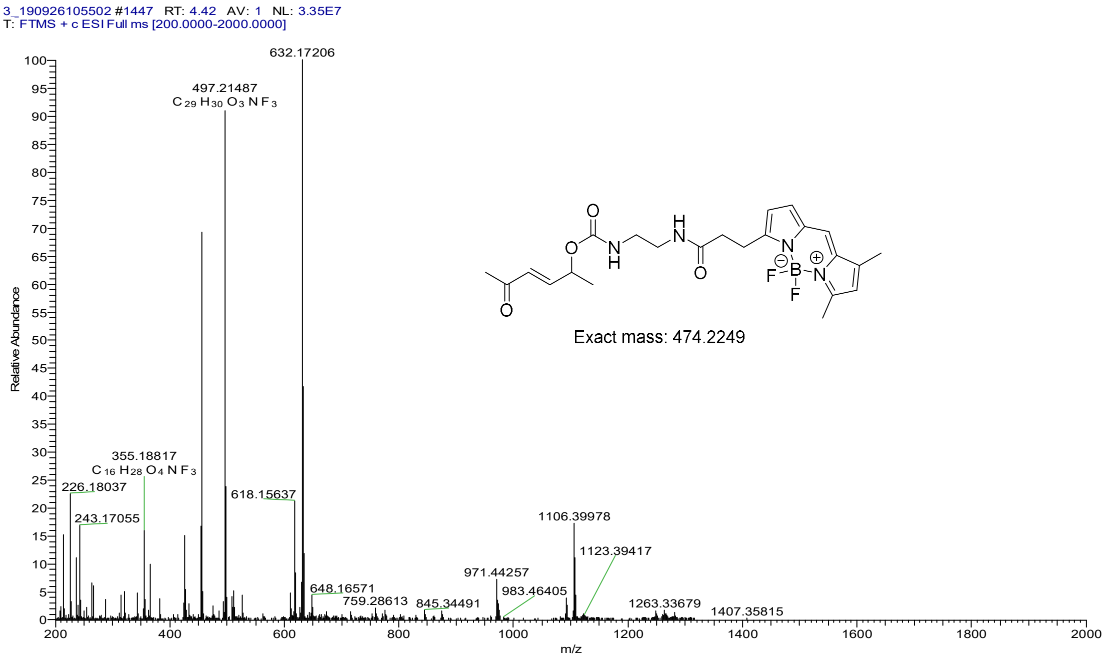

Fig. S9 HRMS data of the free fluorophore generated in the reaction between X-1a and heme

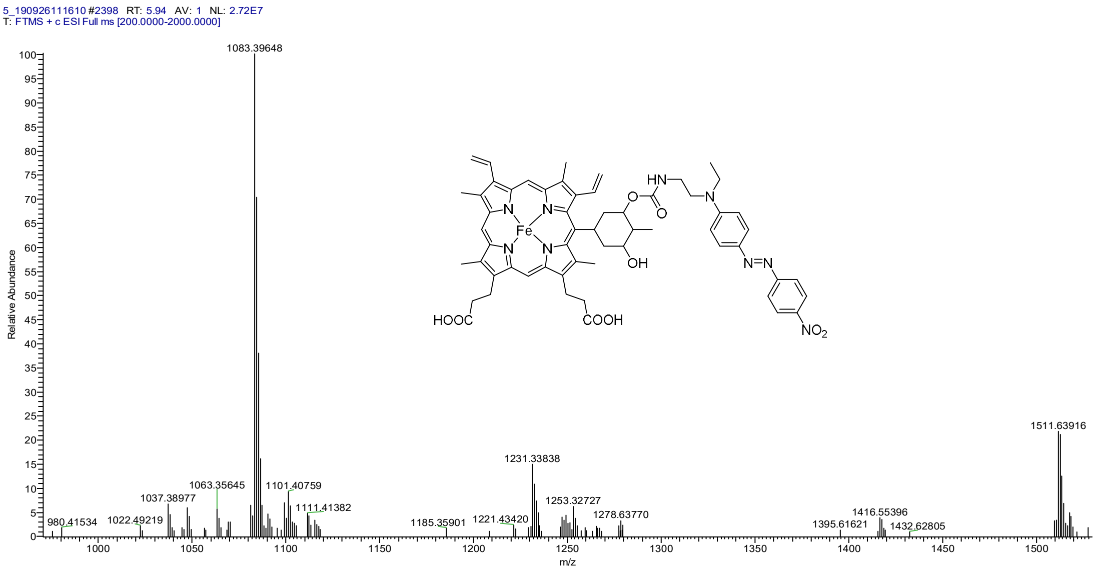

Fig. S10 HRMS data of the alkylated heme generated in the reaction between X-2a and heme

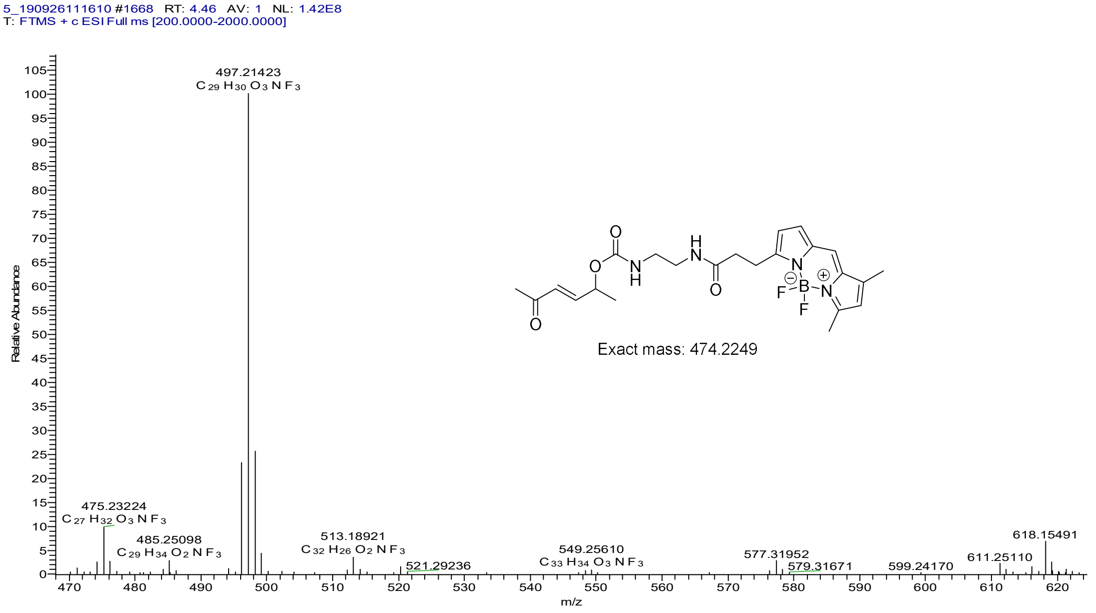

Fig. S11 HRMS data of the free fluorophore generated in the reaction between X-2a and heme

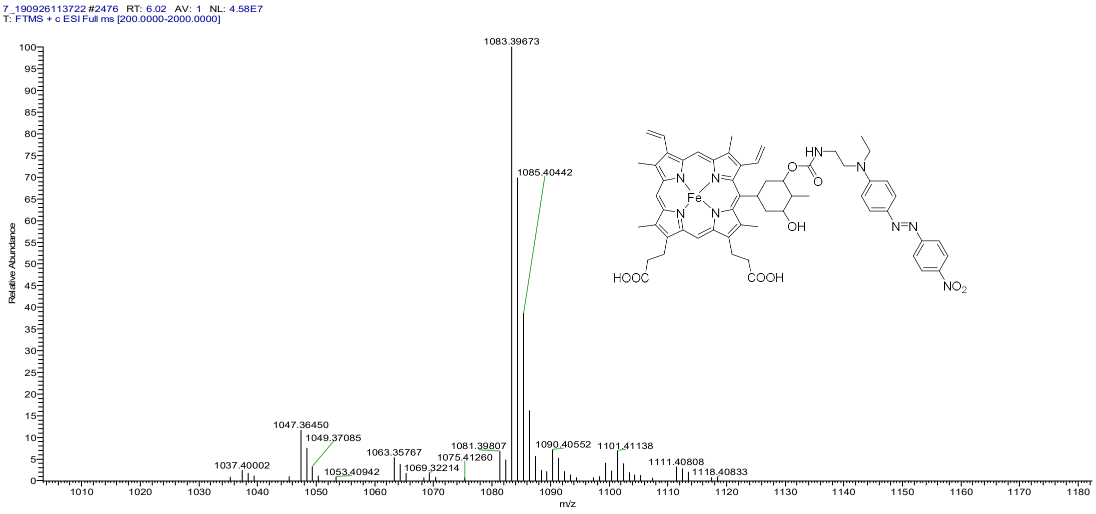

Fig. S12 HRMS data of the alkylated heme generated in the reaction between X-2b and heme

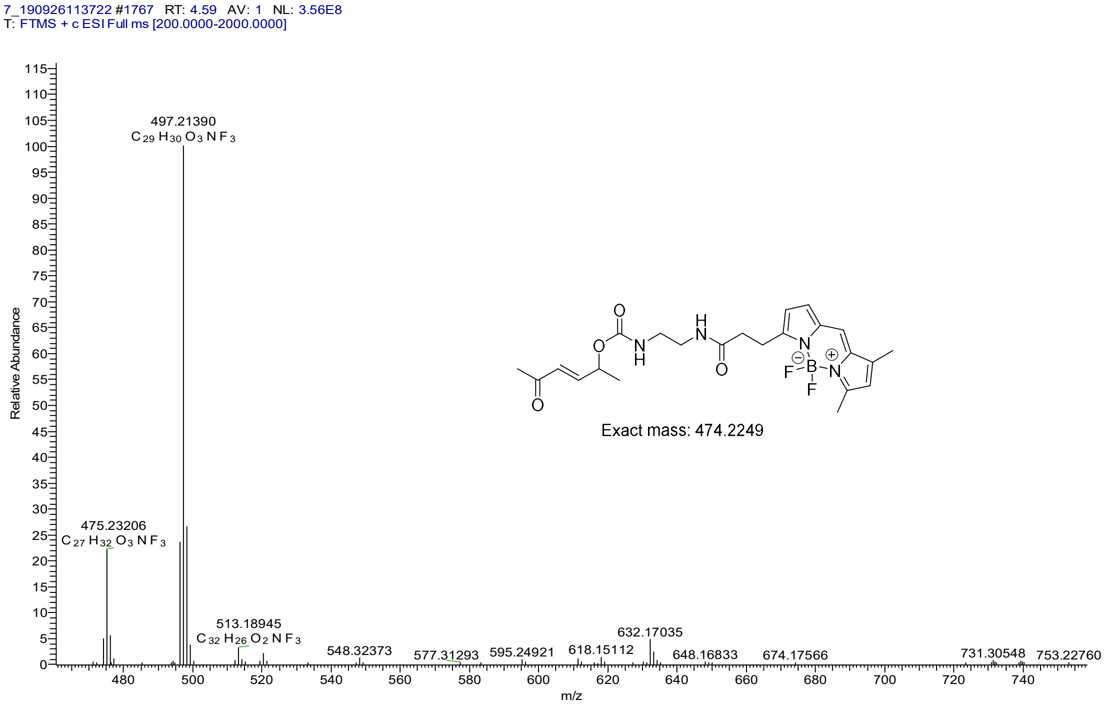

Fig. S13 HRMS data of the free fluorophore generated in the reaction between X-2b and heme

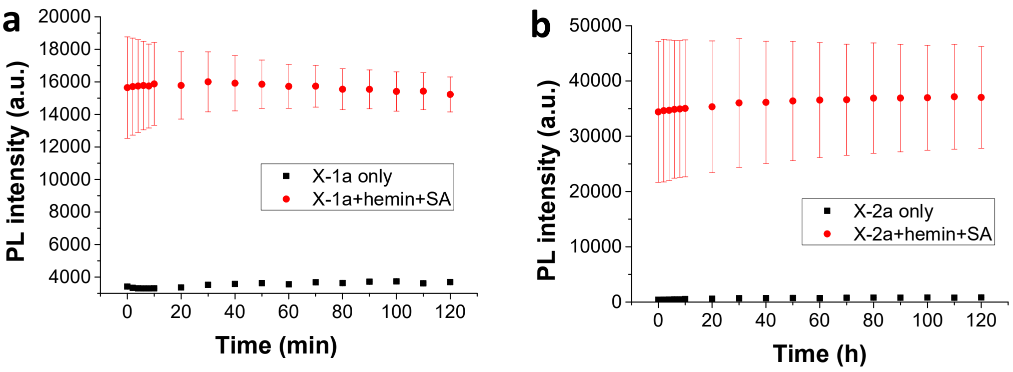

Fig. S14 Time-dependent PL intensity of probes treated with hemin and sodium ascorbate (SA). **a** X-1a. **b** X-2a. The final concentration of the probe, hemin and SA were 50 μM, 50 μM, 500 μM. Results are shown as the means ± SD with n = 3 biological replicates

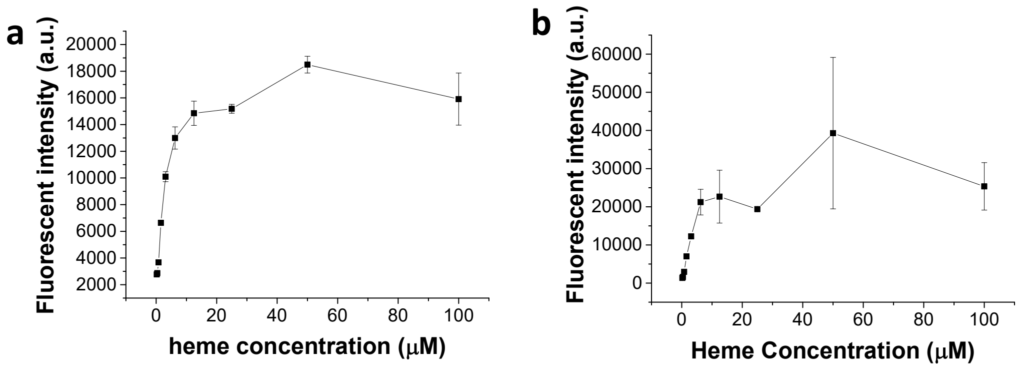

Fig. S15 PL intensity of probes treated with different concentrations of hemin in presence of SA. **a** X-1a. **b** X-2a. Results are shown as the means ± SD with n = 3 biological replicates

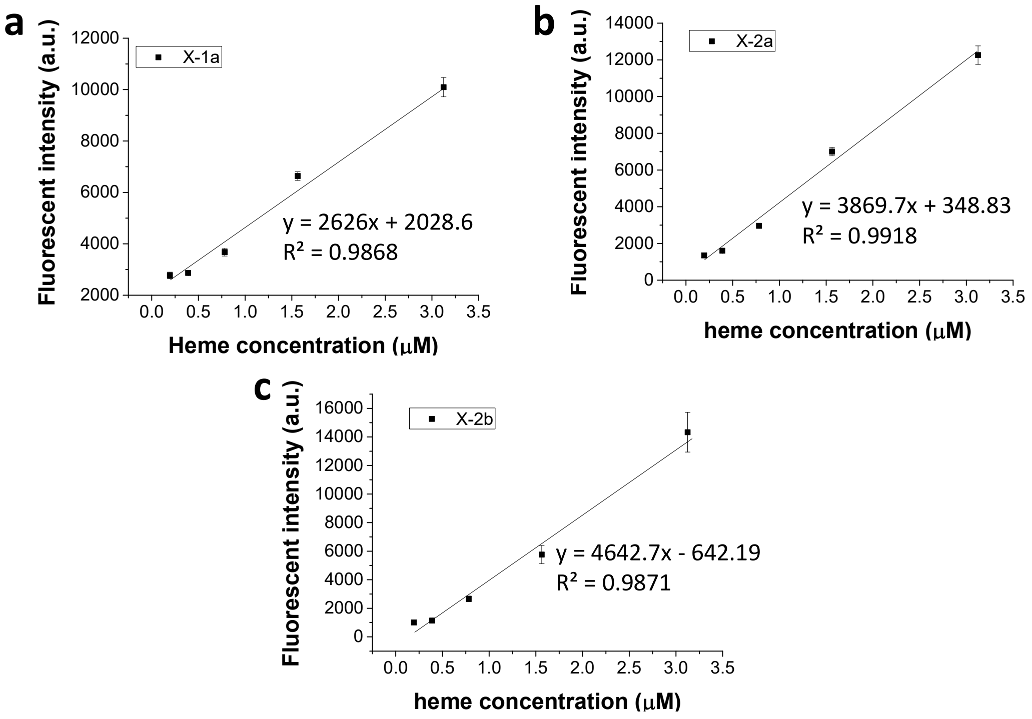

Fig. S16 Linear relationship between PL intensity of probes and low heme concentration. PL intensity of probe X-2a (**a**) or X-2a (**b**) or X-2b (**c**) treated with low concentrations of hemin in presence of sodium ascorbate. The inset equations and value of R^2 represented a good linear relationship between the PL intensity of the probe and heme concentration

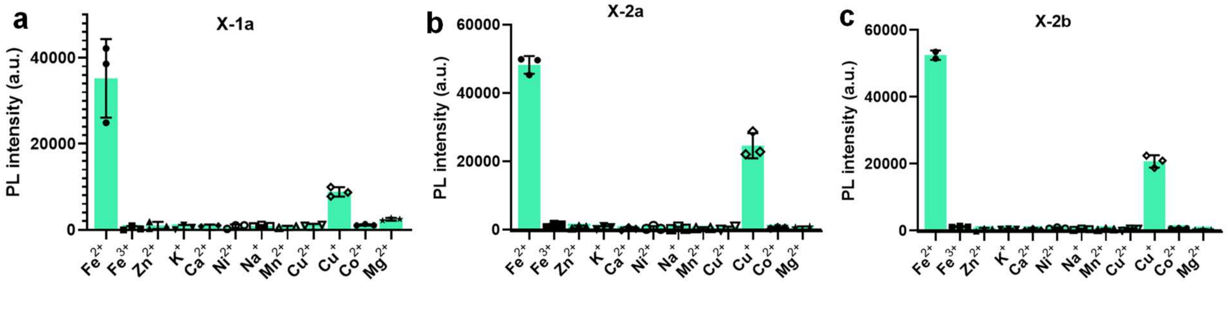

Fig. S17 The PL intensity of probes (0.5 μM) treated with different transition metal ion (50 μM) for 0.5 h. **a** X-1a; **b** X-2a; **c** X-2b. Results are shown as the means ± SD with n = 3 biological replicates

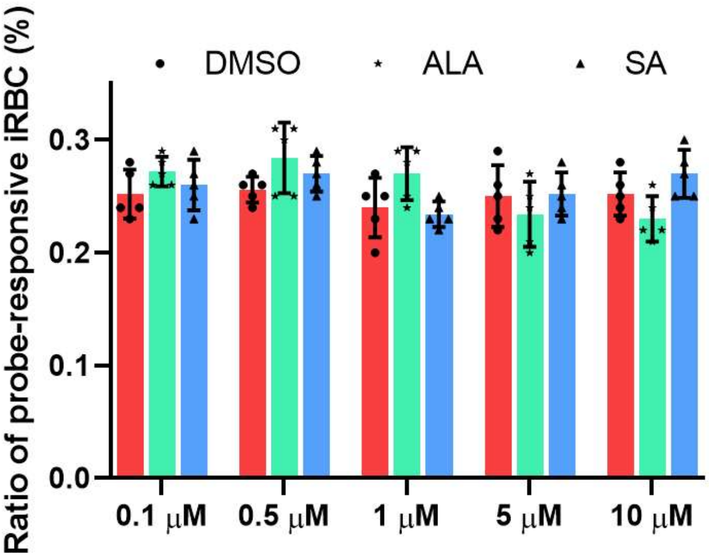

Fig. S18 The heme for the activation of the probe X-2b was mostly from hemoglobin digestion. The ratio of probe-responsive iRBCs treated with ALA or SA for 1 h and subsequently co-incubated with X-2b for 3 h. The ratio of probe-responsive iRBCs denotes the number of probe-imaged RBCs among the examined 100,000 RBCs. Ring-stage parasites were obtained by treating un-synchronized parasites with sorbitol once. ALA, the precursor of heme biosynthesis; SA, the inhibitor of heme biosynthesis. Results are shown as the means ± SD with n = 5 biological replicates

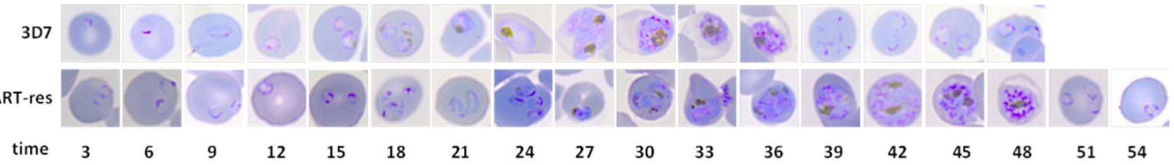

Fig. S19 Time dependent morphorlogy of 3D7 and ART-resistant *P. falciparum* during one life cycle. Syncronized parasites were stained every 3 hour. ART-res, ART-resistant *P. falciparum* (6320)

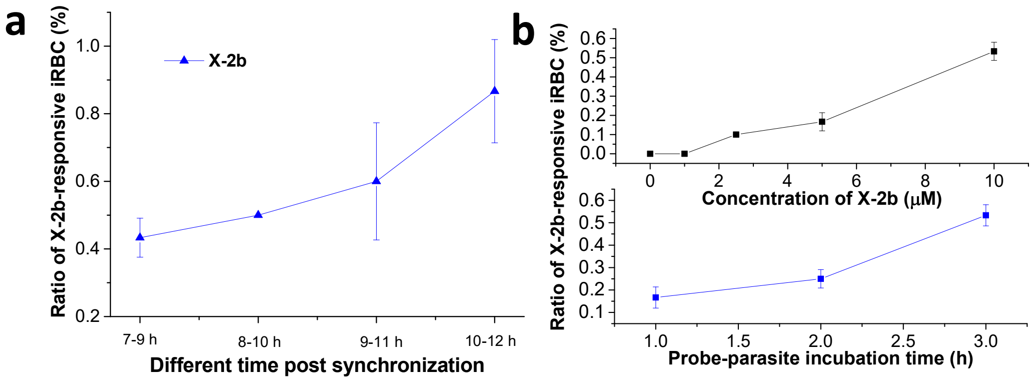

Fig. S20 Optimization of screening conditions for the high-throughput screening. **a** Optimization of screening conditions in terms of synchronized parasites with different ages. **b** The ratio of probe-responsive iRBCs was highly dependent on the concentration of probe X-2b and the time of incubation between probe X-2b and parasites. Results are shown as the means ± SD with n = 3 biological replicates

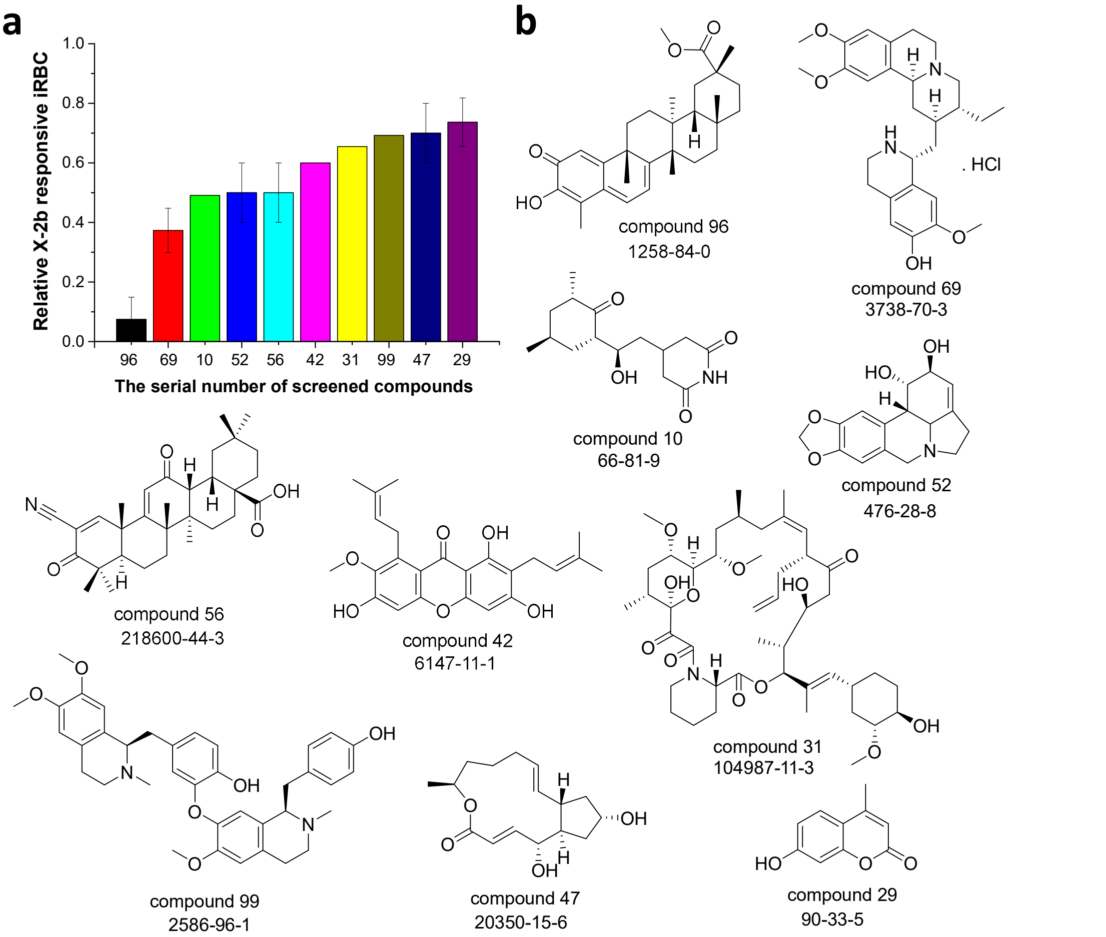

Fig. S21 The top 10 hits which decreased the heme level in the ring stage. **a** The ratio of probe-responsive iRBCs in the presence of the top 10 hits. Results are shown as the means ± SD with n = 2 biological replicates. **b** The chemical structures of top 10 hits

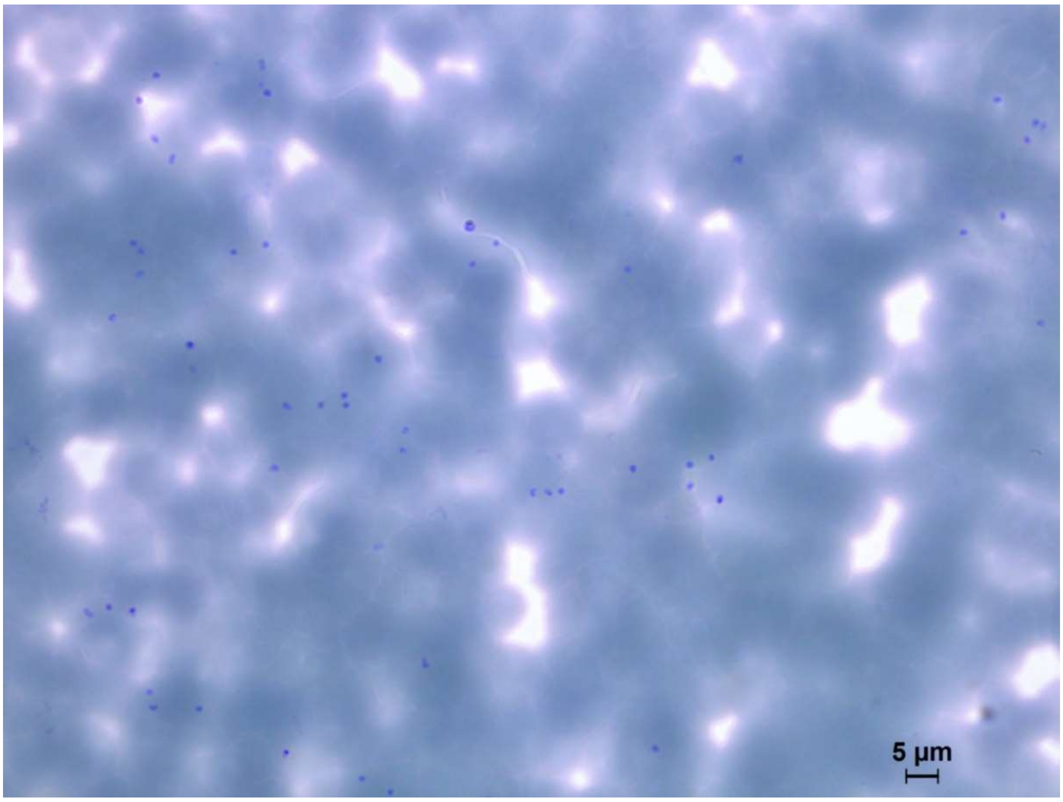

Fig. S22 The morphology of parasites treated with compound 96 for 12 h after synchronization

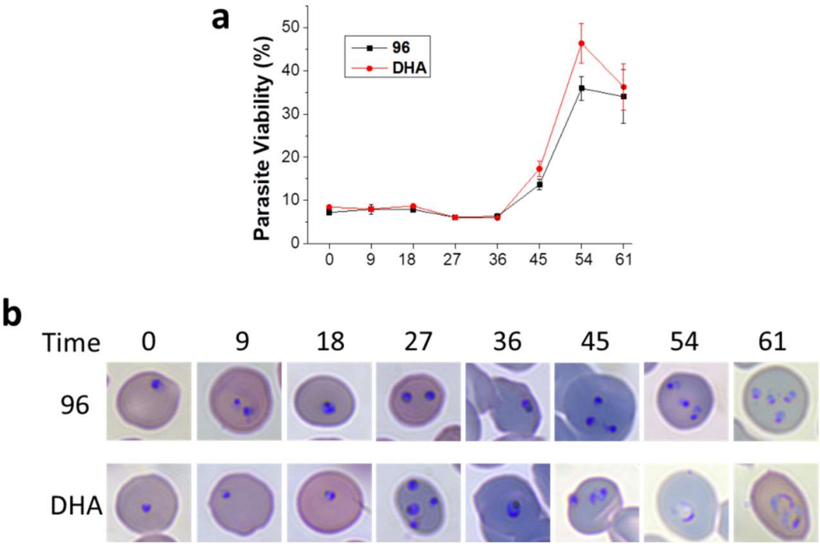

Fig. S23 The viability and morphology of ART-resistant *P. falciparum* treated with 96 or DHA in a 6 h incubation manne. a Viability of ART-resistant *P. falciparum* at different stages treated with compound 96 or DHA in a 6 h incubation manner. Results are shown as the means ± SD with n = 5 biological replicates. **b** The morphology of ART-resistant *P. falciparum* treated with compound 96 or DHA in a 6 h incubation manner

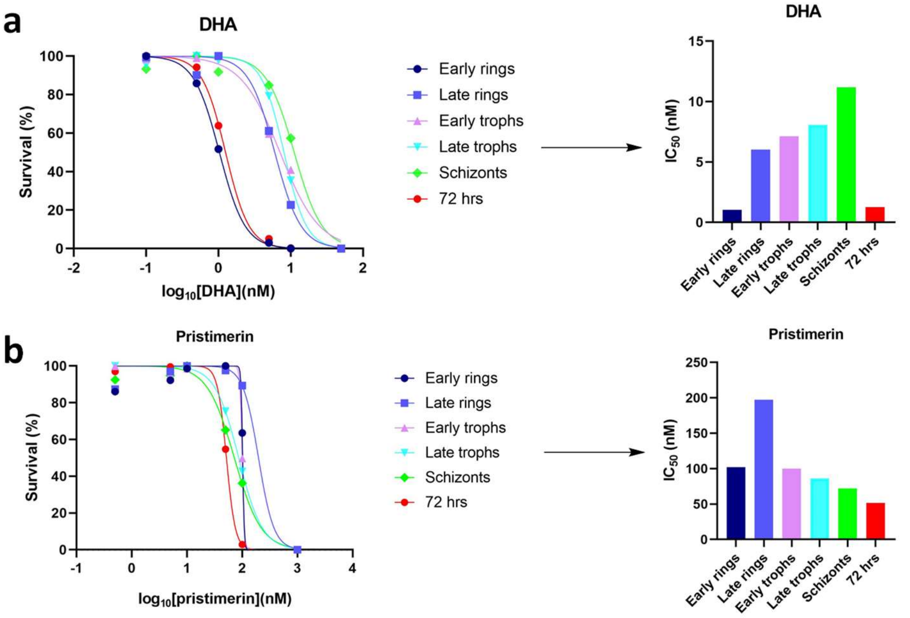

Fig. S24 Detailed asexual blood stage susceptibility profiles for DHA and pristimerin towards *P. falciparum* (3D7)

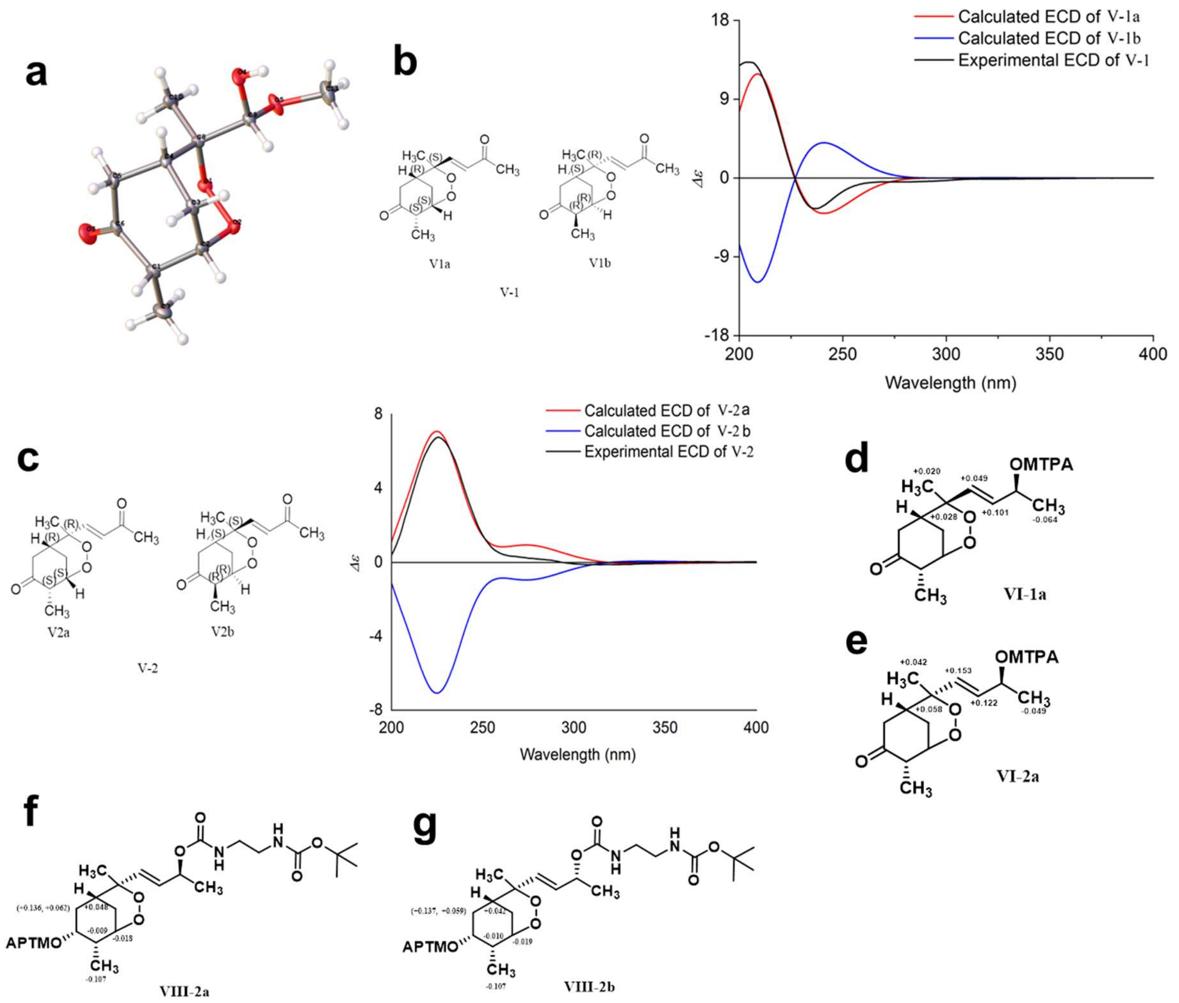

Fig. S25 The structure elucidation of key intermediates. a The crystal structure of IV-1-hemiacetal whose crystal was cultured in methanol. X-ray crystallographic data has been deposited in the Cambridge Crystallographic Data Centre database (http://www.ccdc.cam.ac.uk/) under accession code CCDC 2032747. **b** The measured ECD and theoretical ECD spectra of V-1. **c** The measured ECD and theoretical ECD spectra of V-1. **d** △δ_H of (*S*)-MTPA and (*R*)-MTPA by intermediates VI-1a. **e** △δ_H of (*S*)-MTPA and (*R*)-MTPA by intermediates VI-2a. **f** △δ_H of (*S*)-MTPA and (*R*)-MTPA by intermediate VIII-2a. **g** △δ_H of (*S*)-MTPA and (*R*)-MTPA by intermediate VIII-2b

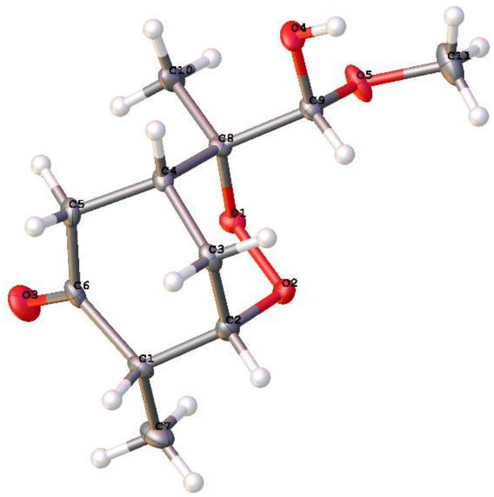

Fig. S26 Enlarged single crystal X-ray structure of IV-1 hemiacetal

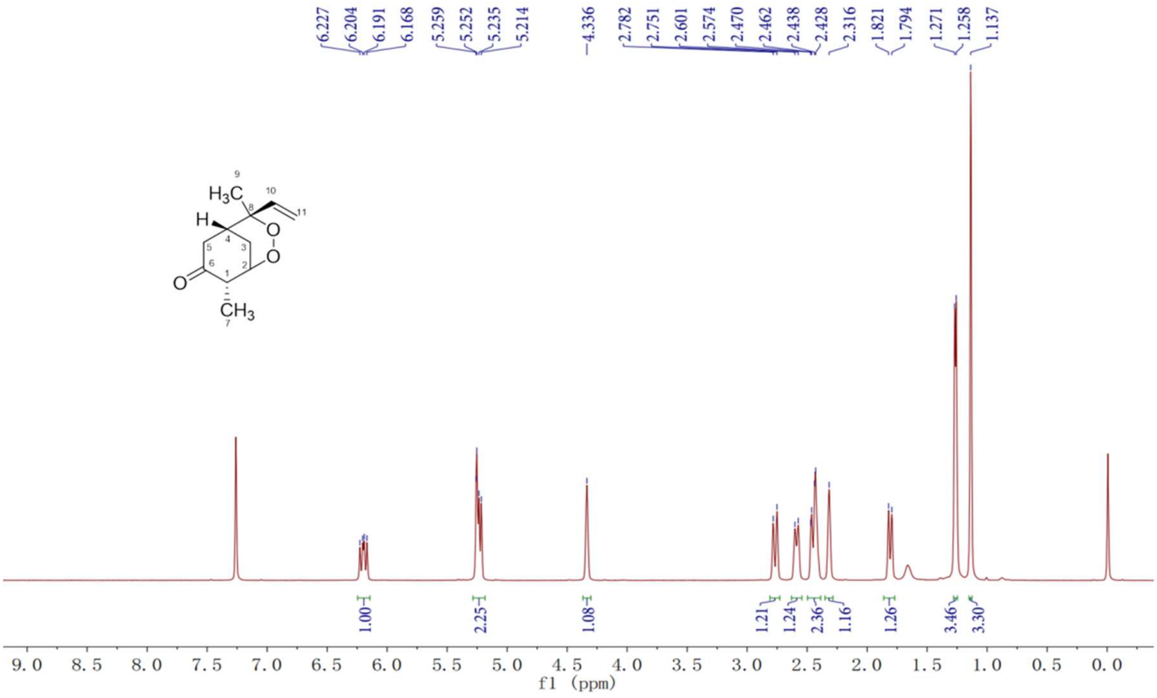

Fig. S27 ^{1}H NMR spectrum of compound III-1 in $CDCl_3$ (500 MHz)

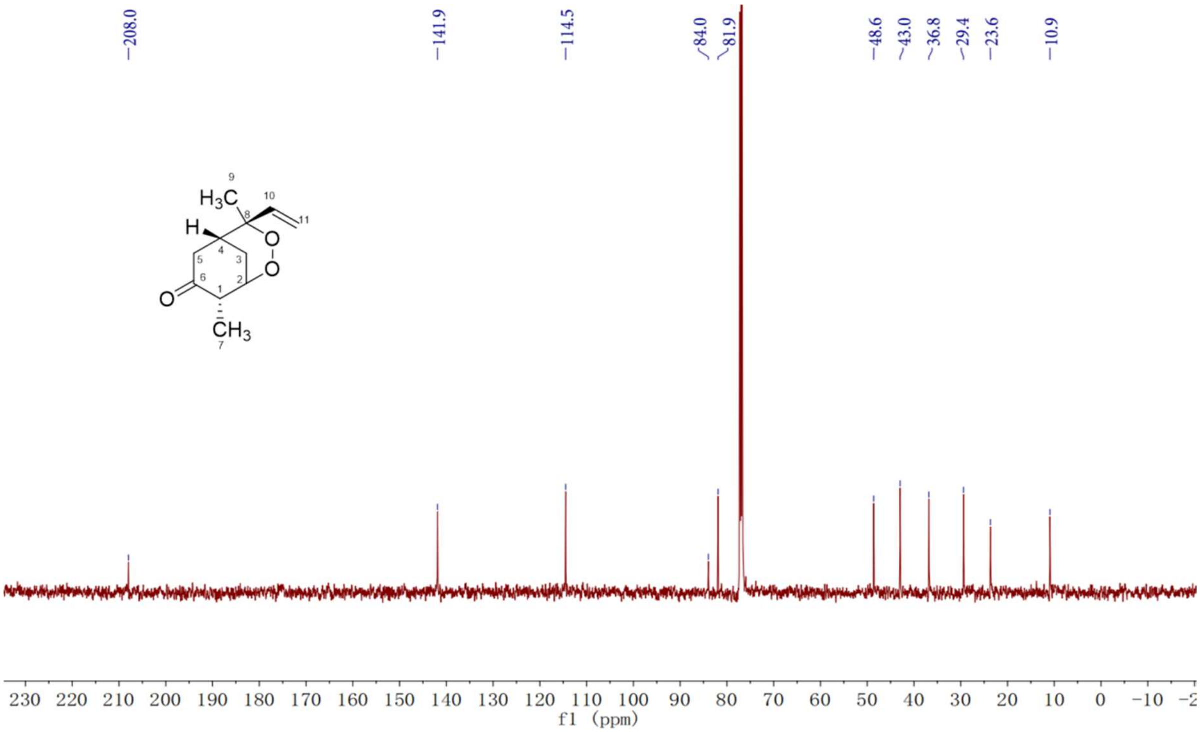

Fig. S28 ^{13}C NMR spectrum of compound III-1 in $CDCl_3$ (125 MHz)

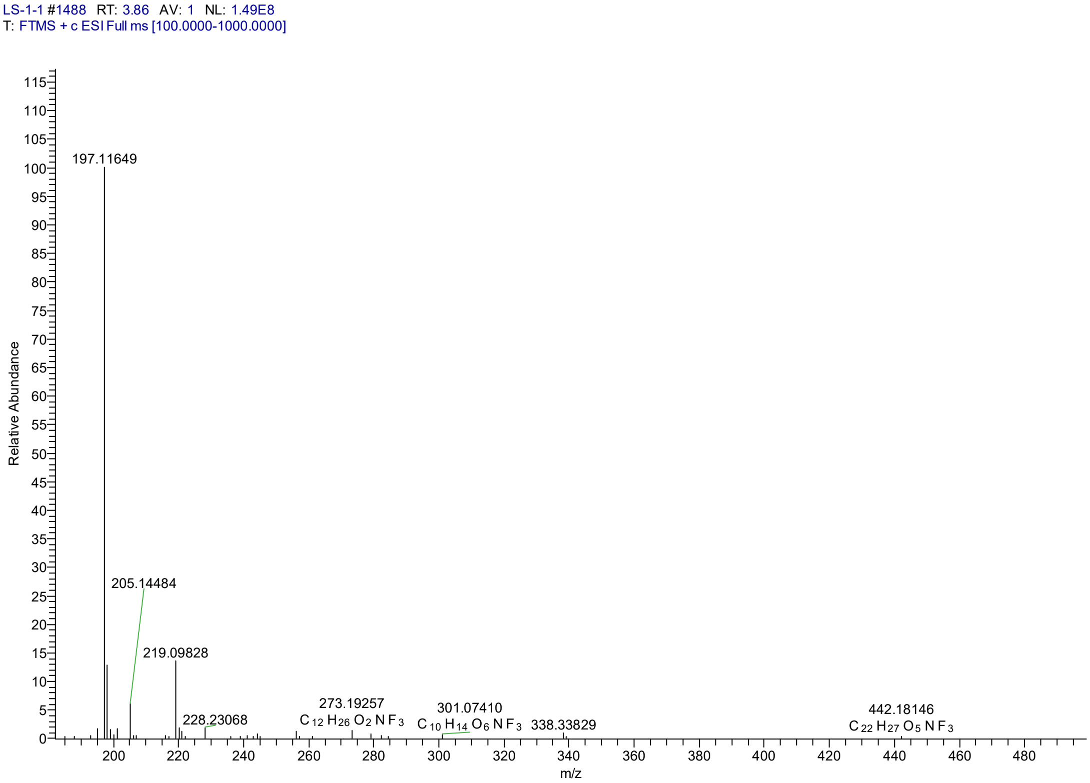

Fig. S29 HRMS data of compound III-1

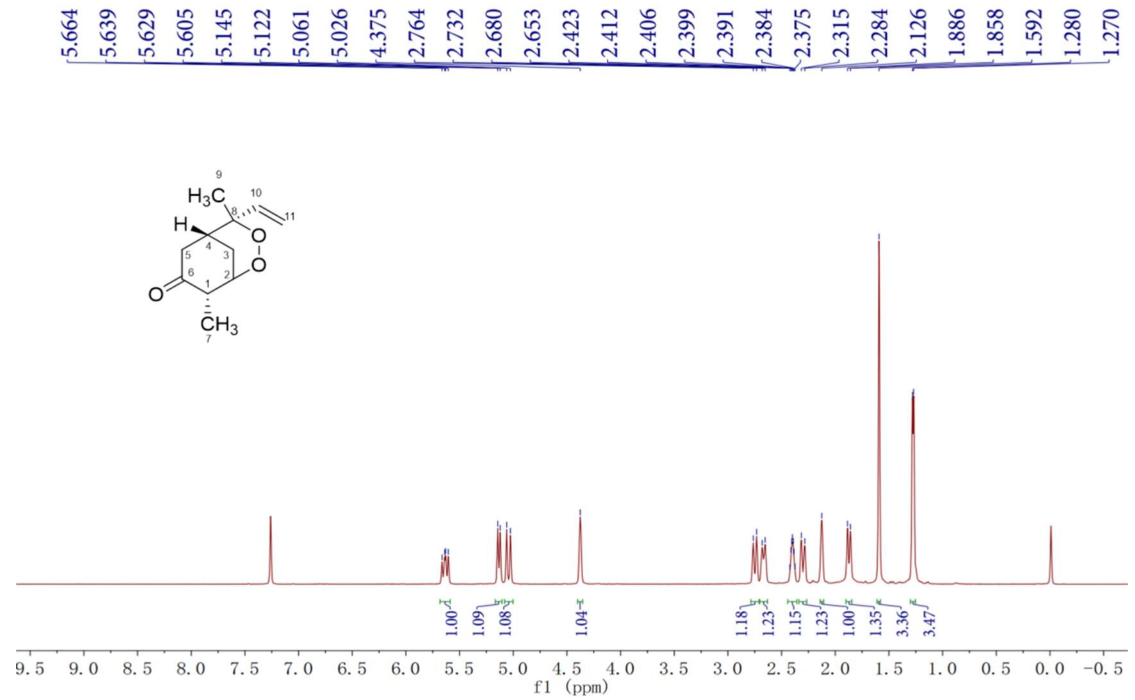

Fig. S30 ^{1}H NMR spectrum of compound III-2 in $CDCl_3$ (500 MHz)

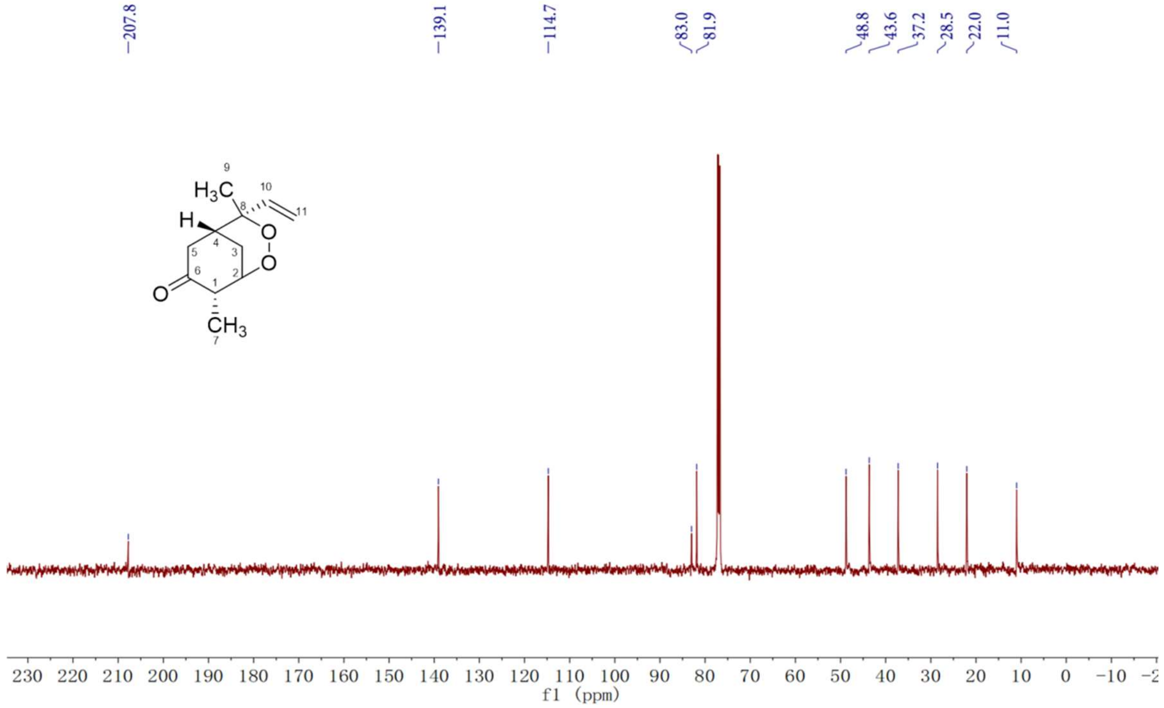

Fig. S31 ^{13}C NMR spectrum of compound III-2 in $CDCl_3$ (125 MHz)

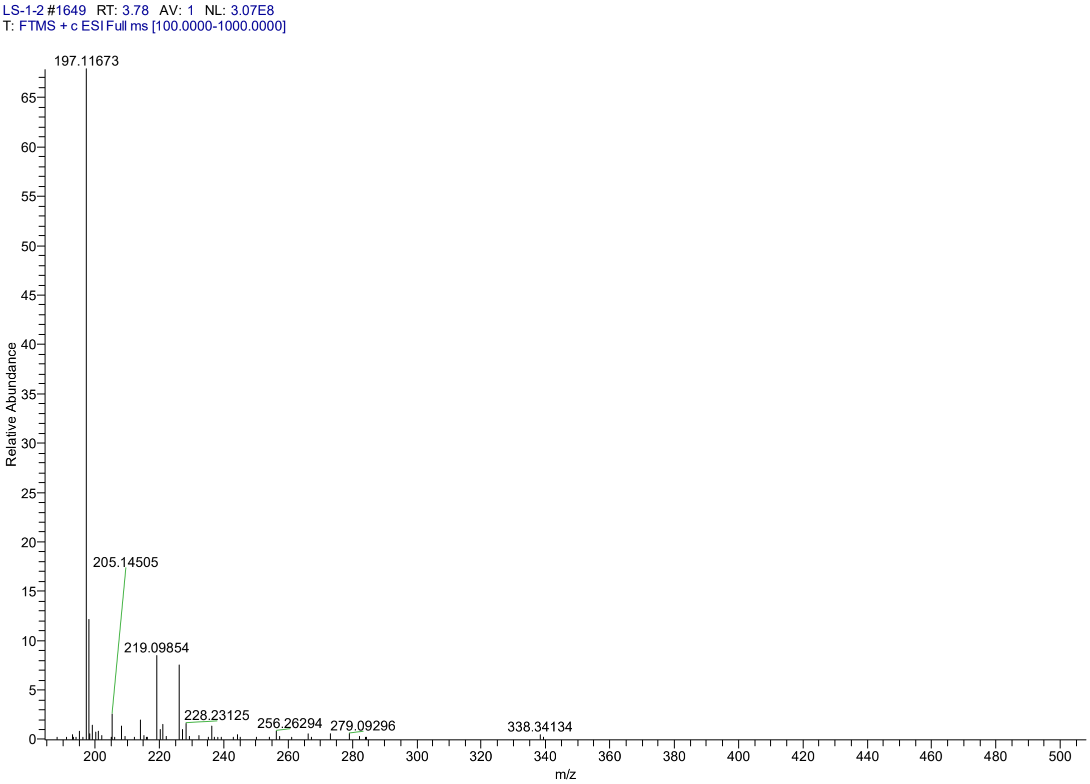

Fig. S32 HRMS data of compound III-2

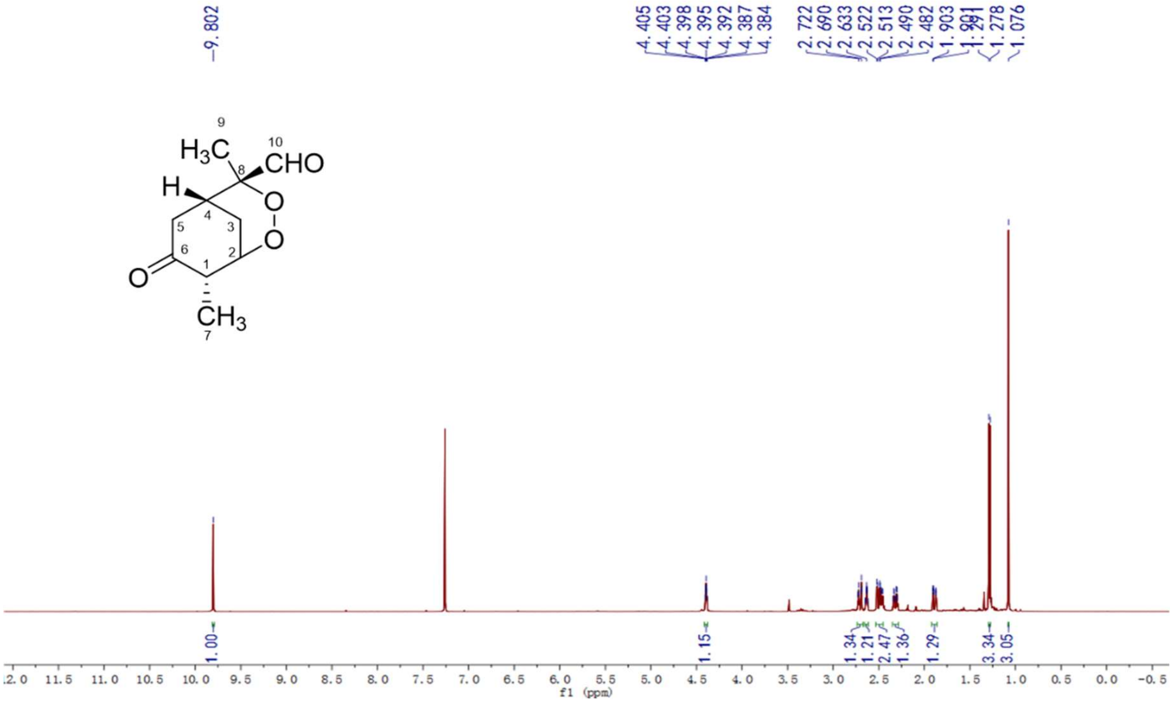

Fig. S33 ^{1}H NMR spectrum of compound IV-1 in CDCl$_3$ (500 MHz)

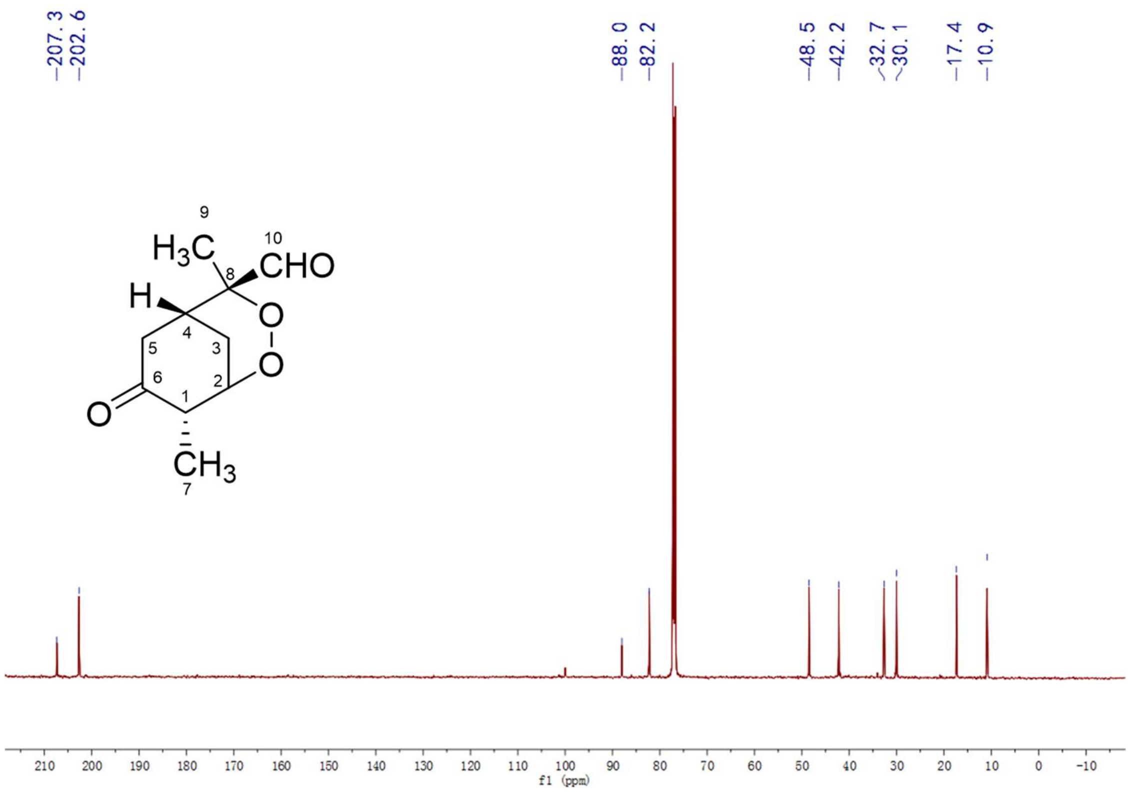

Fig. S34 ^{13}C NMR spectrum of compound IV-1 in $CDCl_3$ (125 MHz)

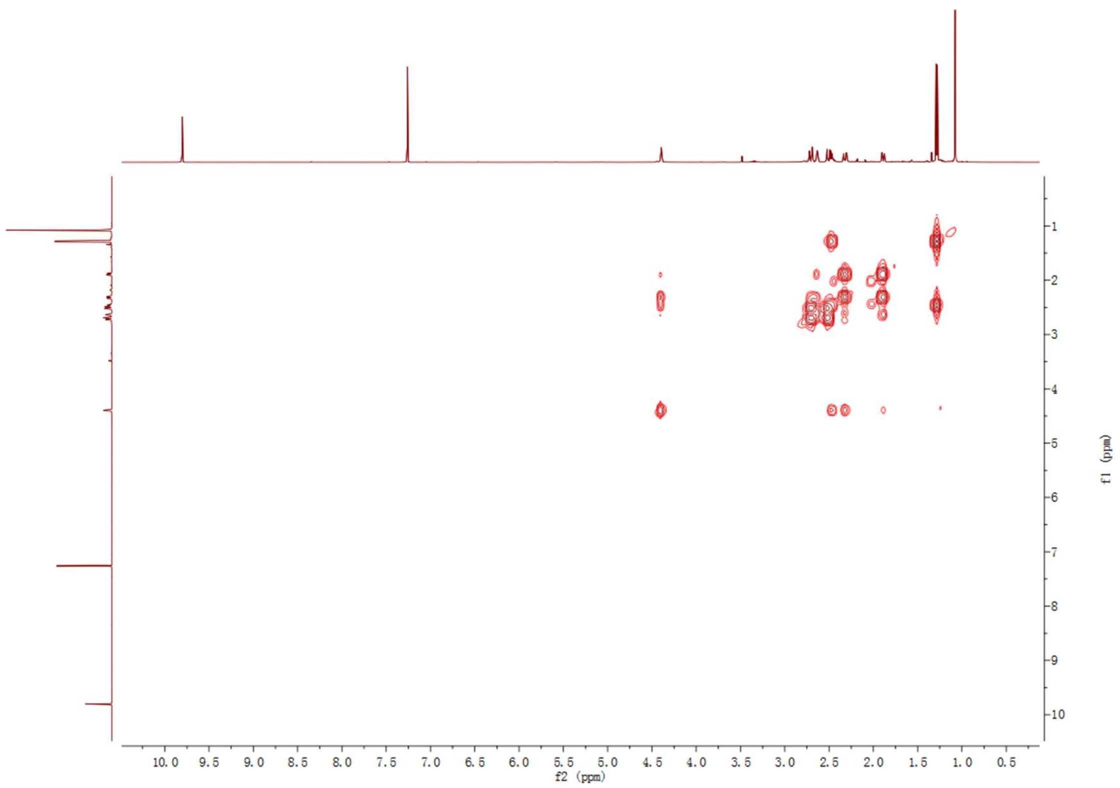

Fig. S35 ^{1}H-^{1}H COSY spectrum of compound IV-1 in $CDCl_3$ (500 MHz)

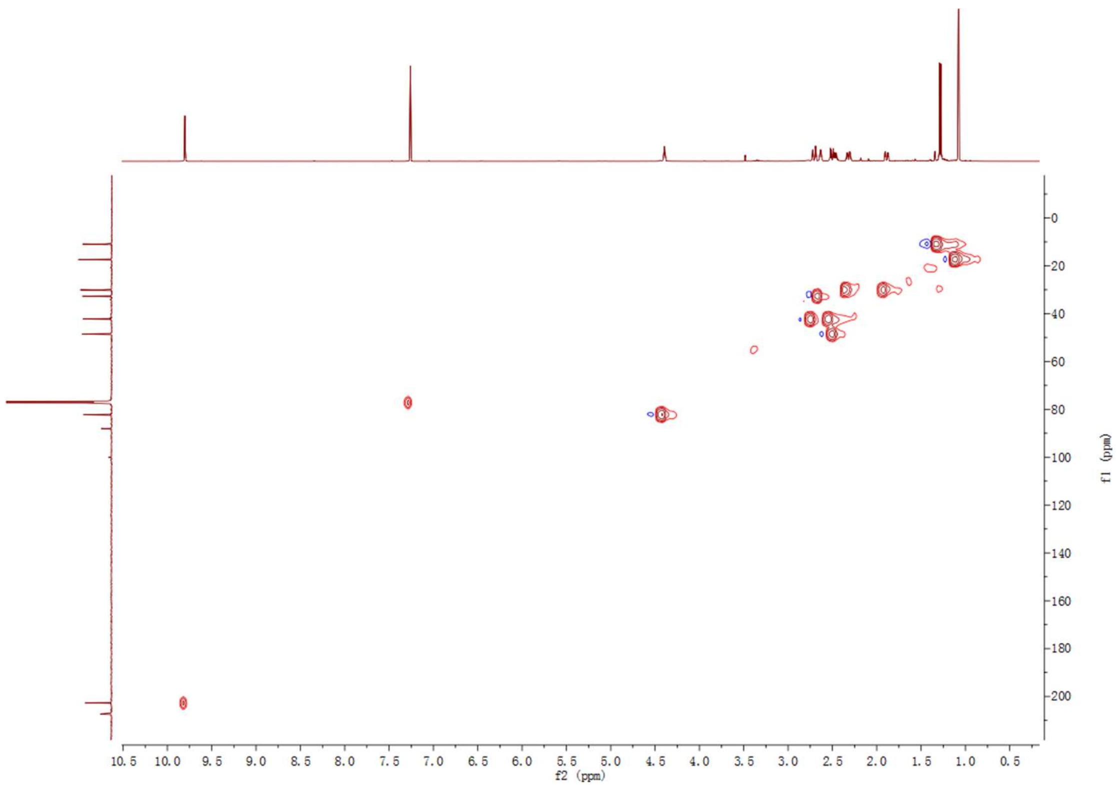

Fig. S36 HSQC spectrum of compound IV-1 in $CDCl_3$ (500 MHz)

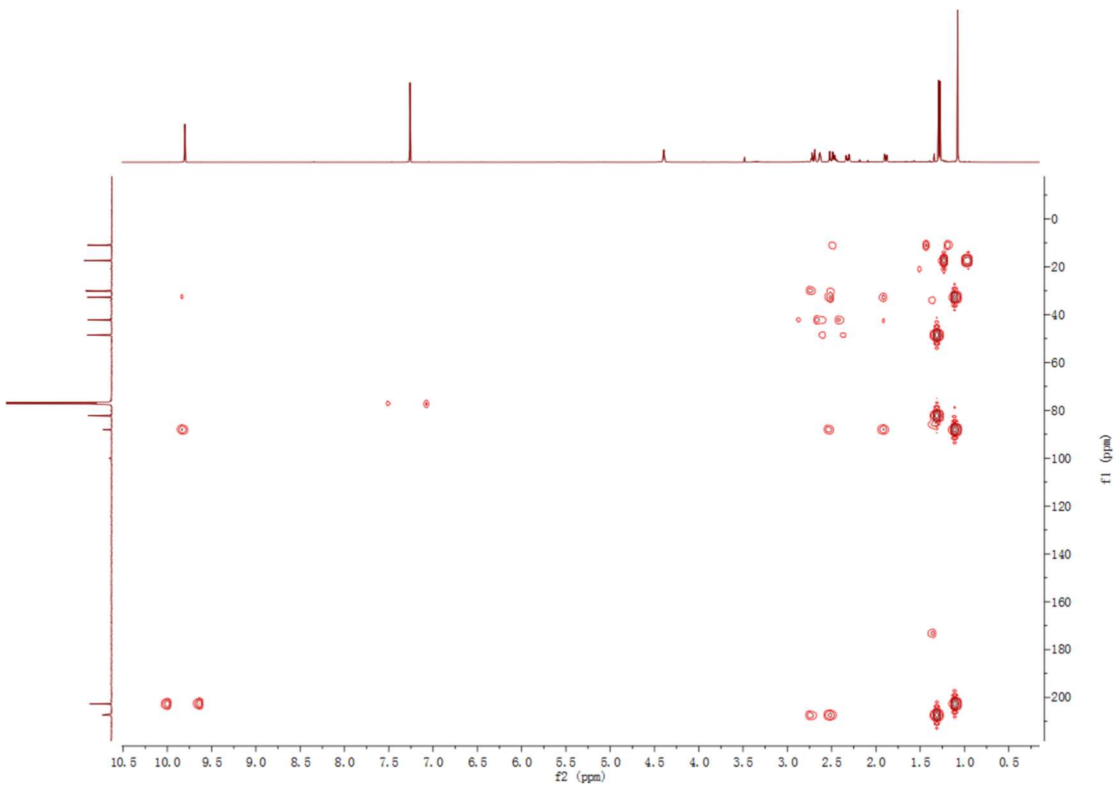

Fig. S37 HMBC spectrum of compound IV-1 in $CDCl_3$ (500 MHz)

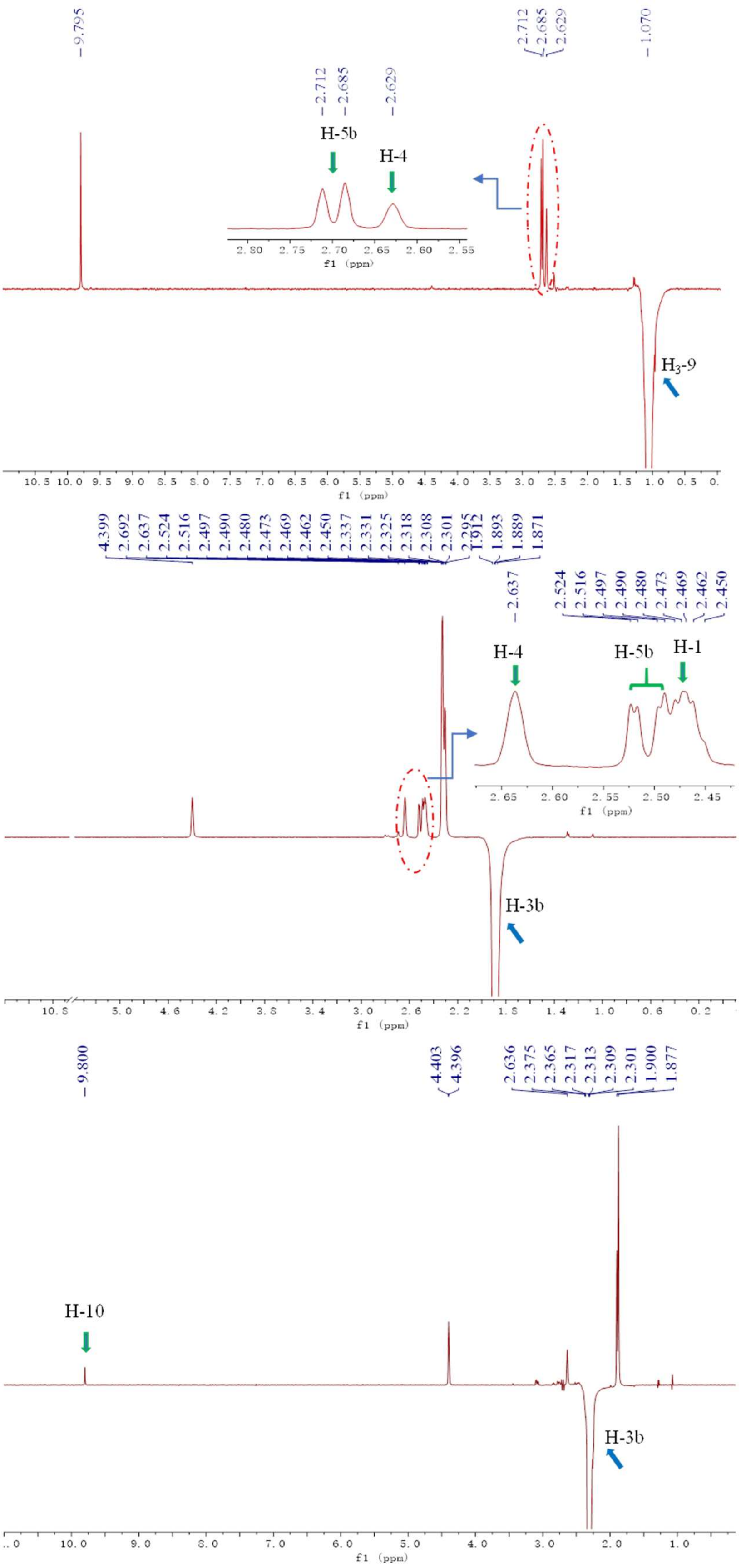

Fig. S38 NOE difference spectra of compound IV-1 in $CDCl_3$ (600 MHz)

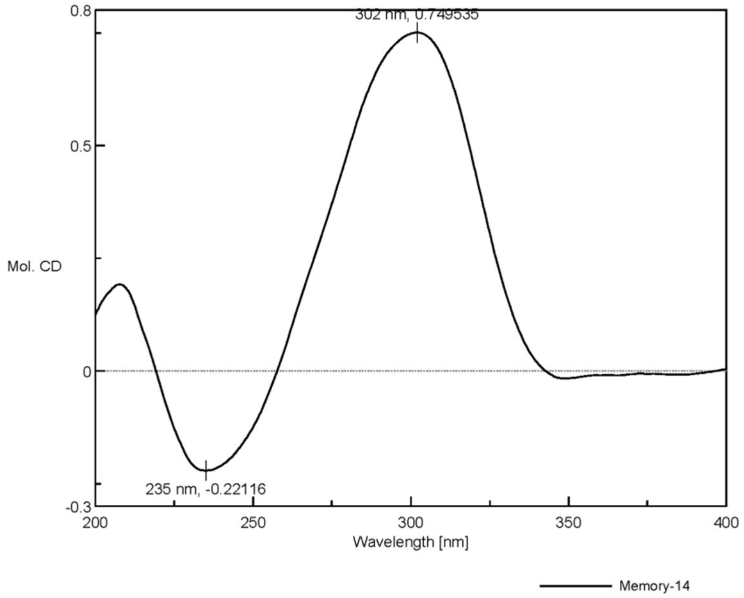

Fig. S39 CD spectrum of compound IV-1 in MeOH

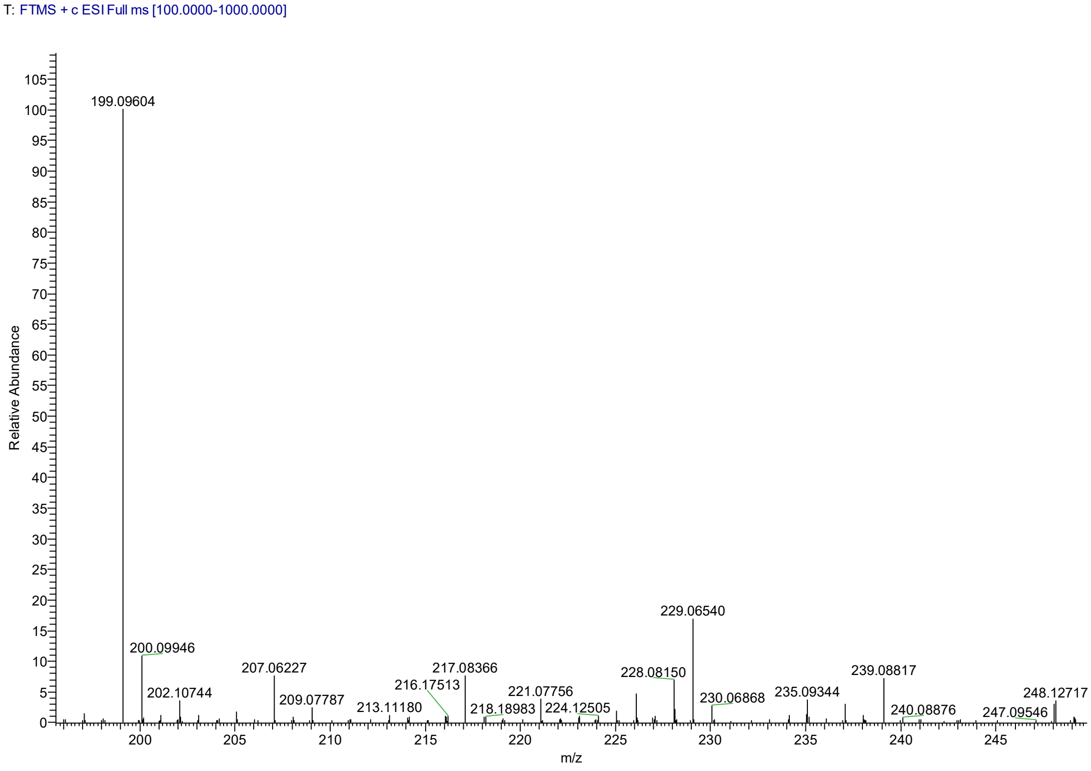

Fig. S40 HRMS data of compound IV-1

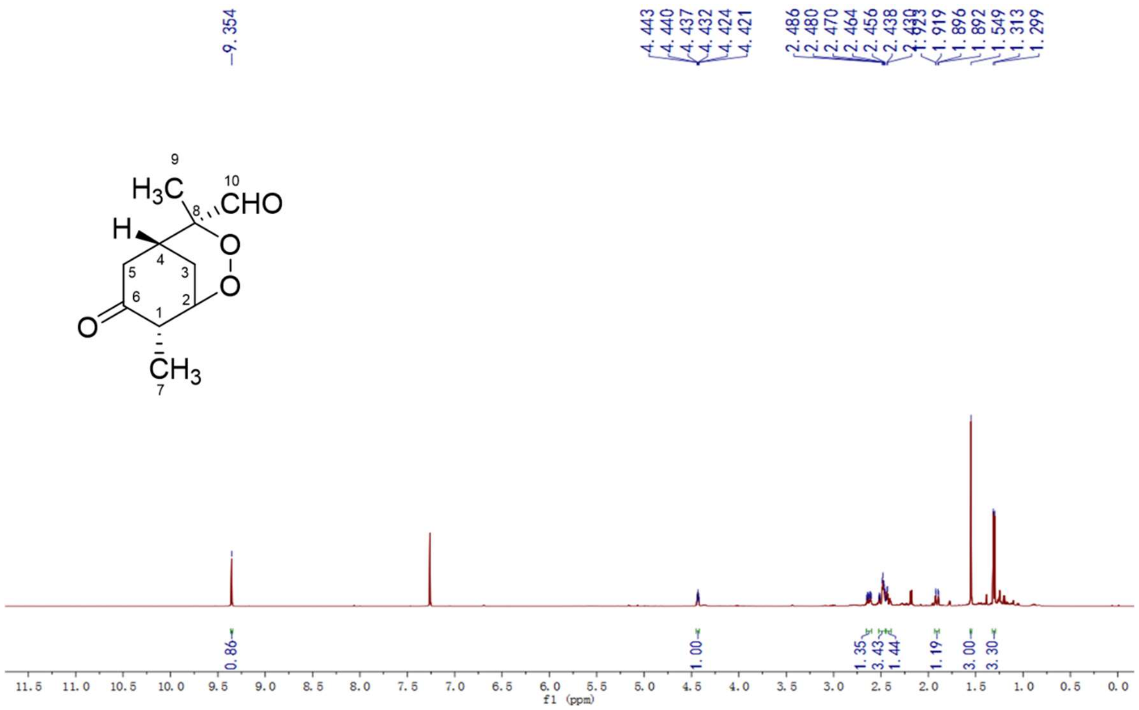

Fig. S41 ^{1}H NMR spectrum of compound IV-2 in $CDCl_3$ (500 MHz)

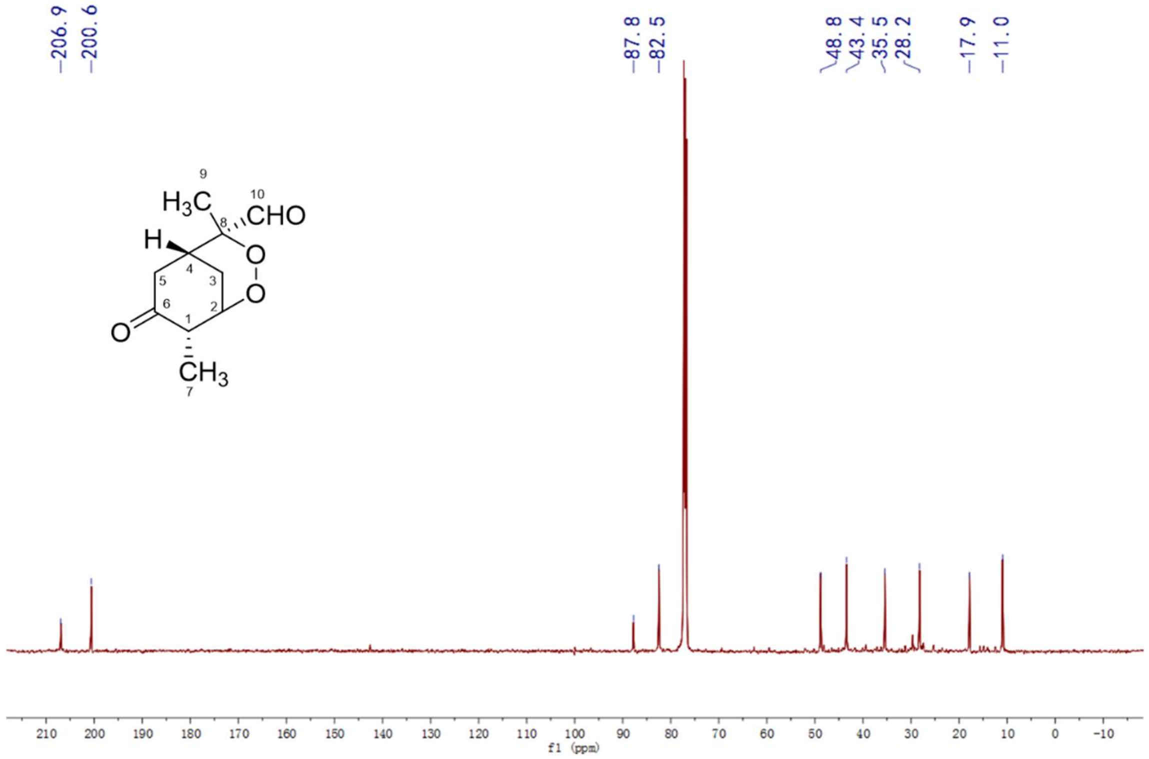

Fig. S42 ^{13}C NMR spectrum of compound IV-2 in $CDCl_3$ (125 MHz)

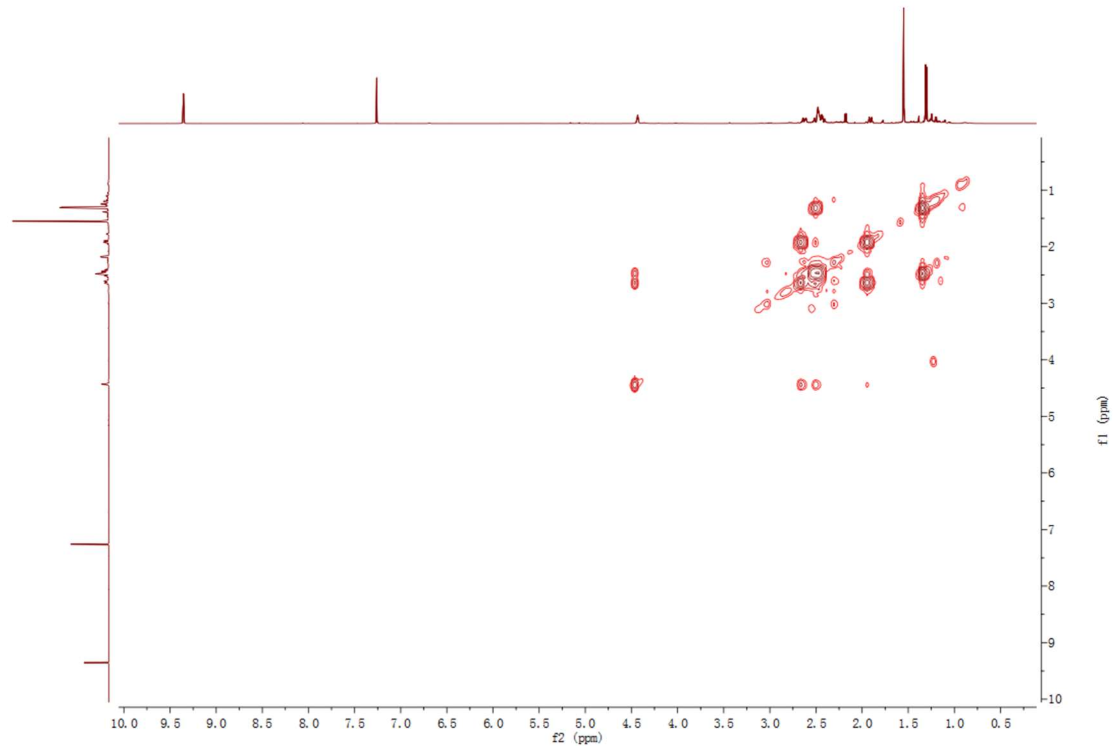

Fig. S43 ^{1}H-^{1}H COSY spectrum of compound IV-2 in $CDCl_3$ (500 MHz)

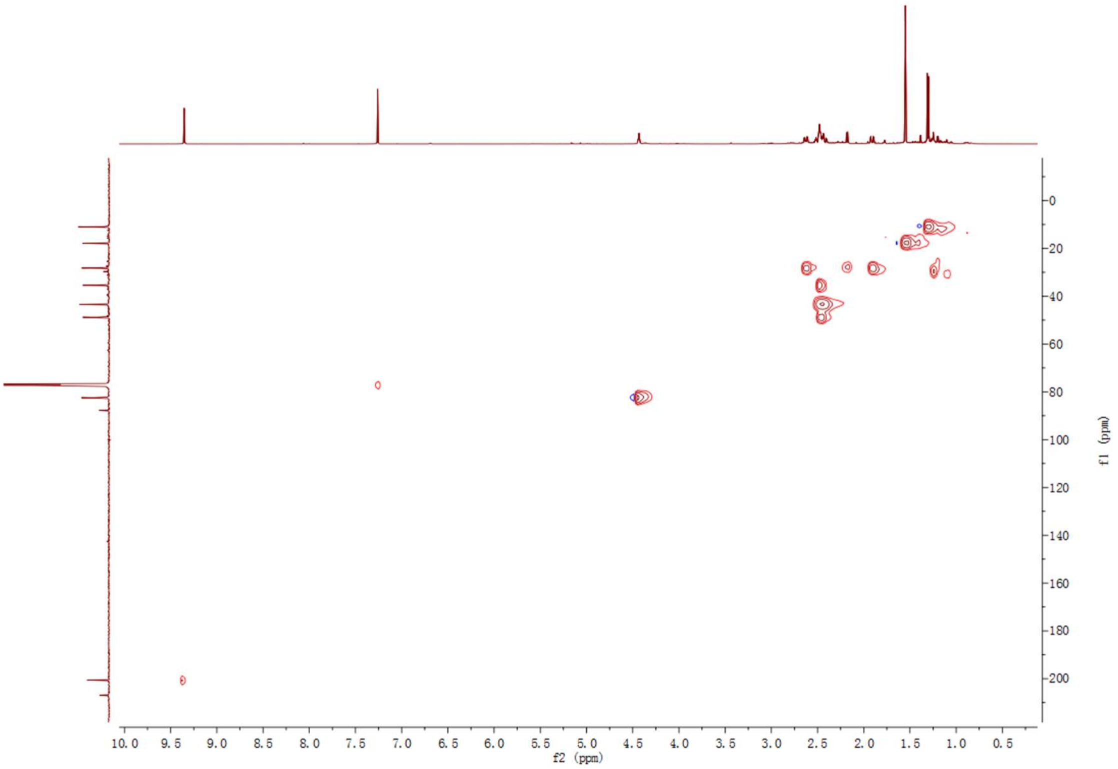

Fig. S44 HSQC spectrum of compound IV-2 in $CDCl_3$ (500 MHz)

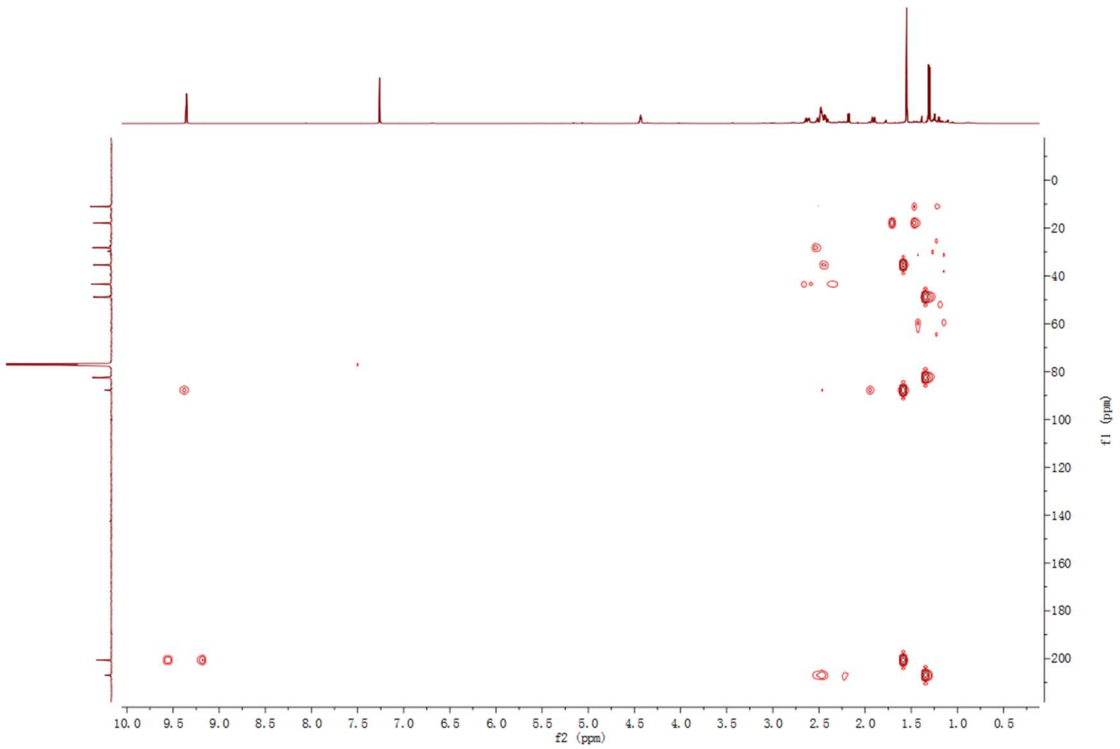

Fig. S45 HMBC spectrum of compound IV-2 in CDCl3 (500 MHz)

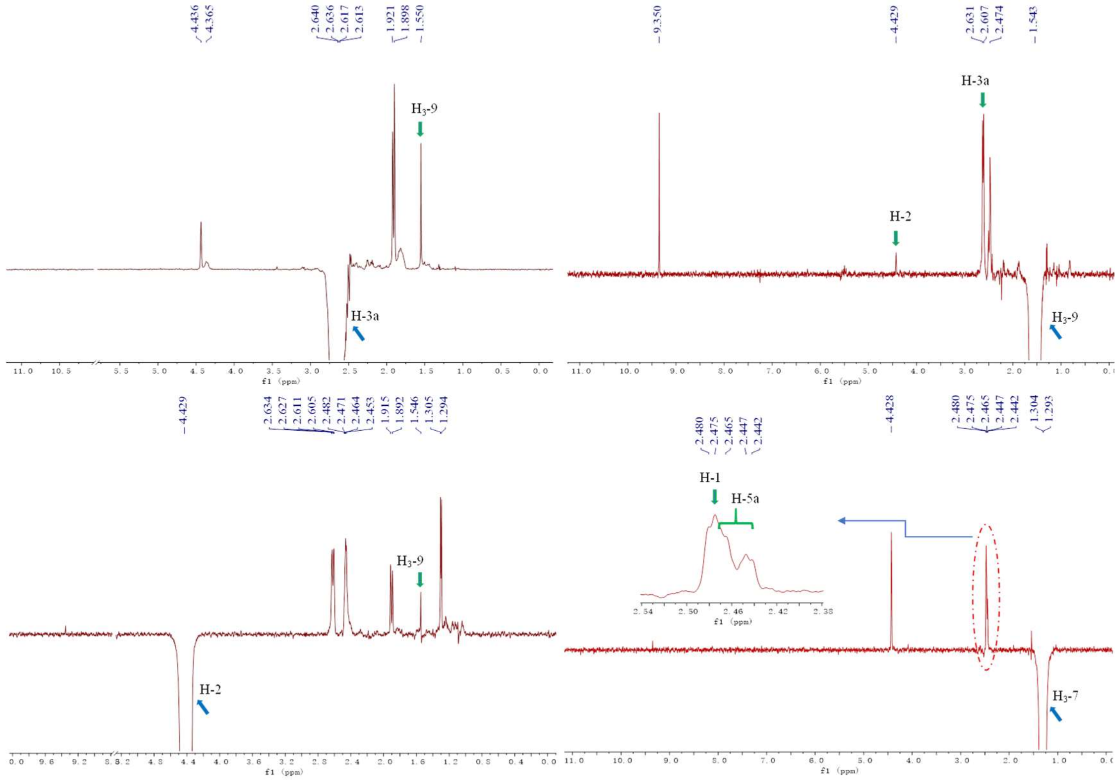

Fig. S46 NOE difference spectra of compound IV-2 in CDCl3 (600 MHz)

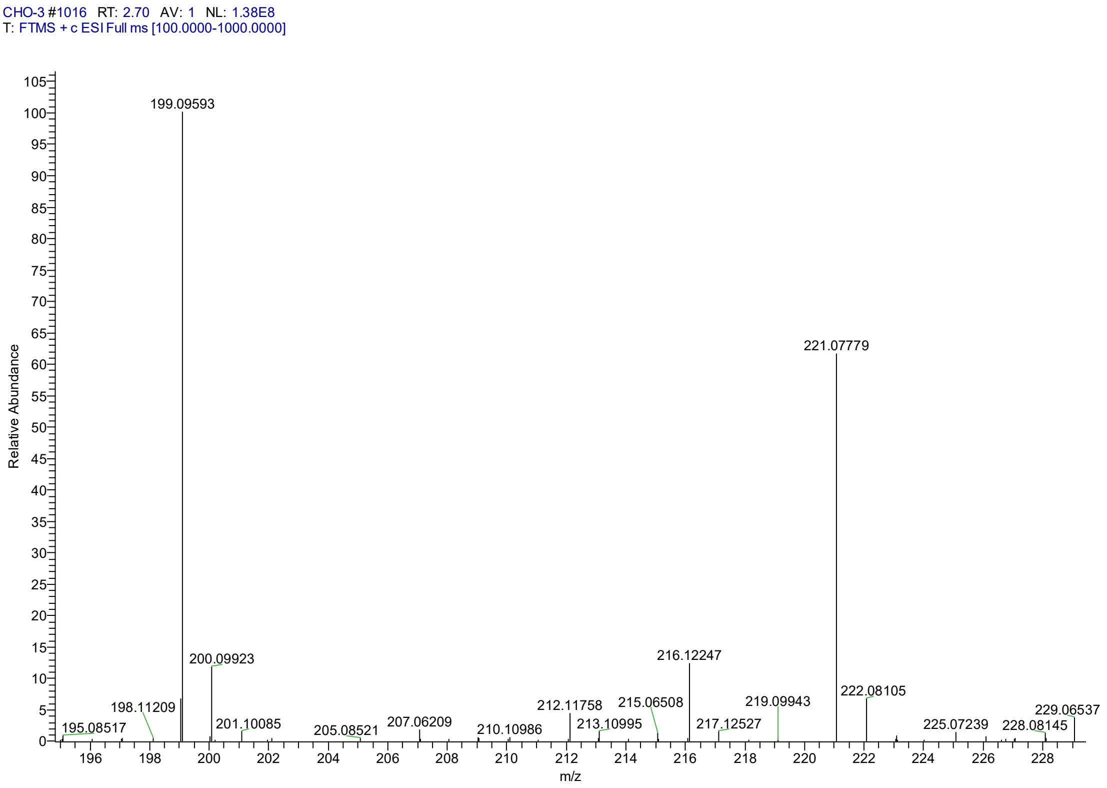

Fig. S47 HRMS data of compound IV-2

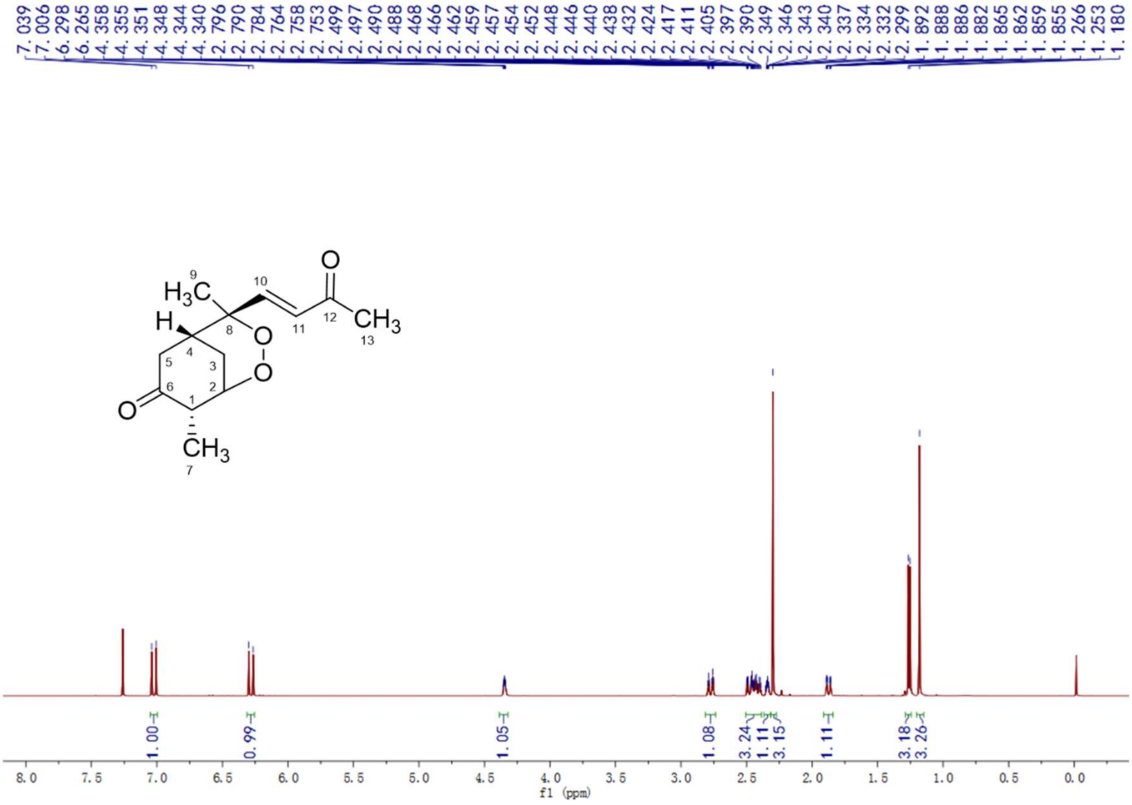

Fig. S48 ^{1}H NMR spectrum of compound V-1 in $CDCl_3$ (500 MHz)

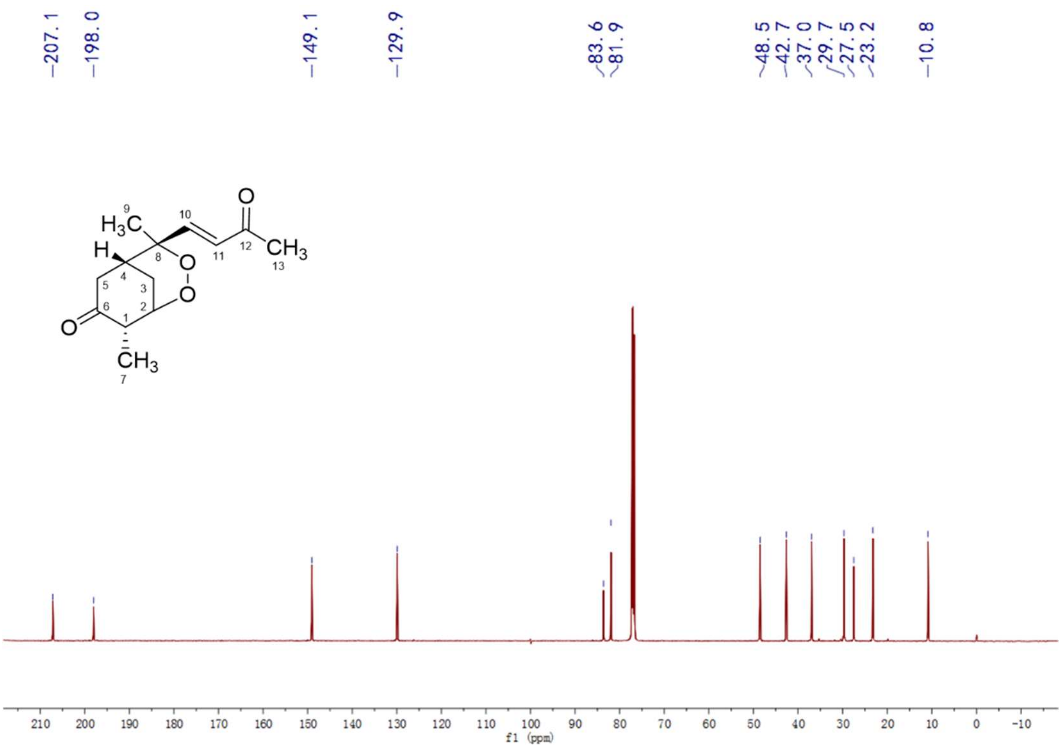

Fig. S49 ^{13}C NMR spectrum of compound V-1 in $CDCl_3$ (125 MHz)

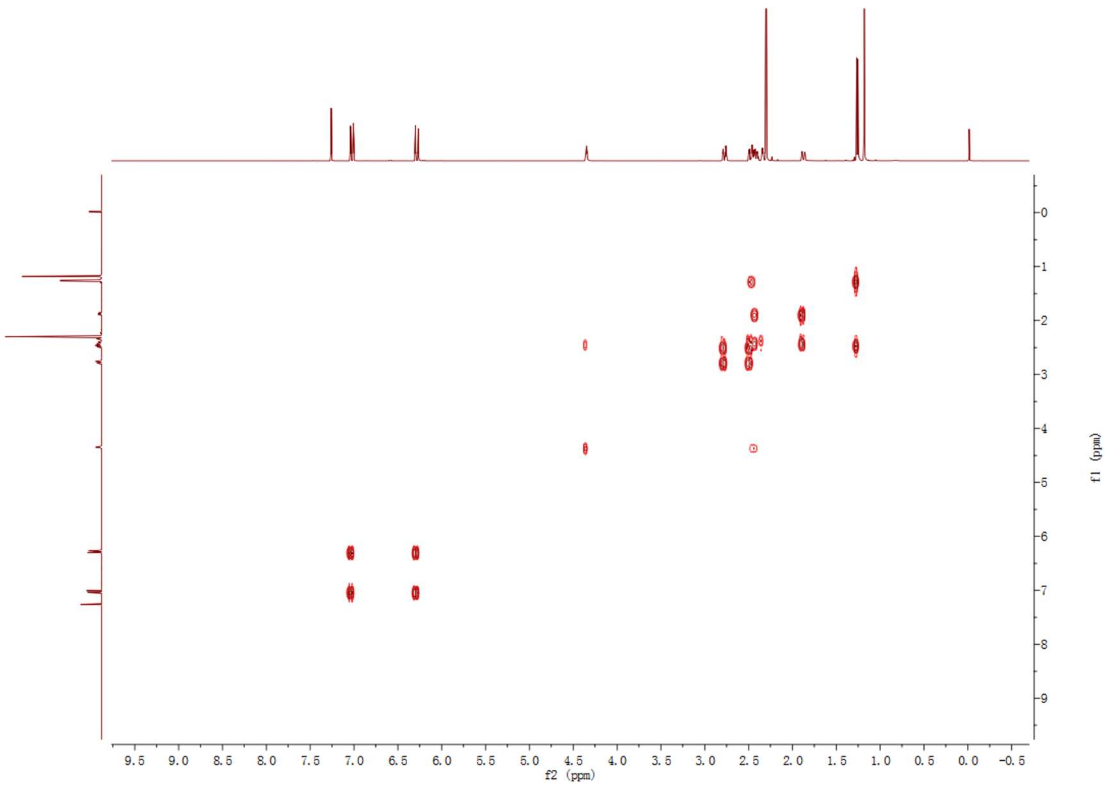

Fig. S50 ^{1}H-^{1}H COSY spectrum of compound V-1 in $CDCl_3$ (500 MHz)

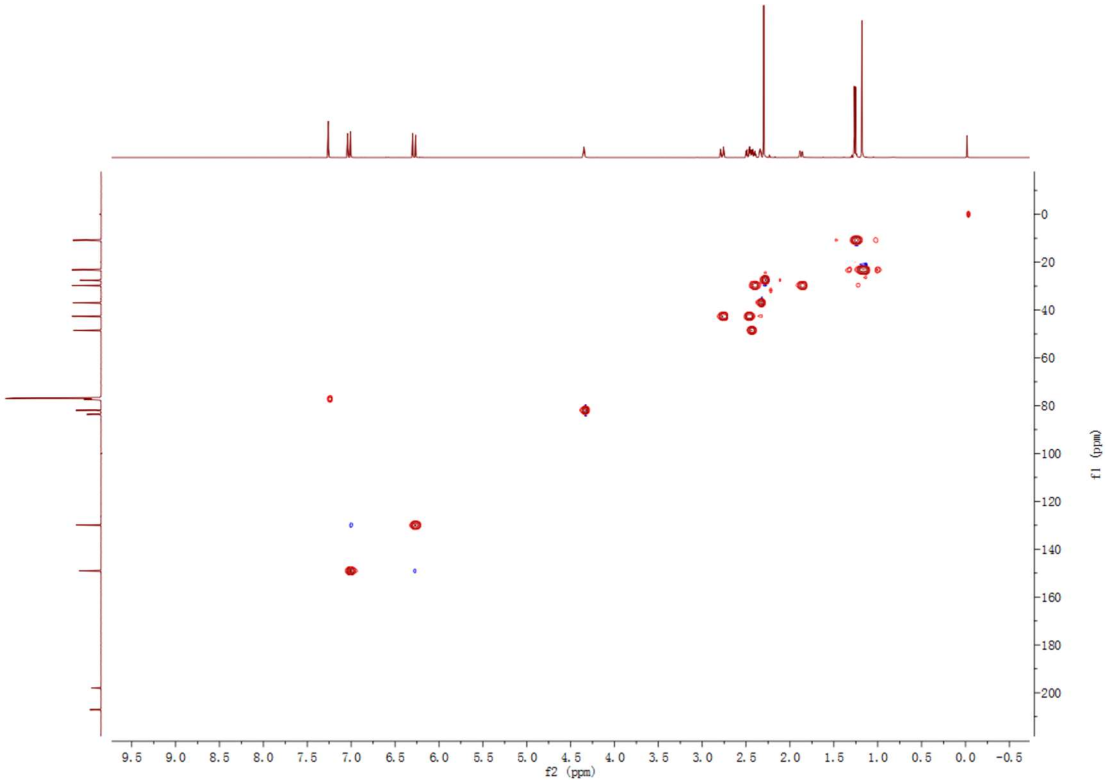

Fig. S51 HSQC spectrum of compound V-1 in $CDCl_3$ (500 MHz)

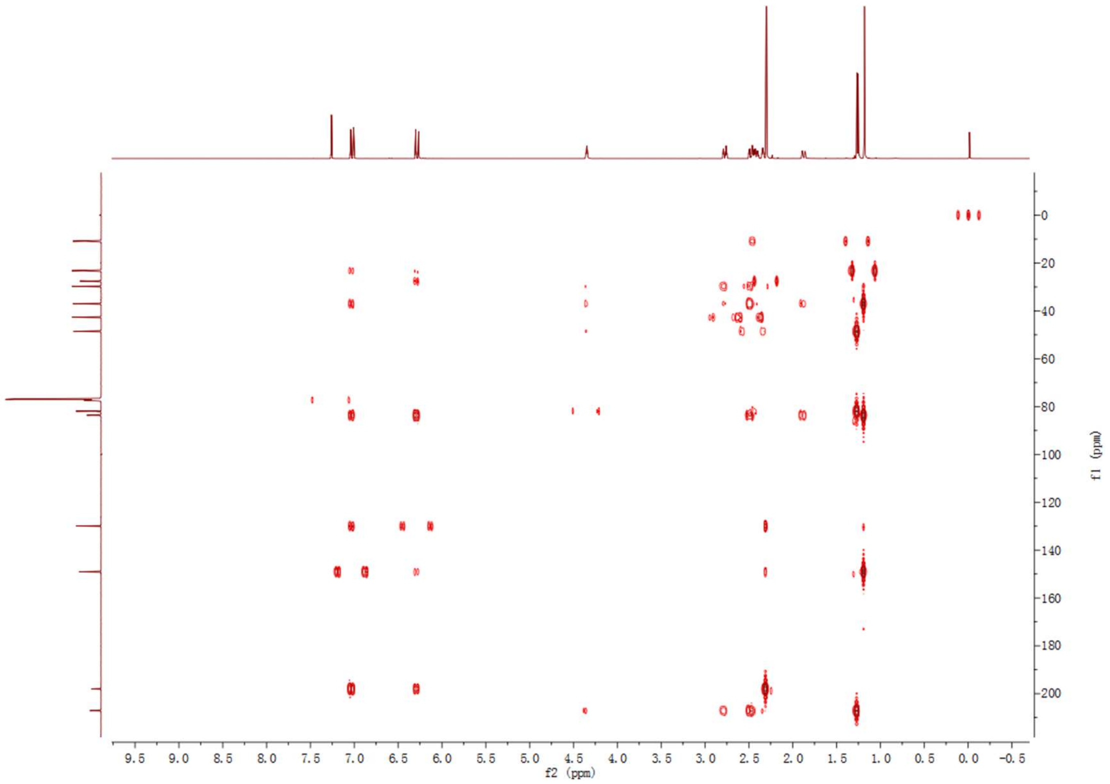

Fig. S52 HMBC spectrum of compound V-1 in $CDCl_3$ (500 MHz)

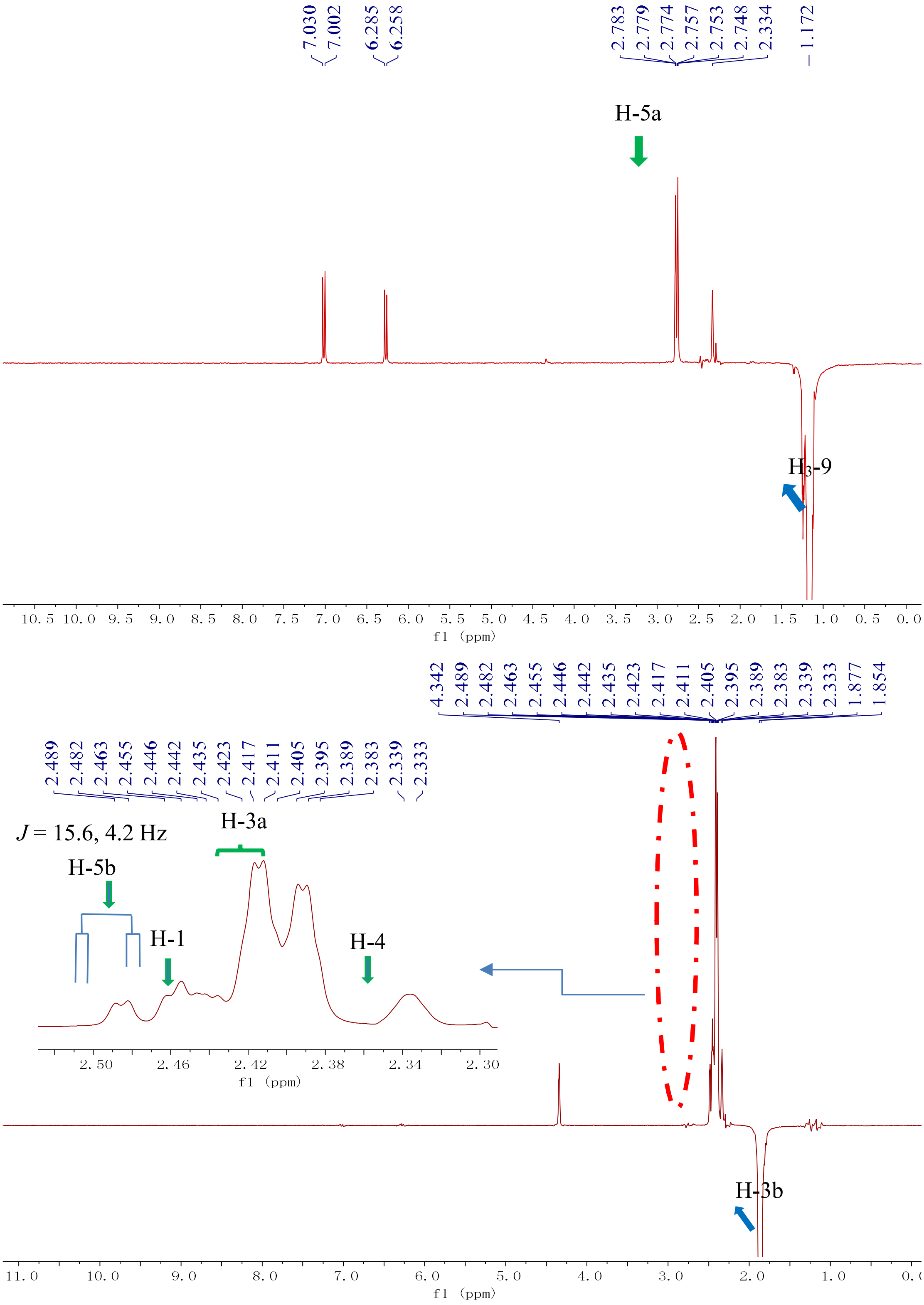

Fig. S53 NOE difference spectra of compound V-1 in $CDCl_3$ (600 MHz)

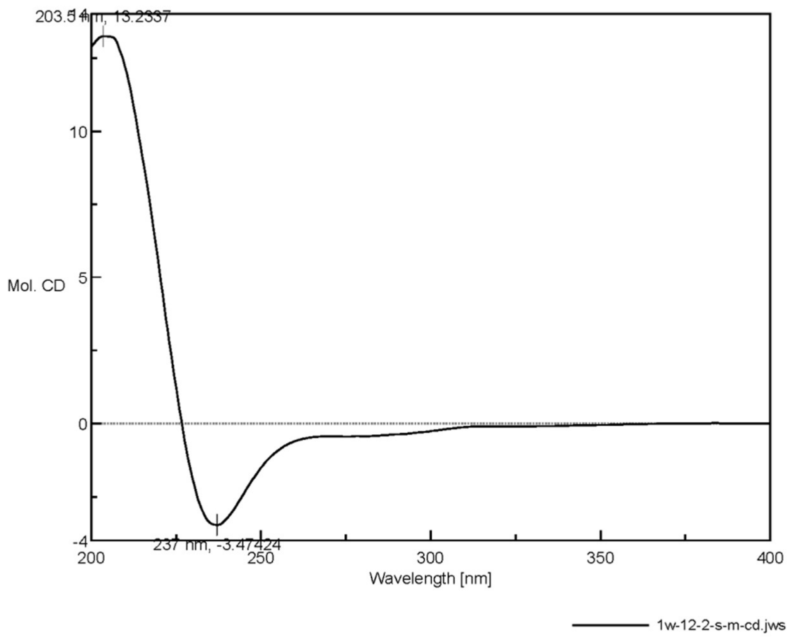

Fig. S54 CD spectrum of compound V-1 in MeOH

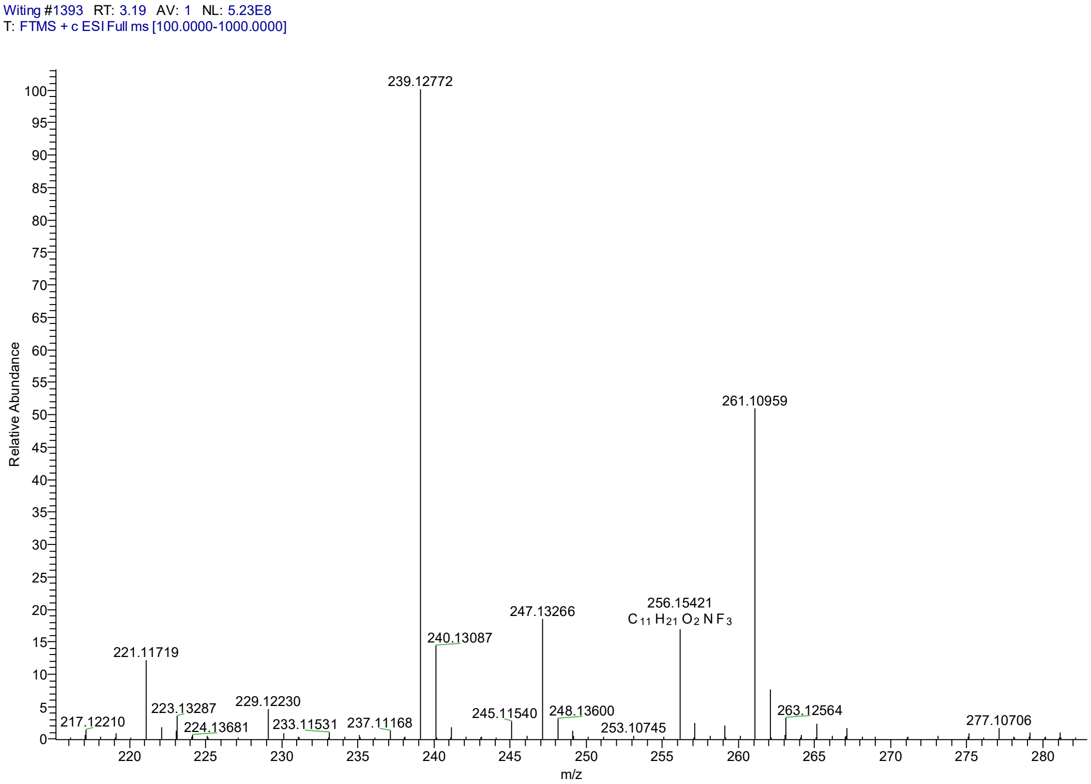

Fig. S55 HRMS data of compound V-1

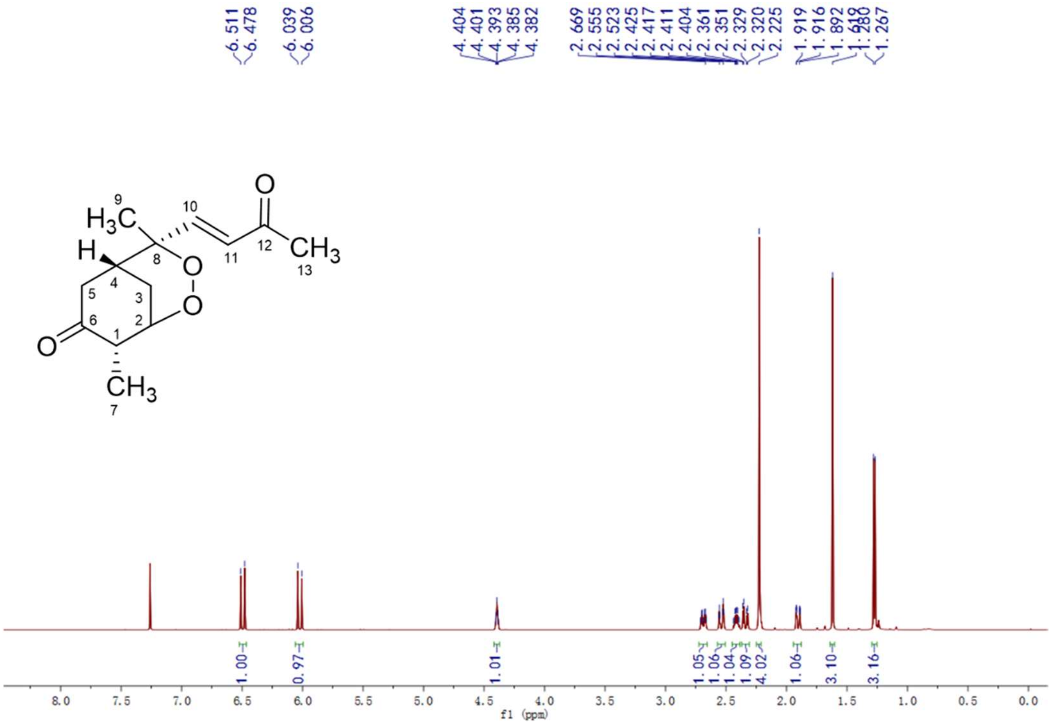

Fig. S56 ^{1}H NMR spectrum of compound V-2 in $CDCl_3$ (500 MHz)

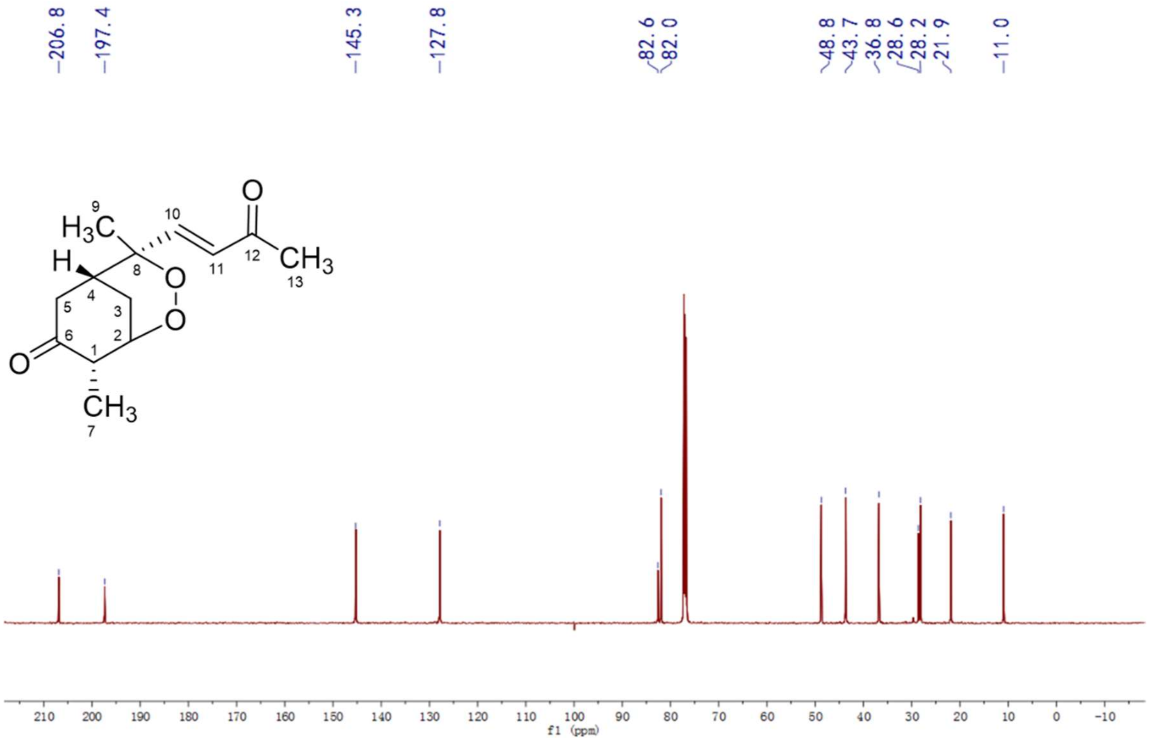

Fig. S57 ^{13}C NMR spectrum of compound V-2 in $CDCl_3$ (125 MHz)

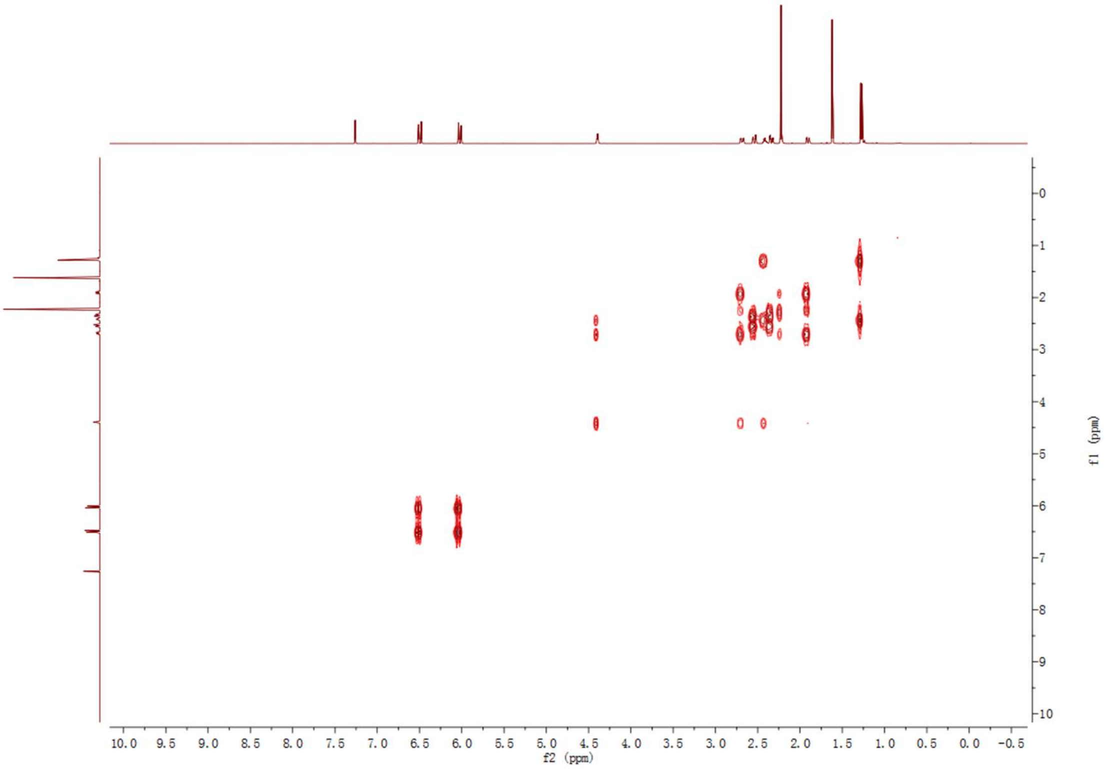

Fig. S58 ^{1}H-^{1}H COSY spectrum of compound V-2 in $CDCl_3$ (500 MHz)

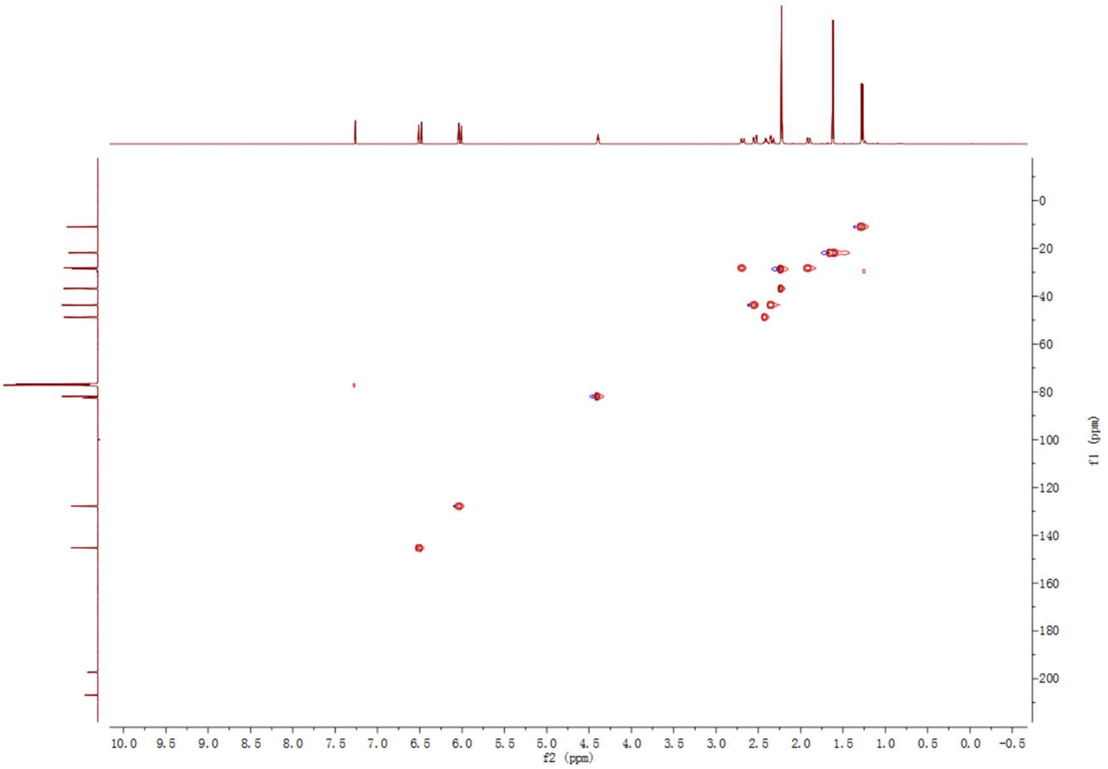

Fig. S59 HSQC spectrum of compound V-2 in $CDCl_3$ (500 MHz)

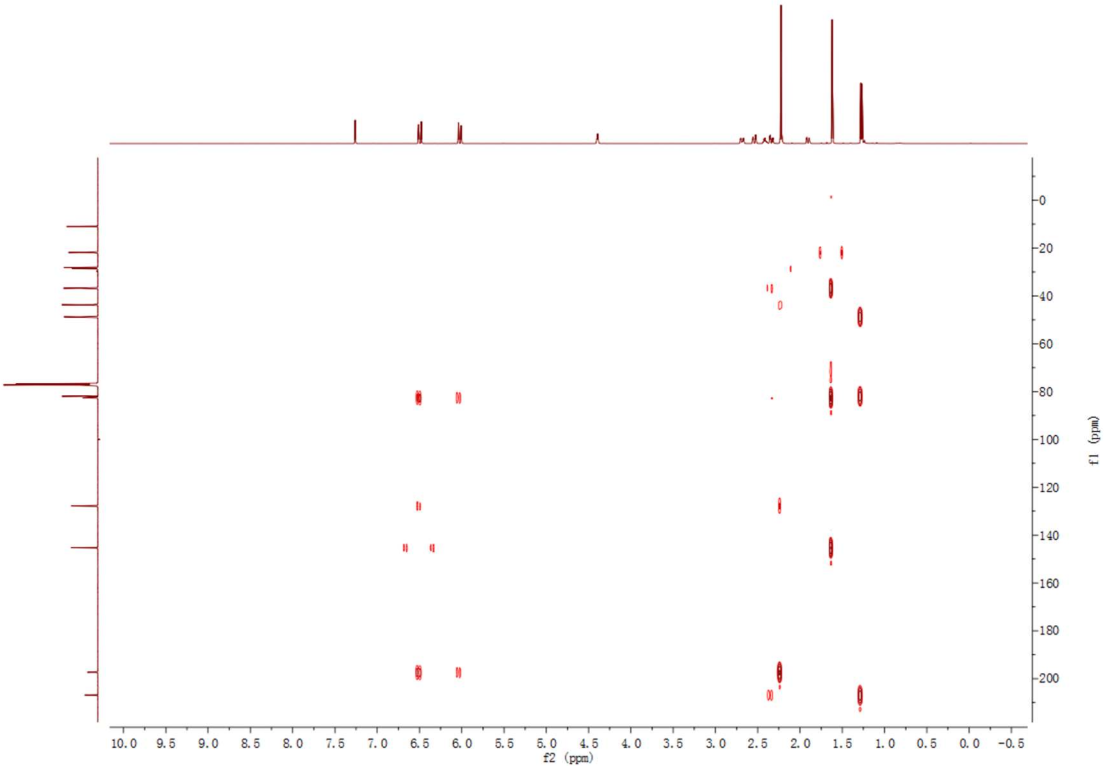

Fig. S60 HMBC spectrum of compound V-2 in CDCl₃ (500 MHz)

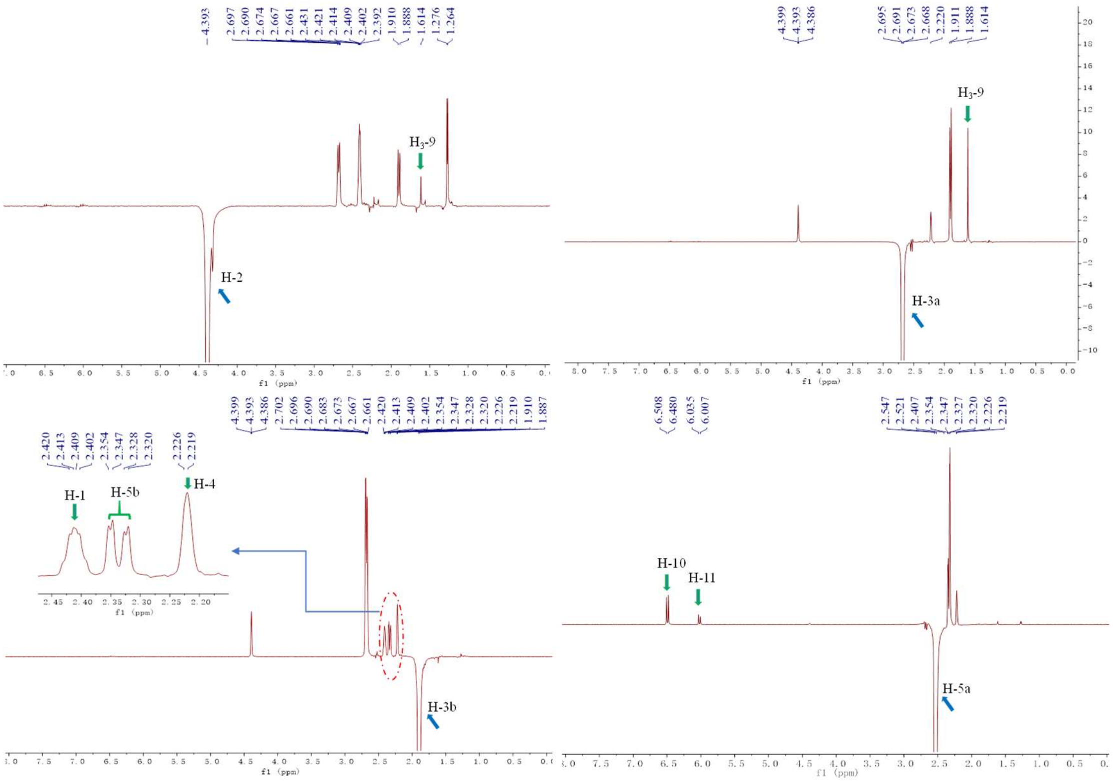

Fig. S61 NOE difference spectra of compound V-2 in CDCl₃ (600 MHz)

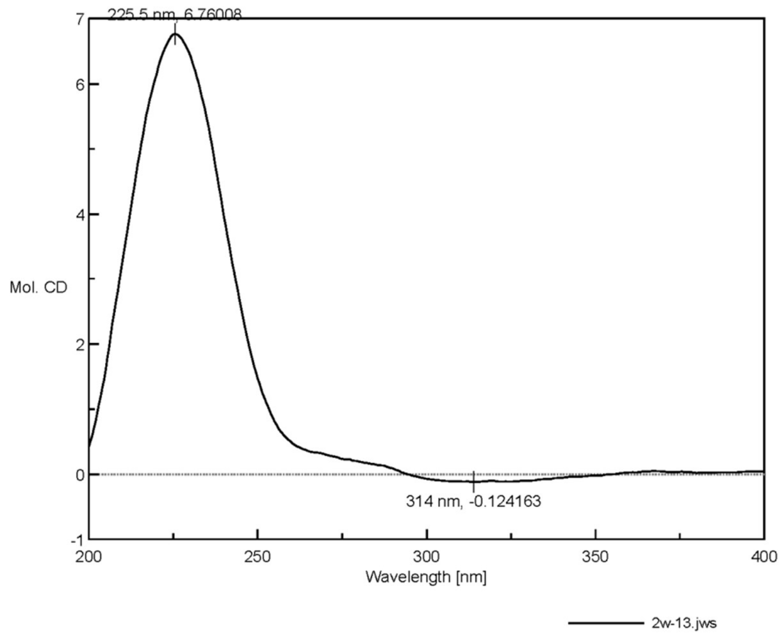

Fig. S62 CD spectrum of compound V-2 in MeOH

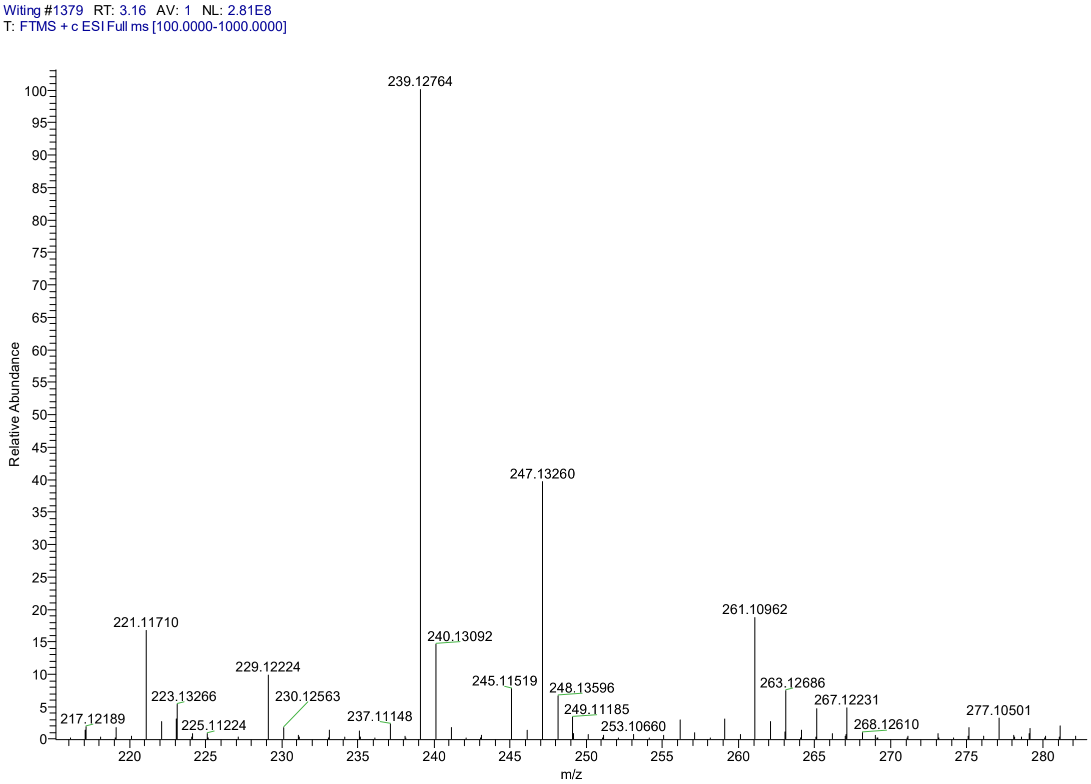

Fig. S63 HRMS data of compound V-2

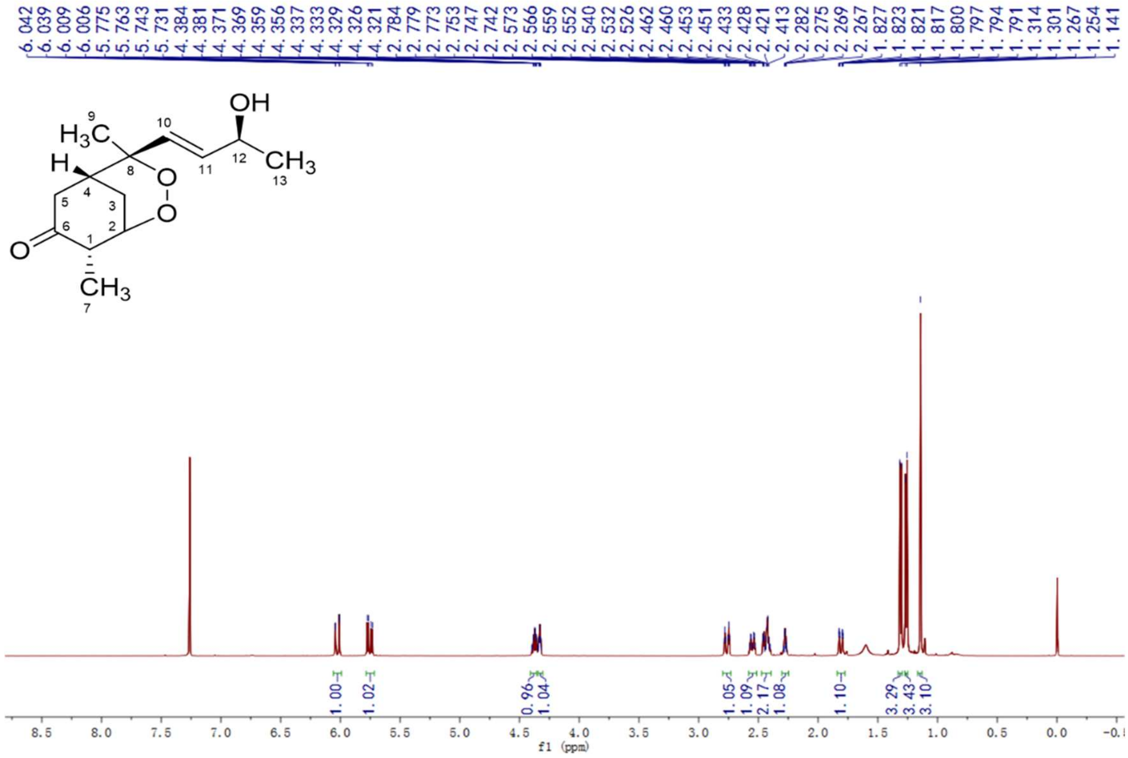

Fig. S64 ^{1}H NMR spectrum of compound VI-1a in $CDCl_3$ (500 MHz)

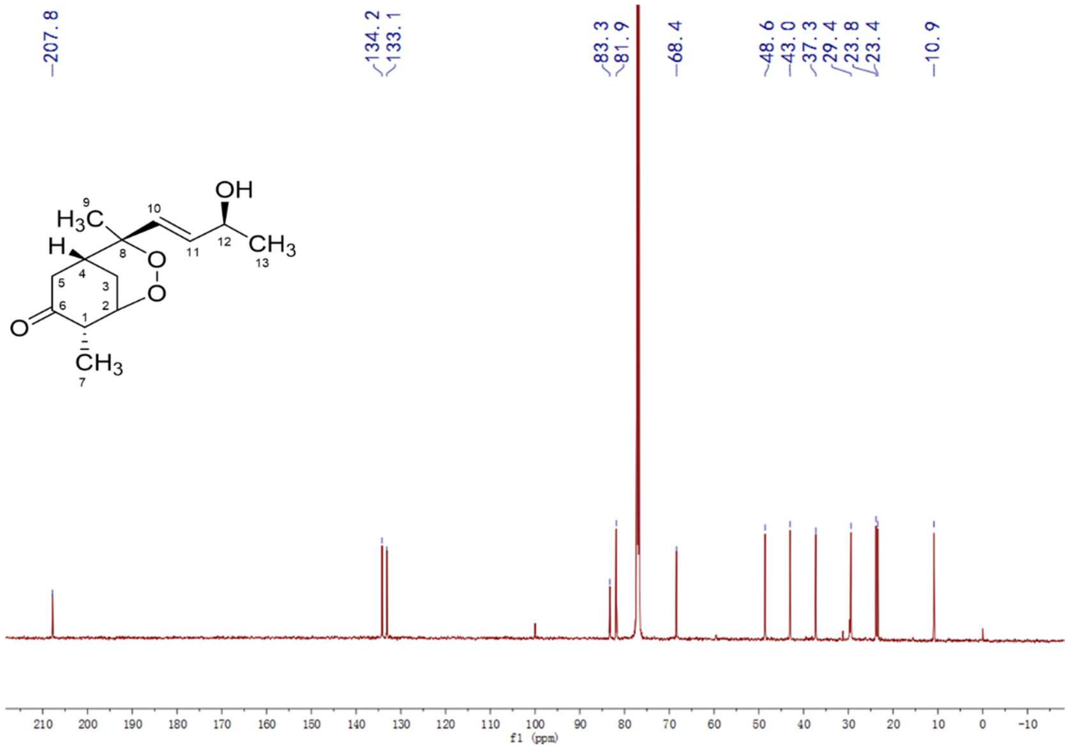

Fig. S65 ^{13}C NMR spectrum of compound VI-1a in $CDCl_3$ (125 MHz)

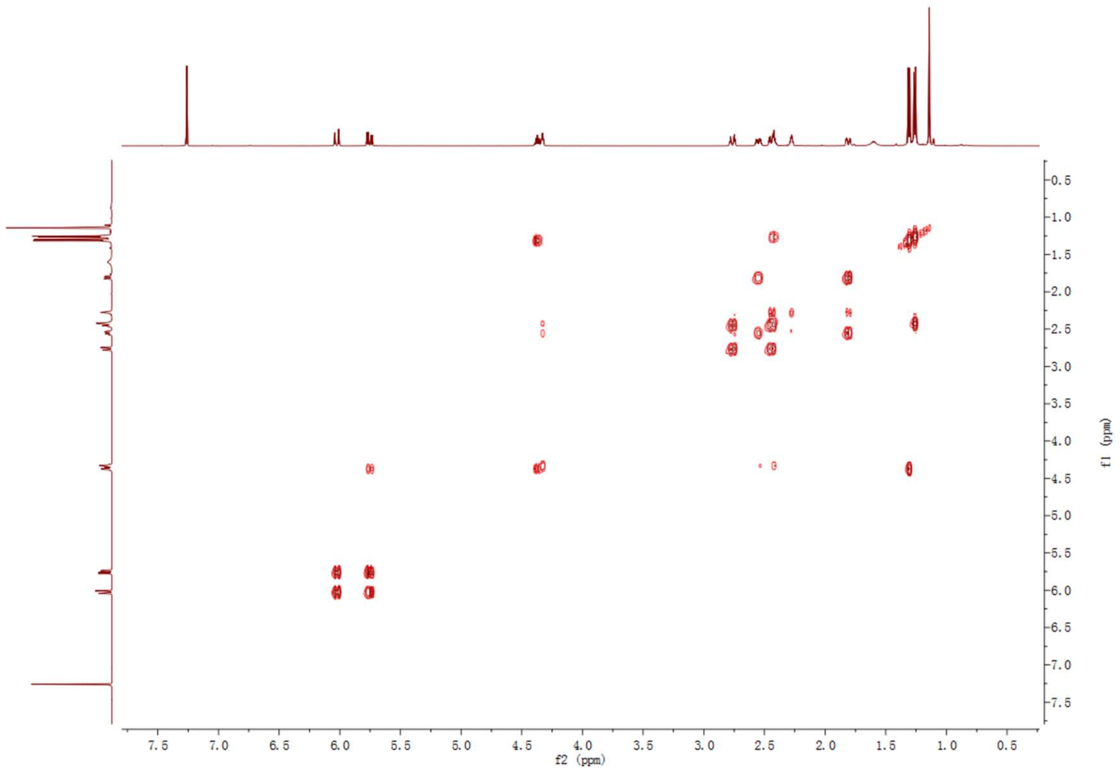

Fig. S66 ^{1}H-^{1}H COSY spectrum of compound VI-1a in $CDCl_3$ (500 MHz)

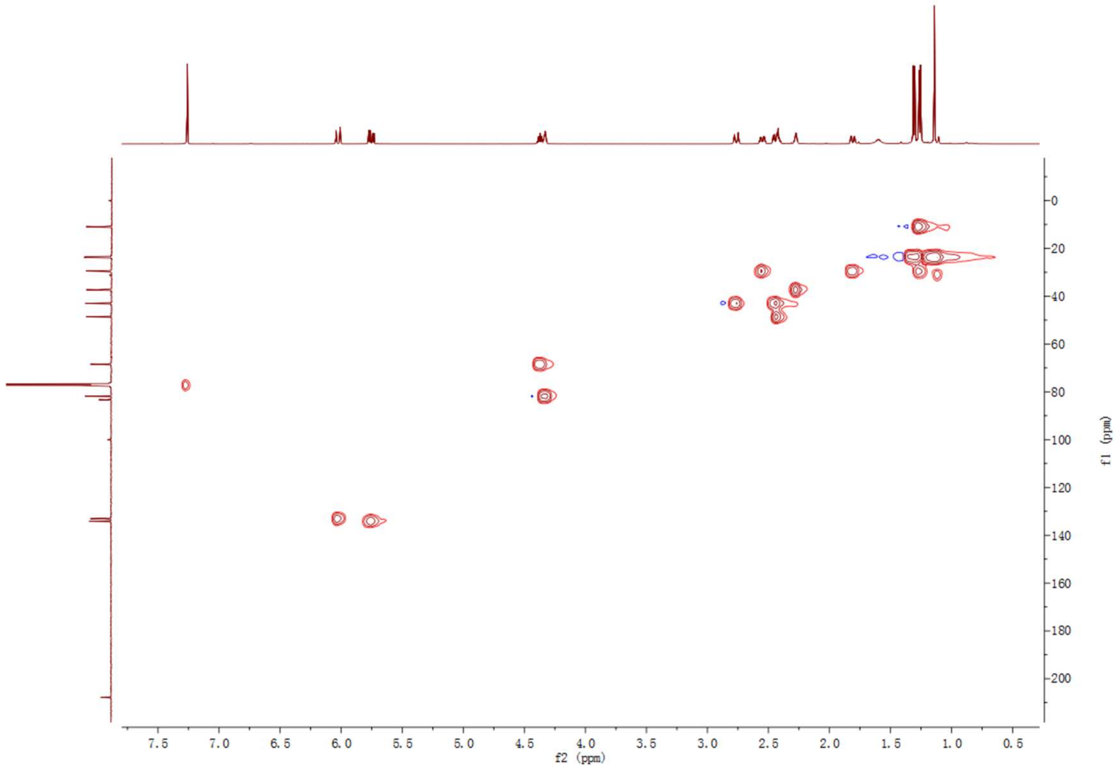

Fig. S67 HSQC spectrum of compound VI-1a in $CDCl_3$ (500 MHz)

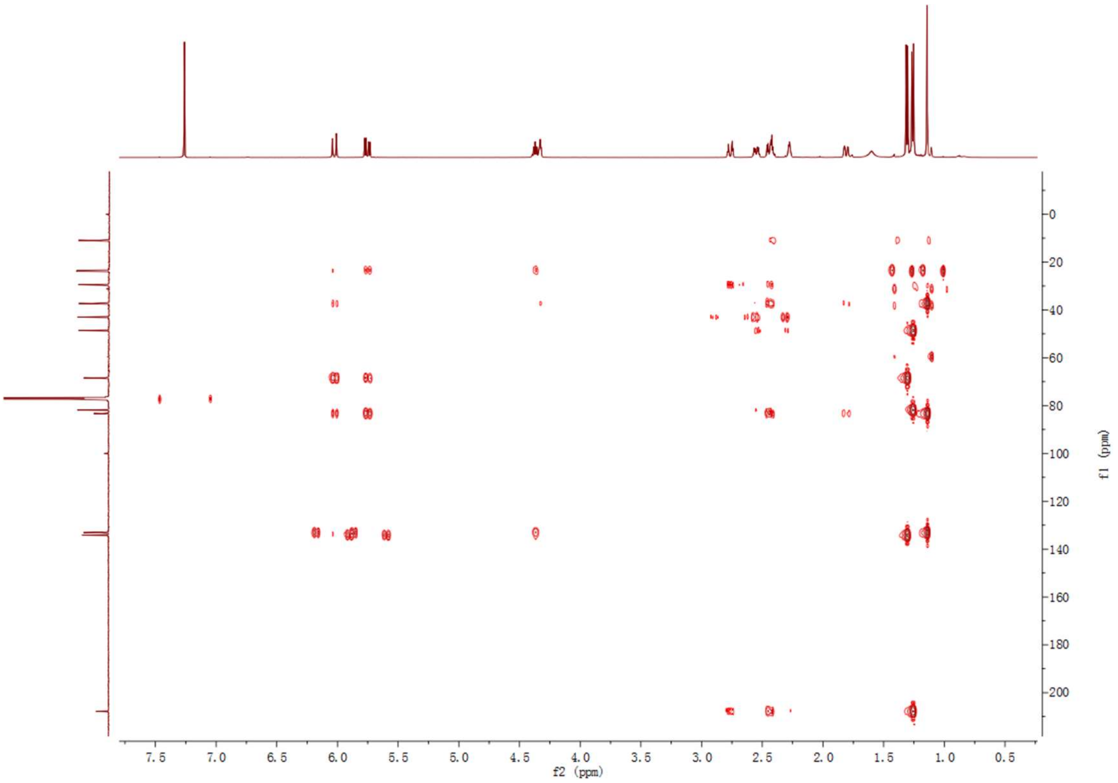

Fig. S68 HMBC spectrum of compound VI-1a in $CDCl_3$ (500 MHz)

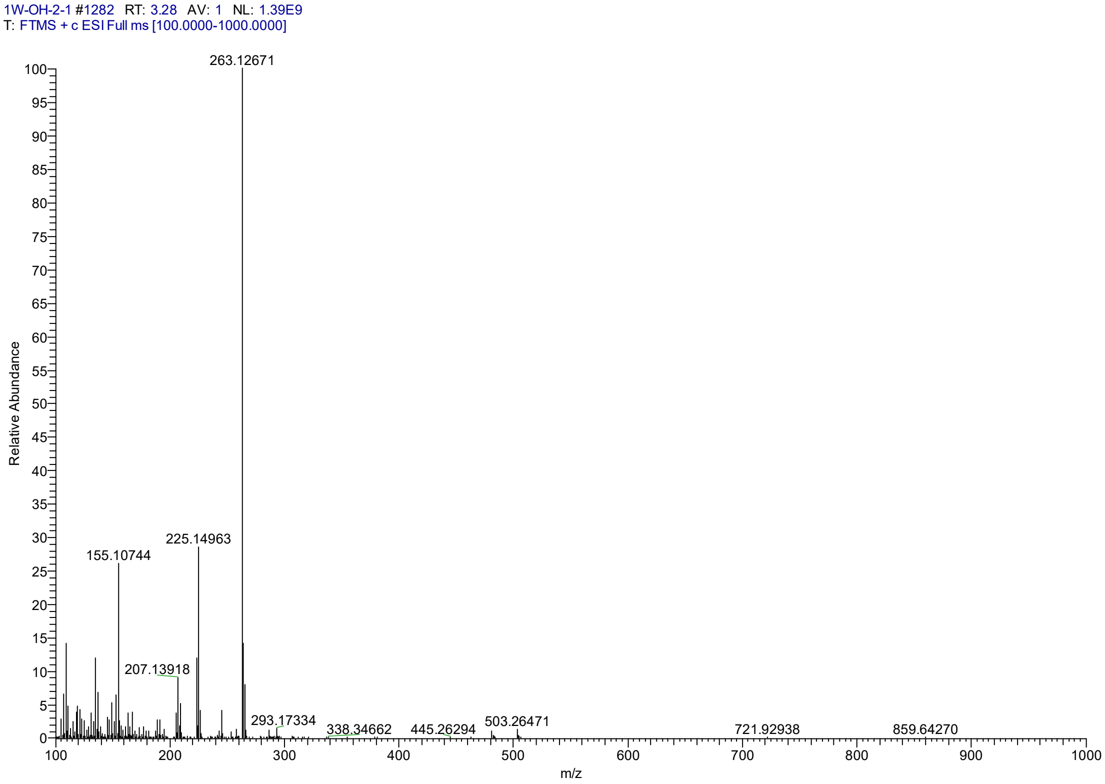

Fig. S69 HRMS data of compound VI-1a

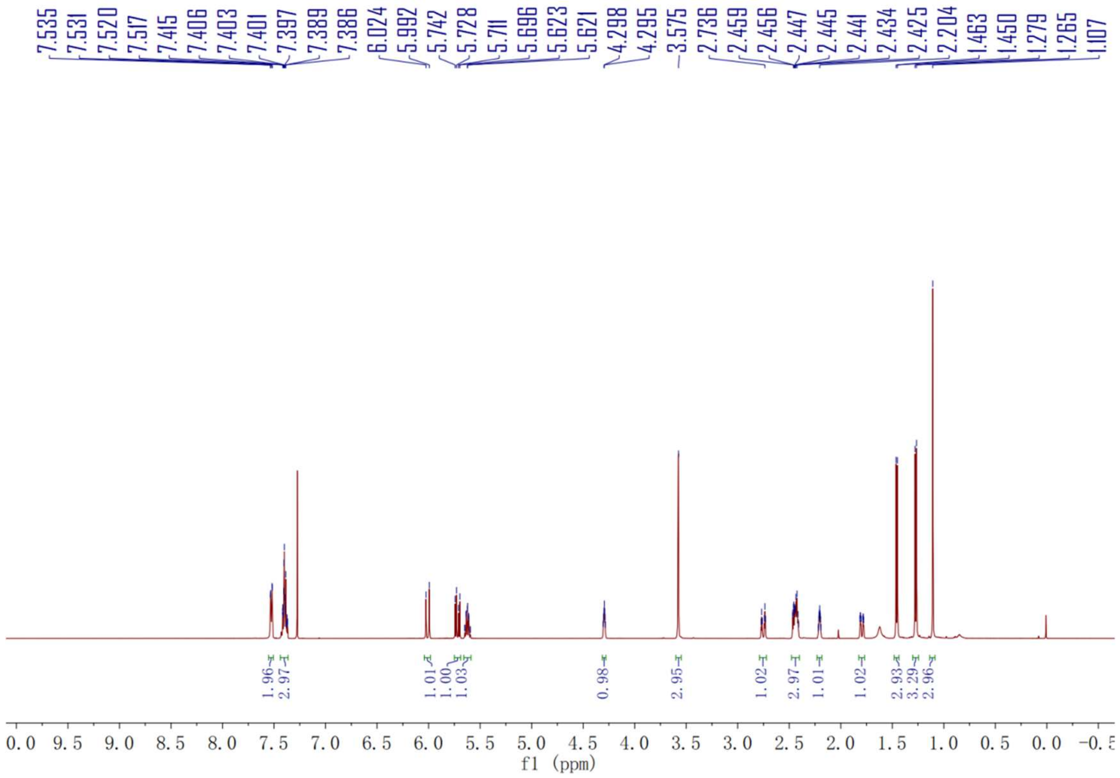

Fig. S70 ^{1}H NMR spectrum of VI-1a (*R*-MTPA ester) in $CDCl_3$ (500 MHz)

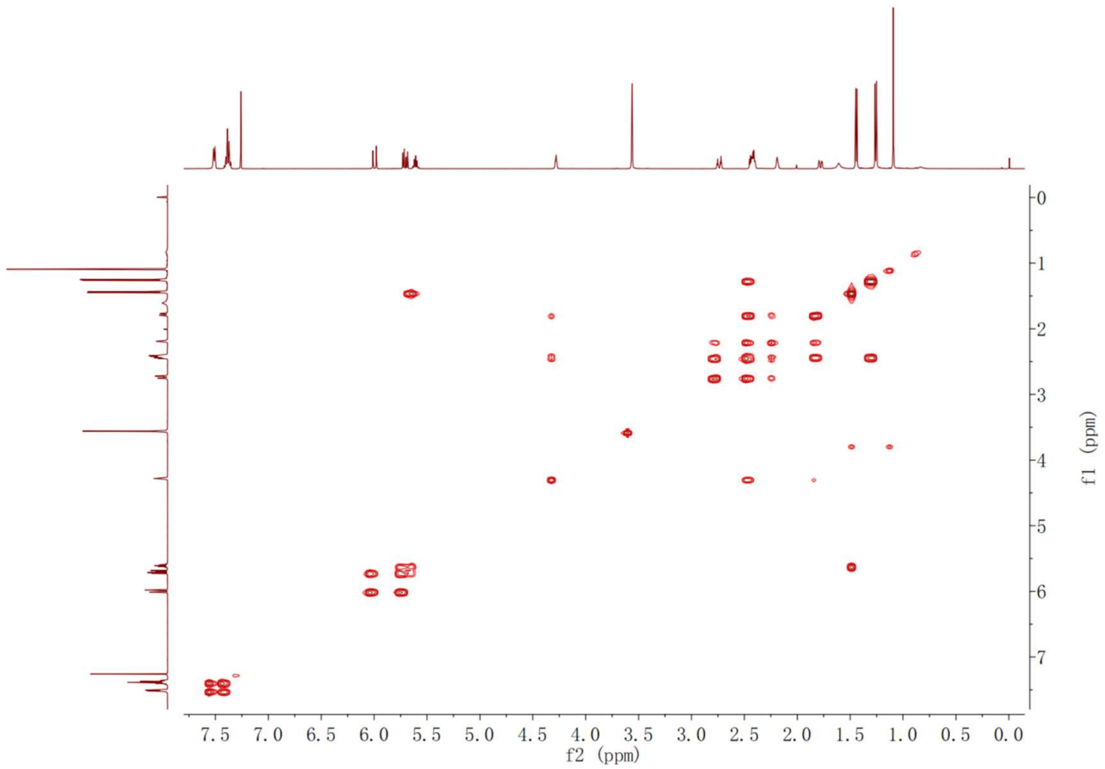

Fig. S71 ^{1}H-^{1}H COSY spectrum of VI-1a (R-MTPA ester) in $CDCl_3$ (500 MHz)

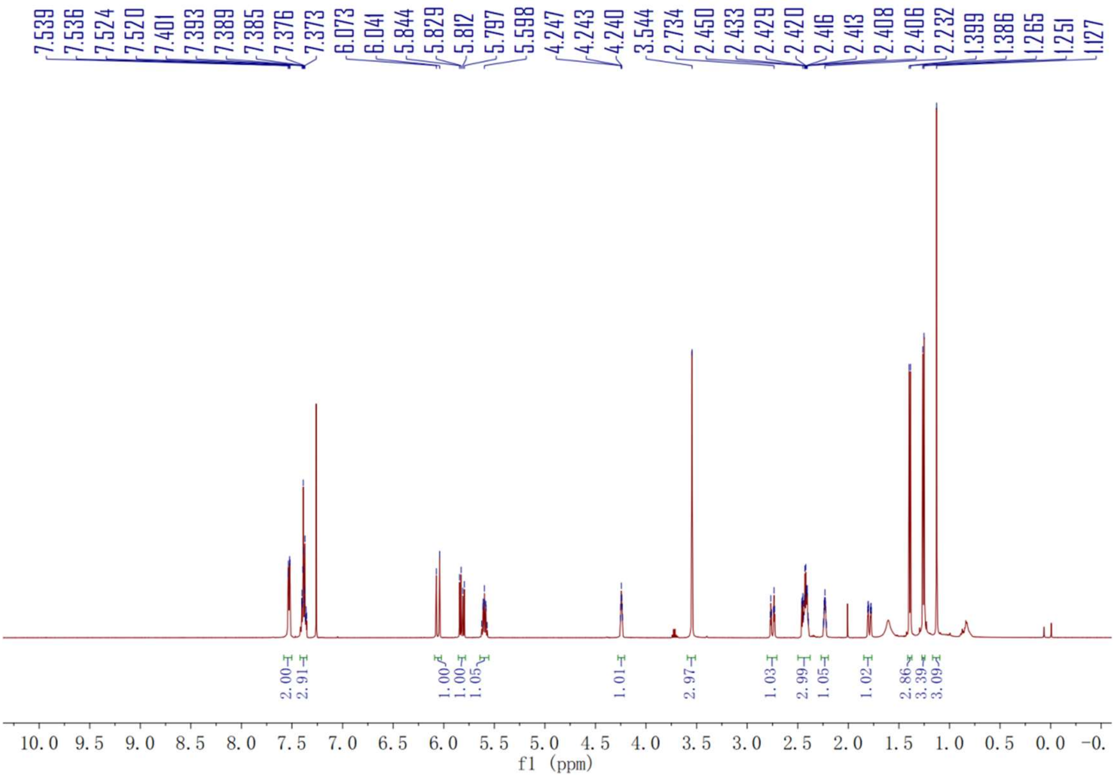

Fig. S72 ^{1}H NMR spectrum of VI-1a (*S*-MTPA ester) in $CDCl_3$ (500 MHz)

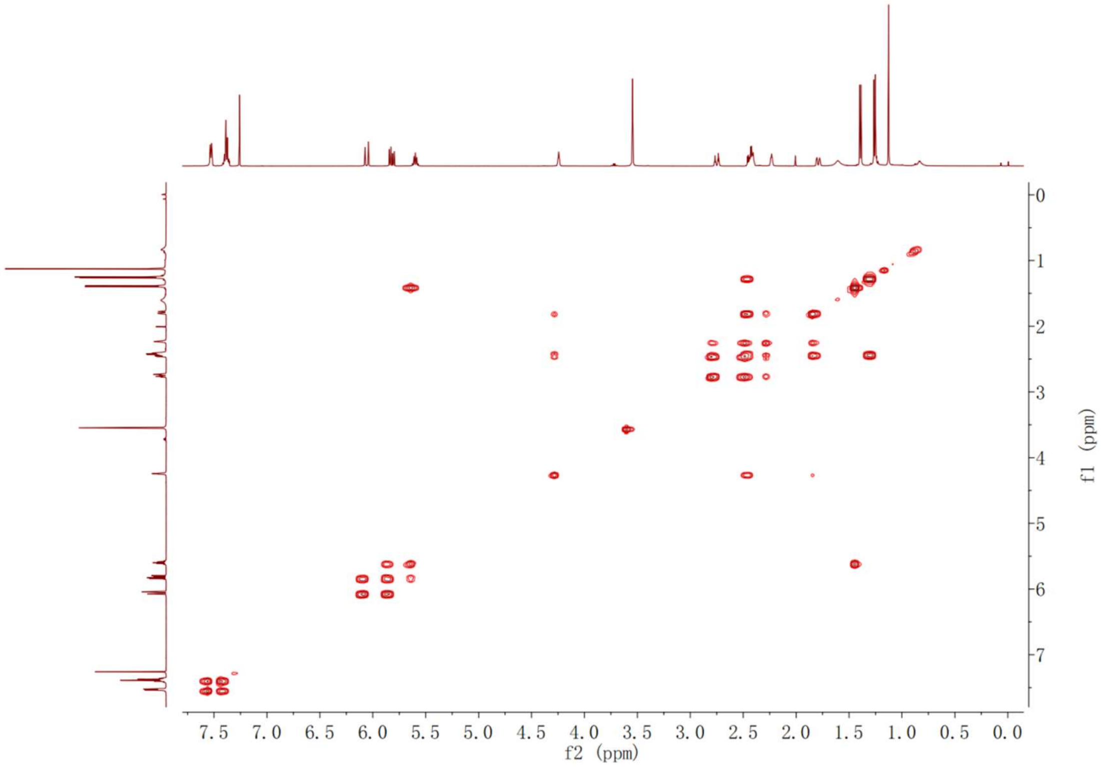

Fig. S73 ^{1}H-^{1}H COSY spectrum of VI-1a (S-MTPA ester) in $CDCl_3$ (500 MHz)

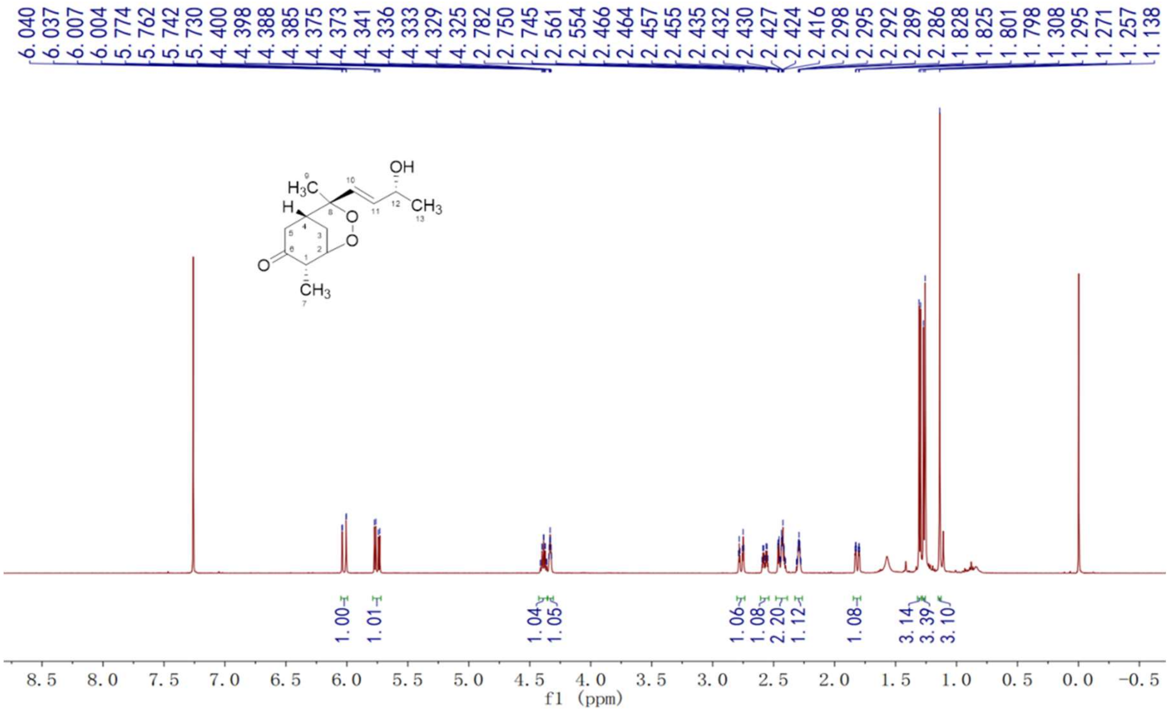

Fig. S74 ^{1}H NMR spectrum of compound VI-1b in $CDCl_3$ (500 MHz)

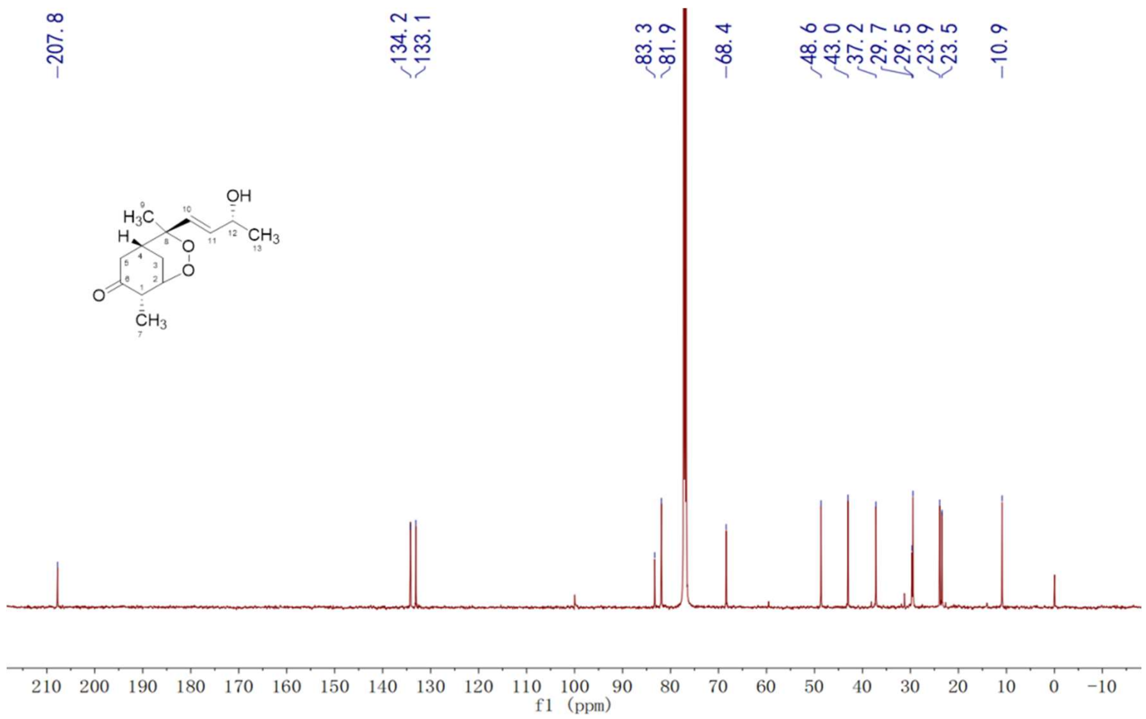

Fig. S75 ^{13}C NMR spectrum of compound VI-1b in $CDCl_3$ (125 MHz)

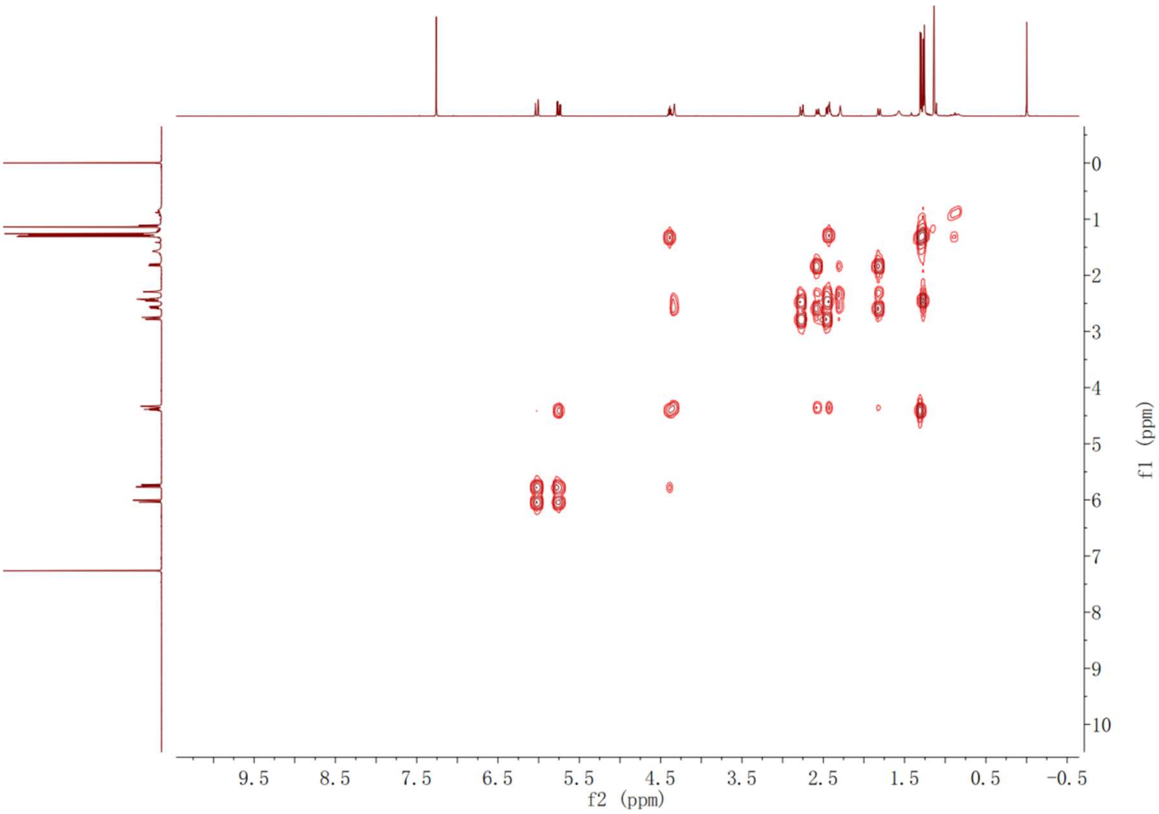

Fig. S76 ^{1}H-^{1}H COSY spectrum of compound VI-1b in $CDCl_3$ (500 MHz)

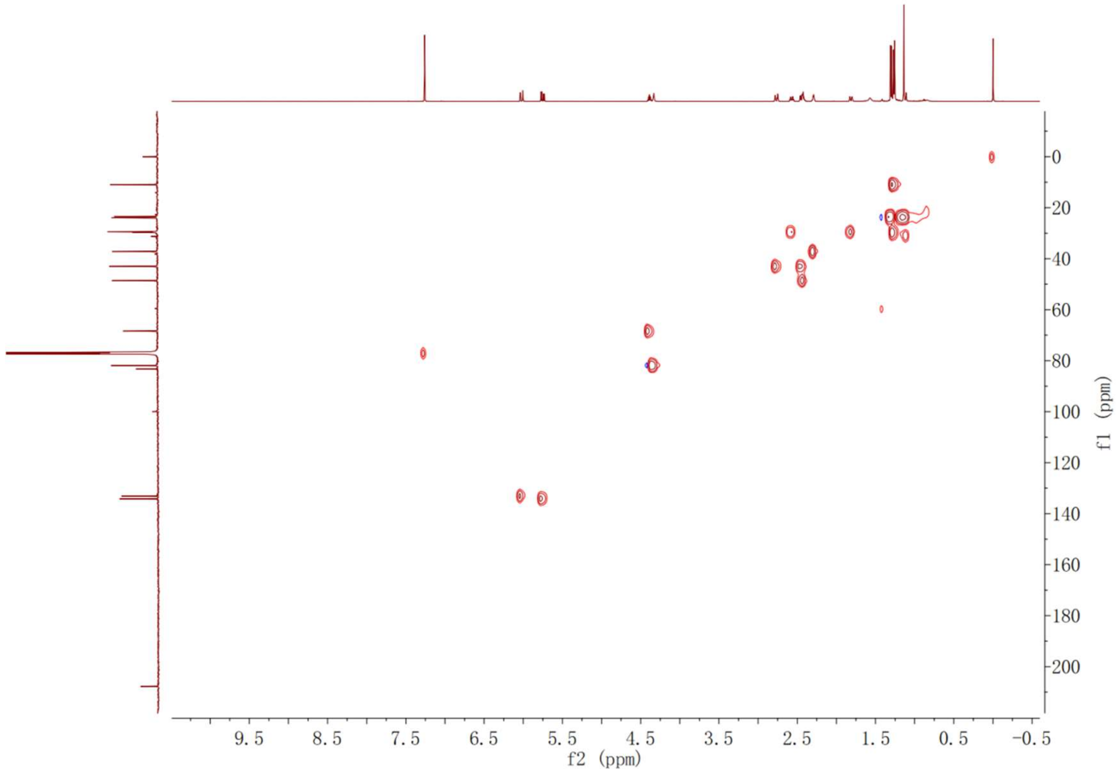

Fig. S77 HSQC spectrum of compound VI-1b in $CDCl_3$ (500 MHz)

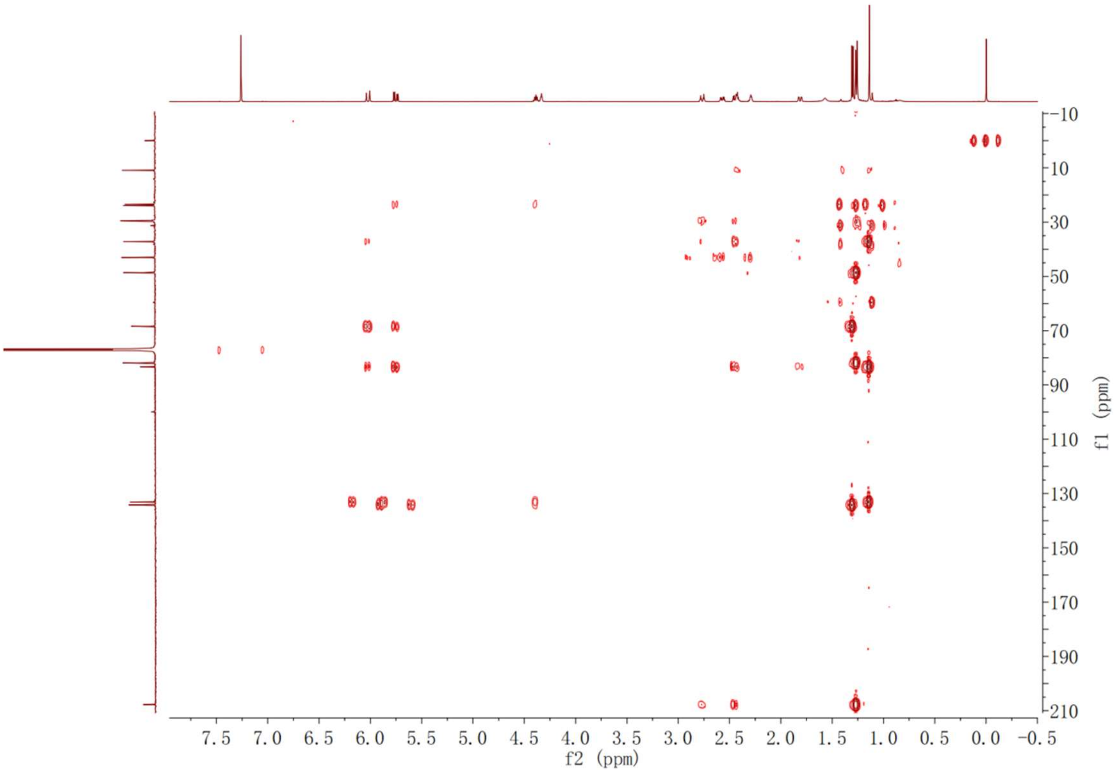

Fig. S78 HMBC spectrum of compound VI-1b in $CDCl_3$ (500 MHz)

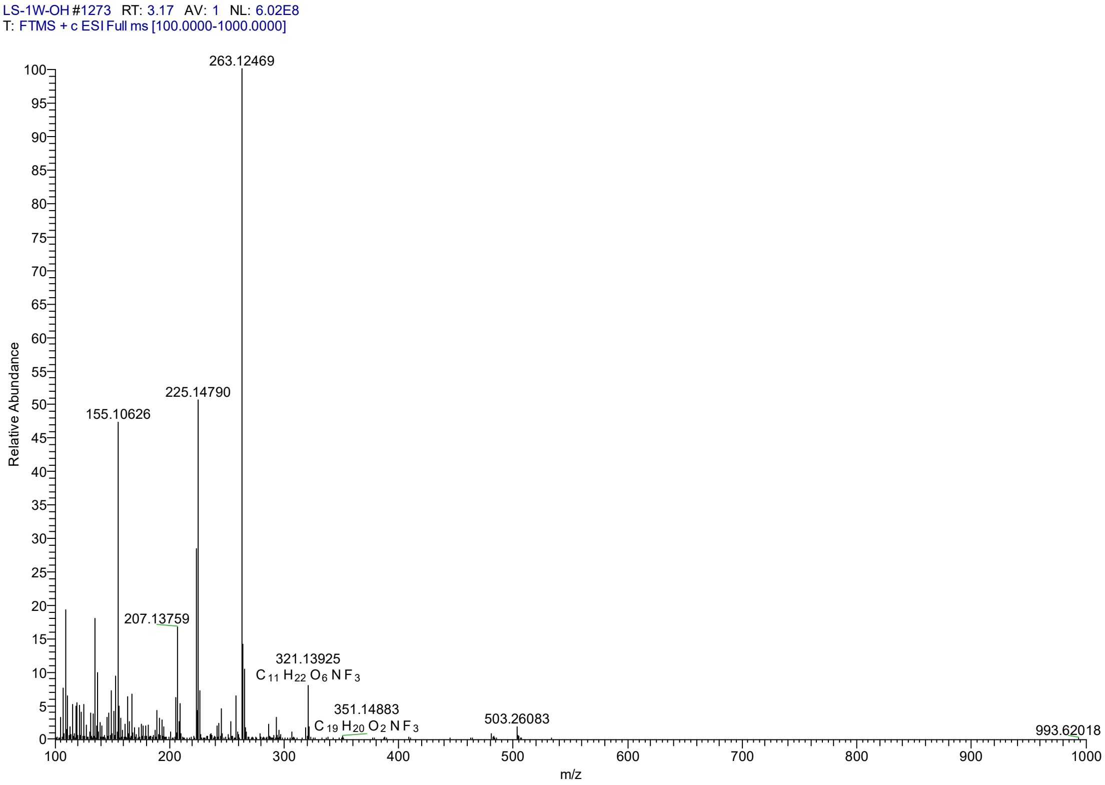

Fig. S79 HRMS data of compound VI-1b

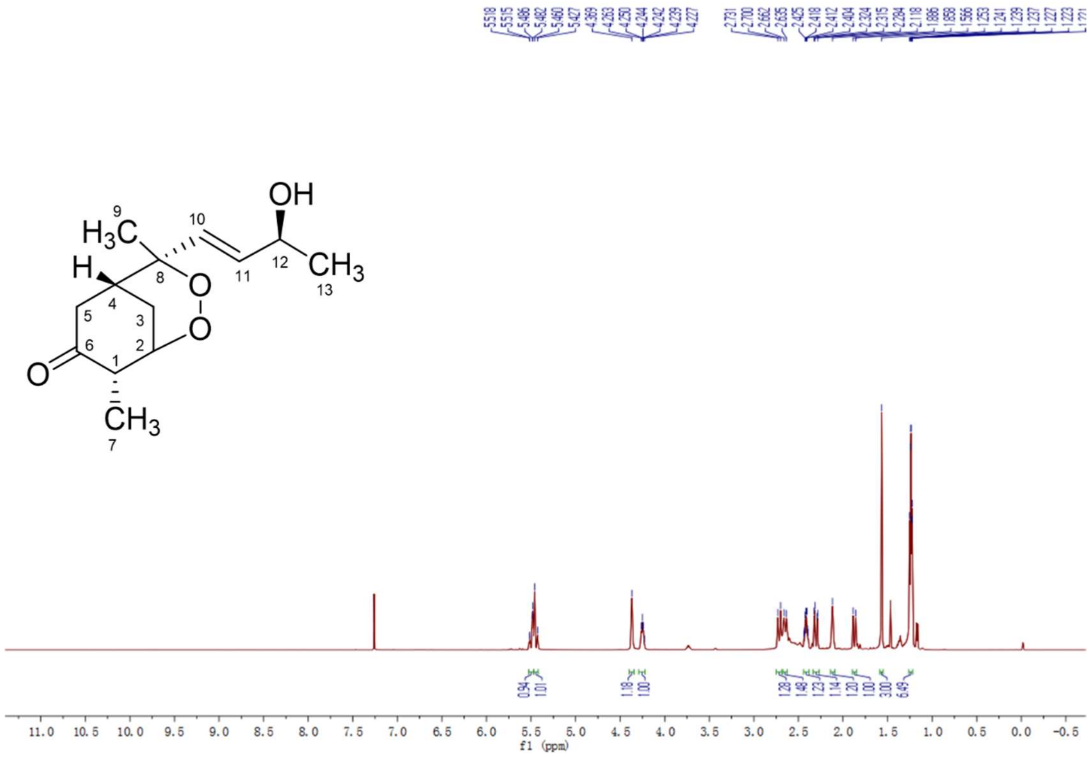

Fig. S80 ^{1}H NMR spectrum of compound VI-2a in $CDCl_3$ (500 MHz)

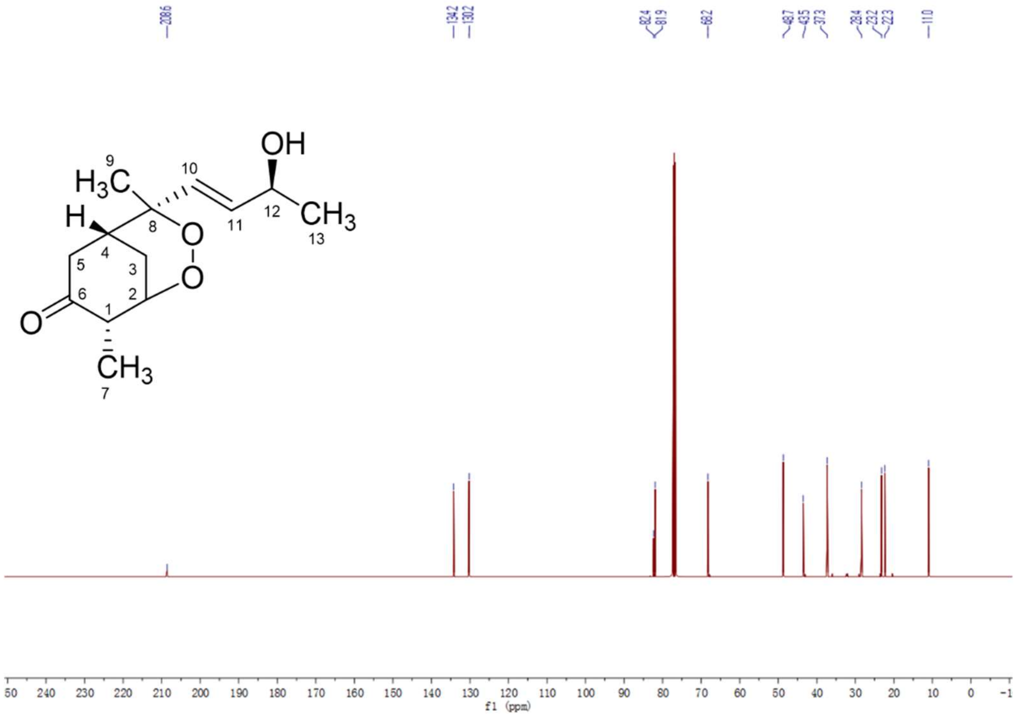

Fig. S81 ^{13}C NMR spectrum of compound VI-2a in $CDCl_3$ (125 MHz)

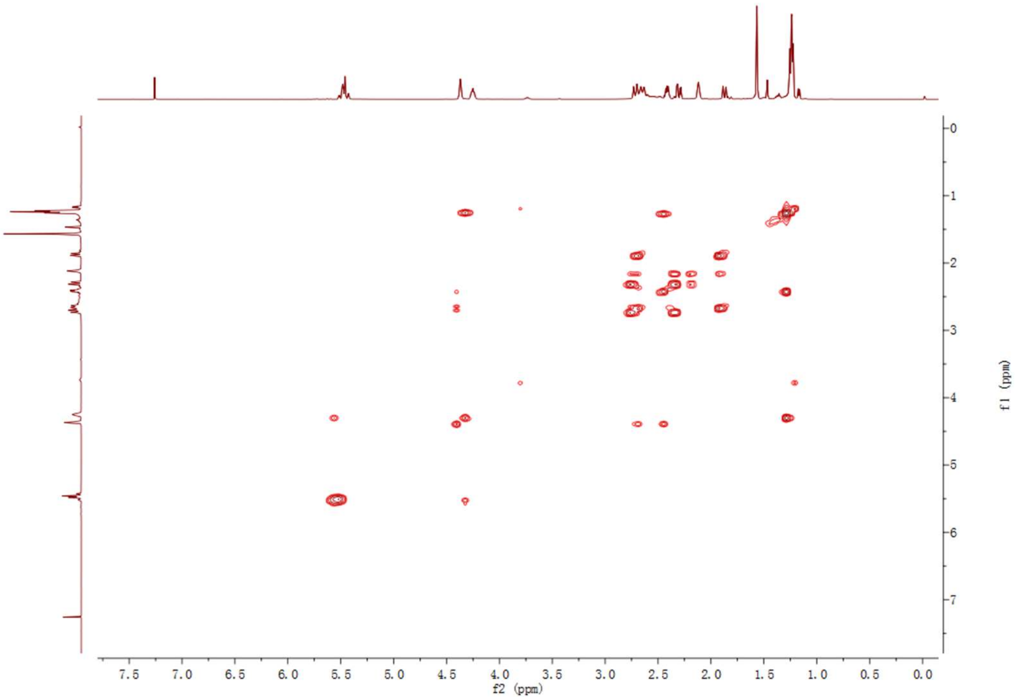

Fig. S82 ¹H-¹H COSY spectrum of compound VI-2a in $CDCl_3$ (500 MHz)

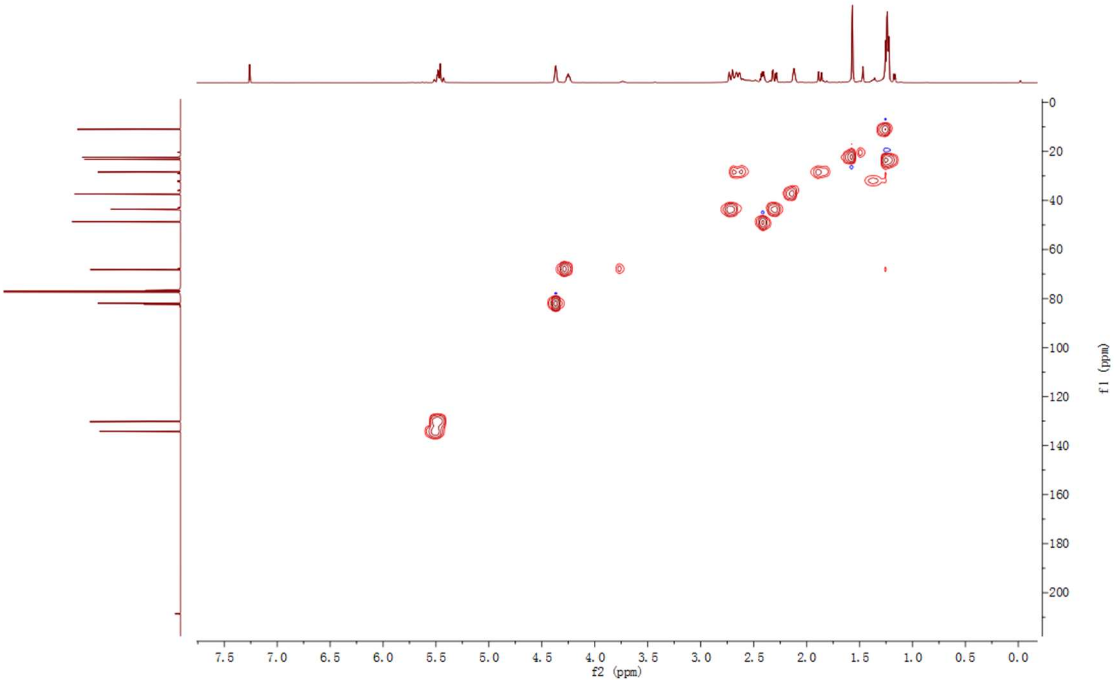

Fig. S83 HSQC spectrum of compound VI-2a in $CDCl_3$ (500 MHz)

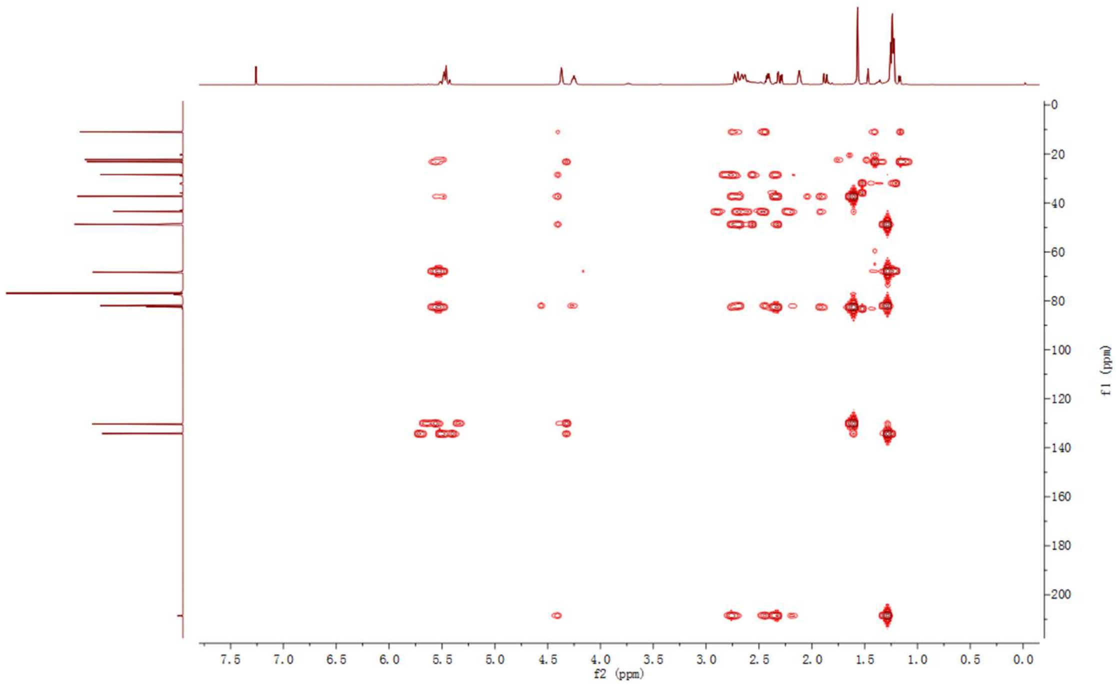

Fig. S84 HMBC spectrum of compound VI-2a in CDCl3 (500 MHz)

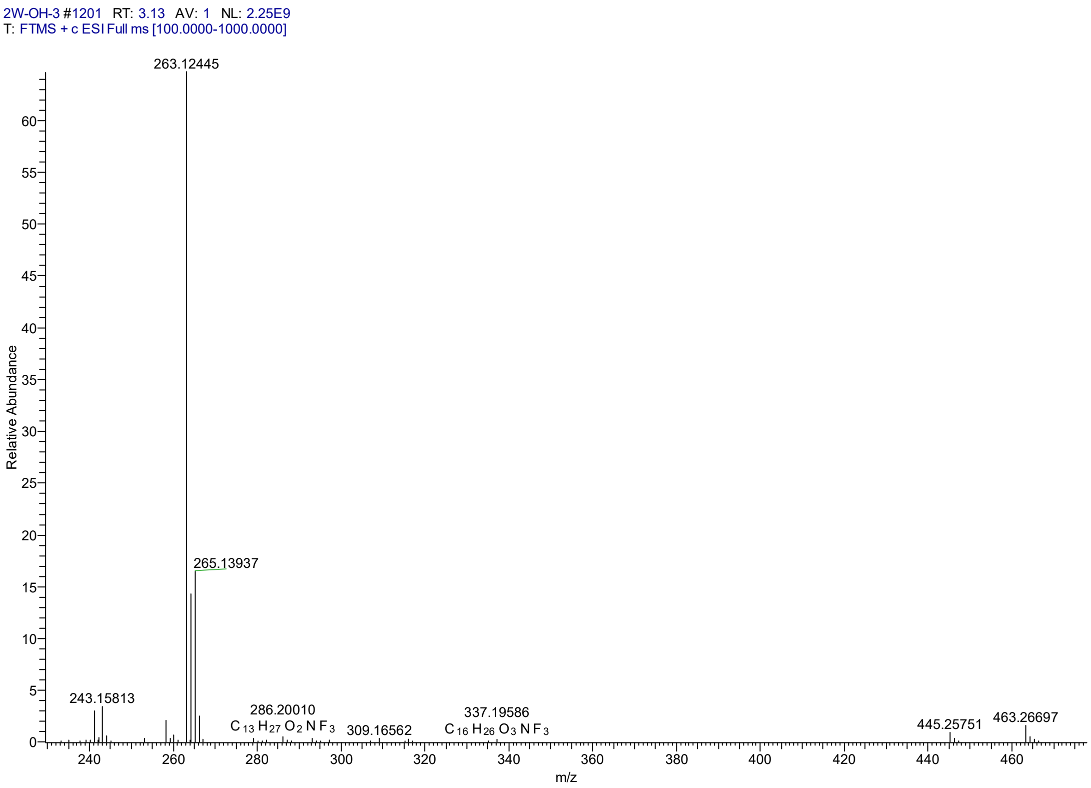

Fig. S85 HRMS data of compound VI-2a

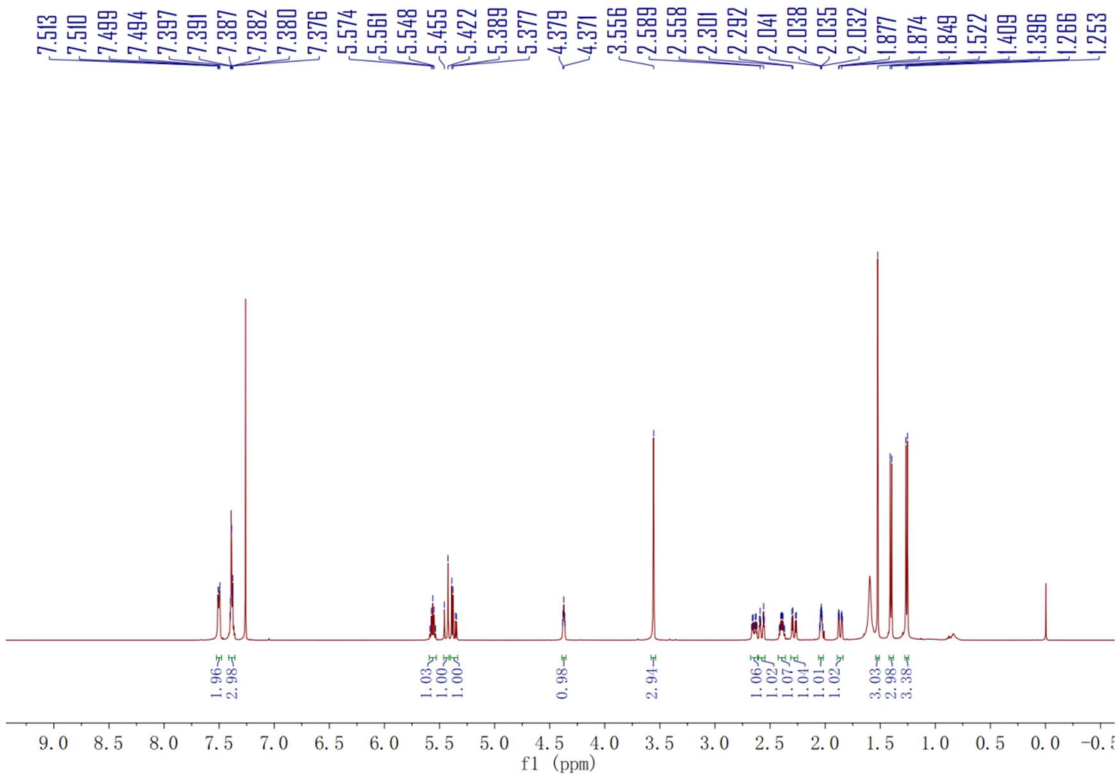

Fig. S86 ^{1}H NMR spectrum of VI-2a (*R*-MTPA ester) in $CDCl_3$ (500 MHz)

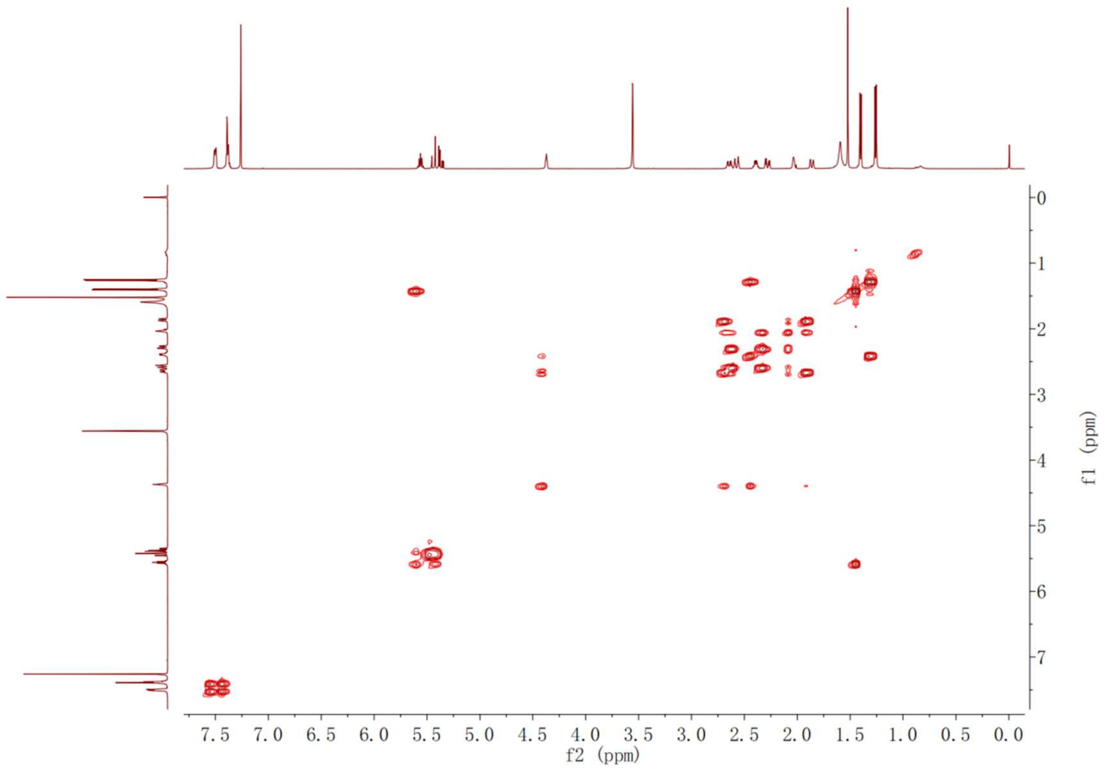

Fig. S87 ^{1}H-^{1}H COSY spectrum of VI-2a (R-MTPA ester) in $CDCl_3$ (500 MHz)

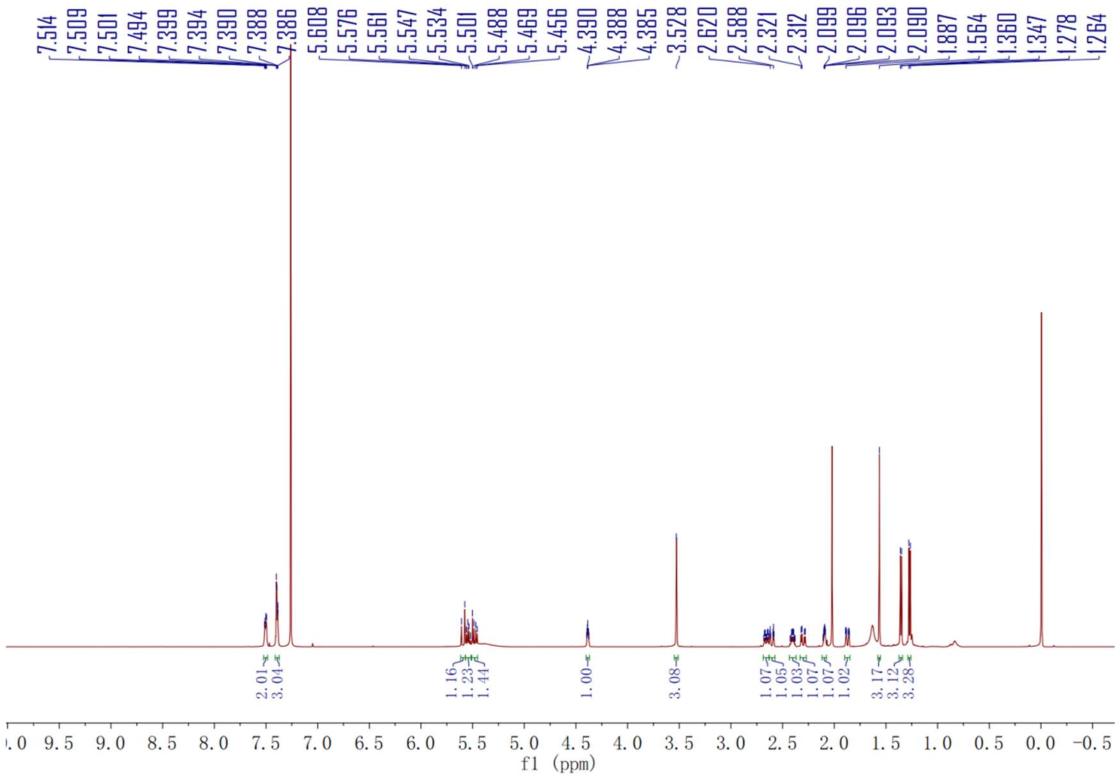

Fig. S88 ¹H NMR spectrum of VI-2a (*S*-MTPA ester) in $CDCl_3$ (500 MHz)

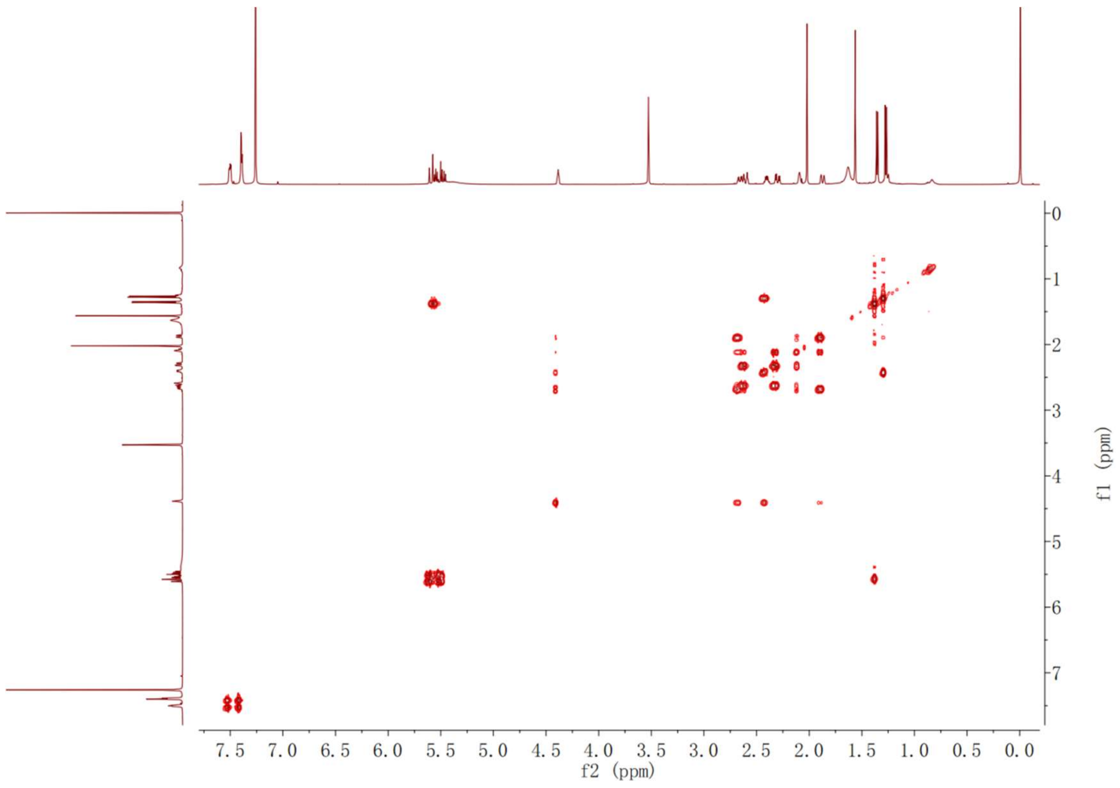

Fig. S89 ¹H-¹H COSY spectrum of VI-2a (S-MTPA ester) in $CDCl_3$ (500 MHz)

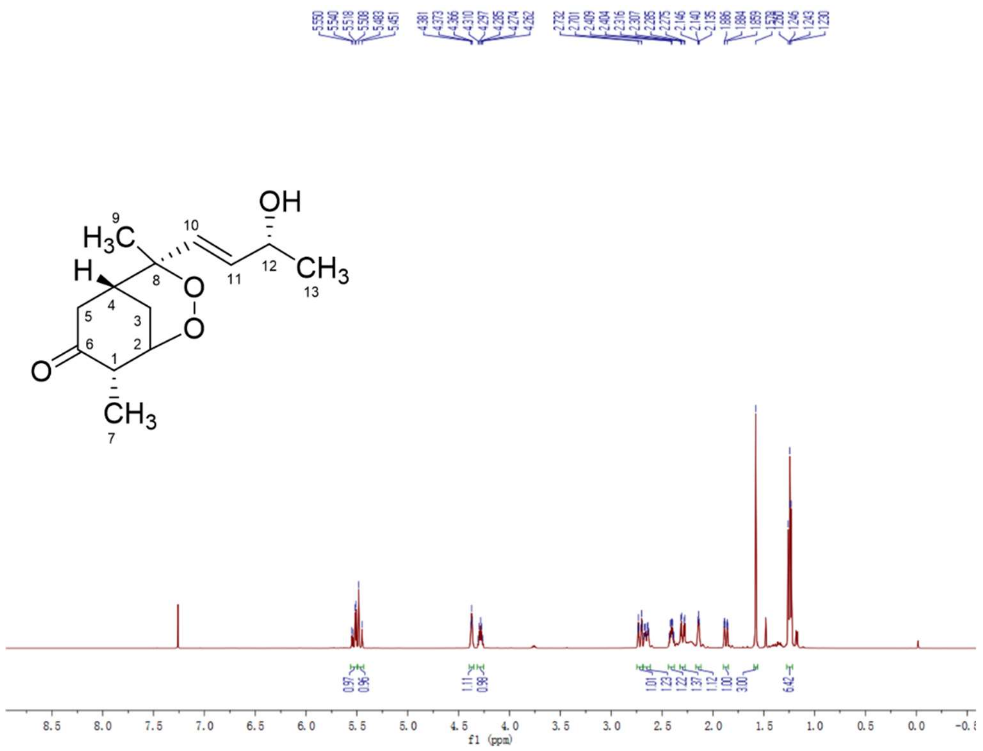

Fig. S90 ¹H NMR spectrum of compound VI-2b in CDCl₃ (500 MHz)

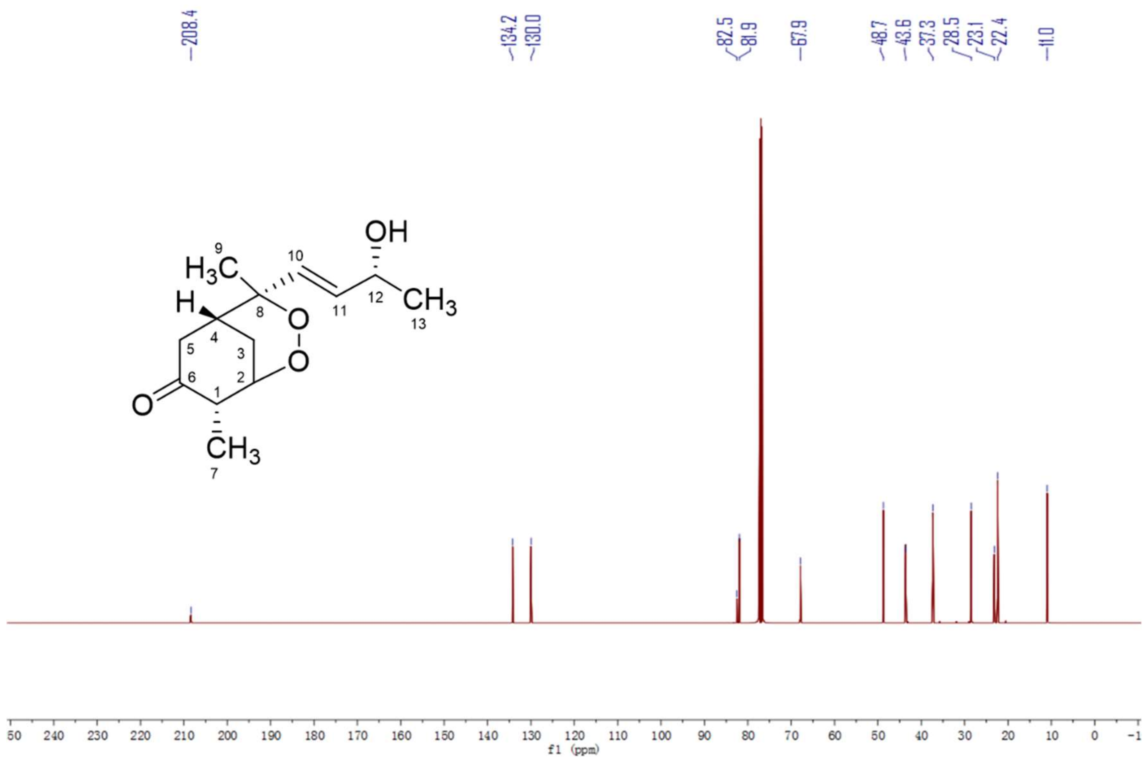

Fig. S91 ¹³C NMR spectrum of compound VI-2b in CDCl₃ (125 MHz)

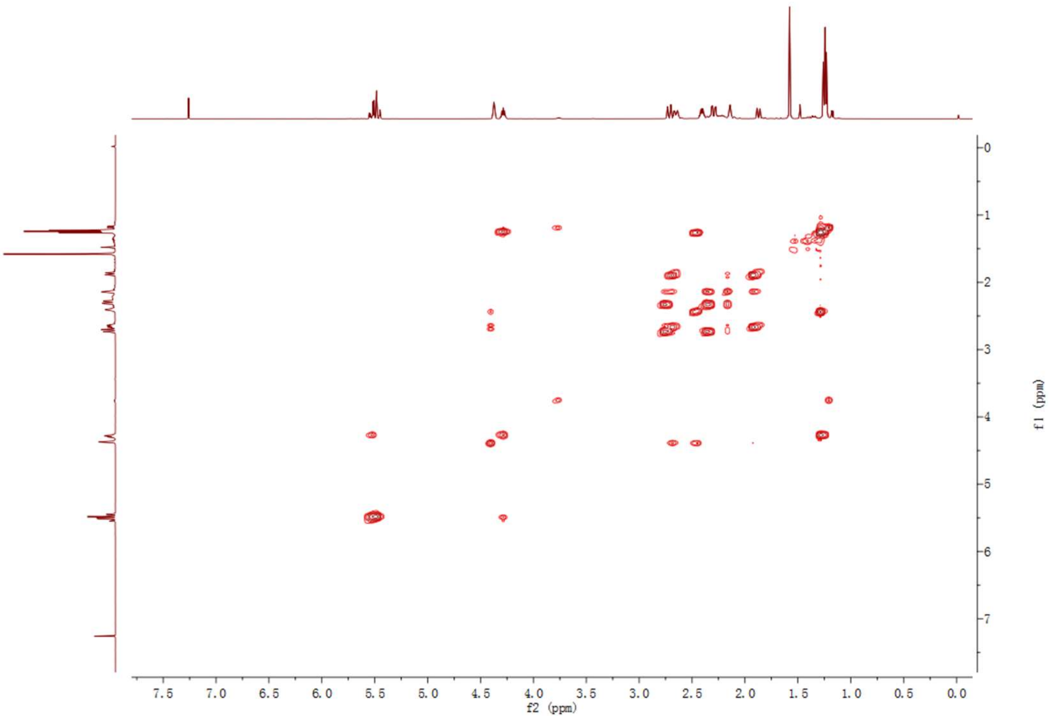

Fig. S92 ^{1}H-^{1}H COSY spectrum of compound VI-2b in $CDCl_3$ (500 MHz)

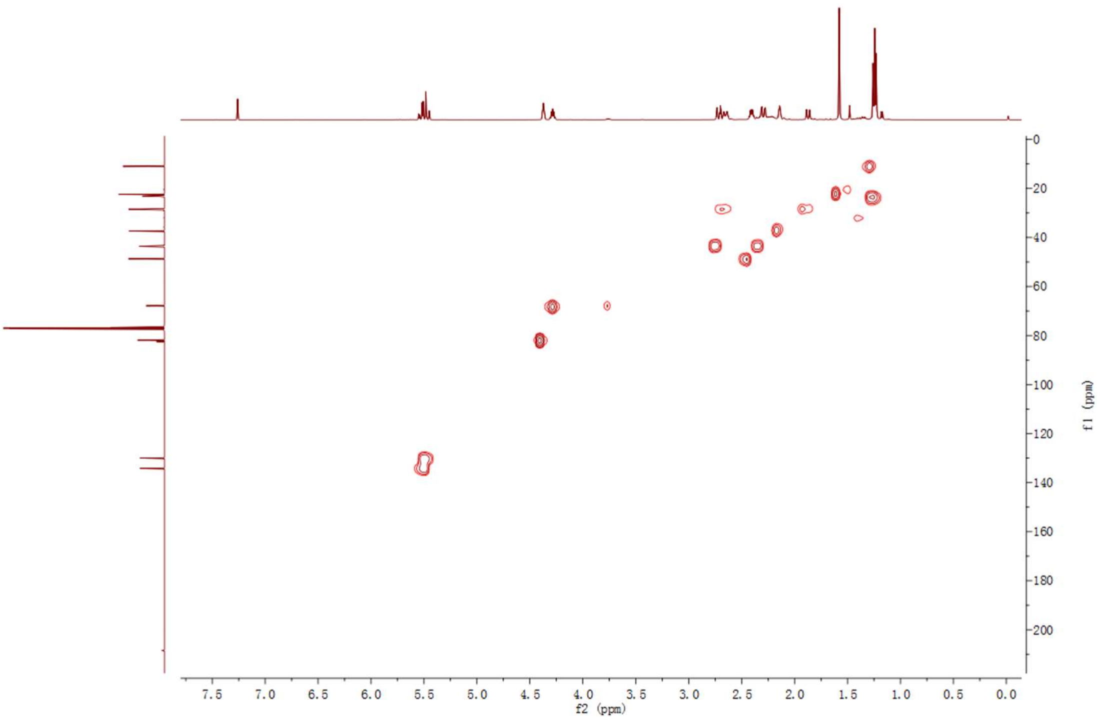

Fig. S93 HSQC spectrum of compound VI-2b in $CDCl_3$ (500 MHz)

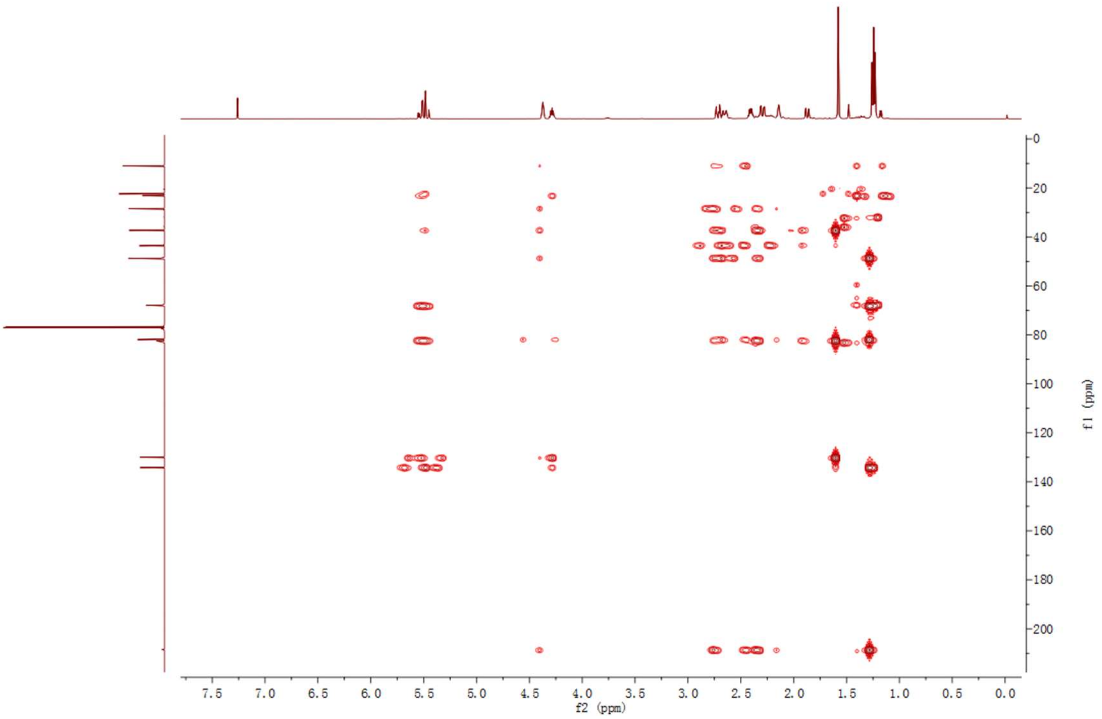

Fig. S94 HMBC spectrum of compound VI-2b in $CDCl_3$ (500 MHz)

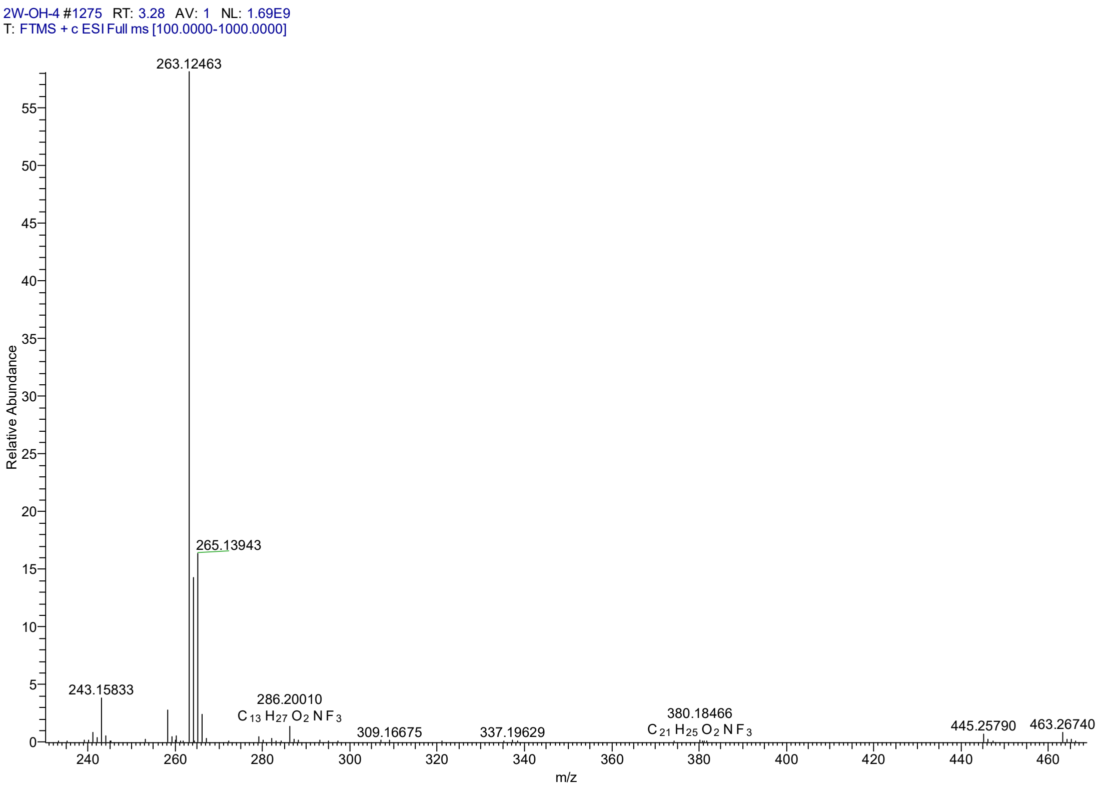

Fig. S95 HRMS data of compound VI-2b

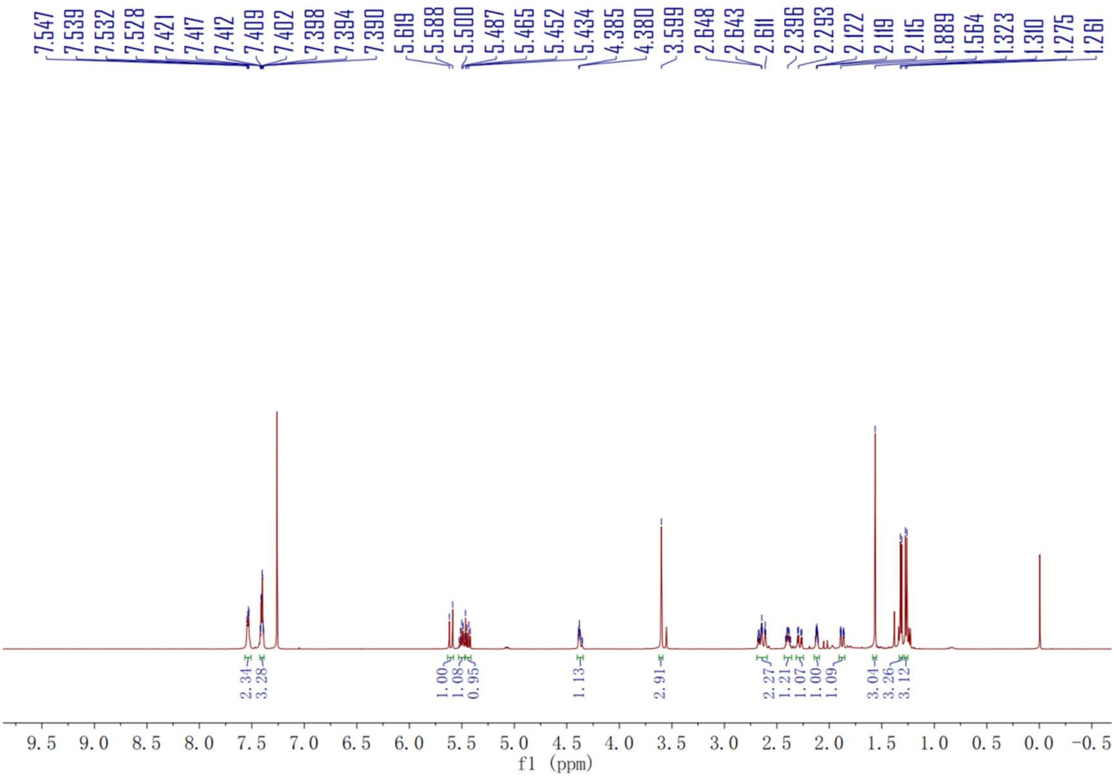

Fig. S96 ^{1}H NMR spectrum of VI-2b (*R*-MTPA ester) in $CDCl_3$ (500 MHz)

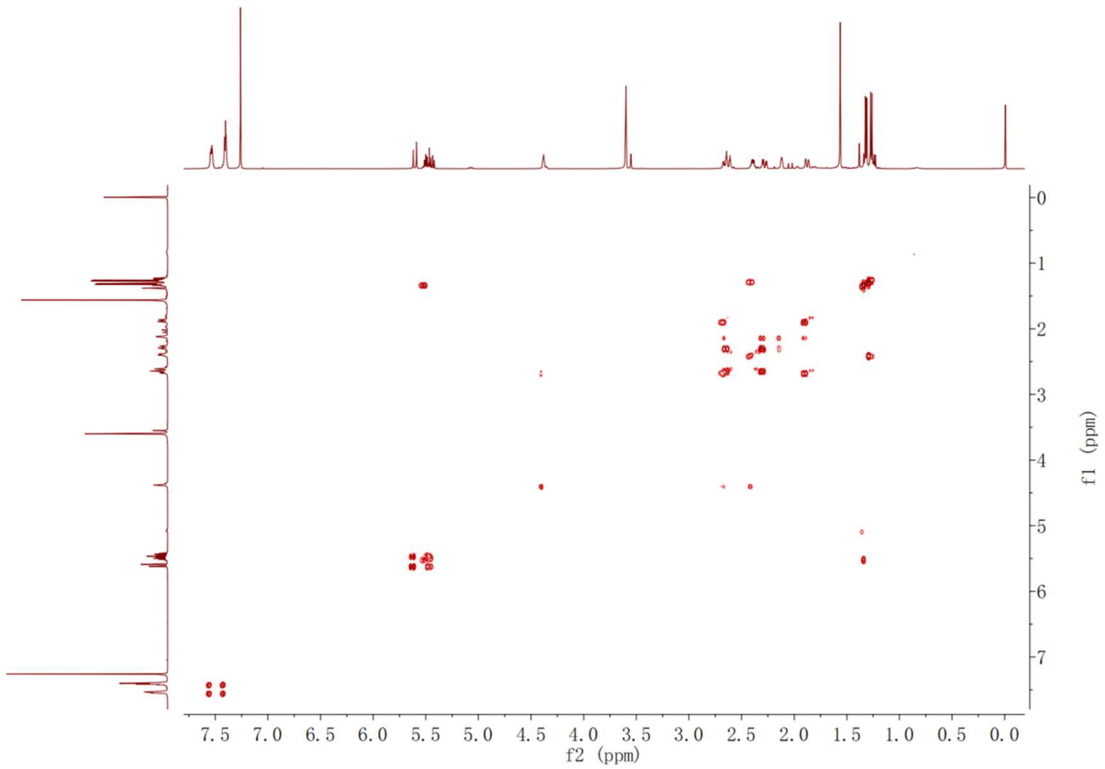

Fig. S97 ^{1}H-^{1}H COSY spectrum of VI-2b (*R*-MTPA ester) in $CDCl_3$ (500 MHz)

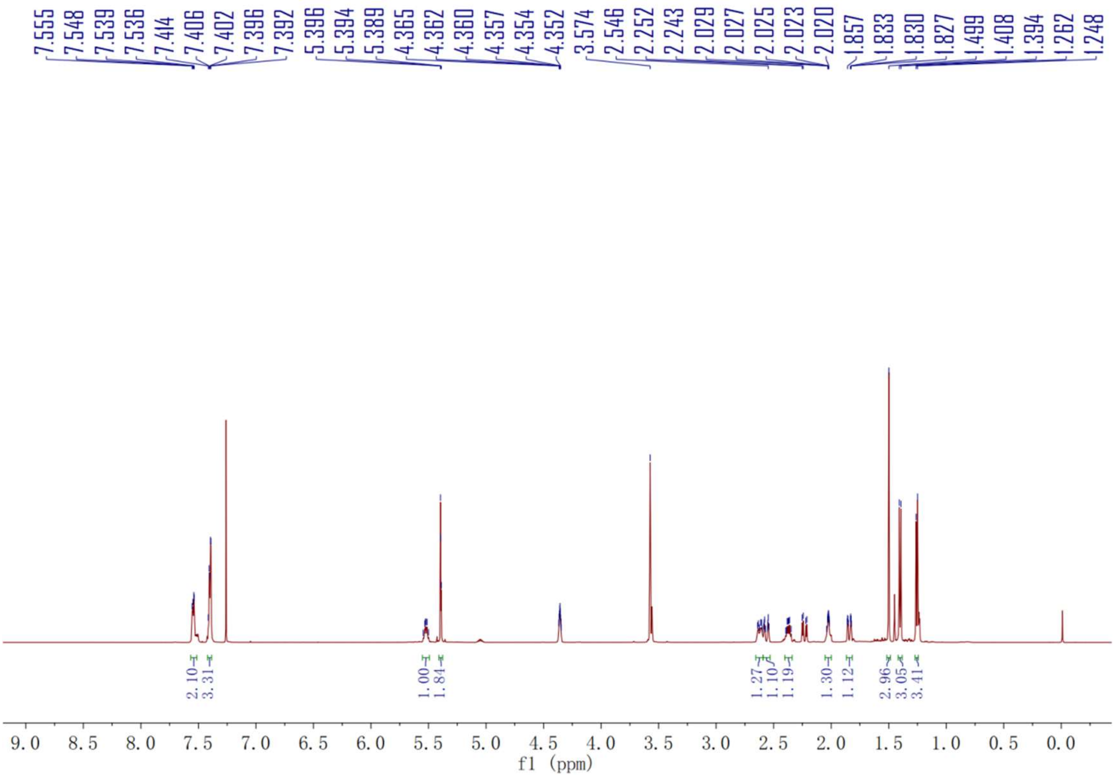

Fig. S98 ^{1}H NMR spectrum of VI-2b (*S*-MTPA ester) in $CDCl_3$ (500 MHz)

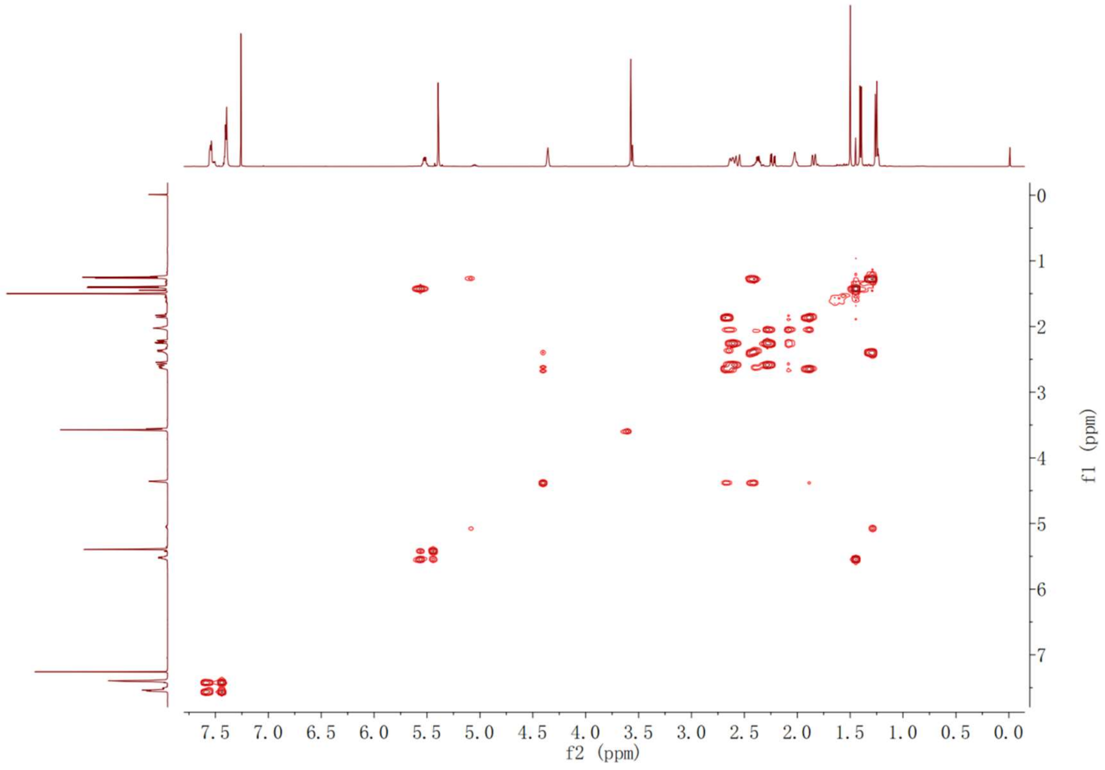

Fig. S99 ^{1}H-^{1}H COSY spectrum of VI-2b (*S*-MTPA ester) in $CDCl_3$ (500 MHz)

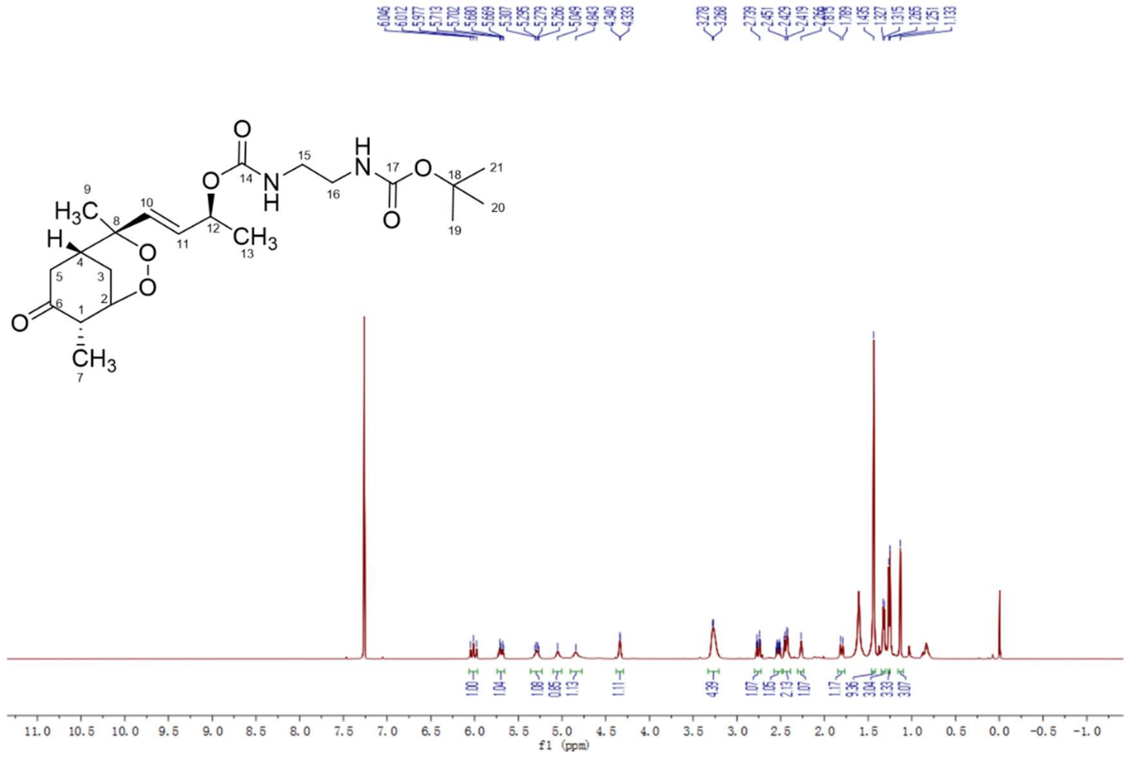

Fig. S100 ^{1}H NMR spectrum of compound VII-1a in $CDCl_3$ (500 MHz)

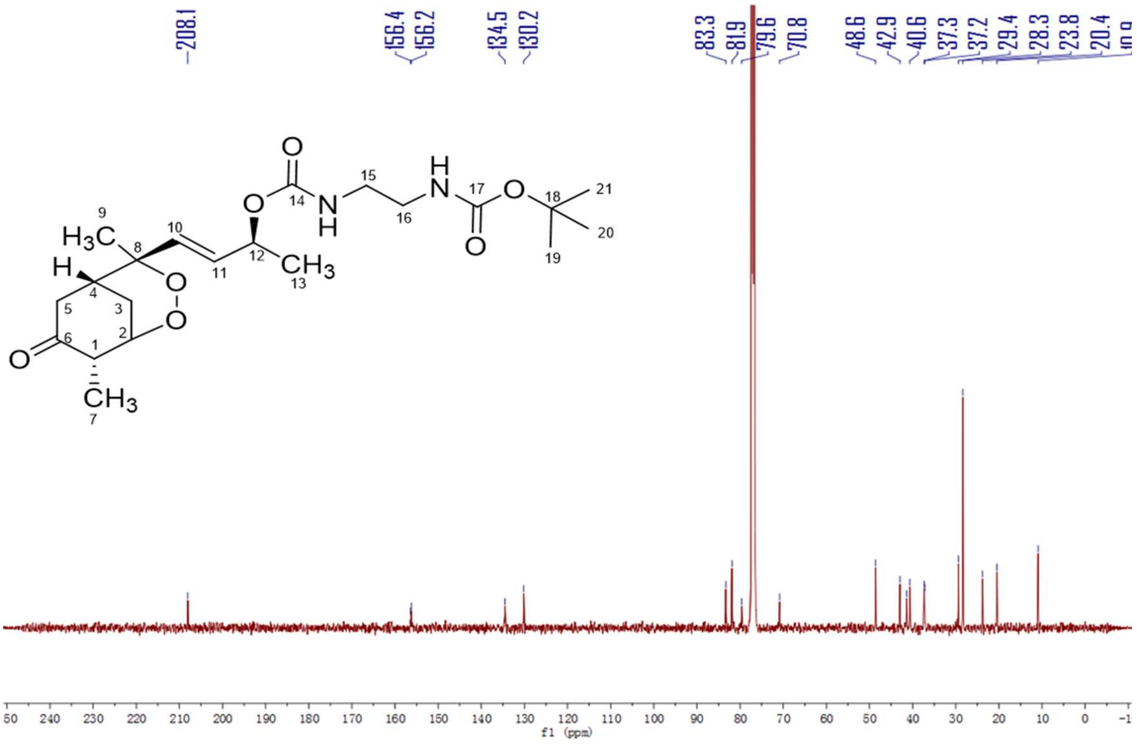

Fig. S101 ^{13}C NMR spectrum of compound VII-1a in $CDCl_3$ (125 MHz)

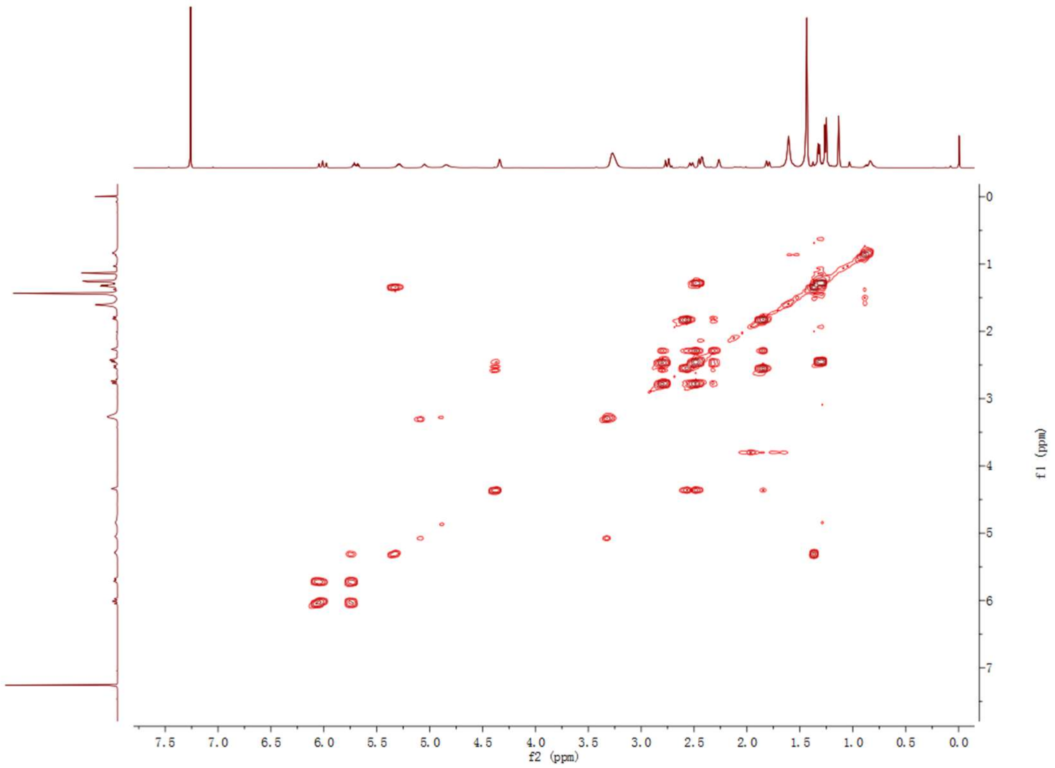

Fig. S102 ^{1}H-^{1}H COSY spectrum of compound VII-1a in $CDCl_3$ (500 MHz)

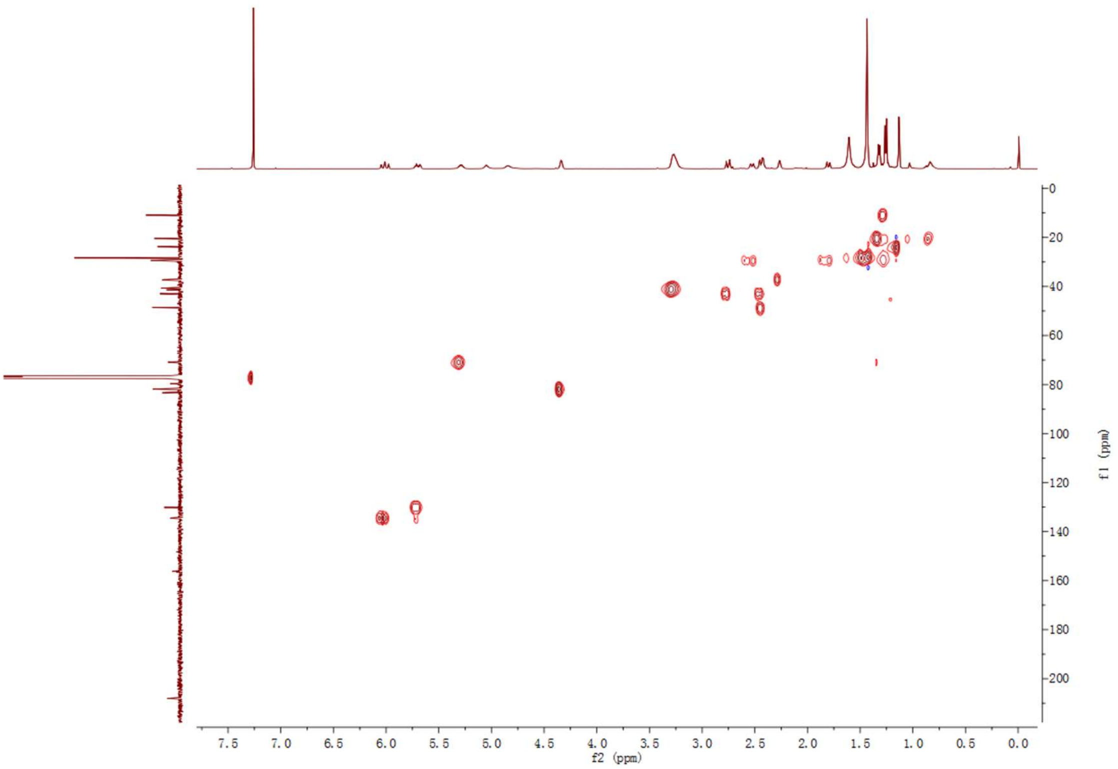

Fig. S103 HSQC spectrum of compound VII-1a in $CDCl_3$ (500 MHz)

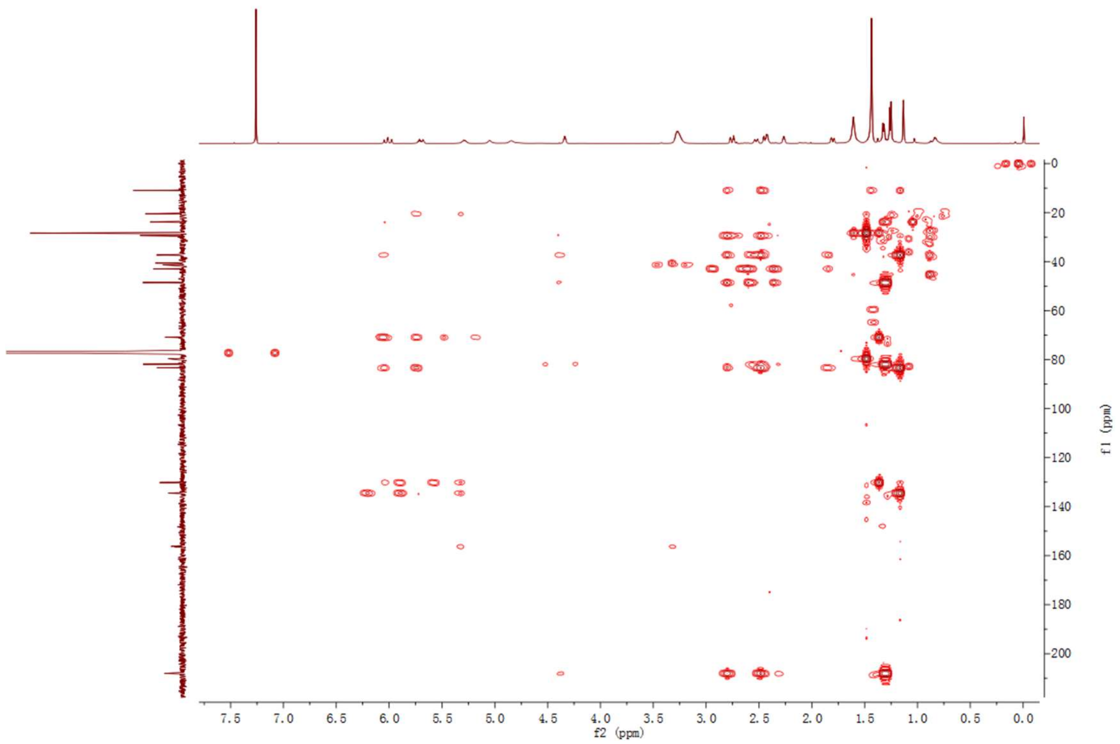

Fig. S104 HMBC spectrum of compound VII-1a in CDCl3 (500 MHz)

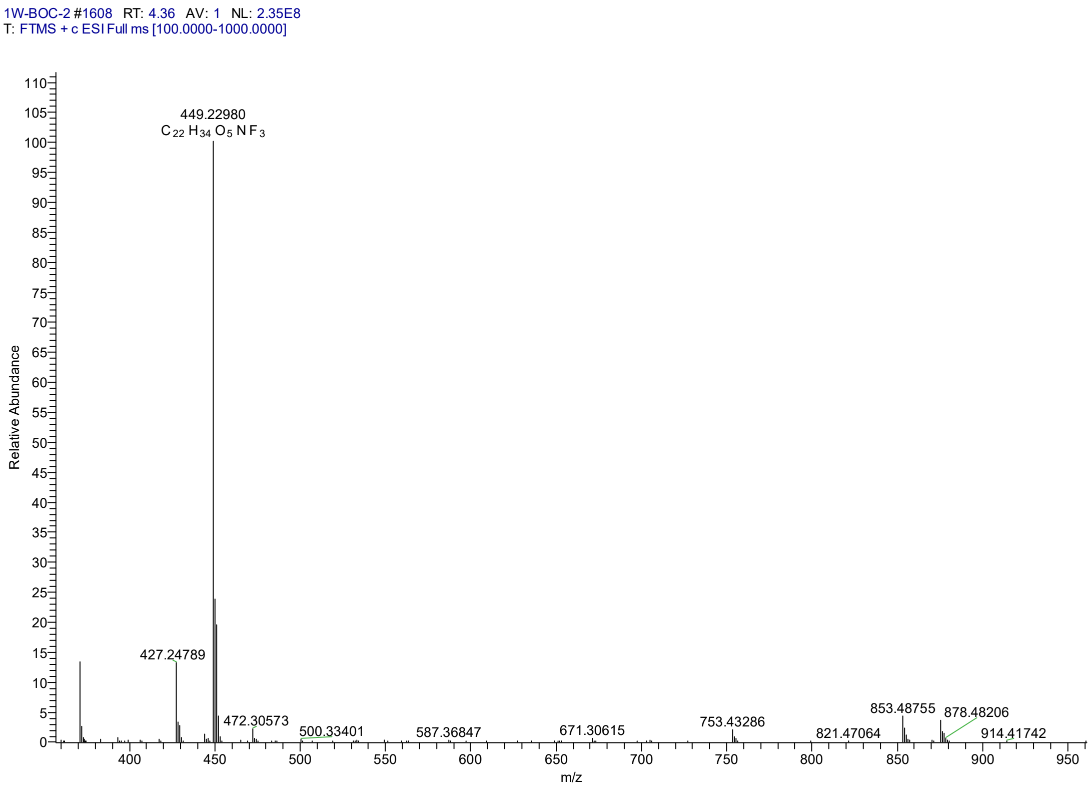

Fig. S105 HRMS data of compound VII-1a

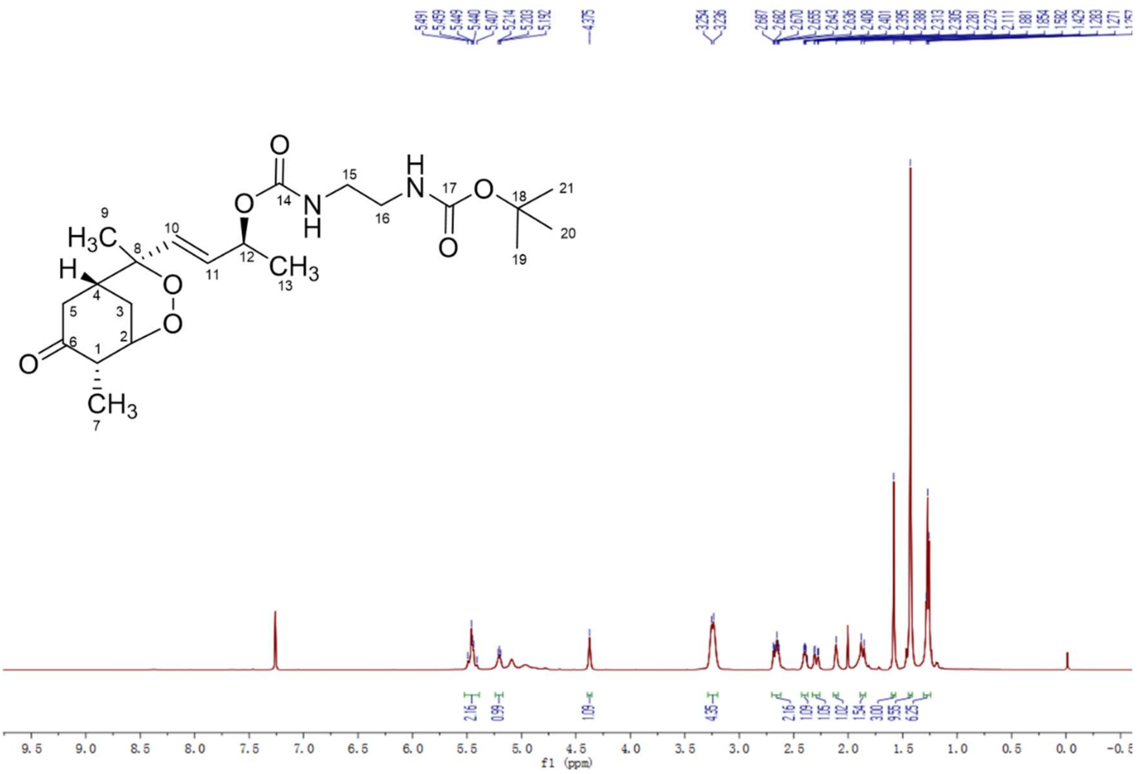

Fig. S106 ^{1}H NMR spectrum of compound VII-2a in $CDCl_3$ (500 MHz)

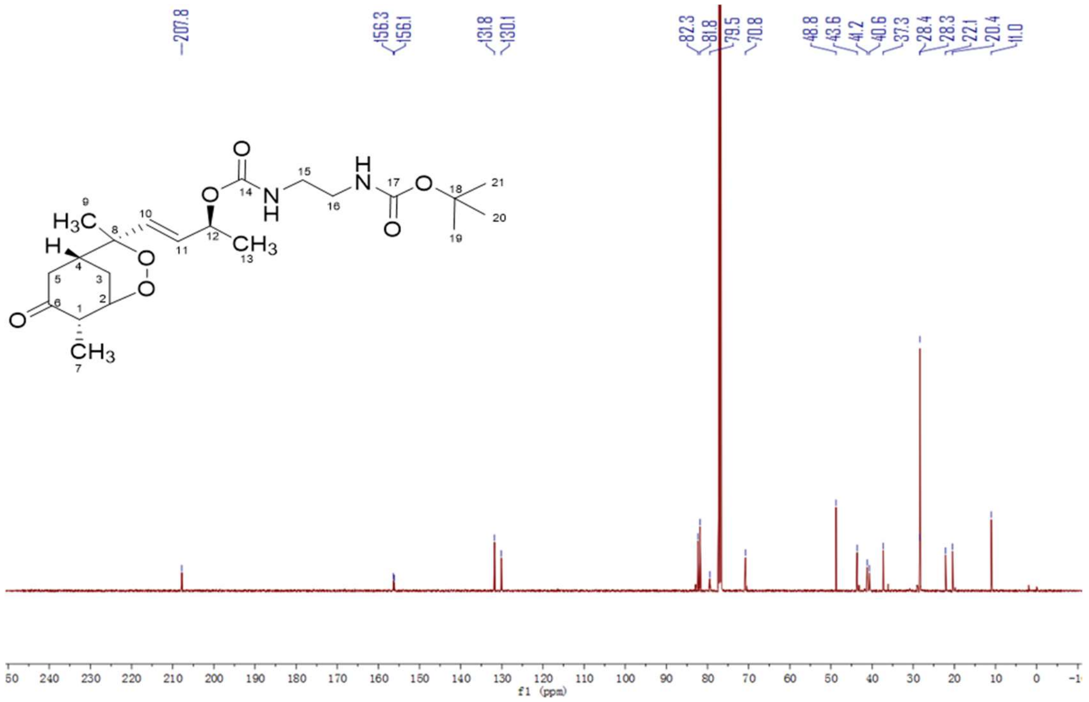

Fig. S107^{13}C NMR spectrum of compound VII-2a in $CDCl_3$ (125 MHz)

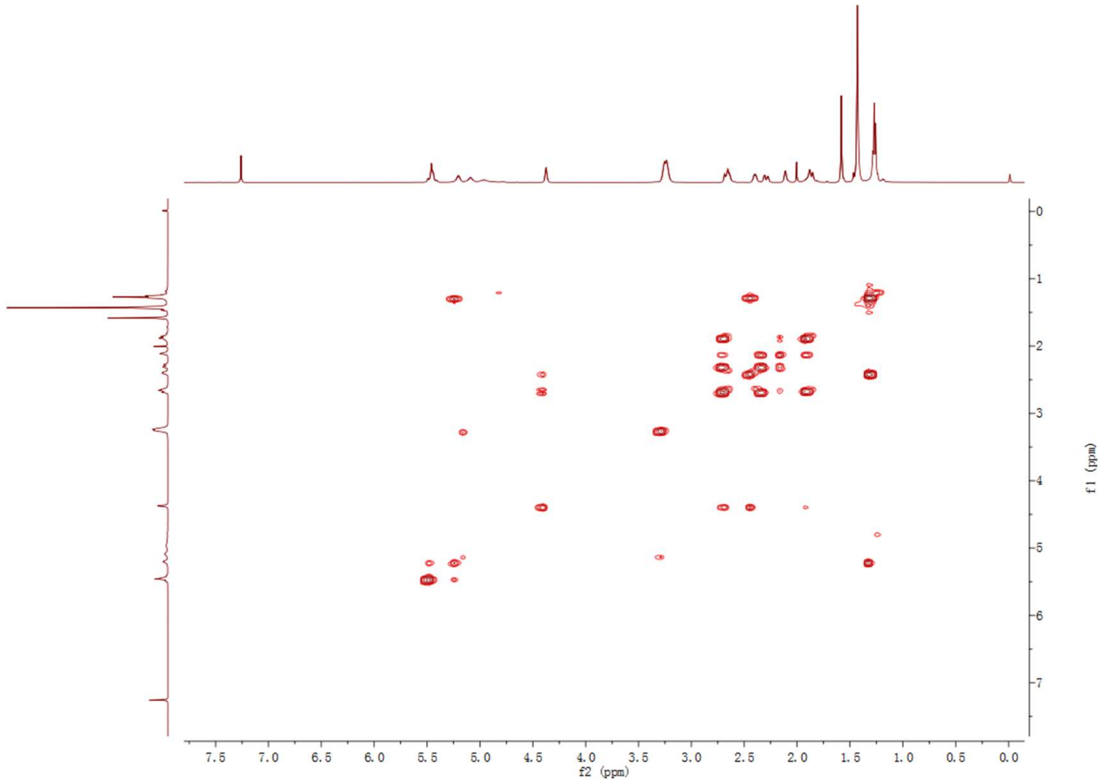

Fig. S108 ^{1}H-^{1}H COSY spectrum of compound VII-2a in $CDCl_3$ (500 MHz)

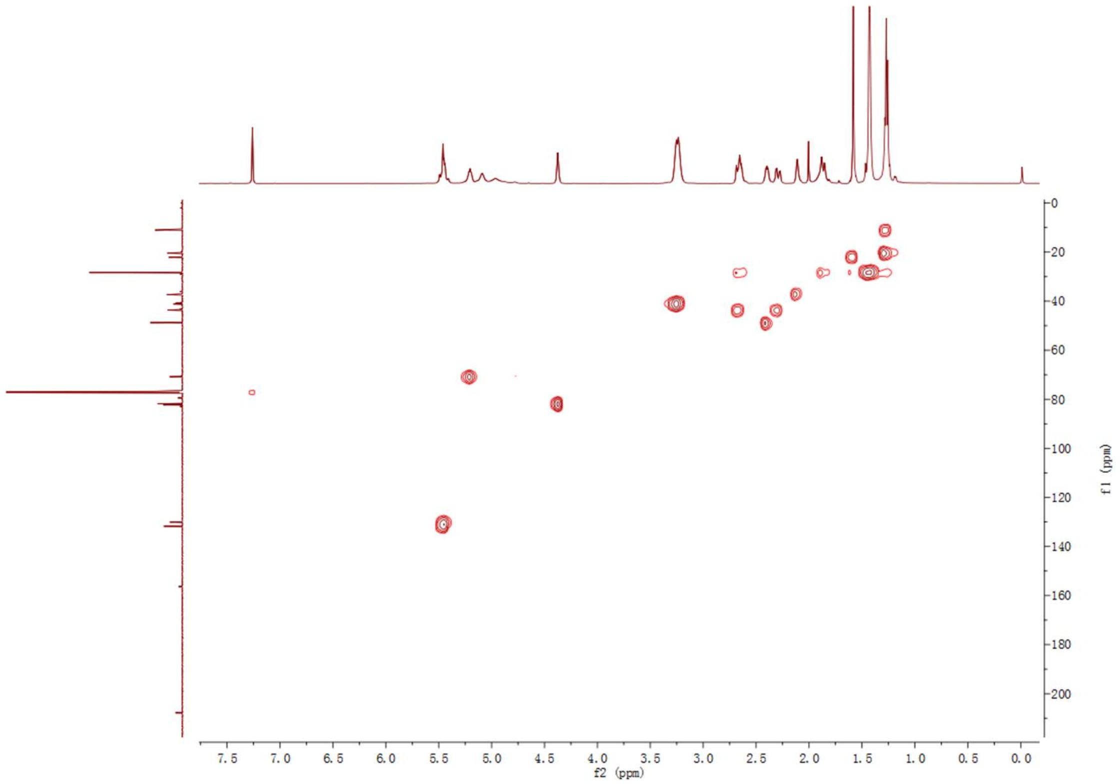

Fig. S109 HSQC spectrum of compound VII-2a in $CDCl_3$ (500 MHz)

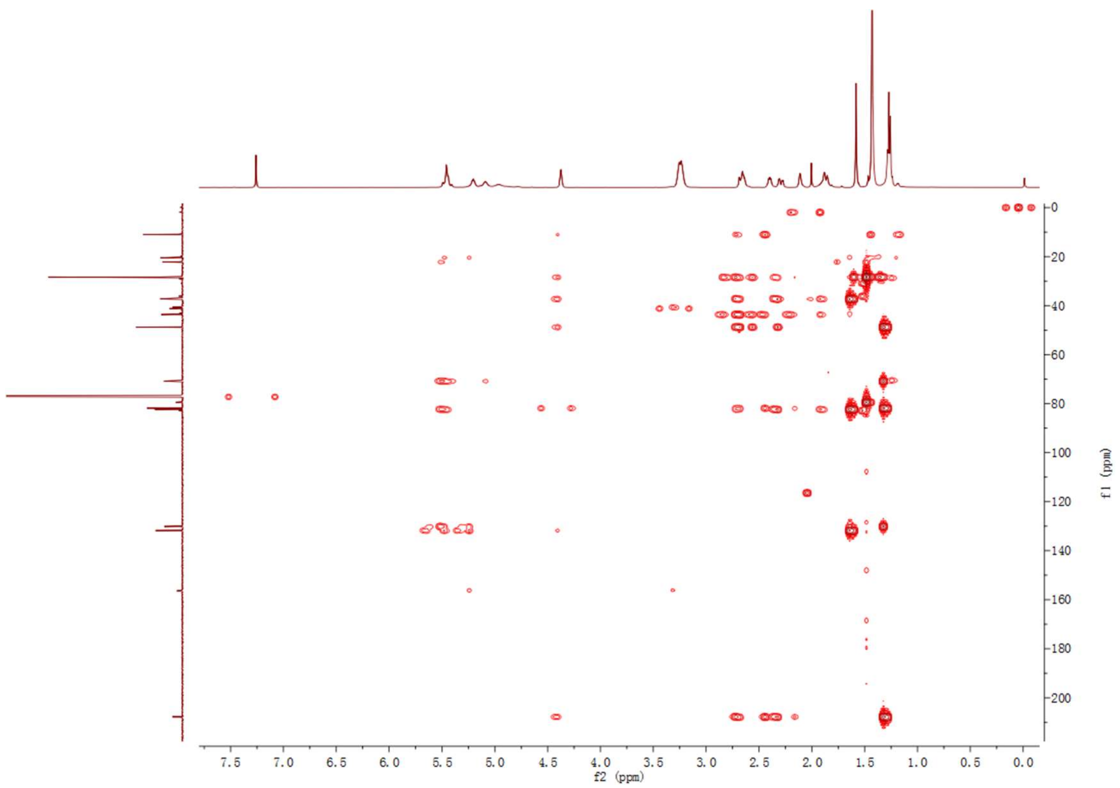

Fig. S110 HMBC spectrum of compound VII-2a in $CDCl_3$ (500 MHz)

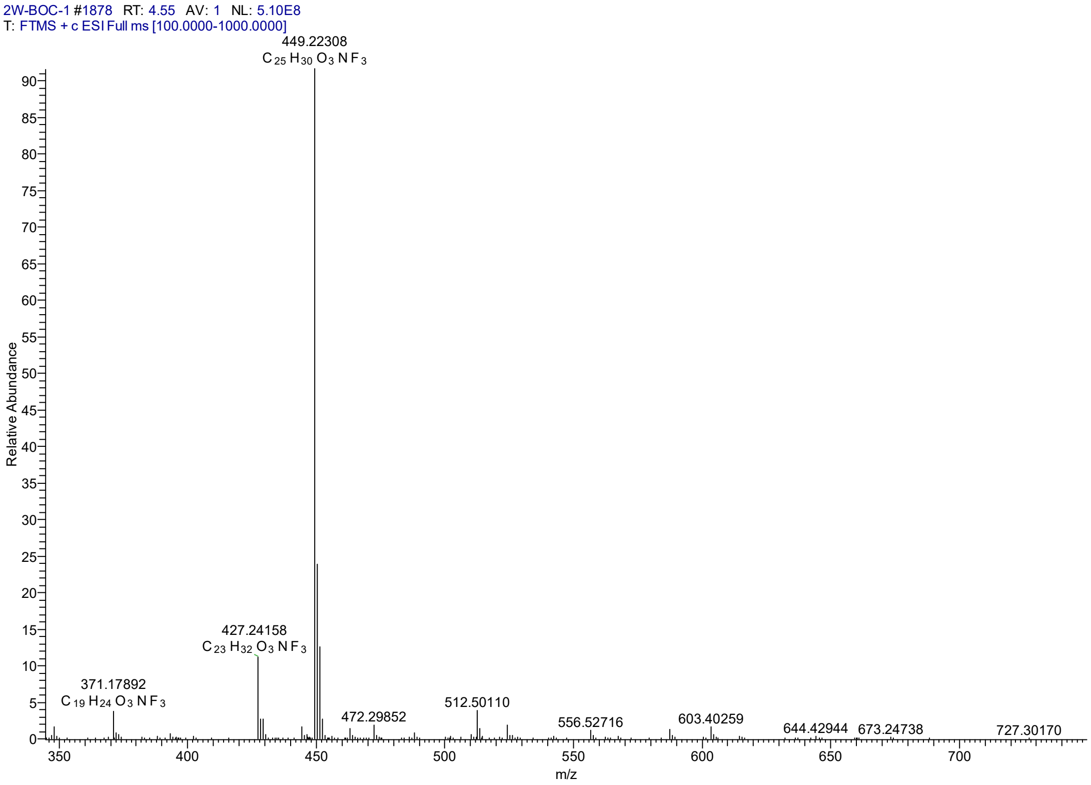

Fig. S111 HRMS data of compound VII-2a

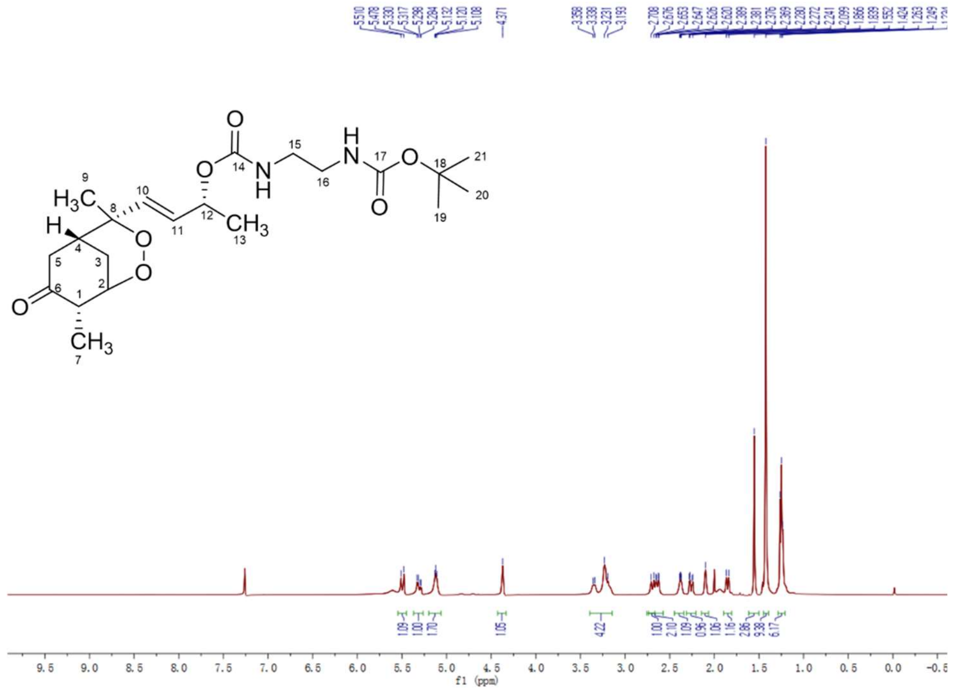

Fig. S112 ^{1}H NMR spectrum of compound VII-2b in $CDCl_3$ (500 MHz)

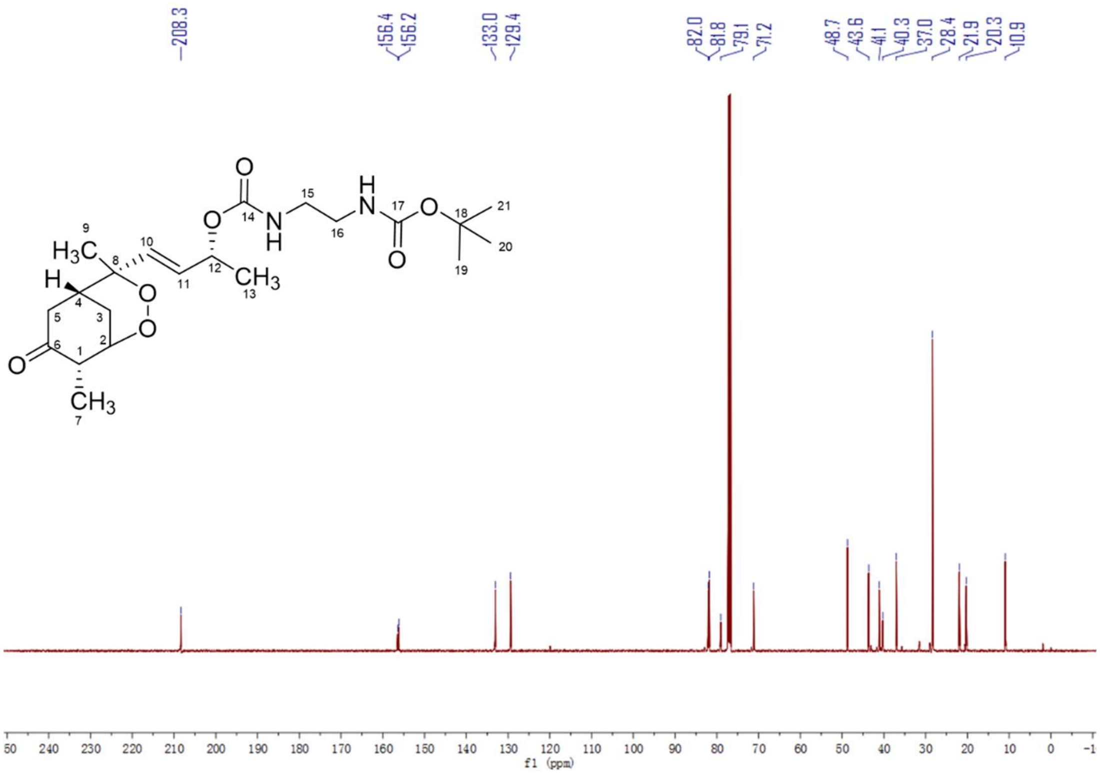

Fig. S113 ^{13}C NMR spectrum of compound VII-2b in $CDCl_3$ (125 MHz)

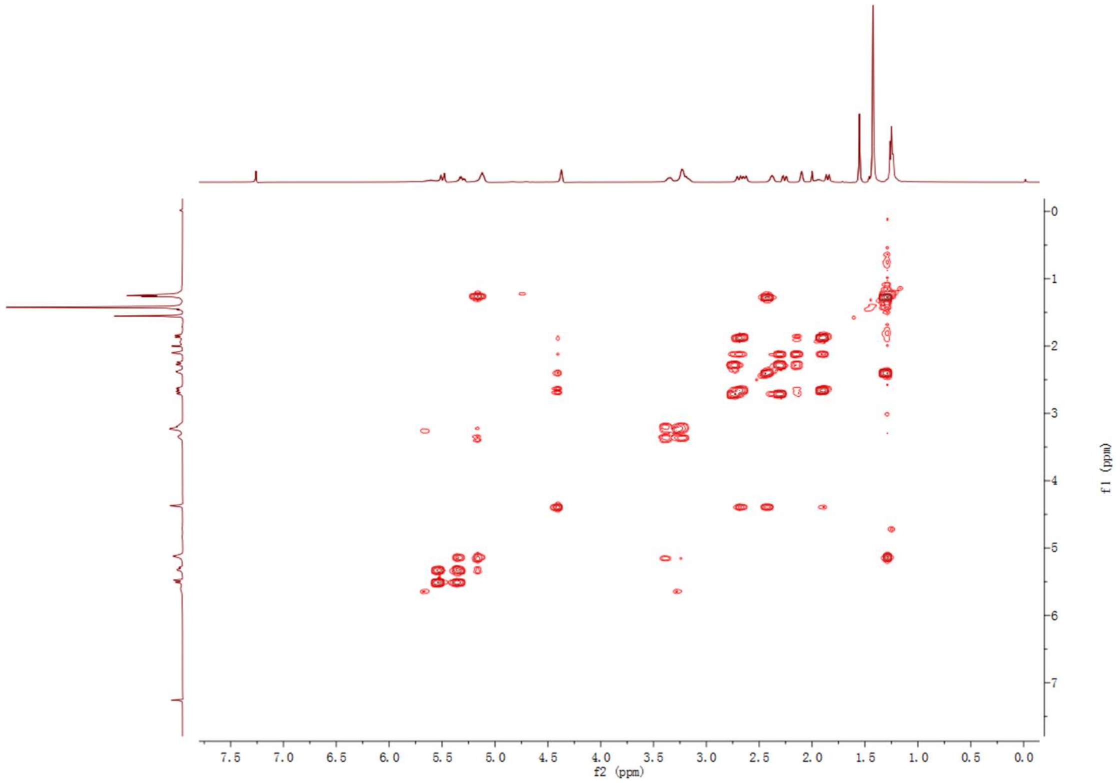

Fig. S114 ^{1}H-^{1}H COSY spectrum of compound VII-2b in $CDCl_3$ (500 MHz)

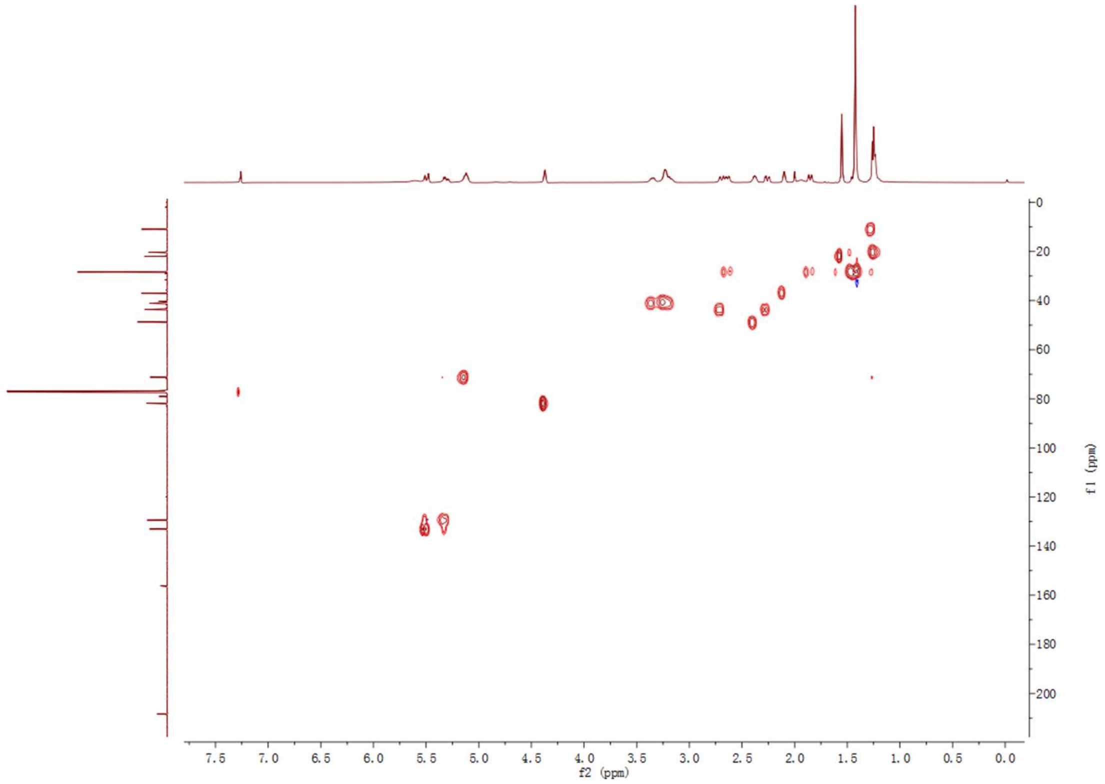

Fig. S115 HSQC spectrum of compound VII-2b in $CDCl_3$ (500 MHz)

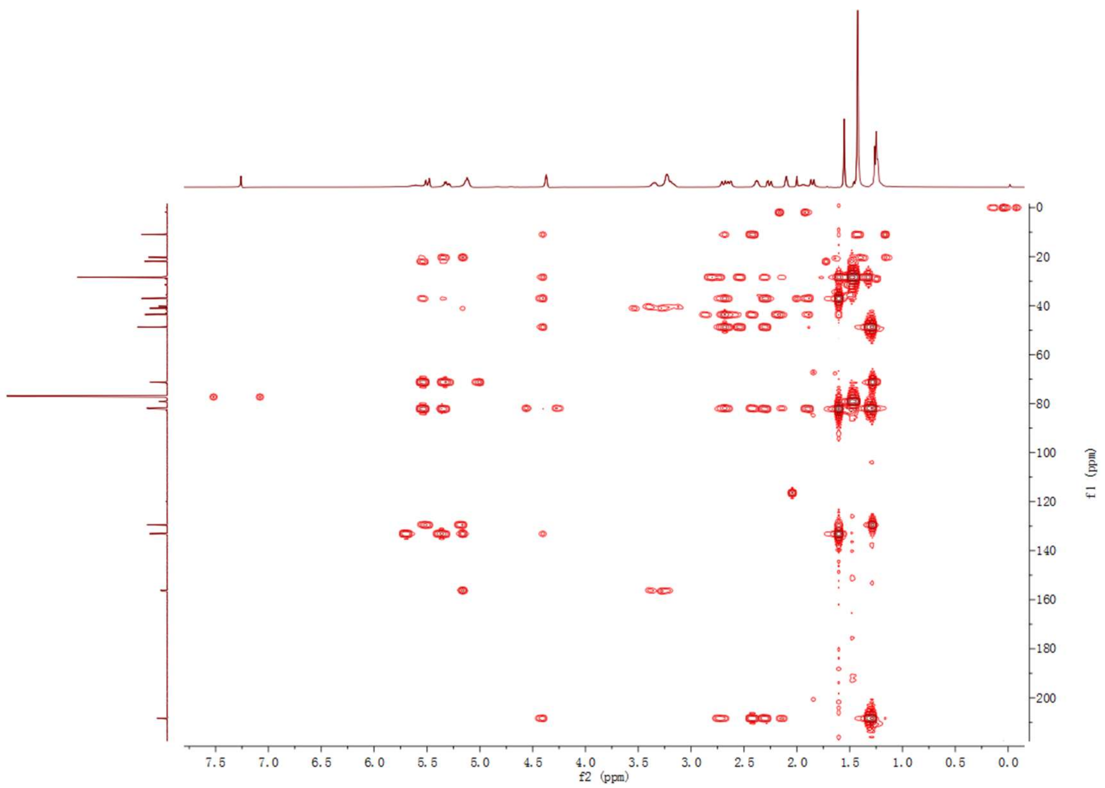

Fig. S116 HMBC spectrum of compound VII-2b in $CDCl_3$ (500 MHz)

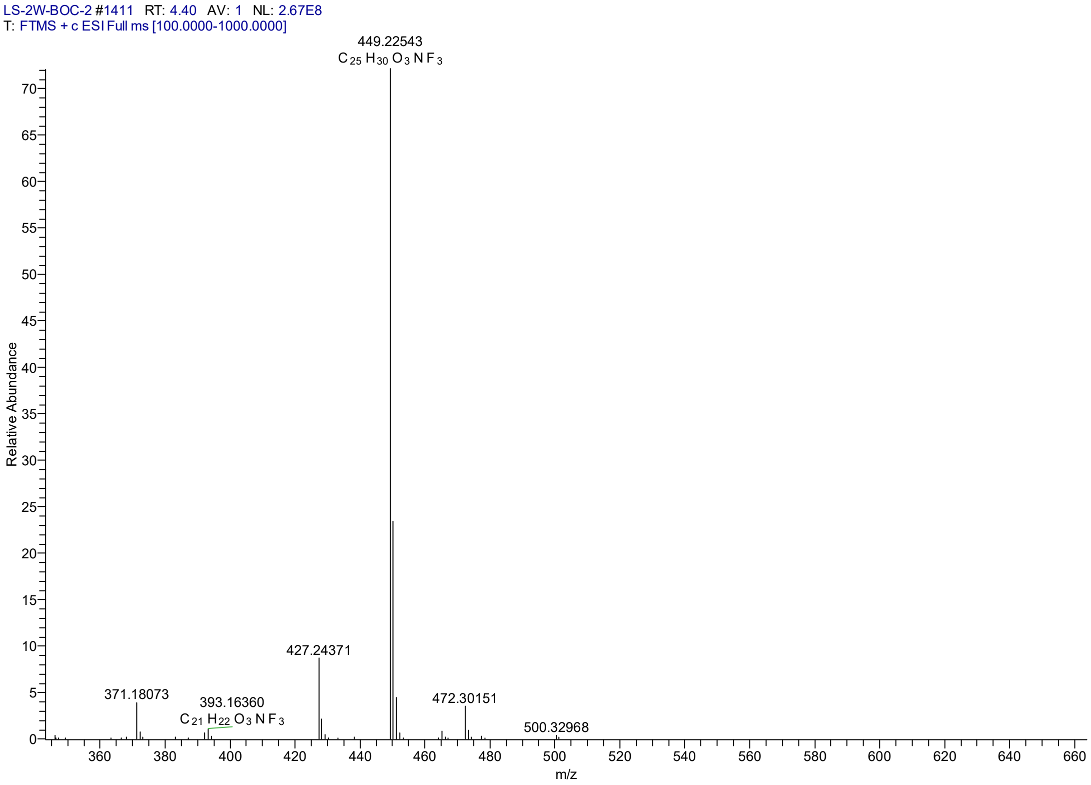

Fig. S117 HRMS data of compound VII-2b

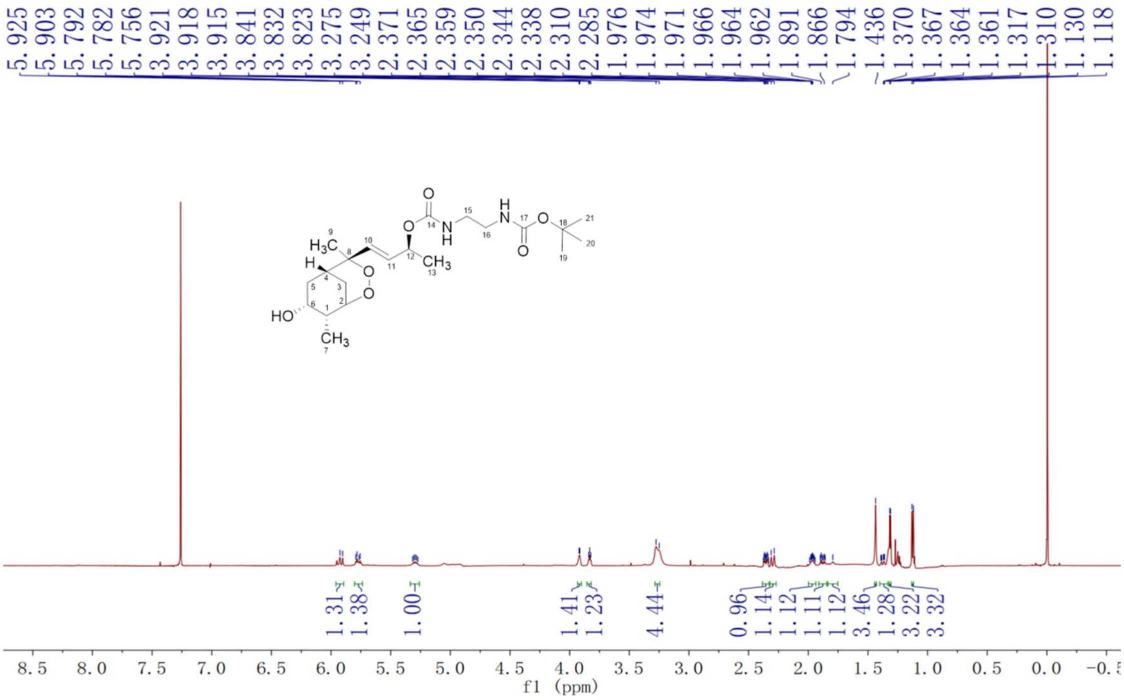

Fig. S118 ^{1}H NMR spectrum of compound VIII-1a in $CDCl_3$ (500 MHz)

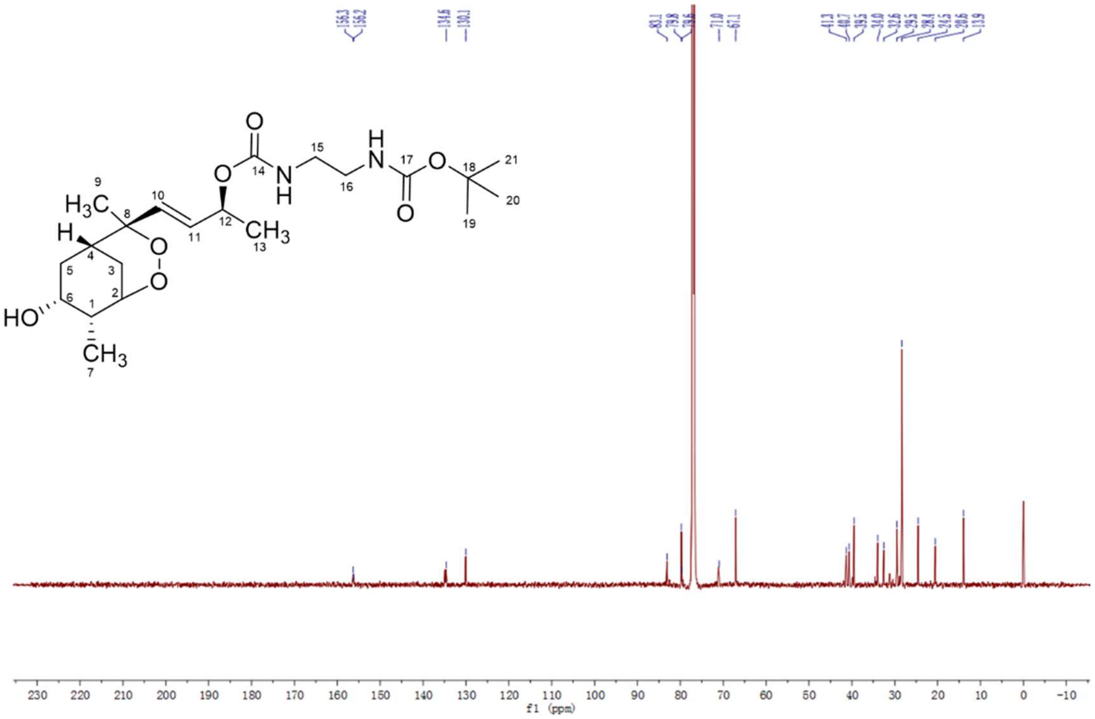

Fig. S119 ^{13}C NMR spectrum of compound VIII-1a in $CDCl_3$ (125 MHz)

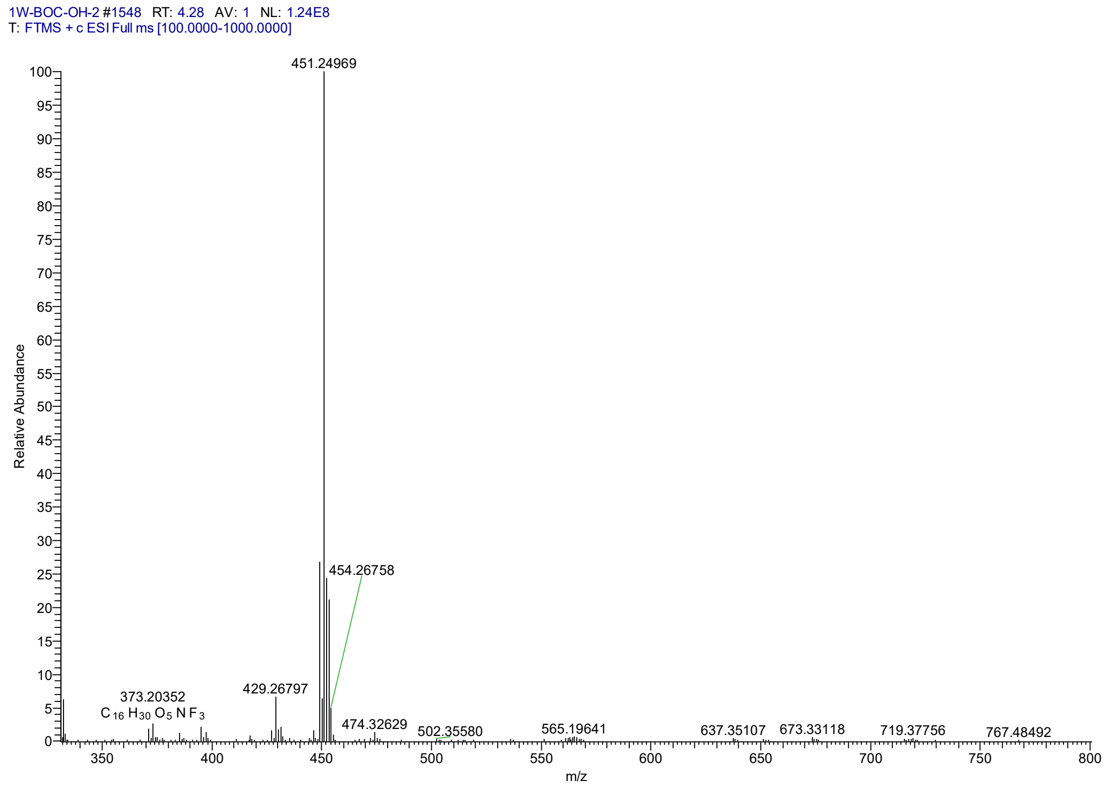

Fig. S120 HRMS data of compound VIII-1a

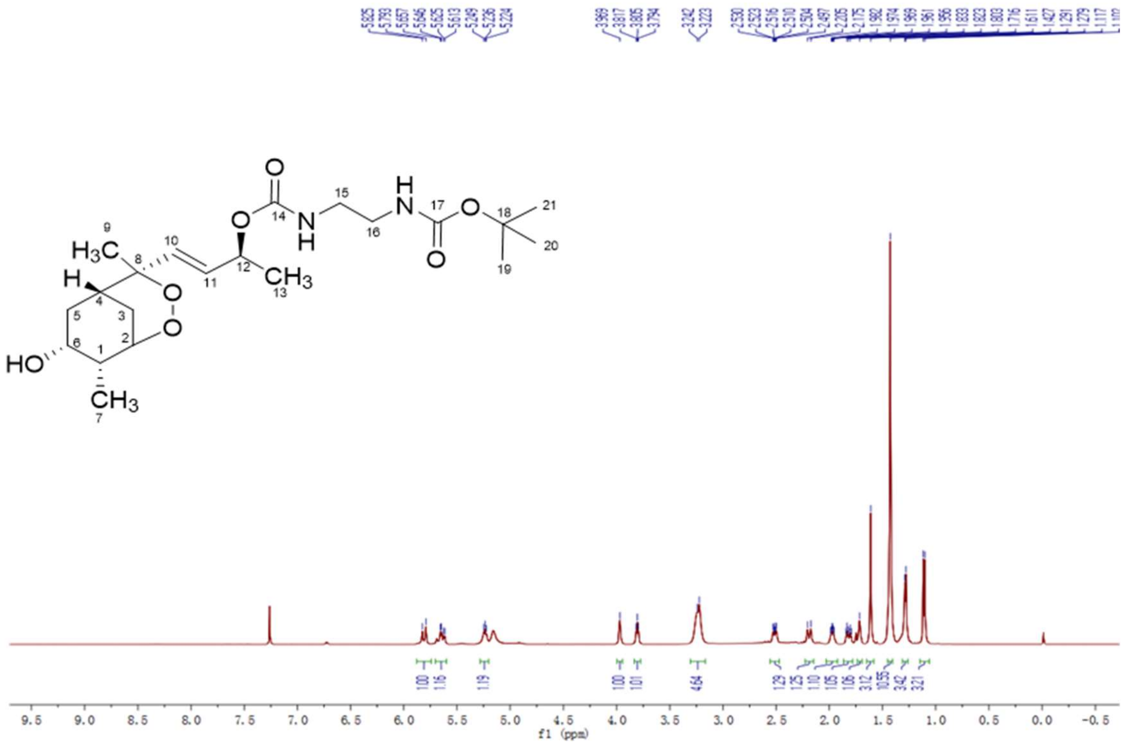

Fig. S121 ^{1}H NMR spectrum of compound VIII-2a in $CDCl_3$ (500 MHz)

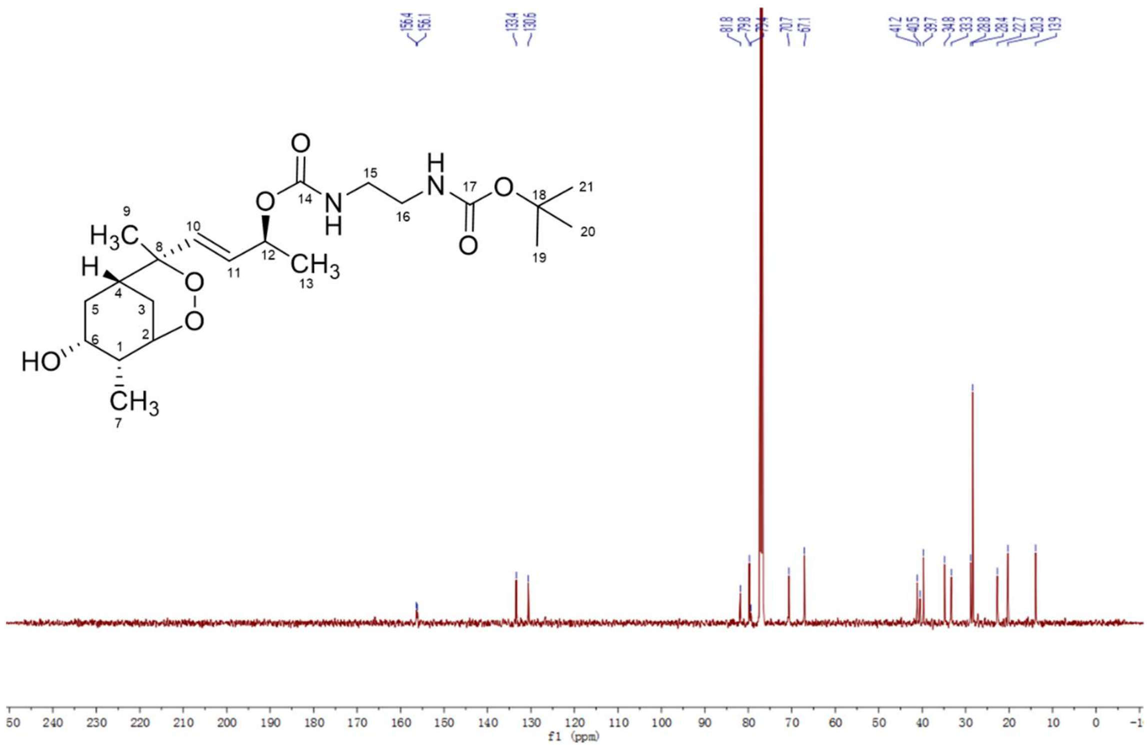

Fig. S122 ^{13}C NMR spectrum of compound VIII-2a in $CDCl_3$ (125 MHz)

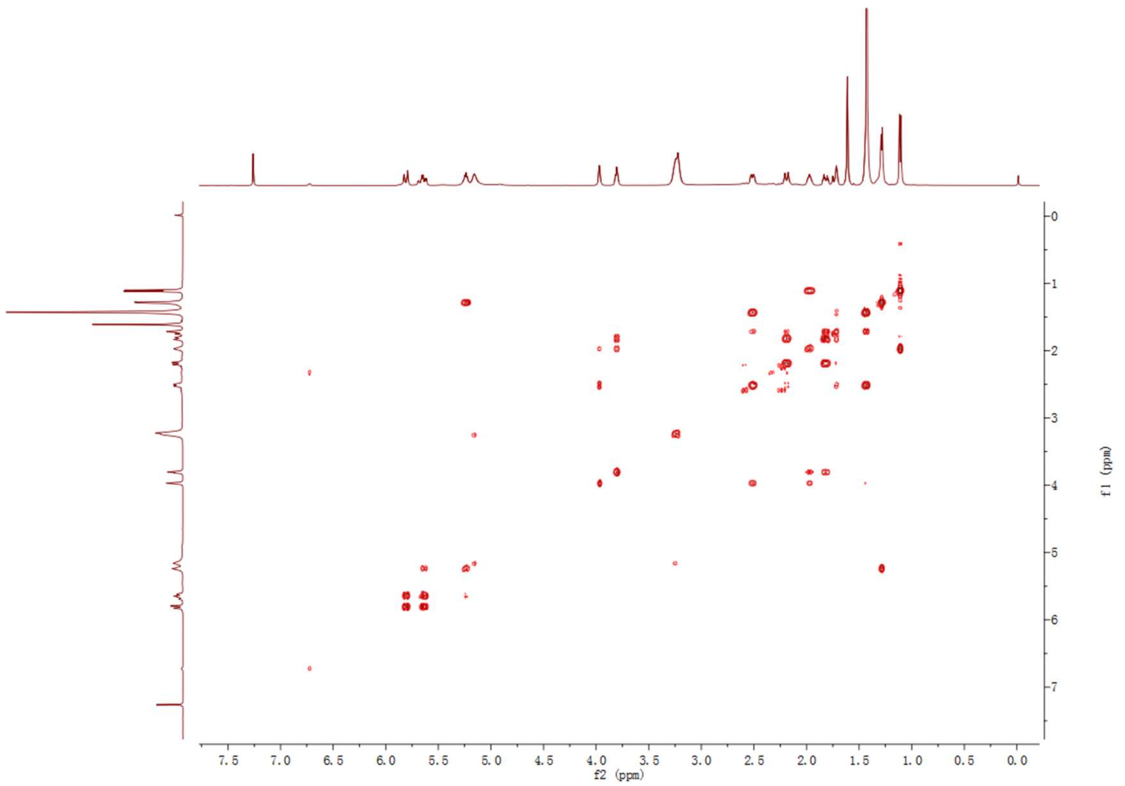

Fig. S123 ^{1}H-^{1}H COSY spectrum of compound VIII-2a in $CDCl_3$ (500 MHz)

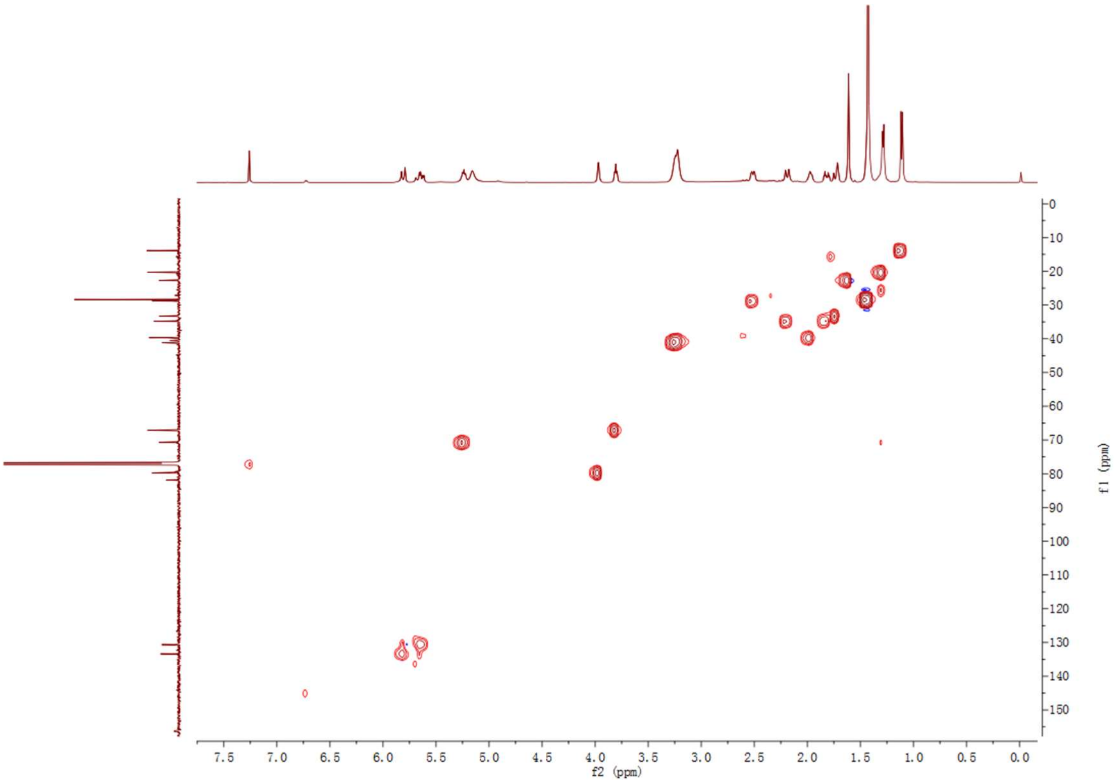

Fig. S124 HSQC spectrum of compound VIII-2a in $CDCl_3$ (500 MHz)

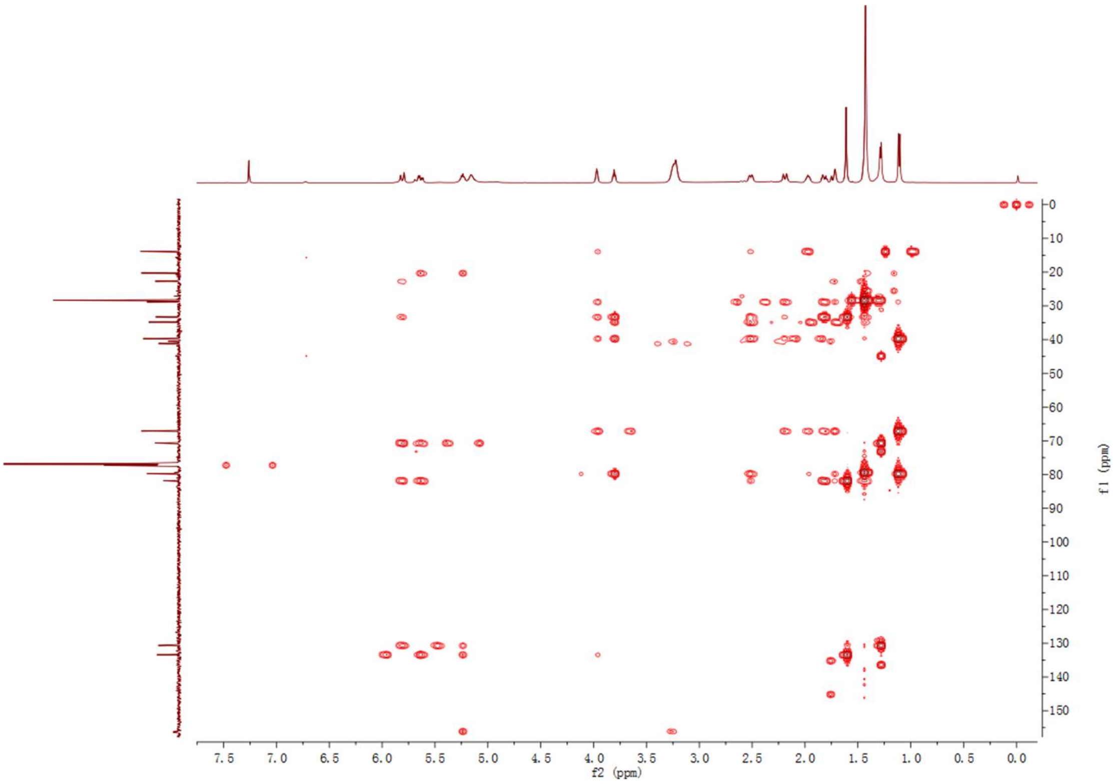

Fig. S125 HMBC spectrum of compound VIII-2a in $CDCl_3$ (500 MHz)

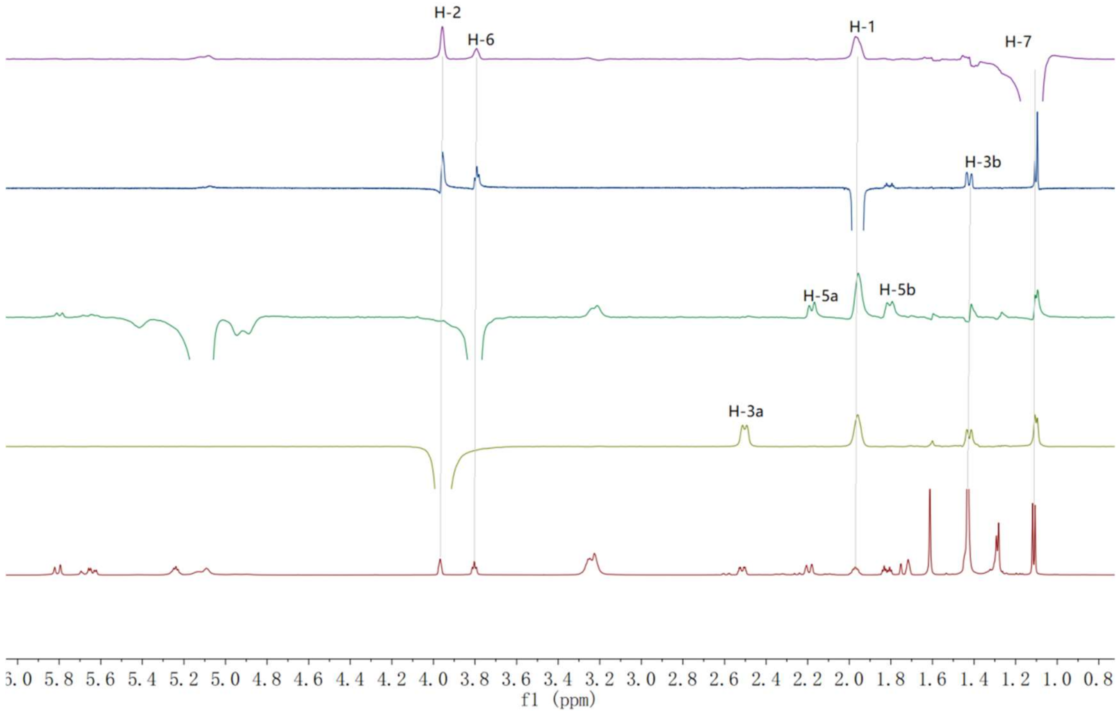

Fig. S126 NOE difference spectra of compound VIII-2a in $CDCl_3$ (600 MHz)

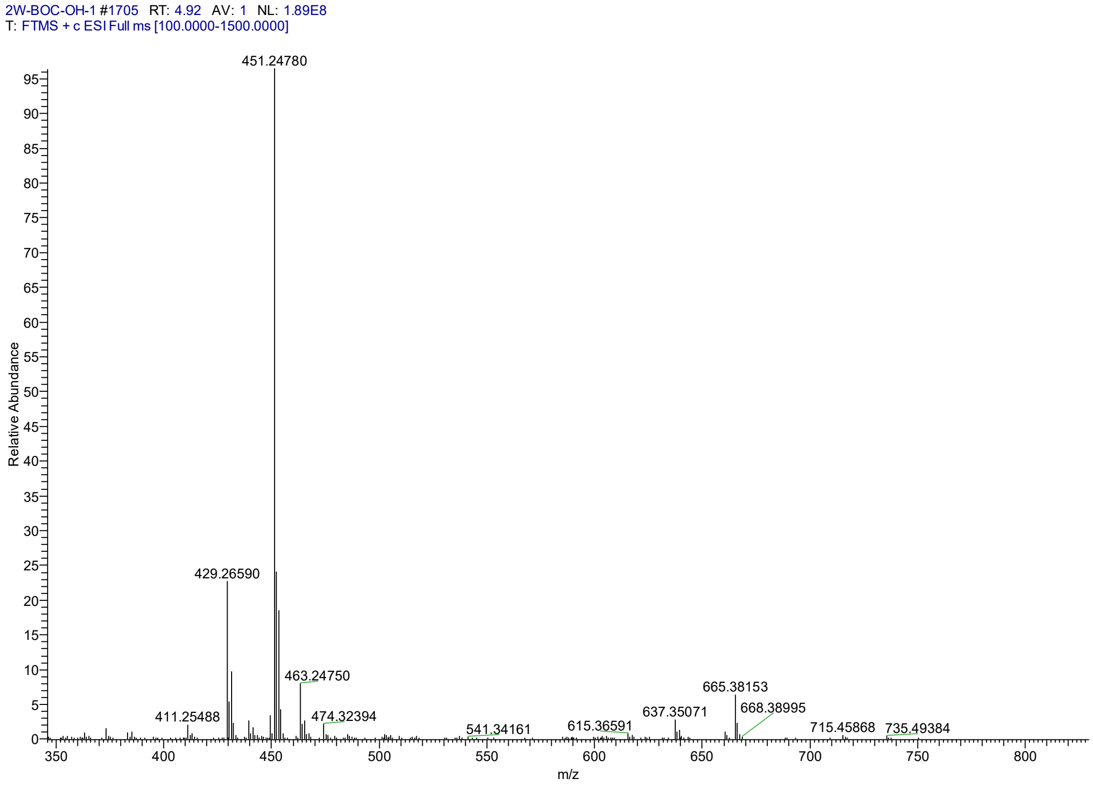

Fig. S127 HRMS data of compound VIII-2a

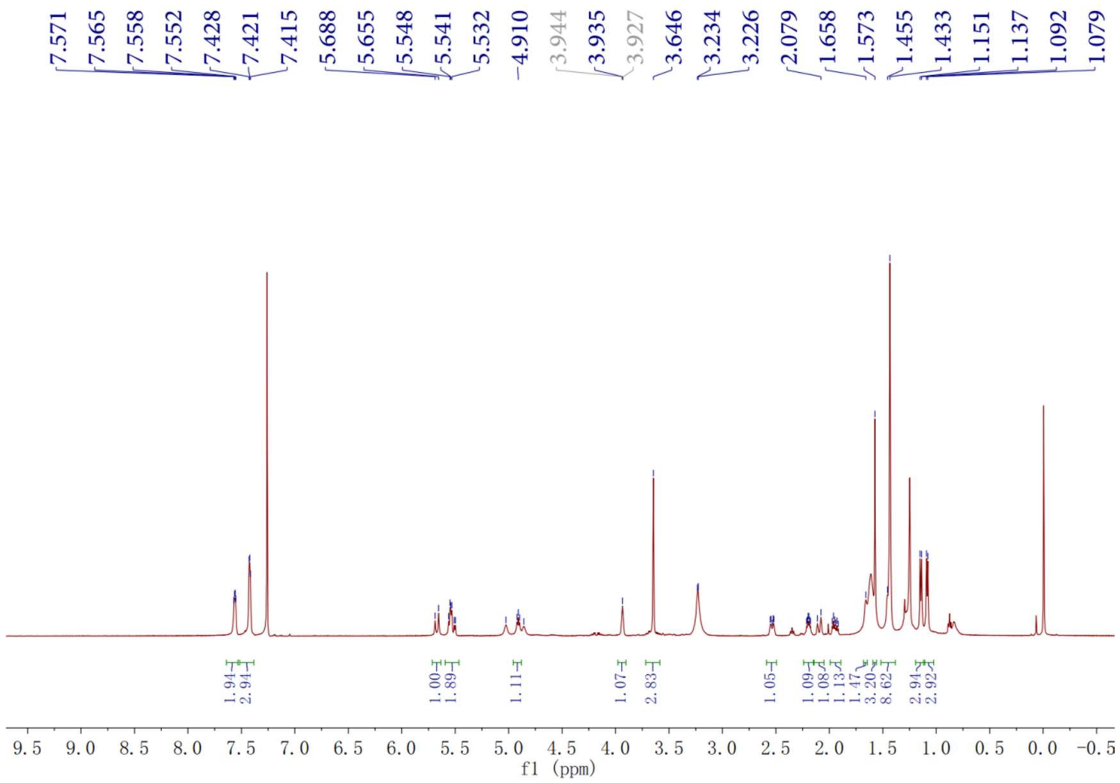

Fig. S128 ^{1}H NMR spectrum of VIII-2a (*R*-MTPA ester) in $CDCl_3$ (500 MHz)

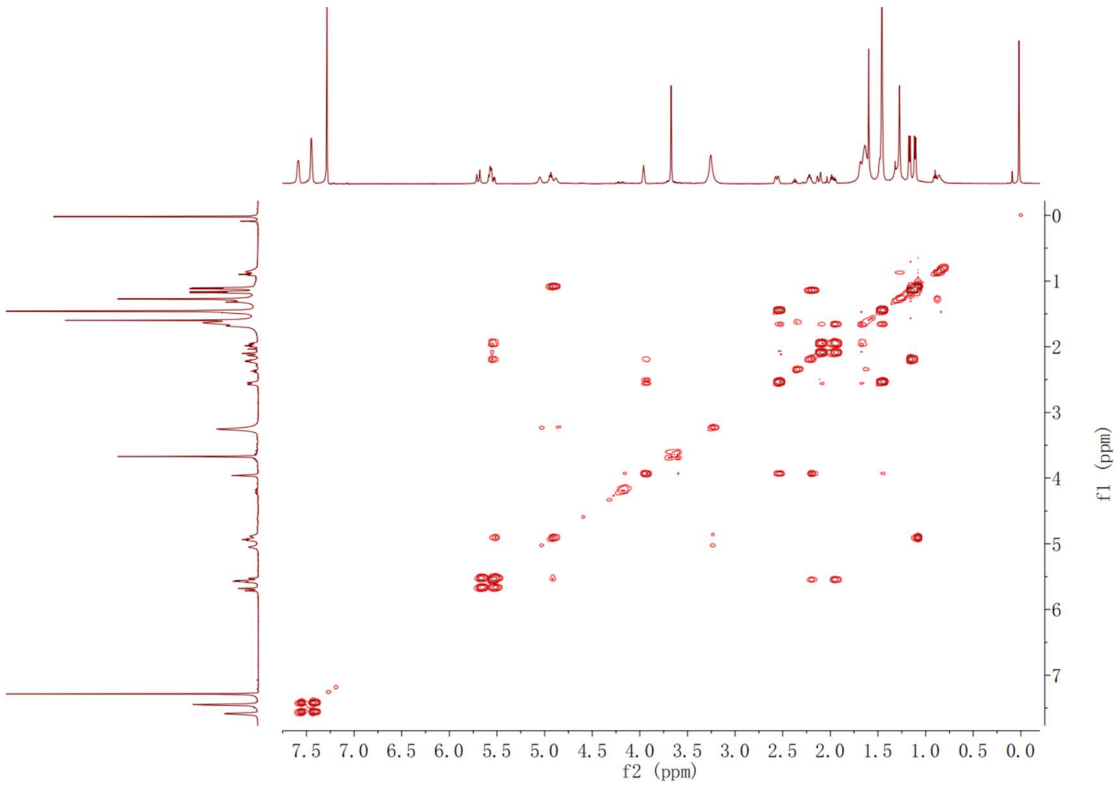

Fig. S129 ^{1}H-^{1}H COSY spectrum of VIII-2a (R-MTPA ester) in $CDCl_3$ (500 MHz)

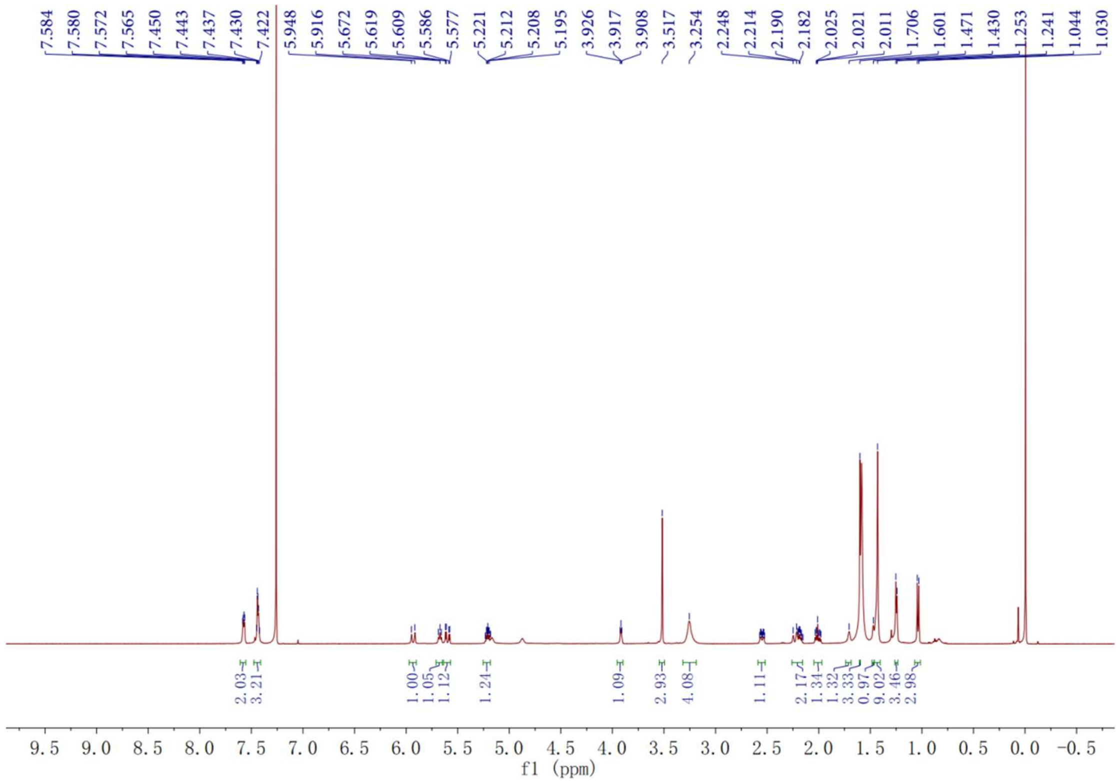

Fig. S130 ^{1}H NMR spectrum of VIII-2a (*S*-MTPA ester) in $CDCl_3$ (500 MHz)

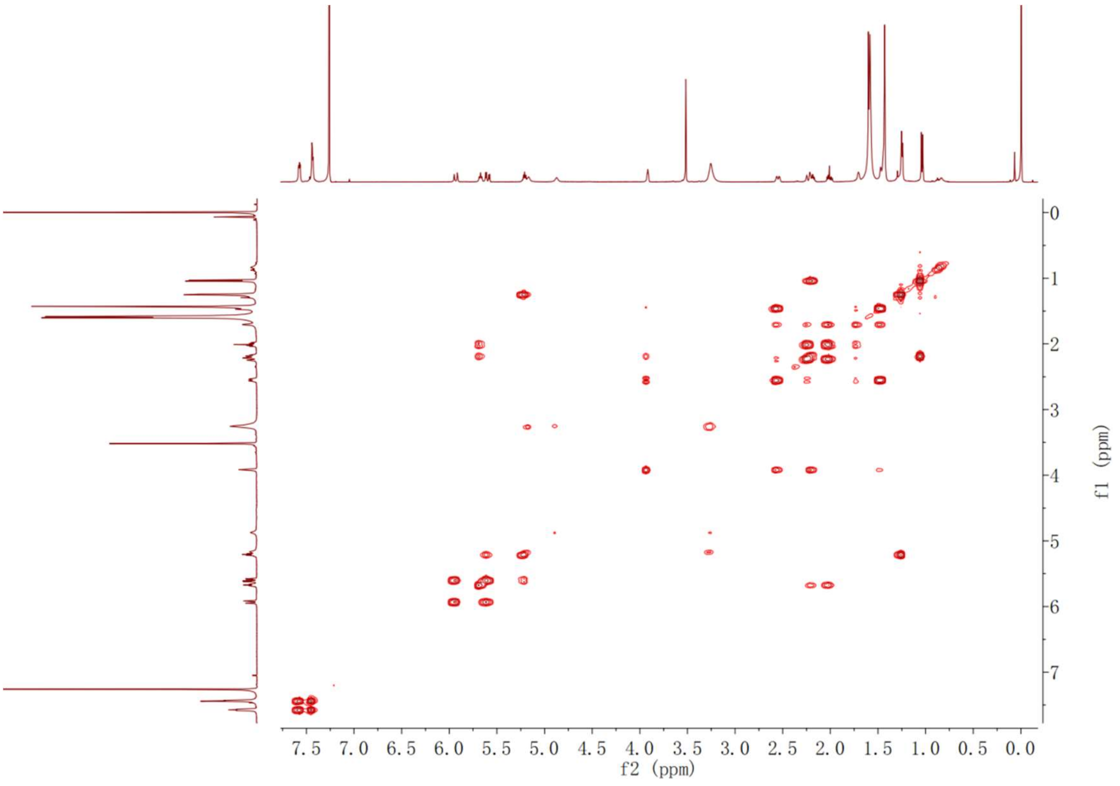

Fig. S131 ^{1}H-^{1}H COSY spectrum of VIII-2a (S-MTPA ester) in $CDCl_3$ (500 MHz)

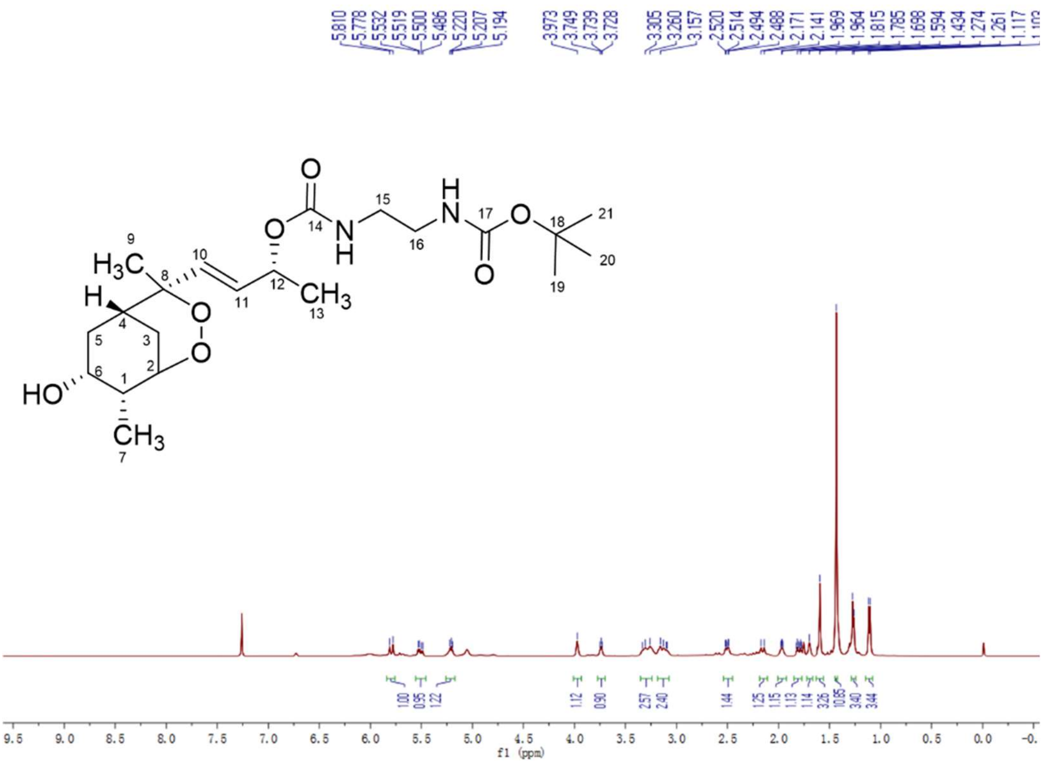

Fig. S132 ^{1}H NMR spectrum of compound VIII-2b in $CDCl_3$ (500 MHz)

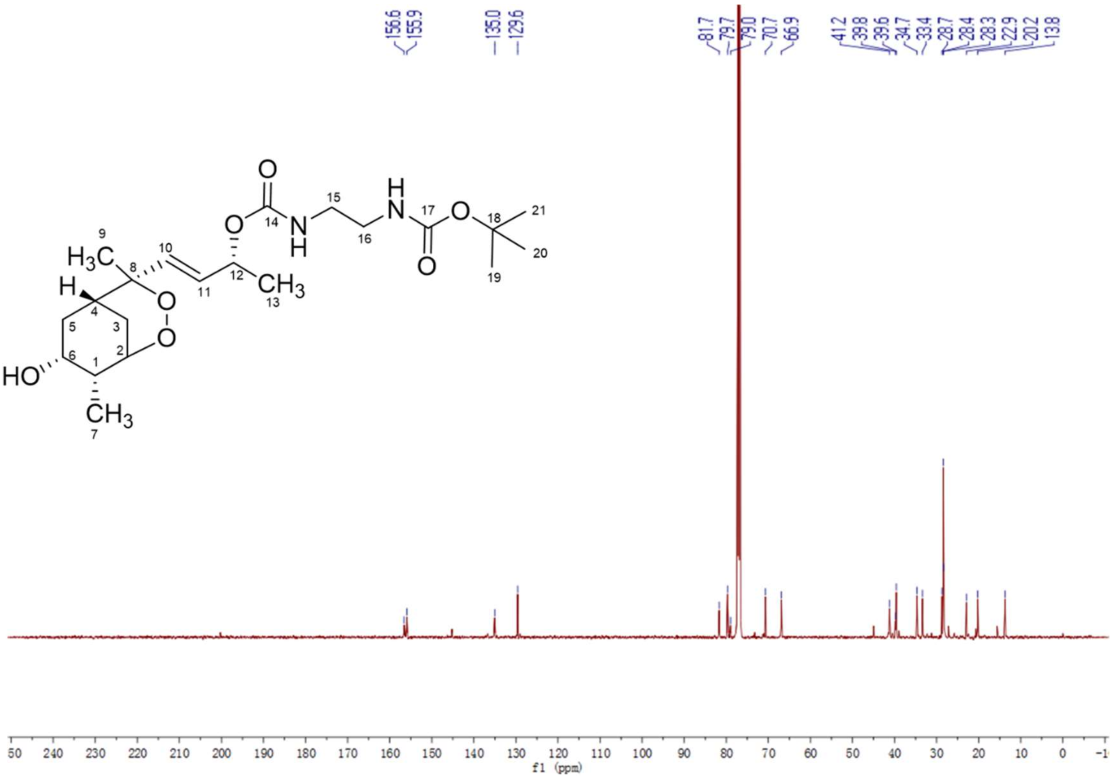

Fig. S133 ^{13}C NMR spectrum of compound VIII-2b in $CDCl_3$ (125 MHz)

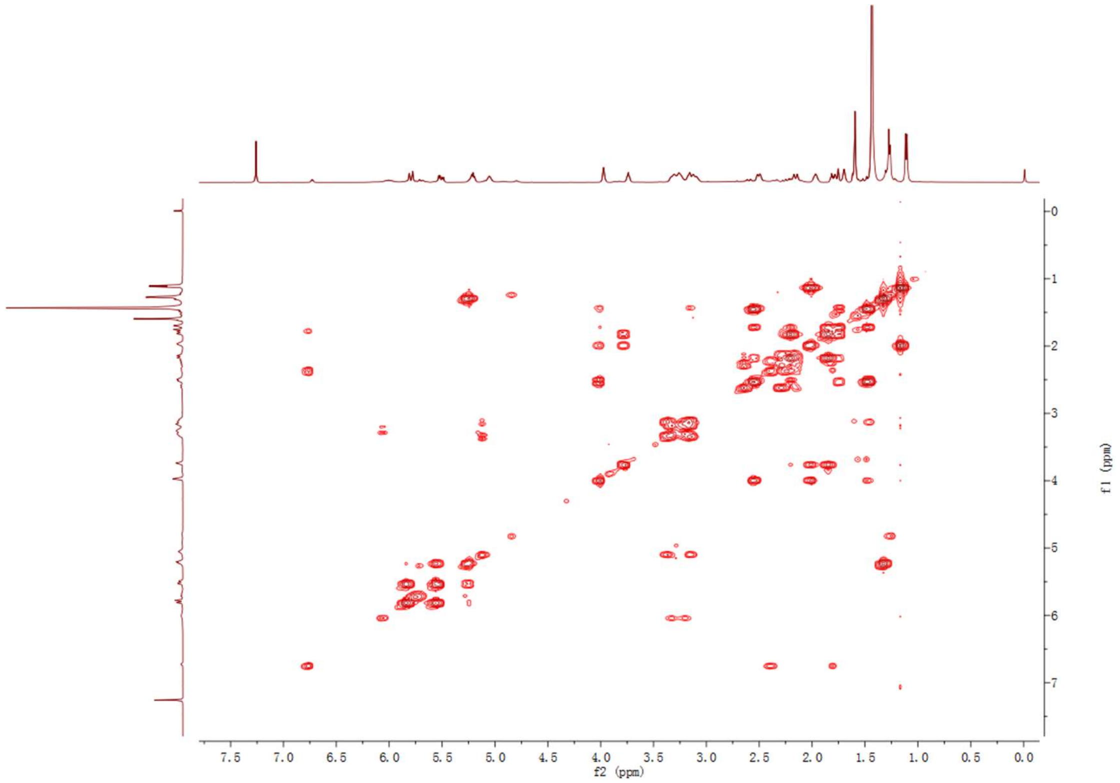

Fig. S134 ¹H-¹H COSY spectrum of compound VIII-2b in CDCl₃ (500 MHz)

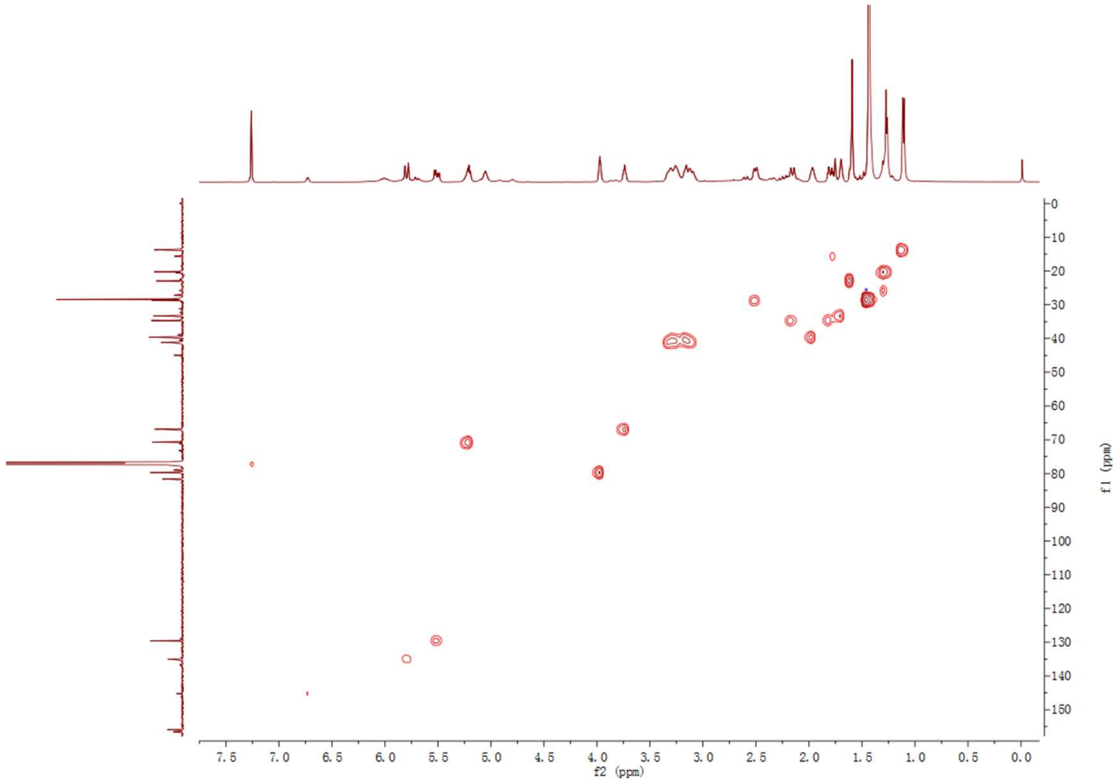

Fig. S135 HSQC spectrum of compound VIII-2b in CDCl₃ (500 MHz)

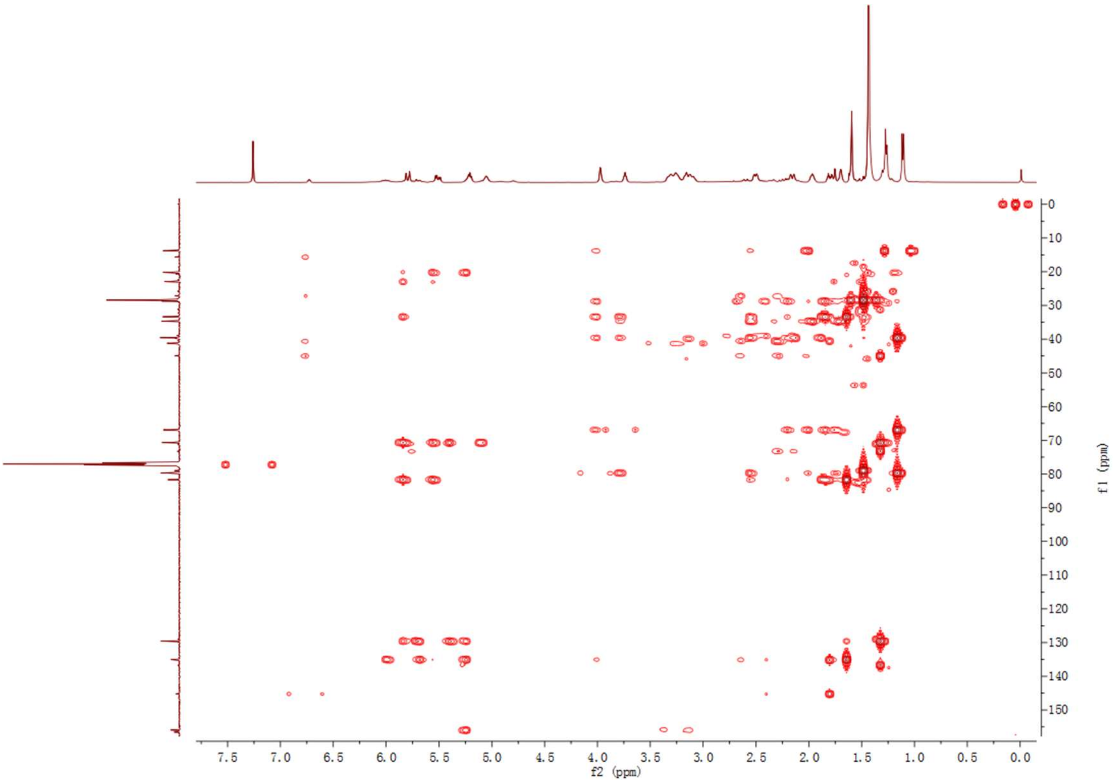

Fig. S136 HMBC spectrum of compound VIII-2b in CDCl₃ (500 MHz)

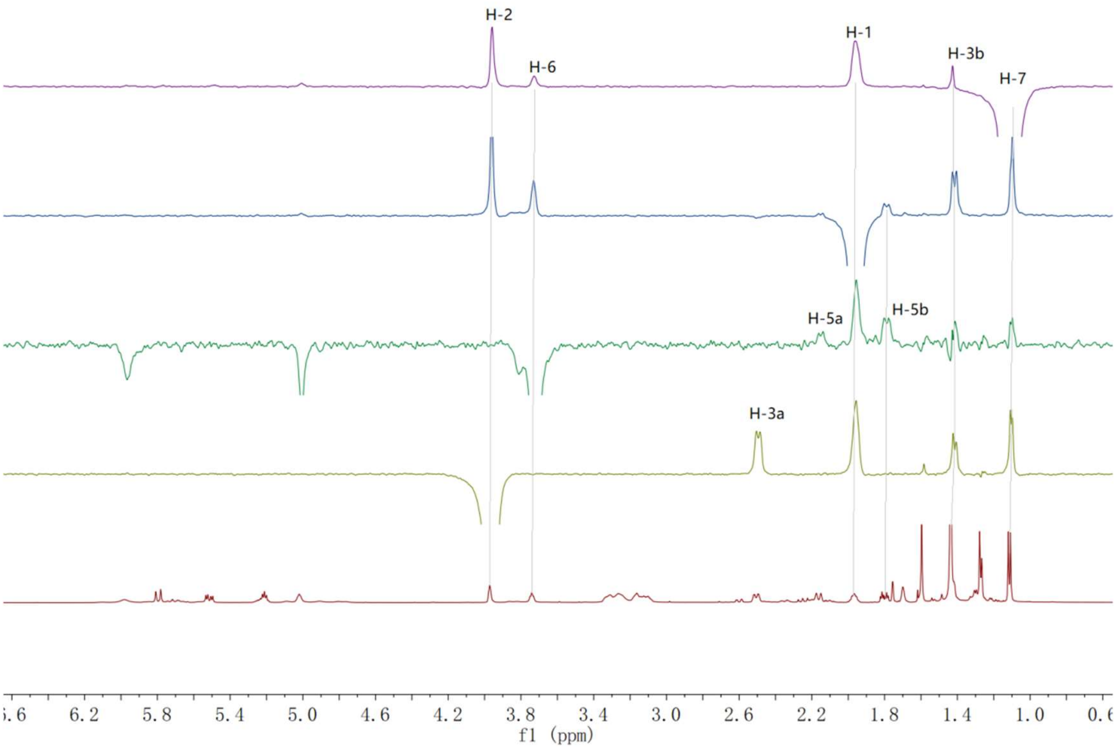

Fig. S137 NOE difference spectra of compound VIII-2b in CDCl₃ (600 MHz)

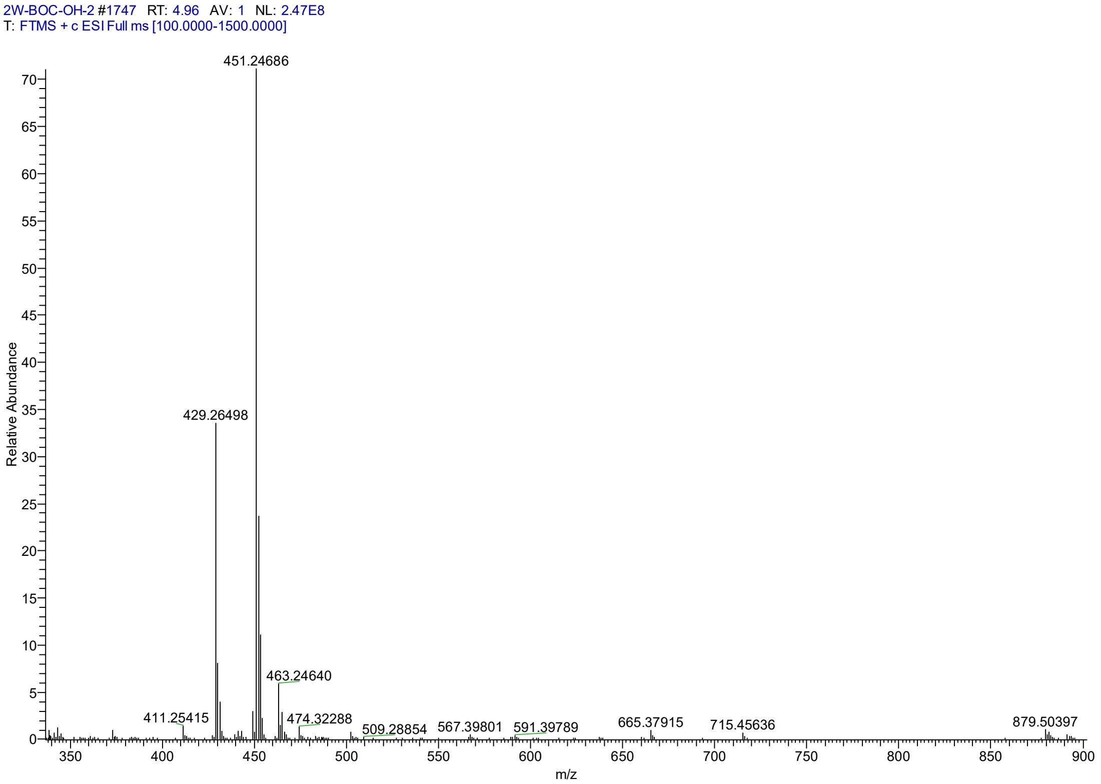

Fig. S138 HRMS data of compound VIII-2b

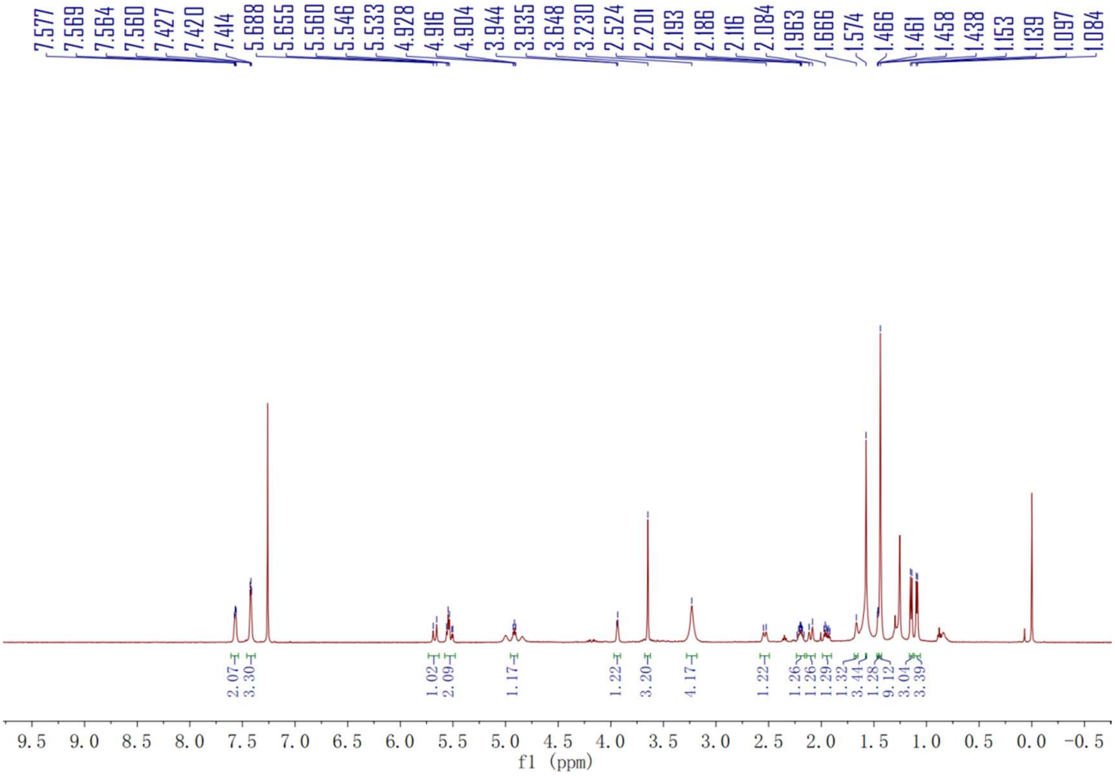

Fig. S139 ^{1}H NMR spectrum of VIII-2b (*R*-MTPA ester) in $CDCl_3$ (500 MHz)

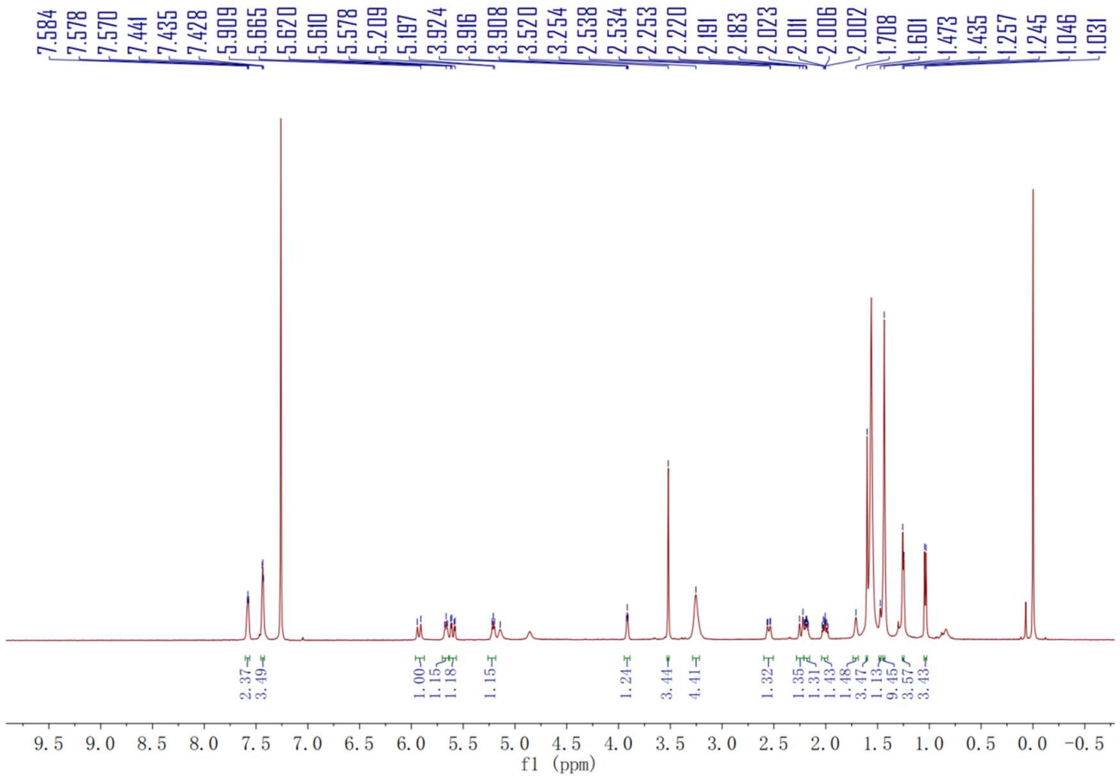

Fig. S140 ^{1}H NMR spectrum of VIII-2b (*S*-MTPA ester) in $CDCl_3$ (500 MHz)

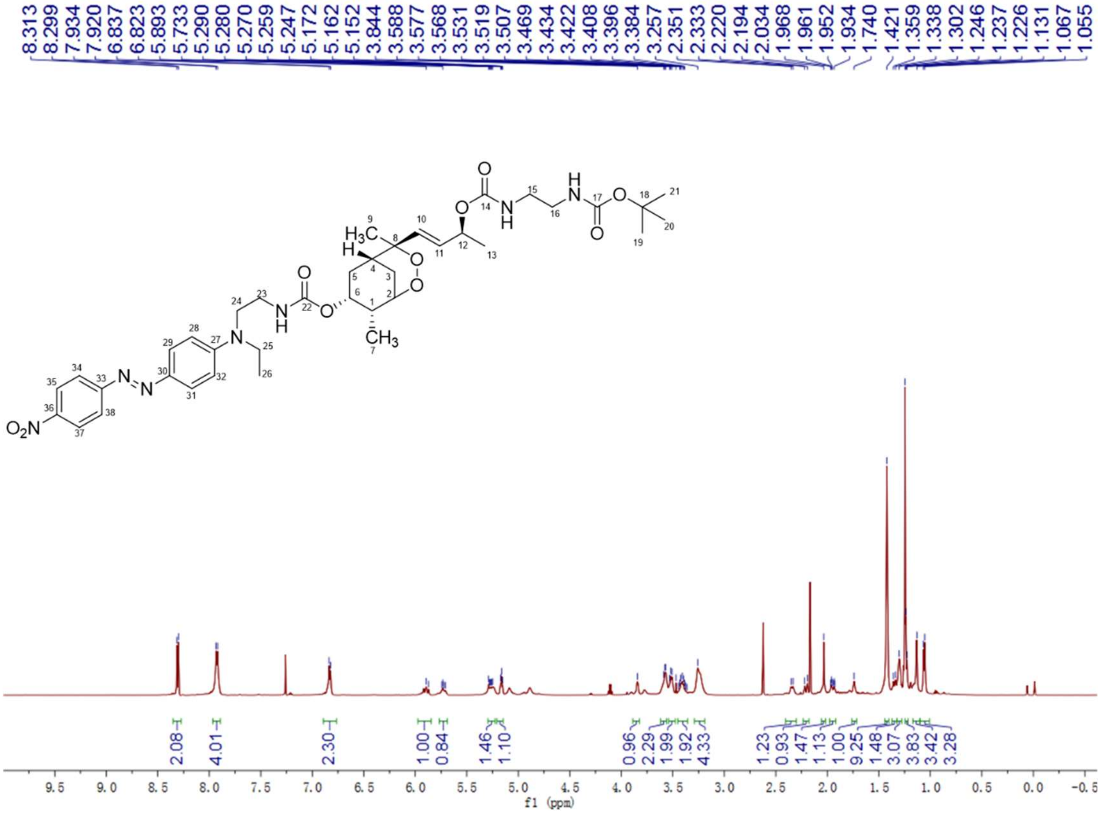

Fig. S141 ^{1}H NMR spectrum of compound IX-1a in $CDCl_3$ (500 MHz)

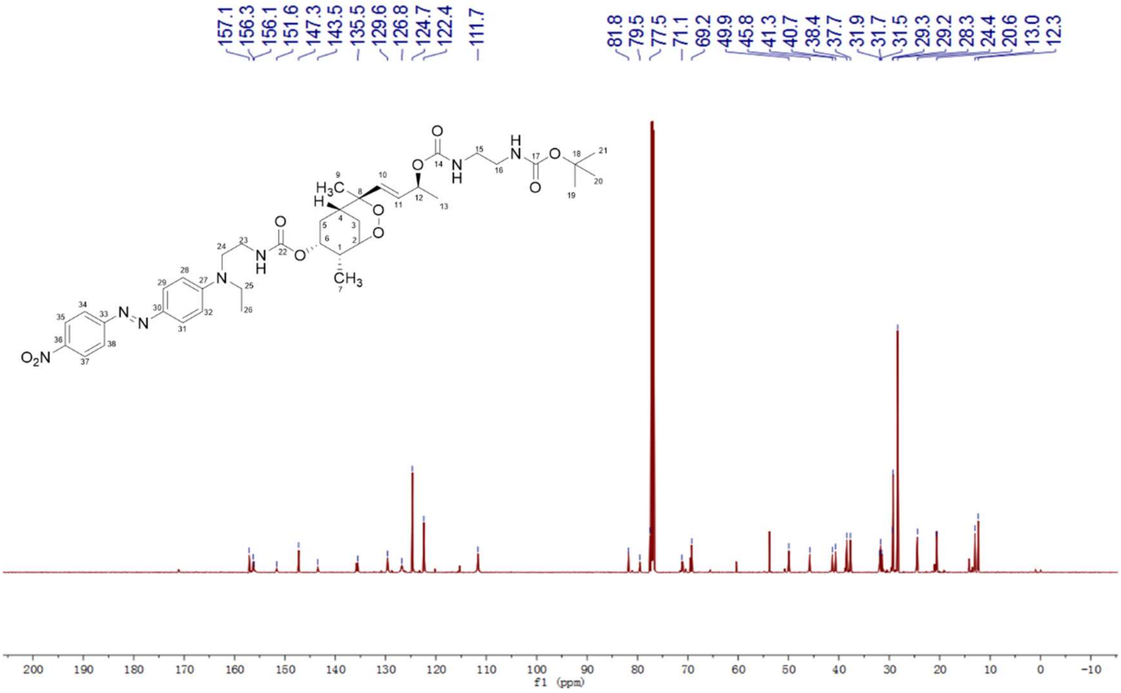

Fig. S142 ^{13}C NMR spectrum of compound IX-1a in $CDCl_3$ (125 MHz)

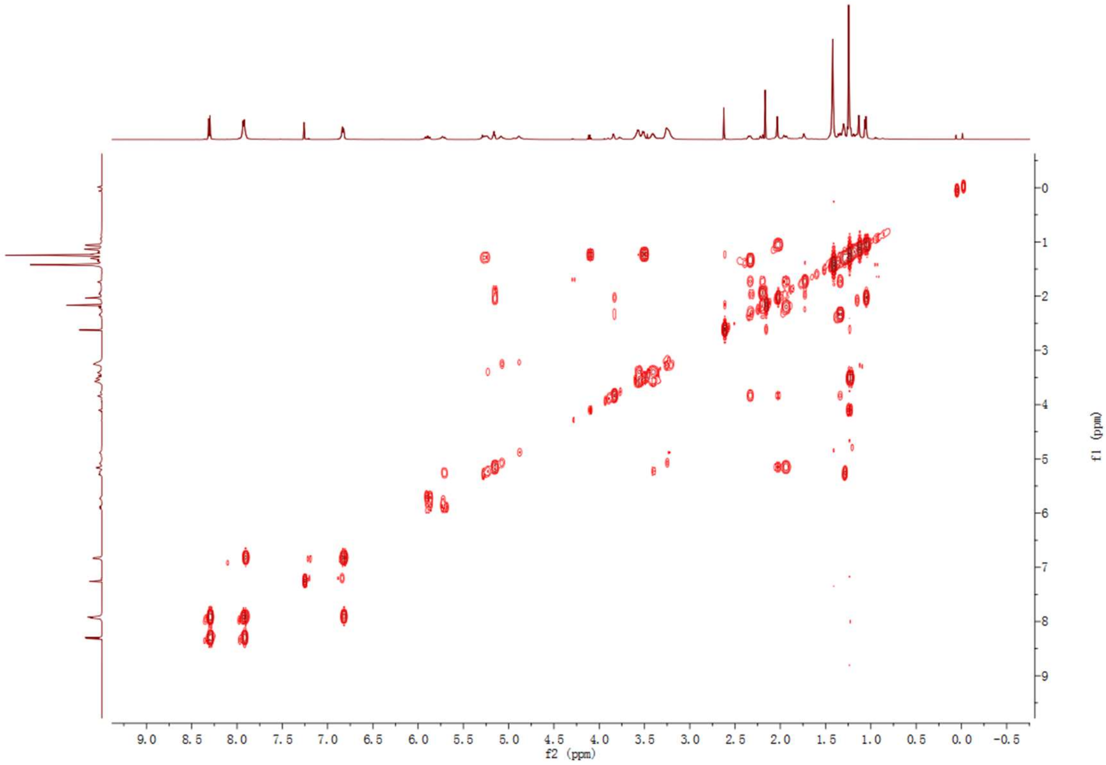

Fig. S143 ^{1}H-^{1}H COSY spectrum of compound IX-1a in $CDCl_3$ (500 MHz)

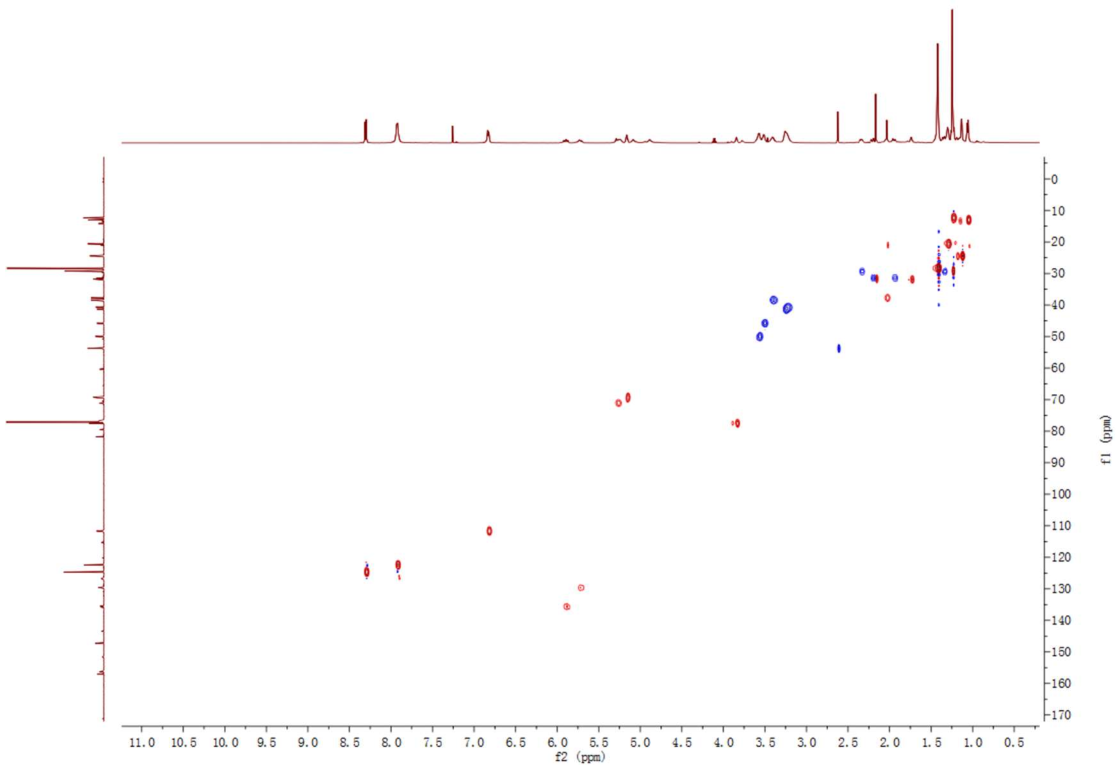

Fig. S144 HSQC spectrum of compound IX-1a in $CDCl_3$ (500 MHz)

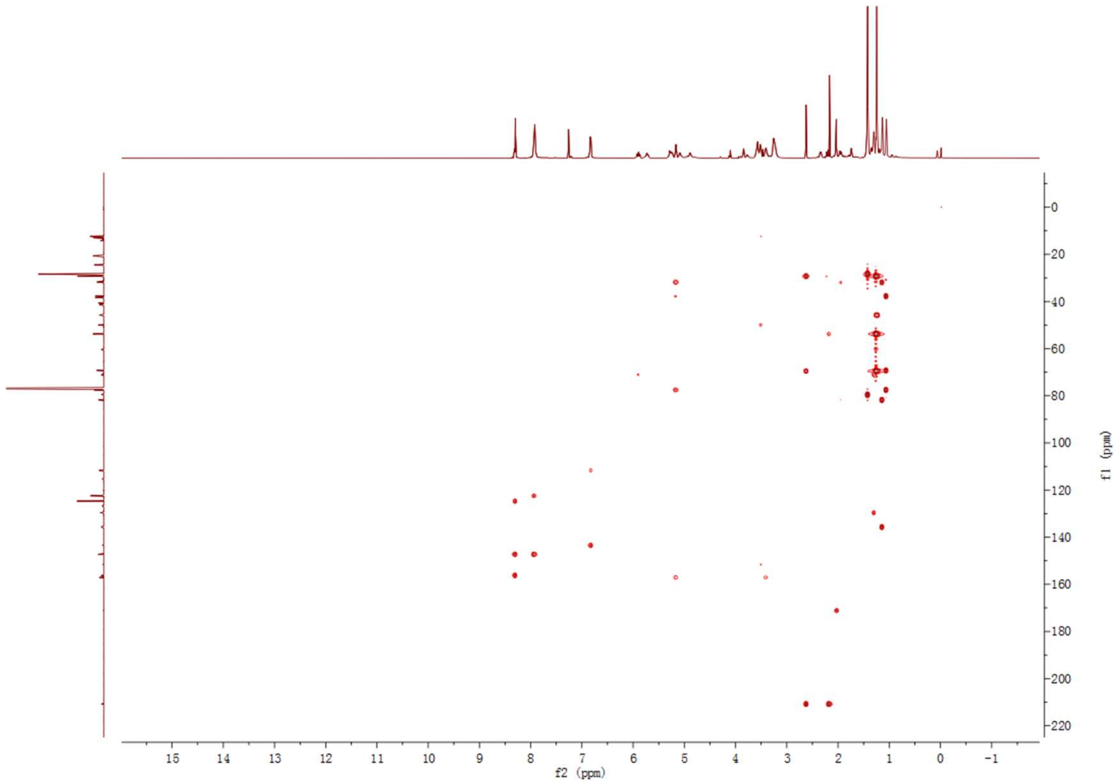

Fig. S145 HMBC spectrum of compound IX-1a in $CDCl_3$ (500 MHz)

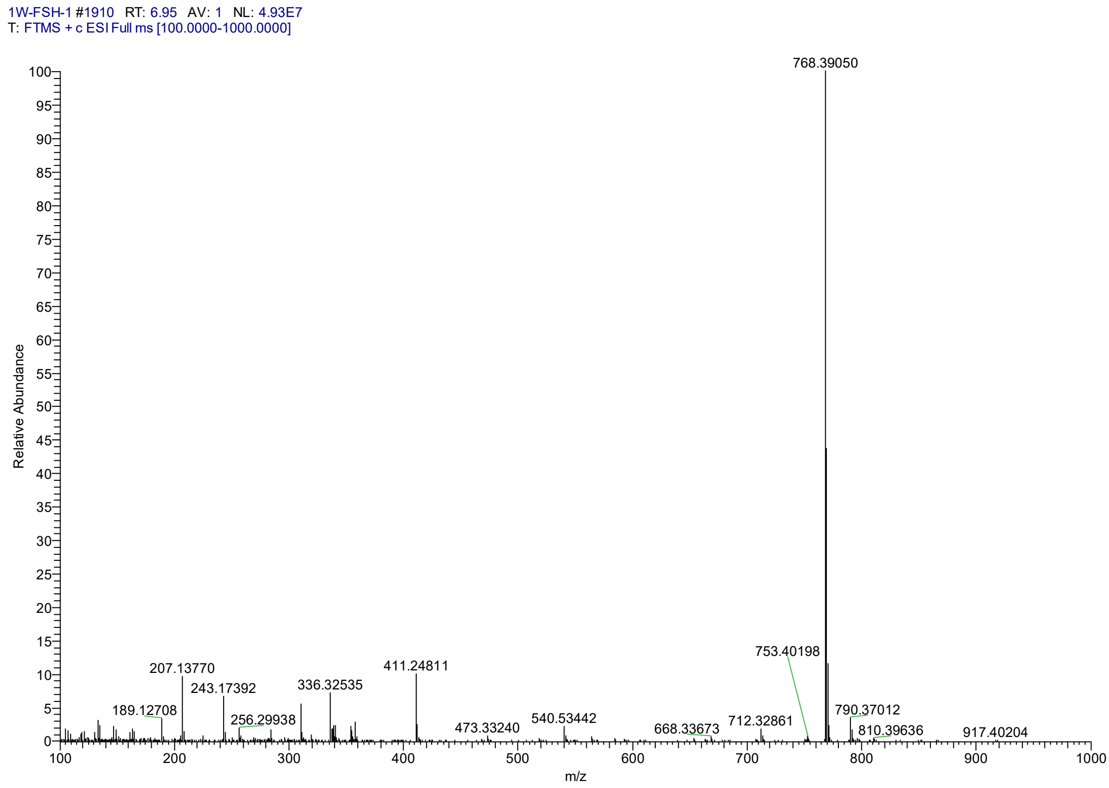

Fig. S146 HRMS data of compound IX-1a

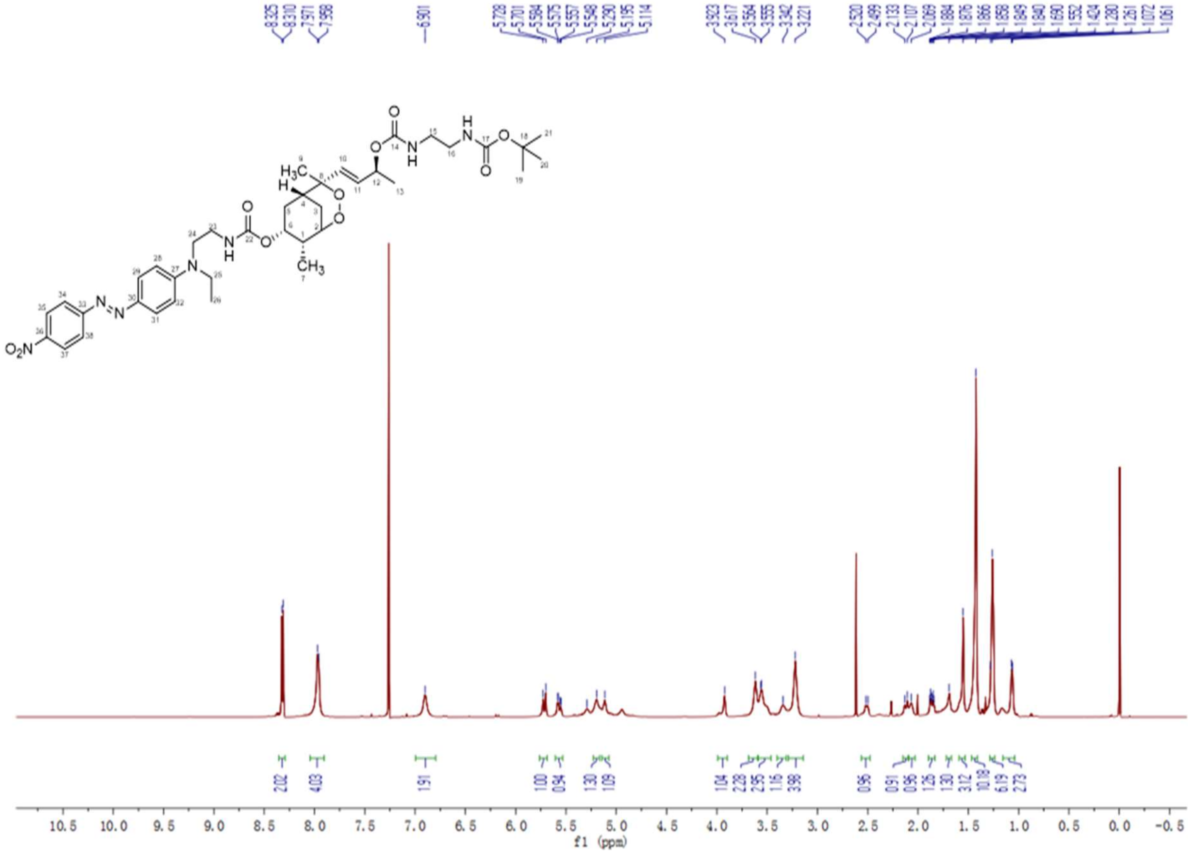

Fig. S147 ^{1}H NMR spectrum of compound IX-2a in $CDCl_3$ (500 MHz)

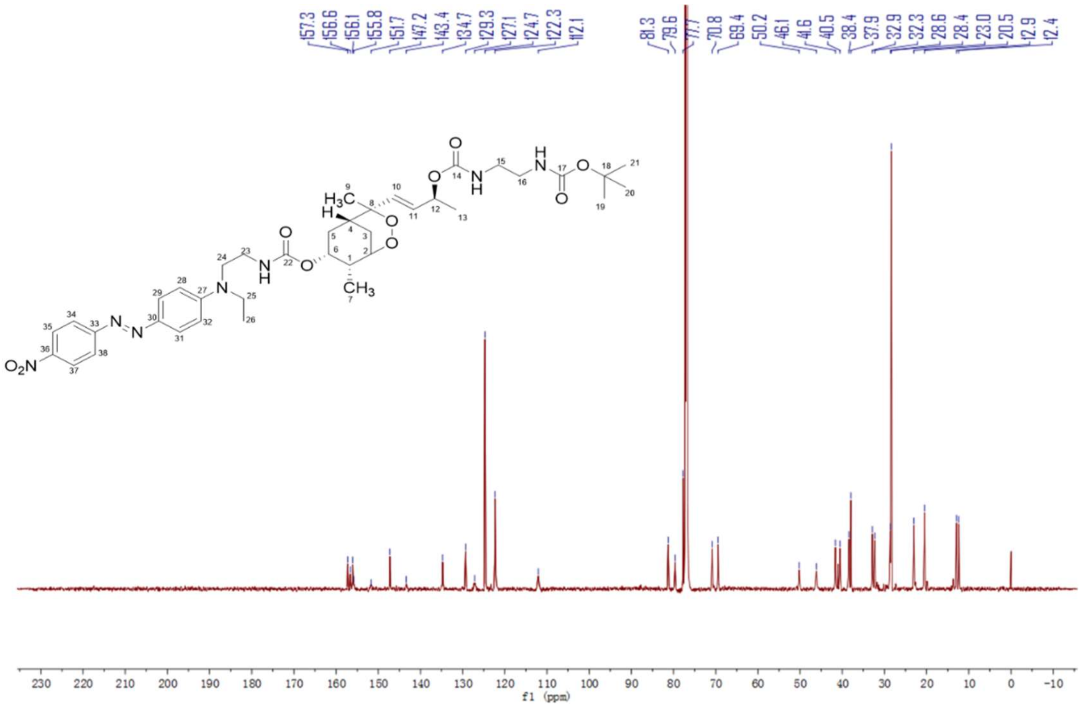

Fig. S148 ^{13}C NMR spectrum of compound IX-2a in $CDCl_3$ (125 MHz)

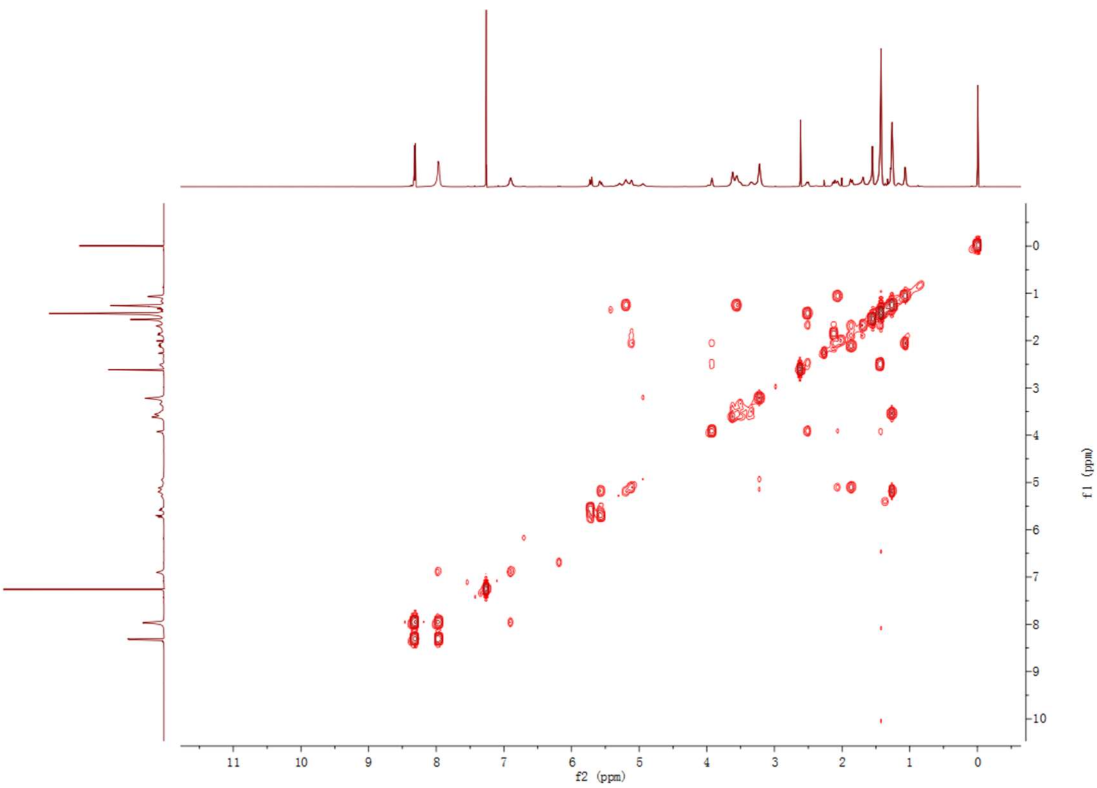

Fig. S149 ^{1}H-^{1}H COSY spectrum of compound IX-2a in $CDCl_3$ (500 MHz)

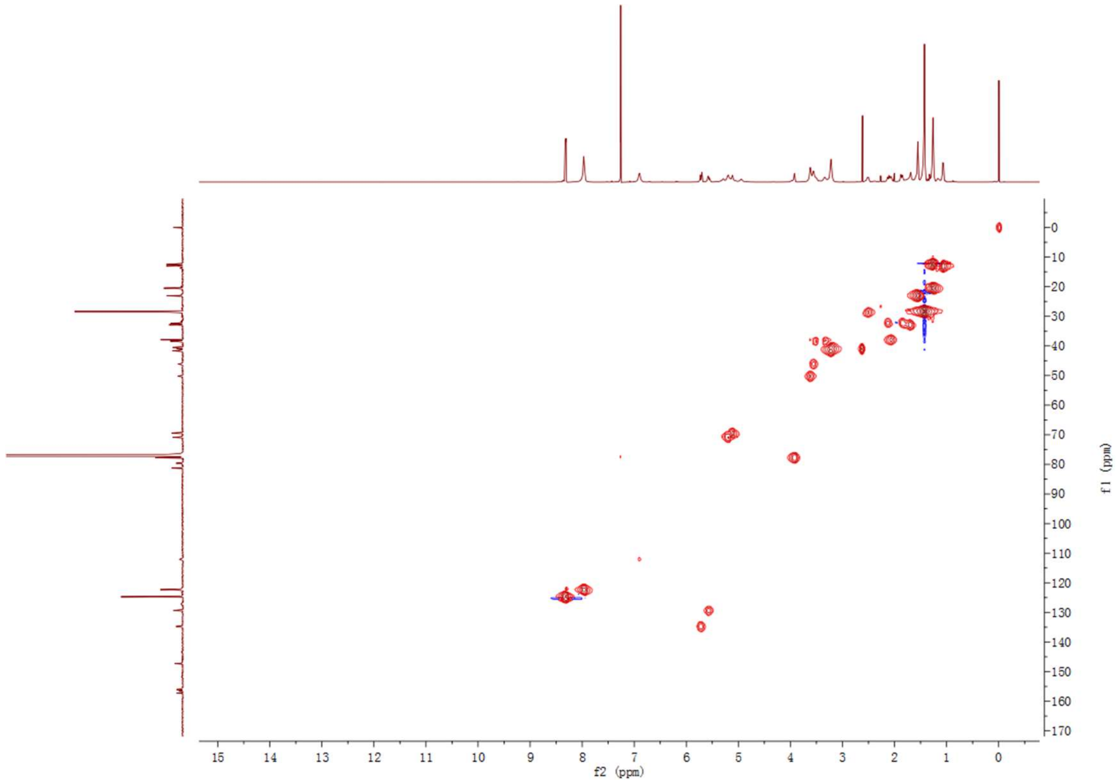

Fig. S150 HSQC spectrum of compound IX-2a in $CDCl_3$ (500 MHz)

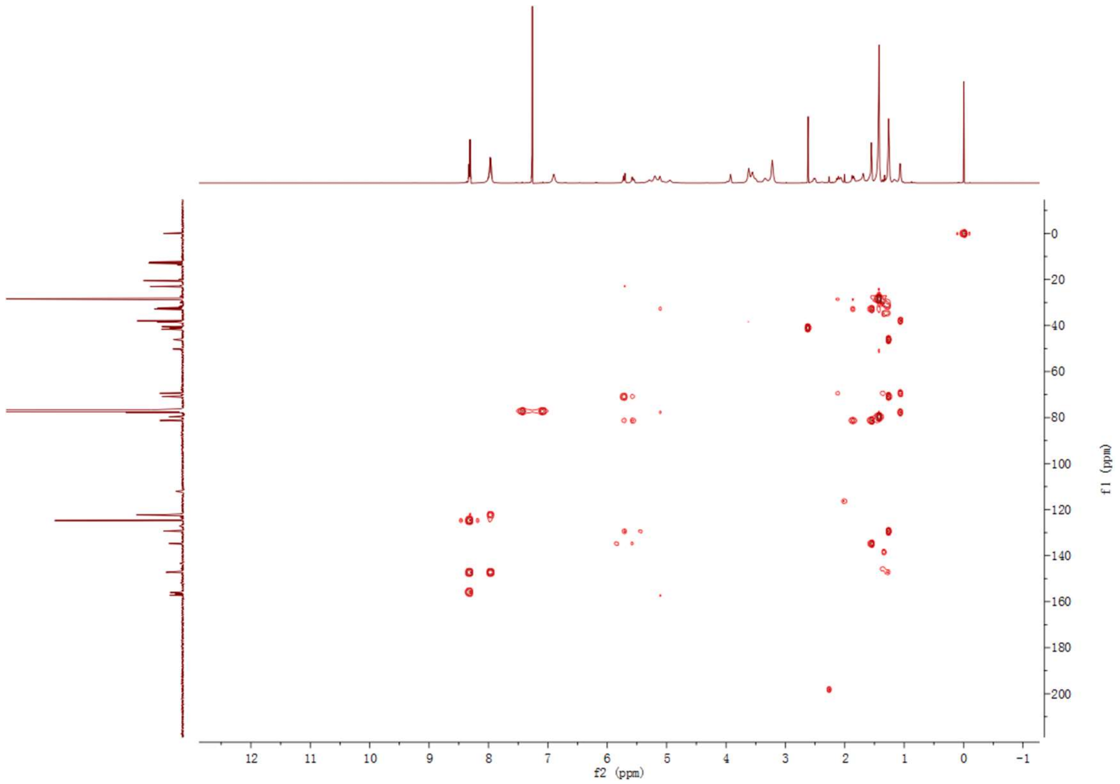

Fig. S151 HMBC spectrum of compound IX-2a in $CDCl_3$ (500 MHz)

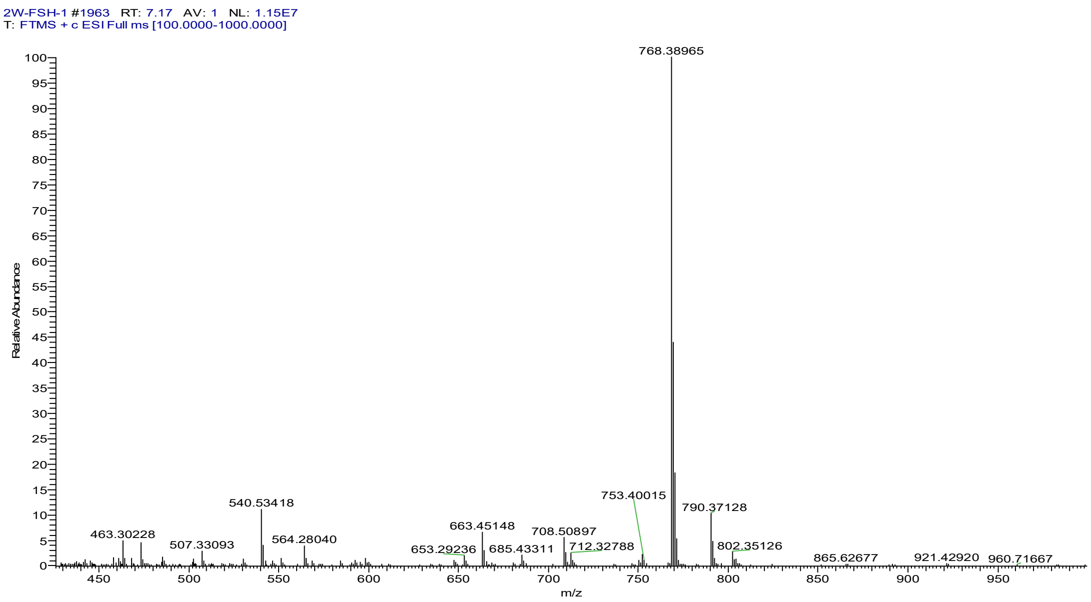

Fig. S152 HRMS data of compound IX-2a

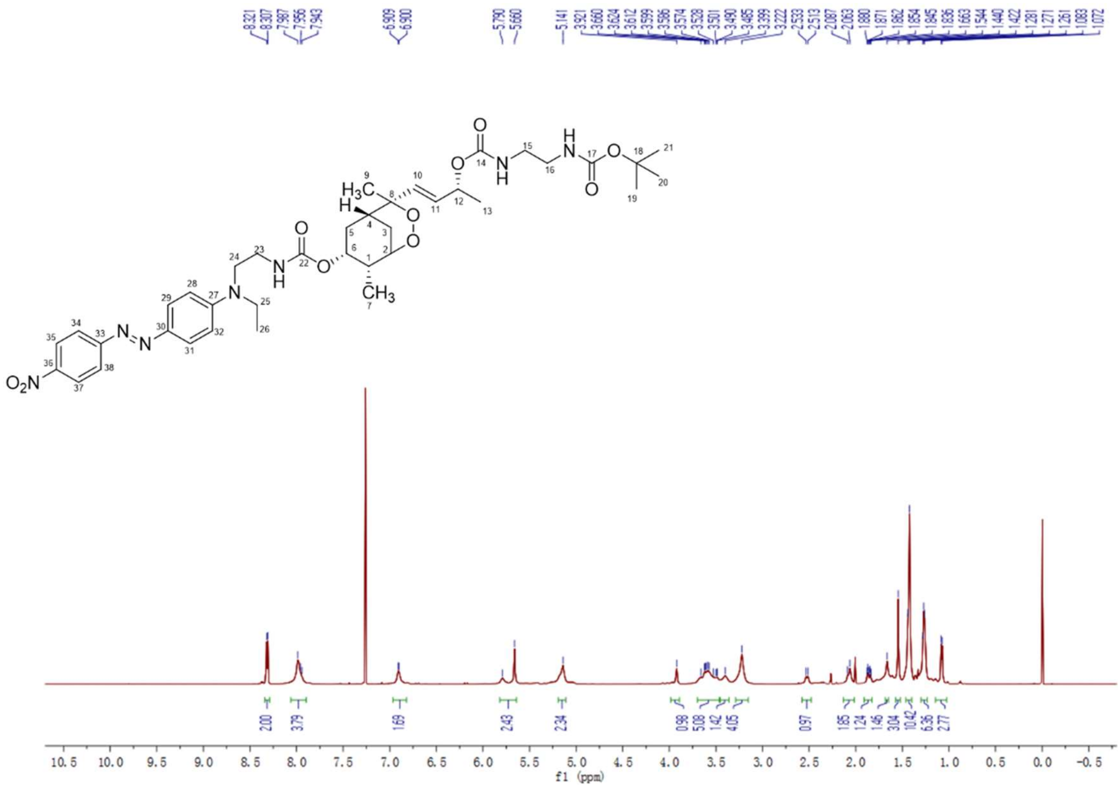

Fig. S153 ^{1}H NMR spectrum of compound IX-2b in $CDCl_3$ (500 MHz)

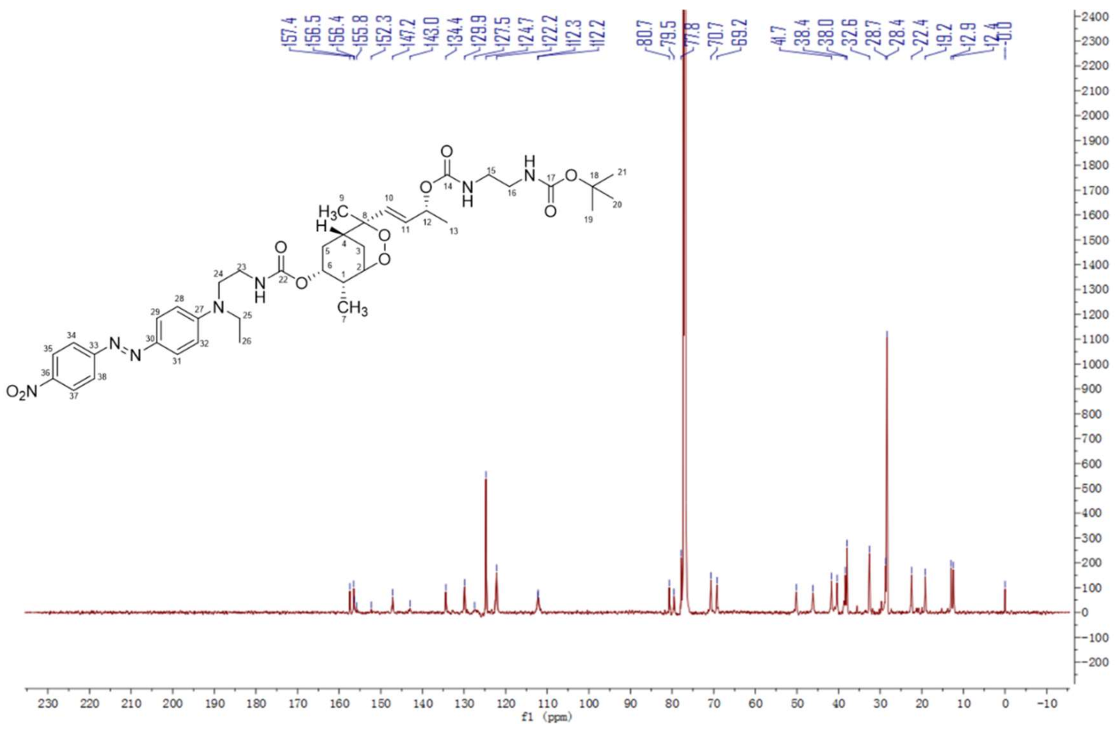

Fig. S154 ^{13}C NMR spectrum of compound IX-2b in $CDCl_3$ (125 MHz)

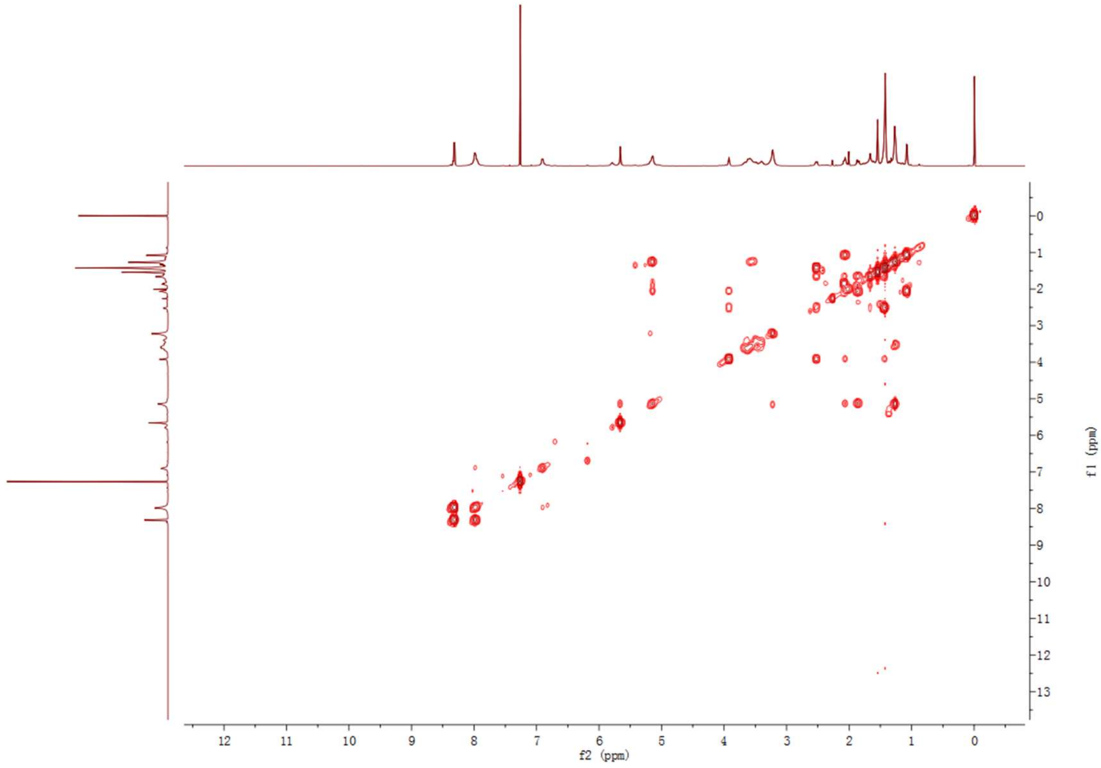

Fig. S155 ^{1}H-^{1}H COSY spectrum of compound IX-2b in $CDCl_3$ (500 MHz)

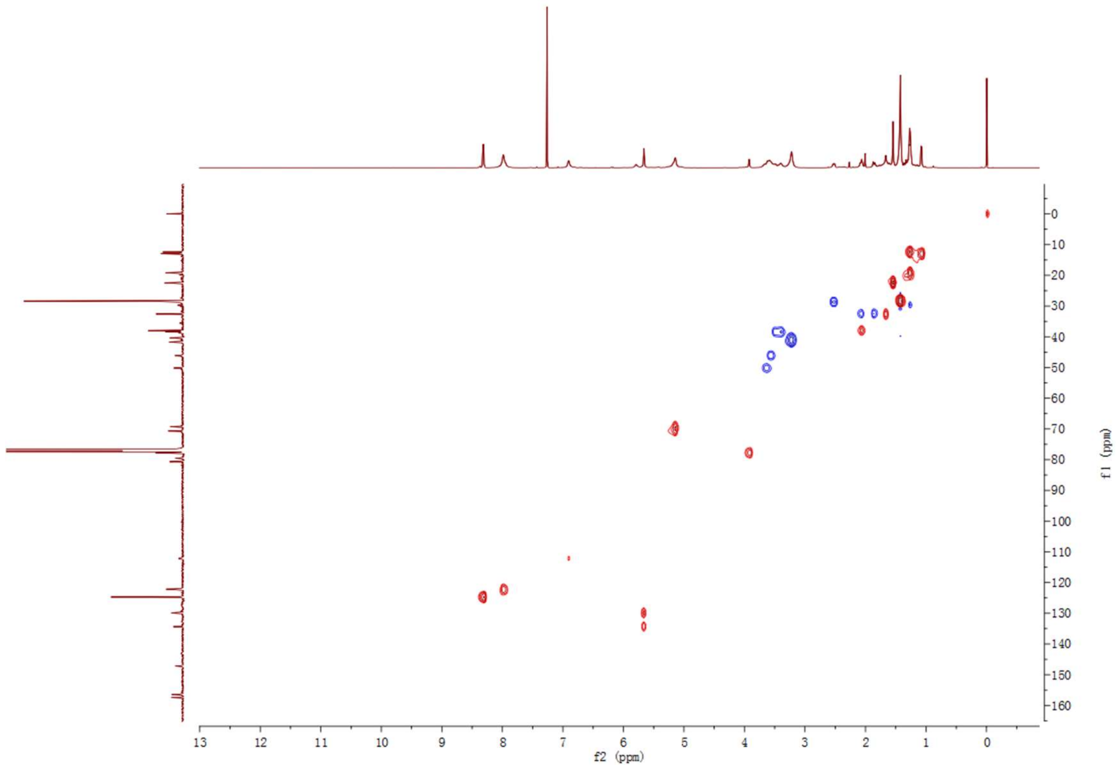

Fig. S156 HSQC spectrum of compound IX-2b in CDCl₃ (500 MHz)

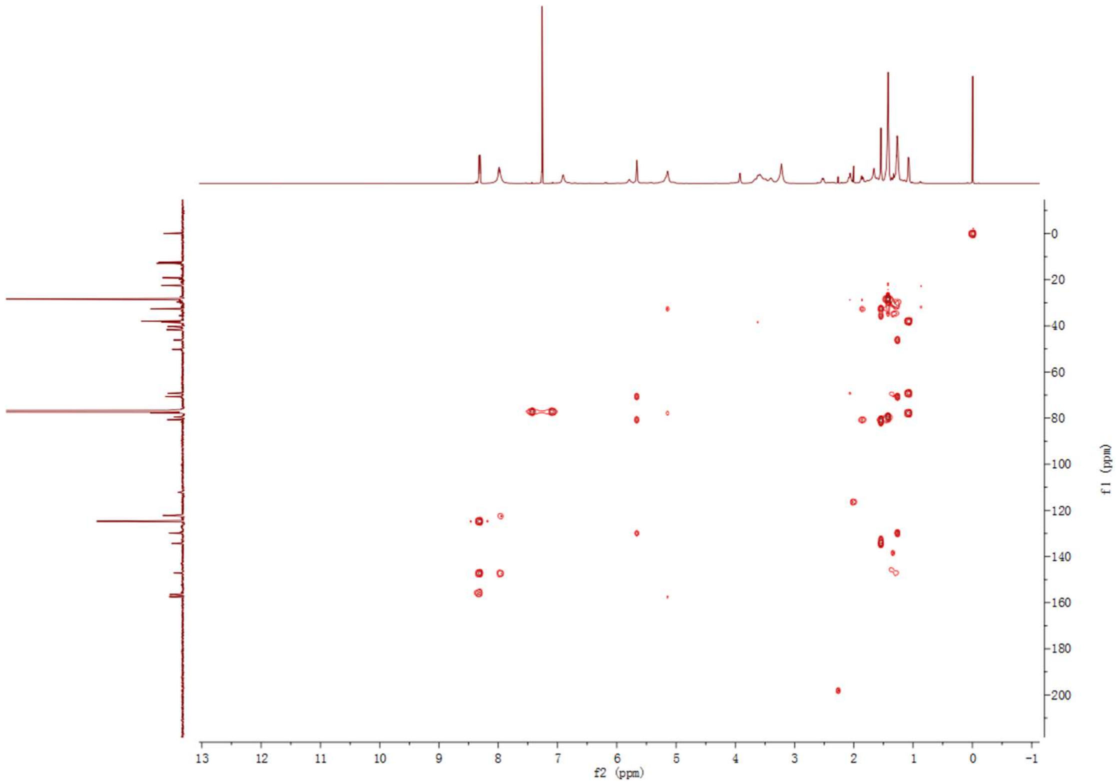

Fig. S157 HMBC spectrum of compound IX-2b in CDCl₃ (500 MHz)

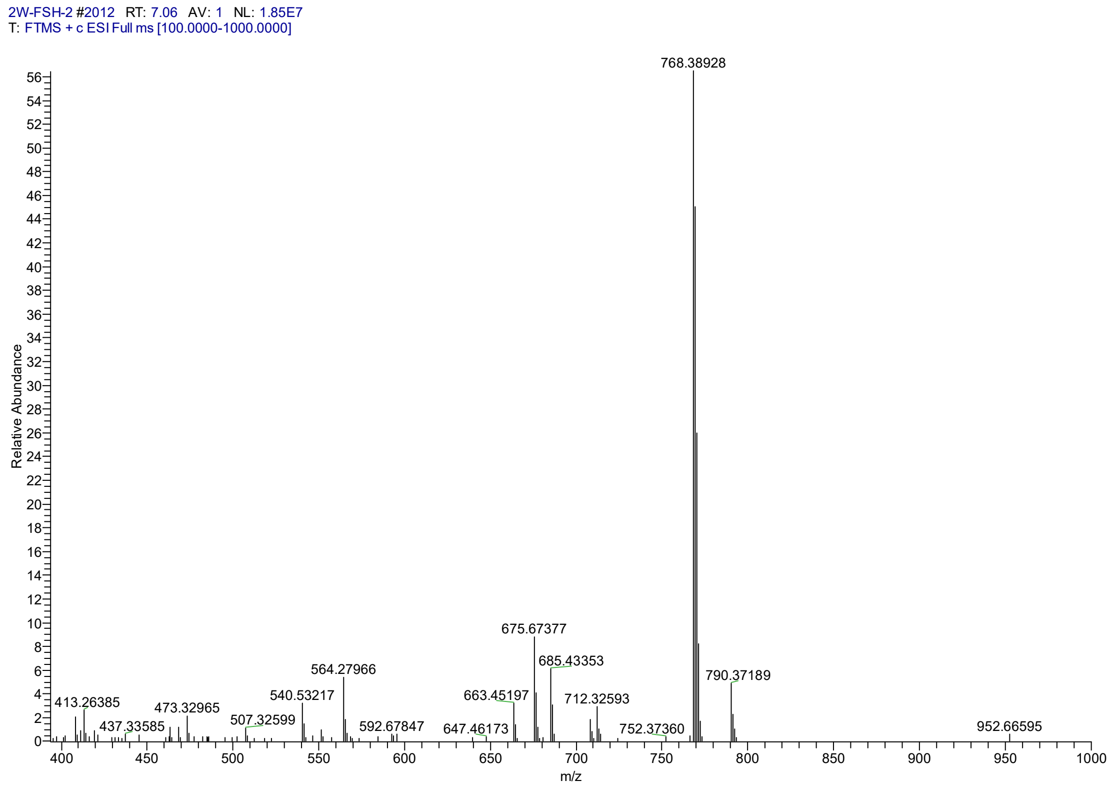

Fig. S158 HRMS data of compound IX-2b

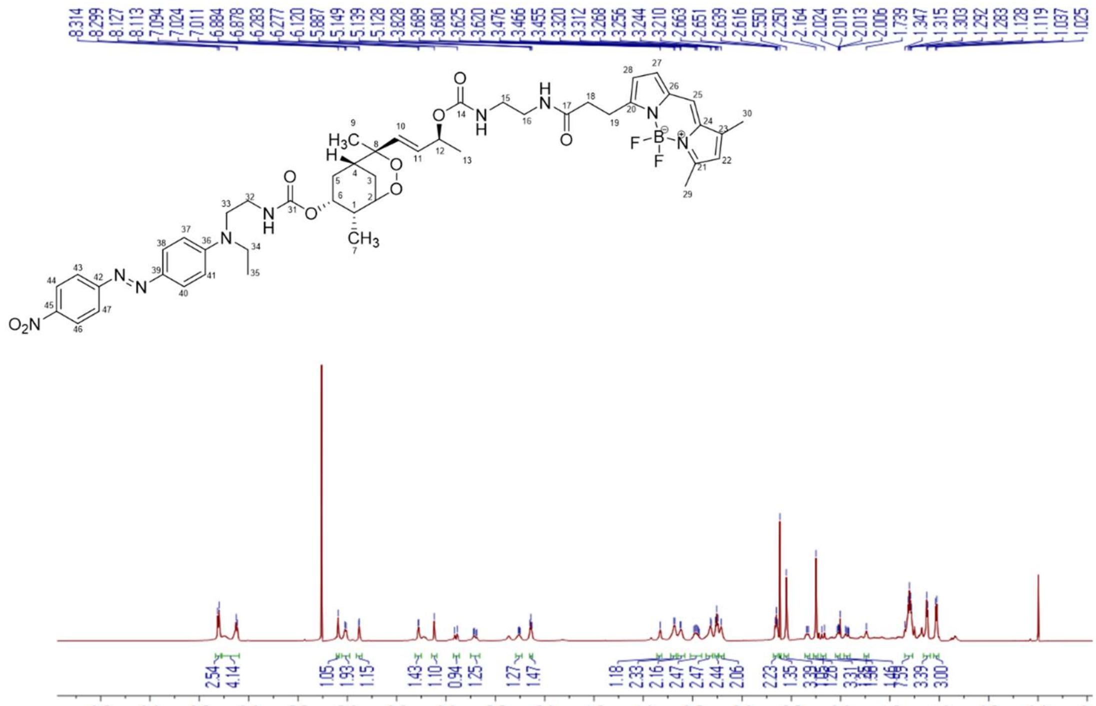

Fig. S159 ^{1}H NMR spectrum of fluorescent probe X-1a in $CDCl_3$ (600 MHz)

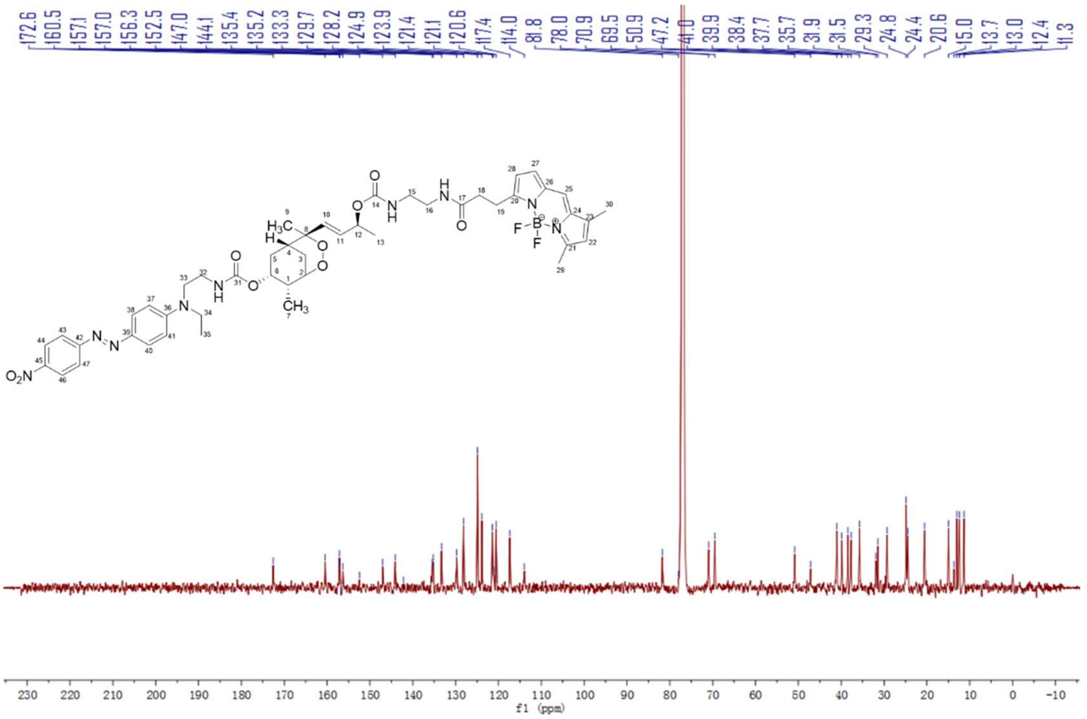

Fig. S160 ^{13}C NMR spectrum of fluorescent probe X-1a in $CDCl_3$ (150 MHz)

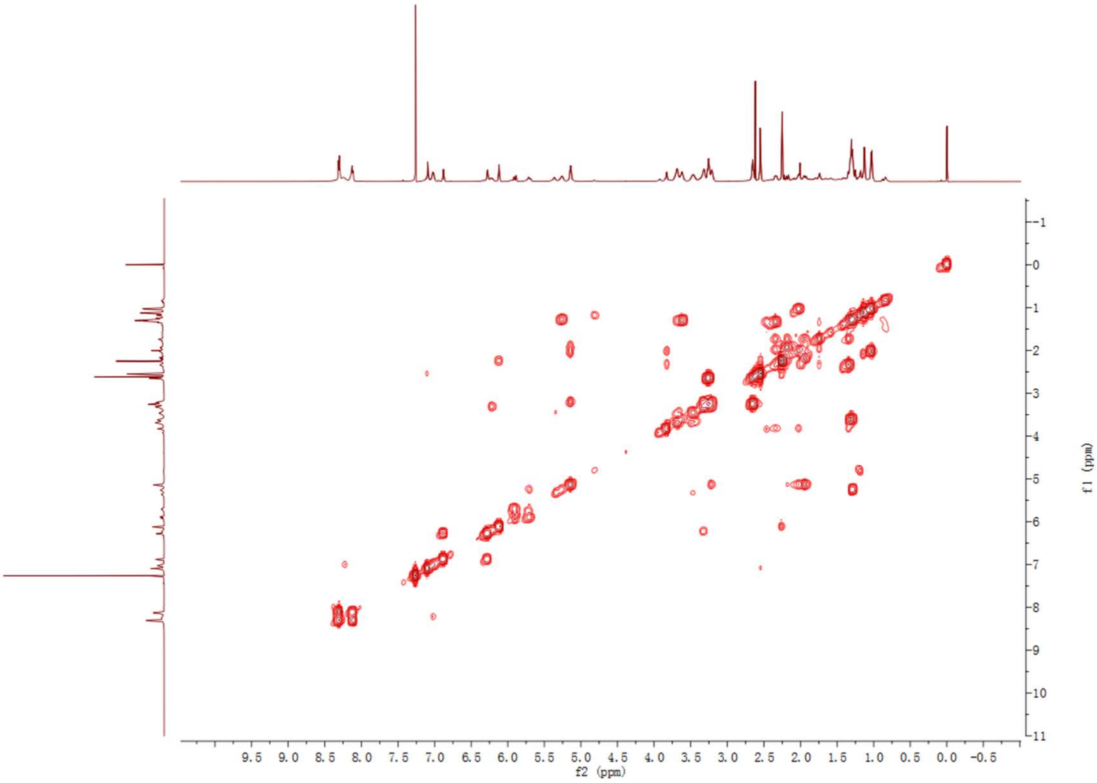

Fig. S161 ^{1}H-^{1}H COSY spectrum of fluorescent probe X-1a in $CDCl_3$ (600 MHz)

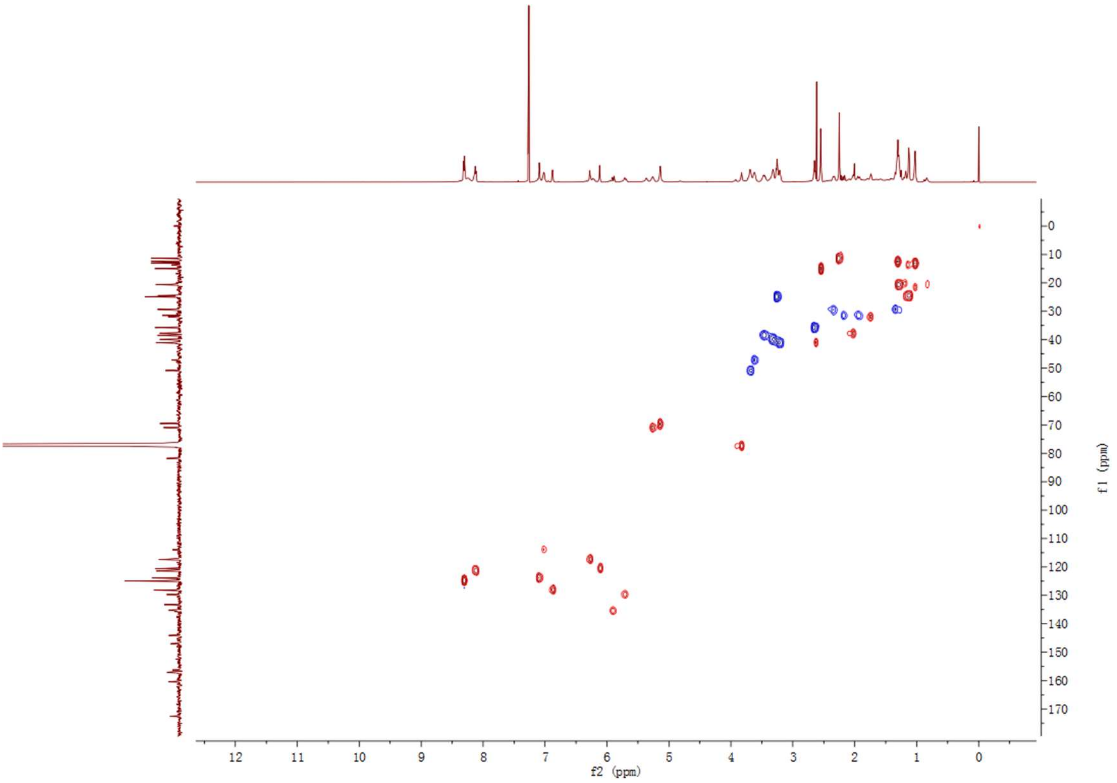

Fig. S162 HSQC spectrum of fluorescent probe X-1a in $CDCl_3$ (600 MHz)

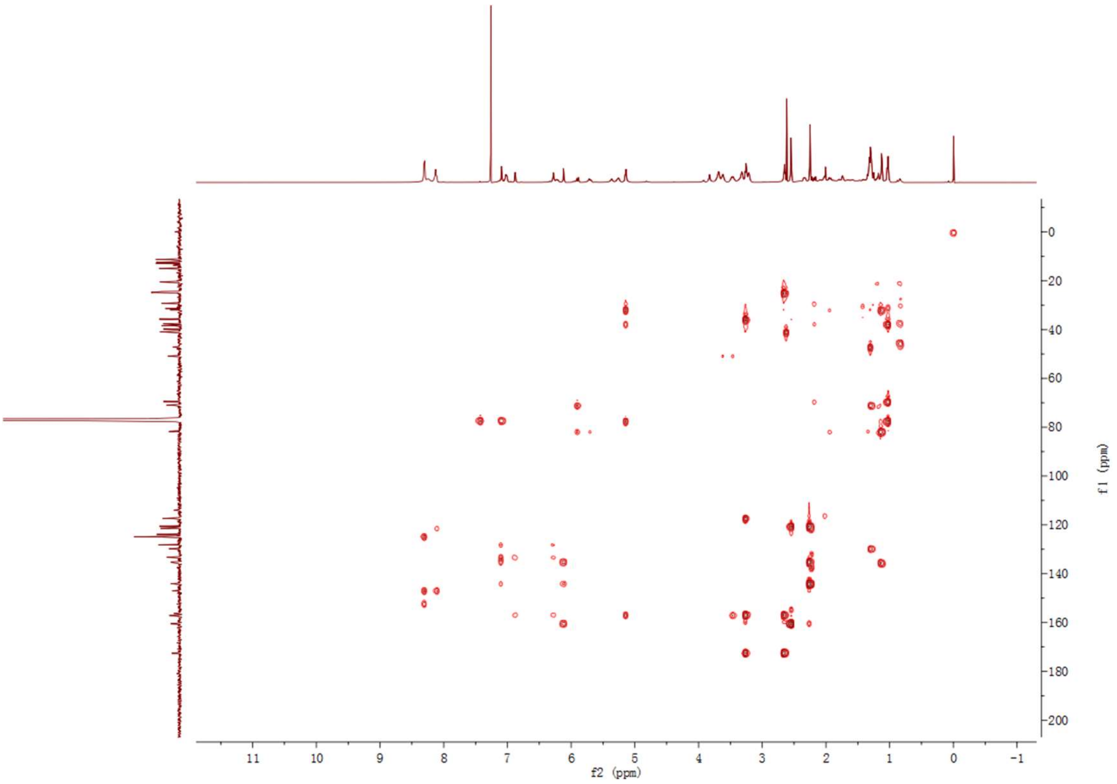

Fig. S163 HMBC spectrum of fluorescent probe X-1a in $CDCl_3$ (600 MHz)

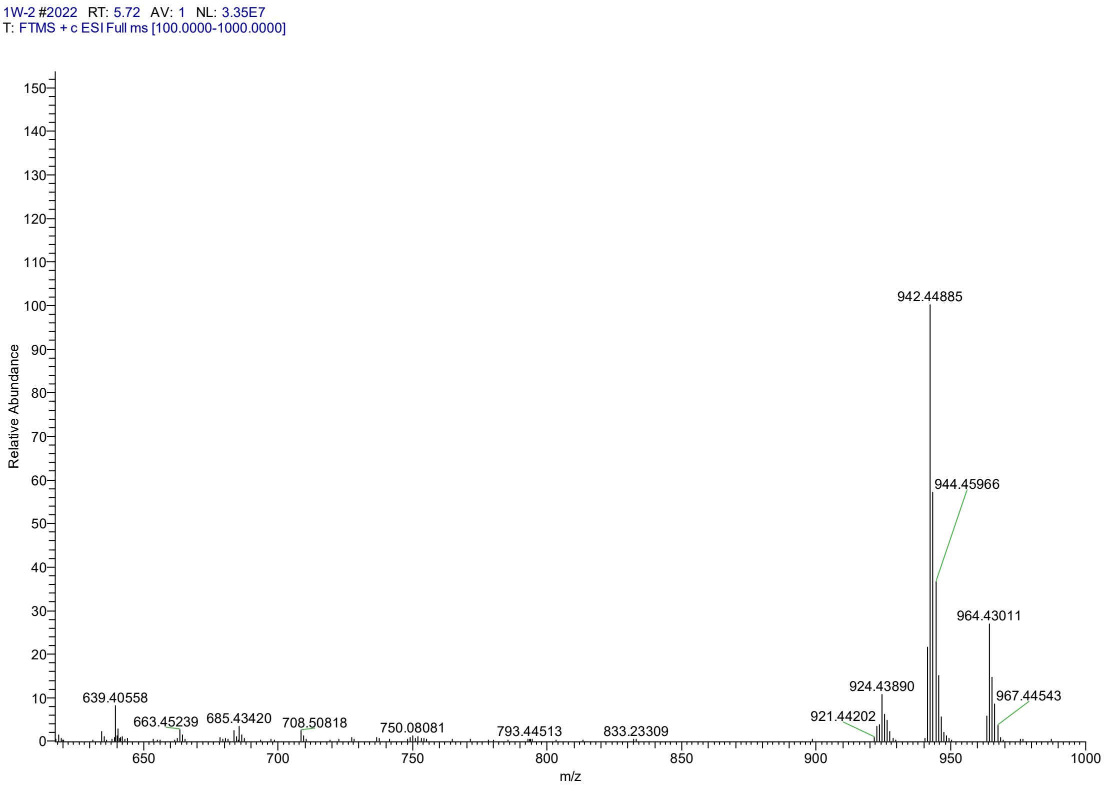

Fig. S164 HRMS data of fluorescent probe X-1a

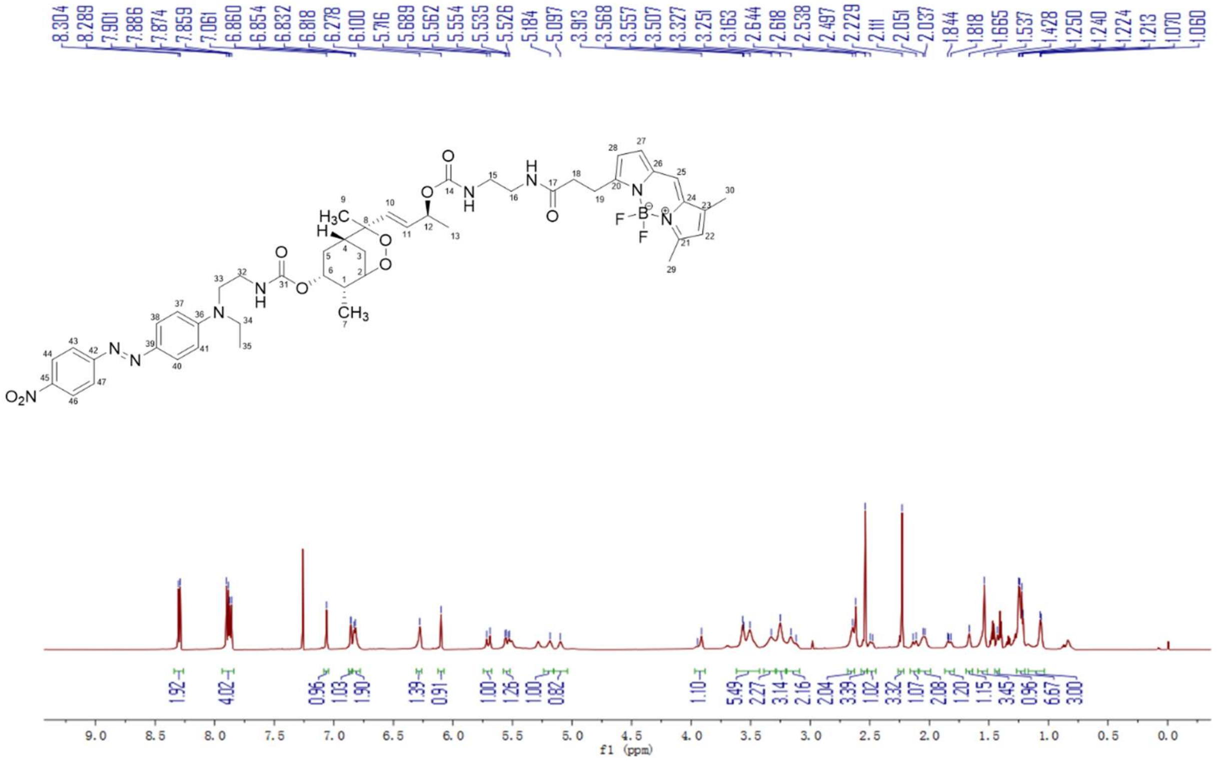

Fig. S165 ^{1}H NMR spectrum of fluorescent probe X-2a in $CDCl_3$ (600 MHz)

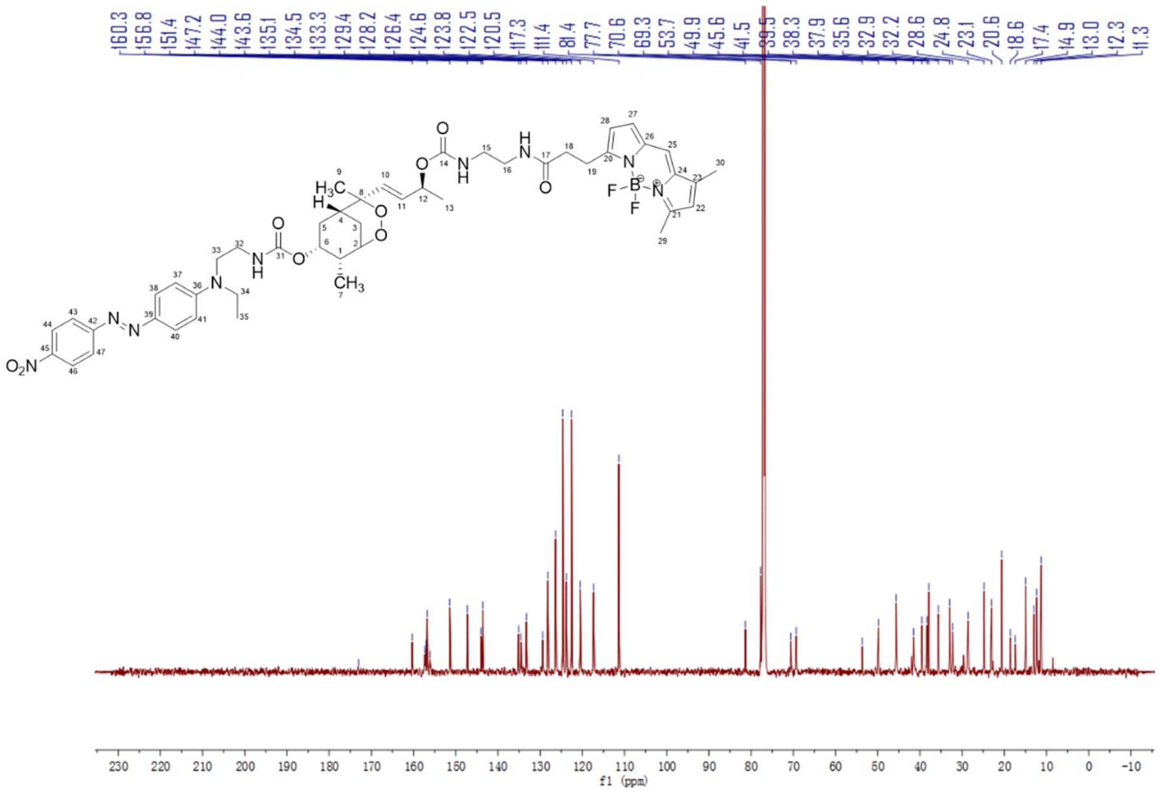

Fig. S166 ^{13}C NMR spectrum of fluorescent probe X-2a in $CDCl_3$ (150 MHz)

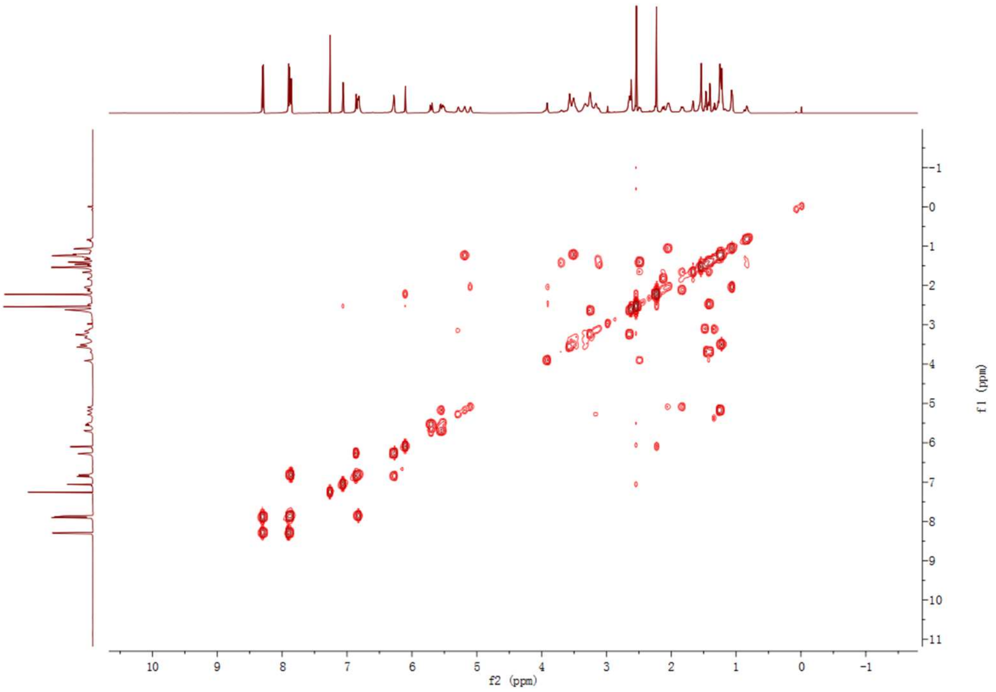

Fig. S167 ^{1}H-^{1}H COSY spectrum of fluorescent probe X-2a in $CDCl_3$ (600 MHz)

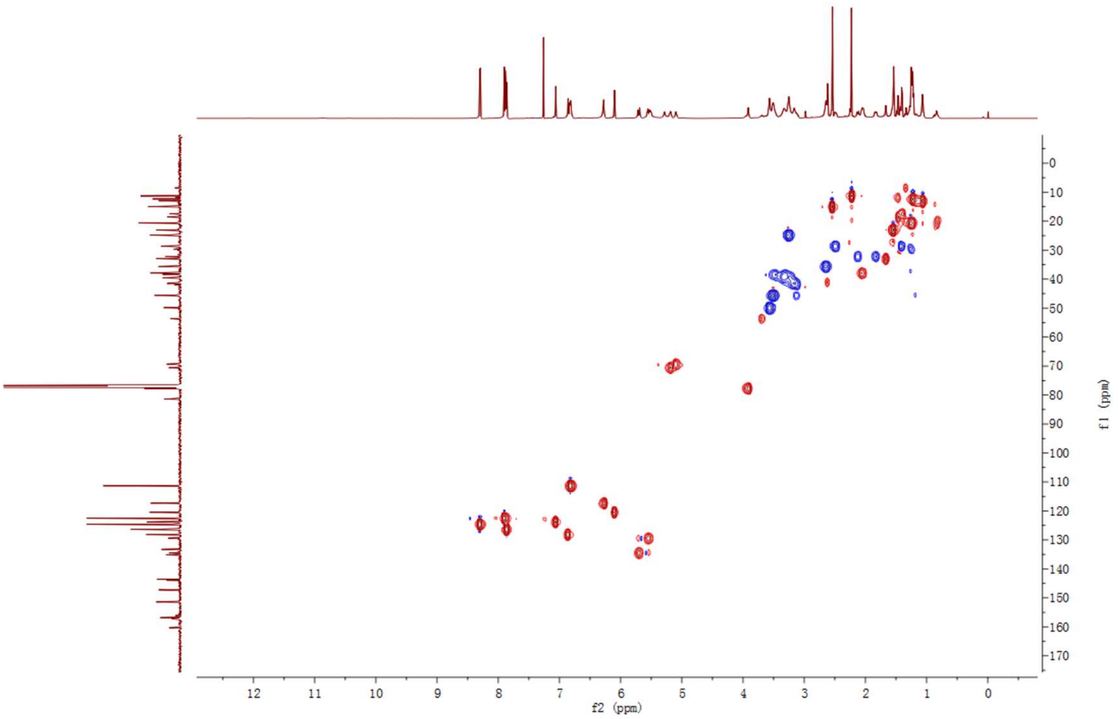

Fig. S168 HSQC spectrum of fluorescent probe X-2a in $CDCl_3$ (600 MHz)

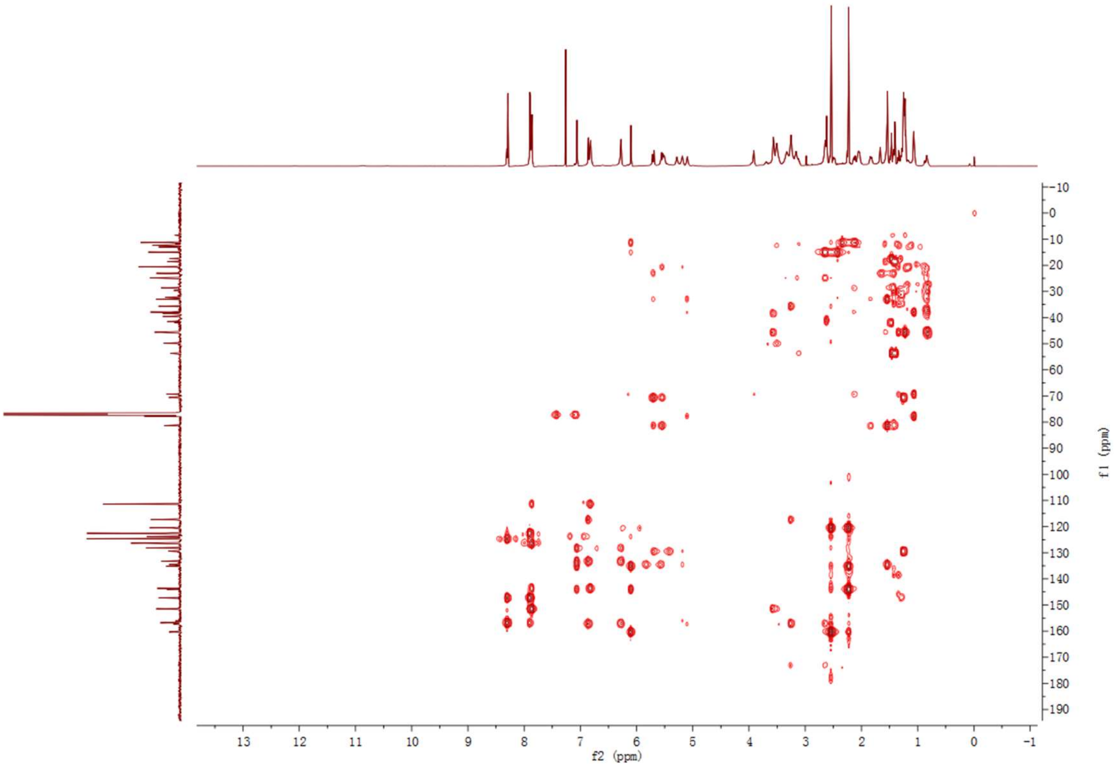

Fig. S169 HMBC spectrum of fluorescent probe X-2a in $CDCl_3$ (600 MHz)

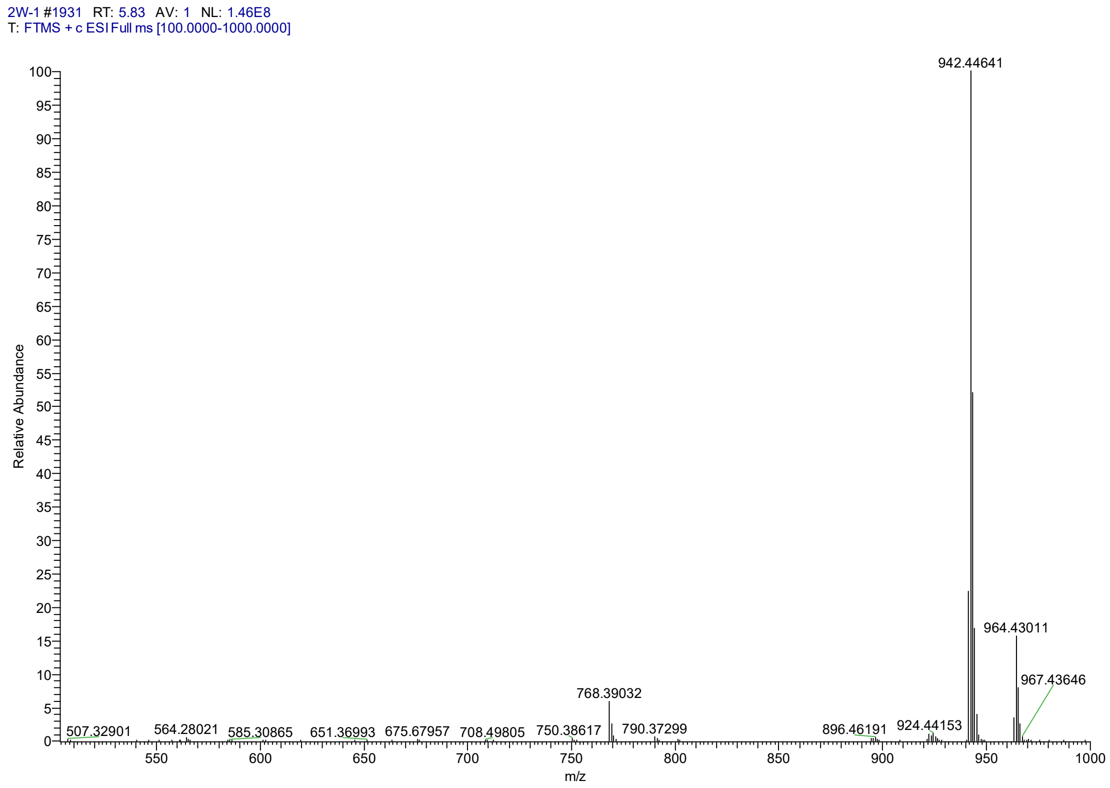

Fig. S170 HRMS data of fluorescent probe X-2a

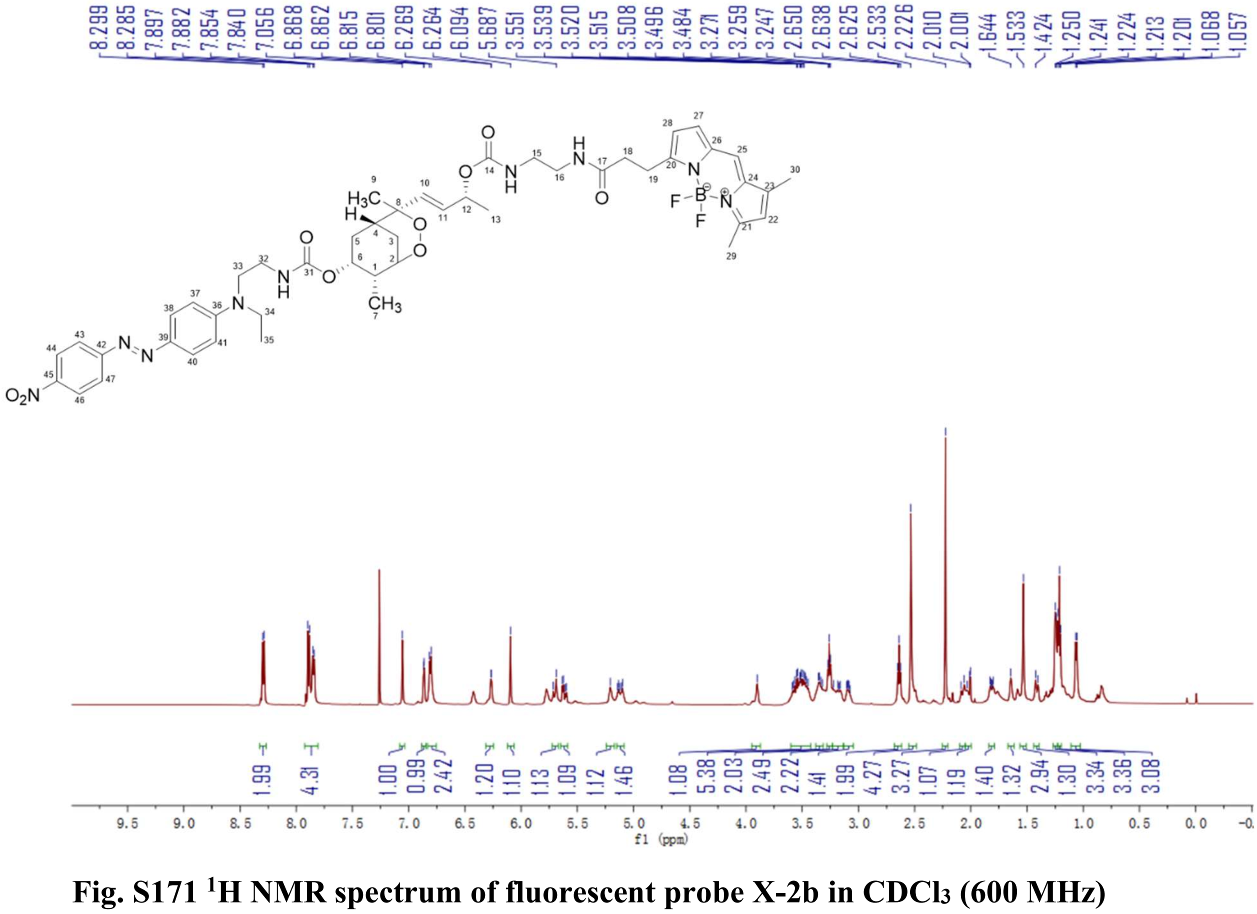

Fig. S171 ^{1}H NMR spectrum of fluorescent probe X-2b in $CDCl_3$ (600 MHz)

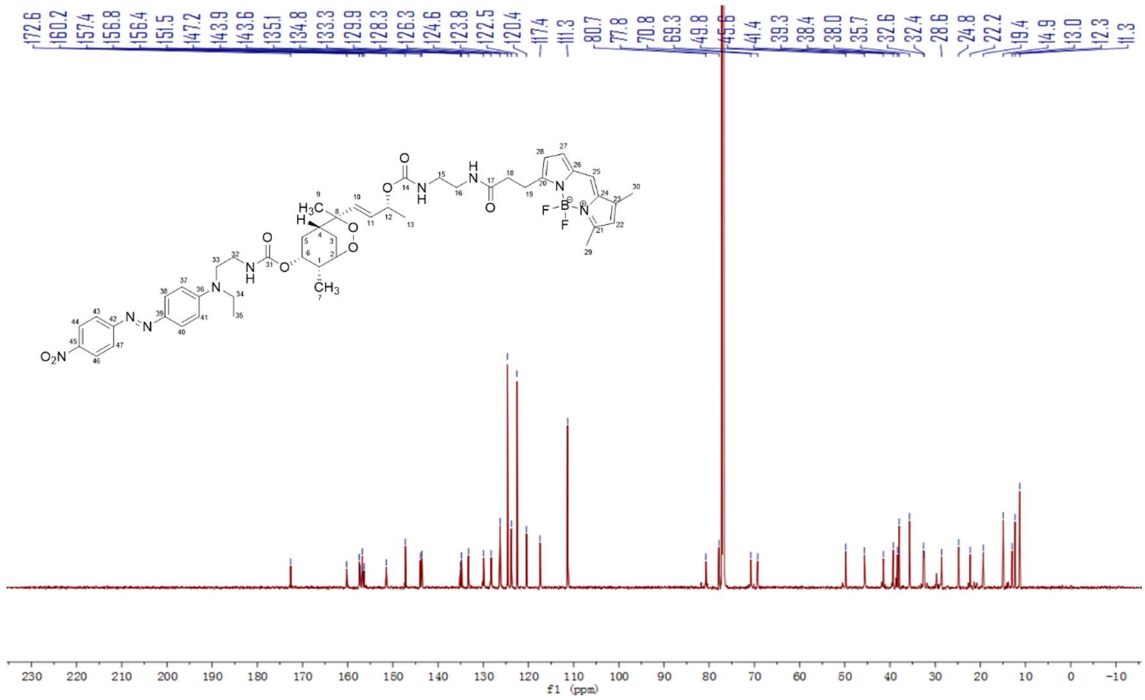

Fig. S172 ^{13}C NMR spectrum of fluorescent probe X-2b in $CDCl_3$ (150 MHz)

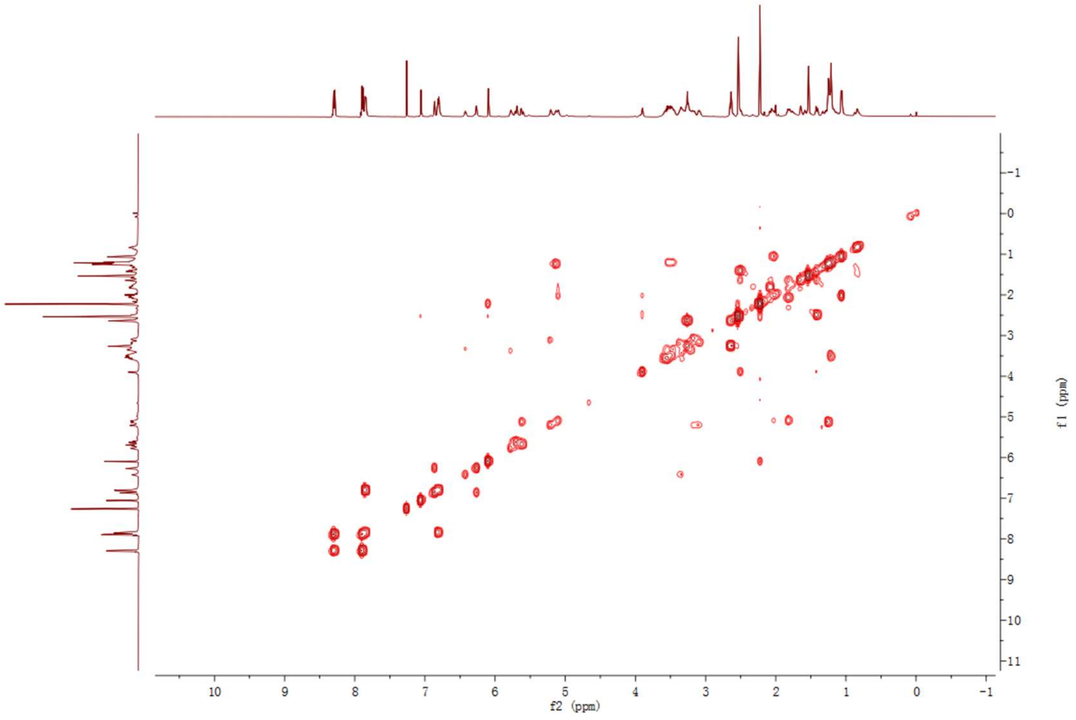

Fig. S173 ^{1}H-^{1}H COSY spectrum of fluorescent probe X-2b in $CDCl_3$ (600 MHz)

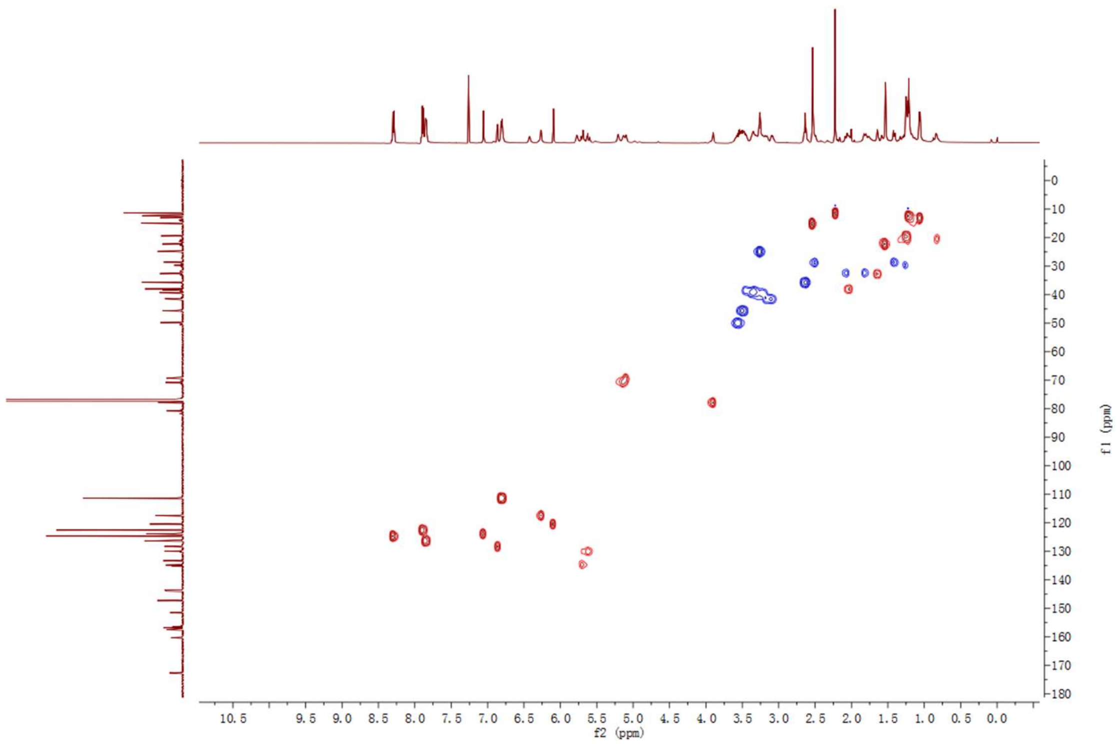

Fig. S174 HSQC spectrum of fluorescent probe X-2b in $CDCl_3$ (600 MHz)

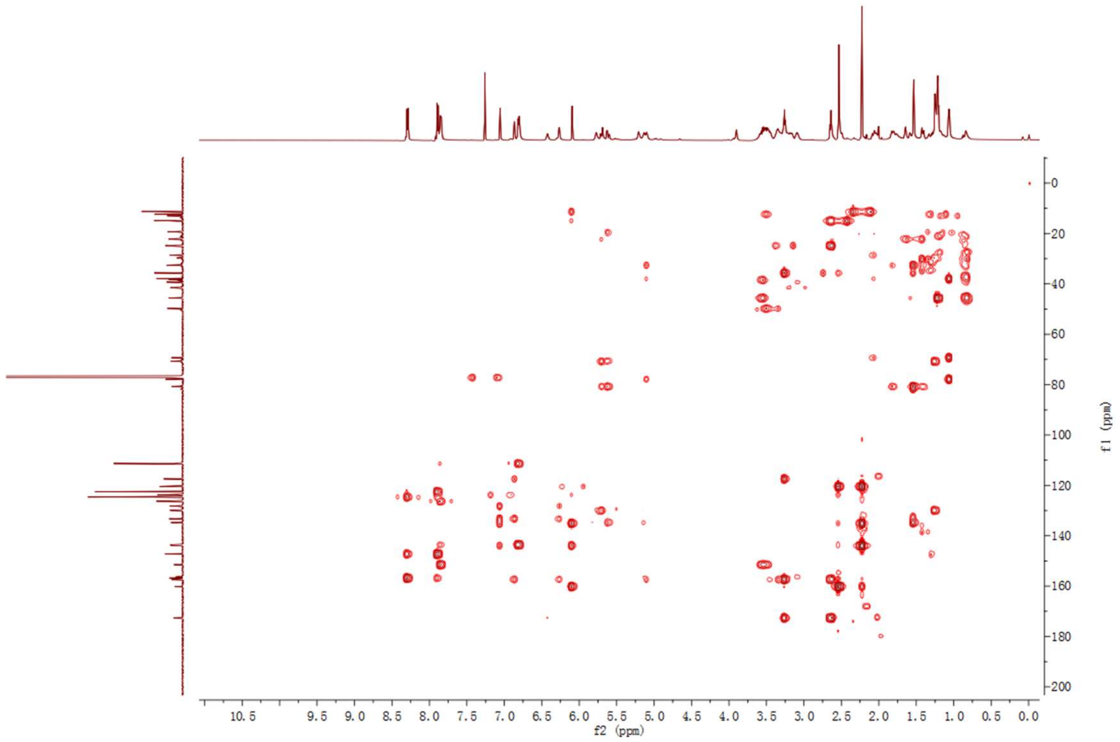

Fig. S175 HMBC spectrum of fluorescent probe X-2b in $CDCl_3$ (600 MHz)

Supplementary Schemes

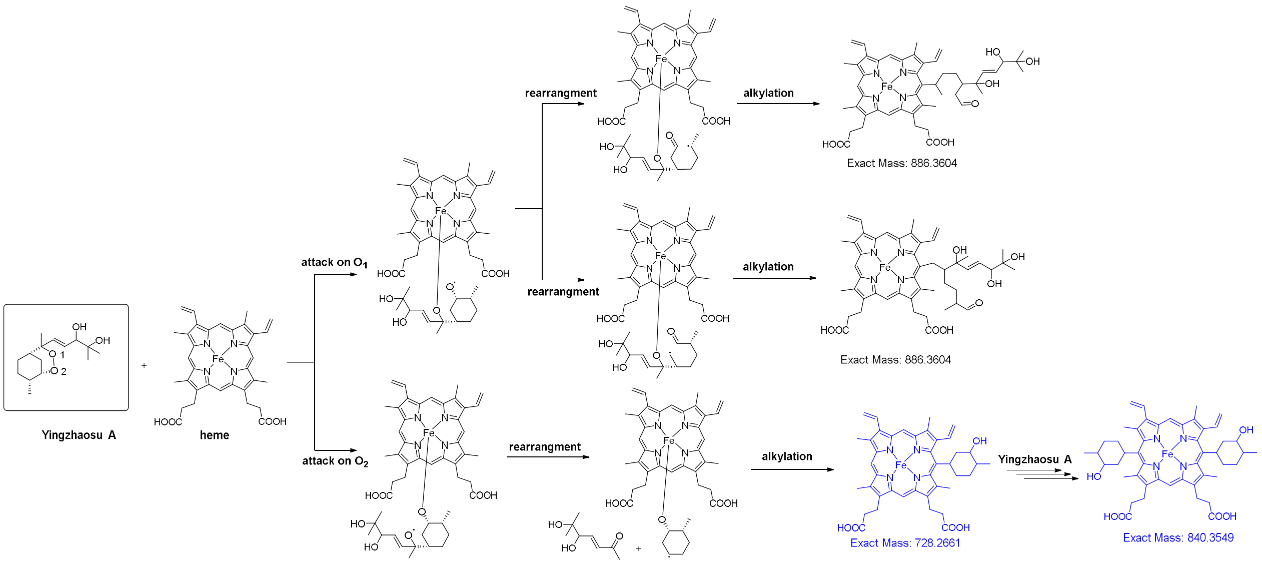

Scheme S1. The plausible products of the reaction between Yingzhaosu A and heme. Only compounds colored in blue were observed in the analysis of mass spectroscopy.

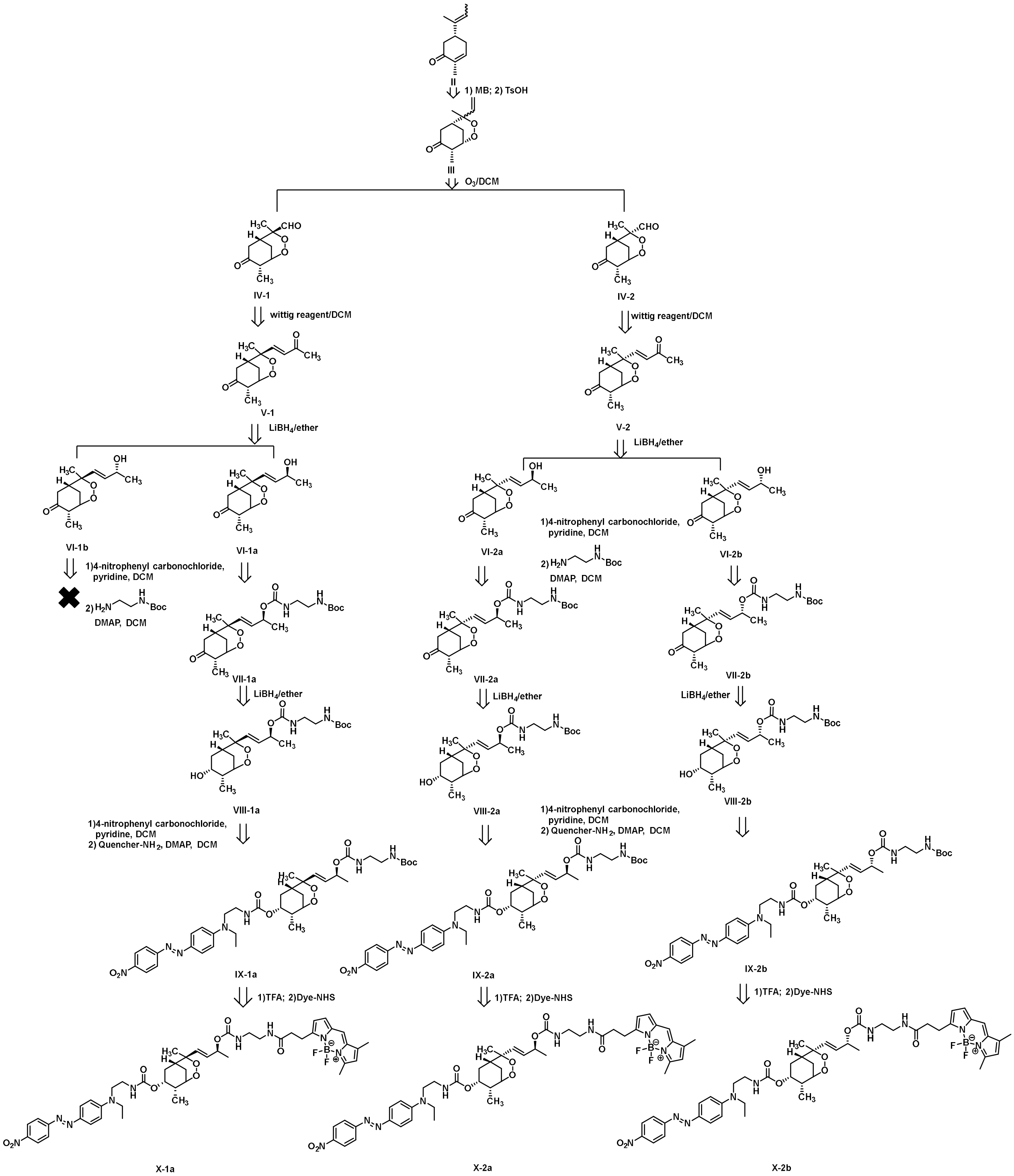

Scheme S2. The complete synthetic route of probes. Following the intermediate VI-1b, we did not obtain the final probe.

Supplementary Tables

Table S1. Selected ^{1}H NMR data for MTPA esters of VI-1aR and VI-1aS (500 MHz, $CDCl_3$)

proton	VI-1aS	VI-1aR	$\Delta\delta^{SR}$ (δ_S - δ_R)
4	2.232	2.204	+0.028
9	1.127 (s)	1.107 (s)	+0.020
10	6.057 (d, 16.0)	6.008 (d, 16.0)	+0.049
11	5.820 (dd, 16.0, 7.5)	5.719 (dd, 16.0, 7.5)	+0.101
13	1.393 (d, 6.5)	1.457 (d, 6.5)	-0.064

Table S2. Selected ^{1}H NMR data for MTPA esters of VI-2aR and VI-2aS (500 MHz, $CDCl_3$)

proton	VI-2aS	VI-2aR	$\Delta\delta^{SR}$ (δ_S - δ_R)
4	2.093	2.035	+0.058
5a	2.657	2.643	+0.014
5b	2.300 (dd, 15.5, 4.5)	2.281 (dd, 15.5, 4.5)	+0.019
9	1.564 (s)	1.522 (s)	+0.042
10	5.592 (d, 16.0)	5.439 (d, 16.5)	+0.153
11	5.489 (dd, 16.0, 6.5)	5.367 (dd, 16.5, 6.0)	+0.122
13	1.354 (d, 6.5)	1.403 (d, 6.5)	-0.049

Table S3. Selected ^{1}H NMR data for MTPA esters of VI-2bR and VI-2bS (500 MHz, $CDCl_3$)

proton	VI-2bS	VI-2bR	$\Delta\delta^{SR}$ (δ_S - δ_R)
4	2.024	2.119	-0.095
5b	2.232 (dd, 16.0, 4.5)	2.282 (dd, 15.5, 4.5)	-0.050
9	1.499 (s)	1.564 (s)	-0.065
10	5.396	5.604 (d, 15.5)	-0.204
11	5.389	5.447 (dd, 15.5, 7.0)	-0.058
13	1.401 (d, 7.0)	1.317 (d, 6.5)	+0.084

Table S4. Selected ^{1}H NMR data for MTPA esters of VIII-2aR and VIII-2aS (500 MHz, $CDCl_3$)

proton	VIII-2aS	VIII-2aR	$\Delta\delta^{SR}$ (δ_S - δ_R)
1	2.186	2.195	-0.009
2	3.917	3.935	-0.018
3a	2.549	2.536	+0.013
3b	1.471	1.455	+0.016
4	1.706	1.658	+0.048
5a	2.231	2.095	+0.136
5b	2.007 (ddd, 16.5, 7.0, 5.0)	1.945 (dt, 16.0, 6.0)	+0.062
6	5.672 (dd, 7.0)	5.672 (dd, 7.0)	0
7	1.037 (d, 7.0)	1.144 (d, 7.0)	-0.107

Table S5. Selected ^{1}H NMR data for MTPA esters of VIII-2bR and VIII-2bS (500 MHz, $CDCl_3$)

proton	VIII-2bS	VIII-2bR	$\Delta\delta^{SR}$ (δ_S - δ_R)
1	2.187	2.197	-0.010
2	3.916	3.935	-0.019
3a	2.550	2.537	+0.013
3b	1.473	1.461	+0.012
4	1.708	1.666	+0.042
5a	2.237	2.100	+0.137
5b	2.006	1.947	+0.059
7	1.039 (d, 7.0)	1.146 (d, 7.0)	-0.107

Table S6. Crystal data and structure refinement for exp_5693 (IV-1 hemiacetal).

Identification code	exp_5693
Empirical formula	$C_{11}H_{18}O_5$
Formula weight	230.25
Temperature / K	111.50(10)
Crystal system	orthorhombic
Space group	$P2_12_12_1$
a / Å, b / Å, c / Å	8.7710(8), 10.9944(5), 11.9136(4)
α/°, β/°, γ/°	90, 90, 90
Volume / Å^3	1148.86(13)
Z	4
ρ_{calc} / mg mm^{-3}	1.331
μ / mm^{-1}	0.878
F(000)	496
Crystal size / mm^3	0.400 × 0.360 × 0.180
2Θ range for data collection	10.95 to 142.346°
Index ranges	$-6 \le h \le 10$, $-13 \le k \le 8$, $-14 \le l \le 11$
Reflections collected	3749
Independent reflections	2163[R(int) = 0.0271 (inf-0.9Å)]
Data/restraints/parameters	2163/0/149
Goodness-of-fit on F^2	1.036
Final R indexes [$I>2\sigma (I)$ i.e. $F_o>4\sigma (F_o)$]	R_1 = 0.0360, wR_2 = 0.0904
Final R indexes [all data]	R_1 = 0.0370, wR_2 = 0.0914
Largest diff. peak/hole / e Å^{-3}	0.246/-0.283
Flack Parameters	-0.09(11)
Completeness	0.9984

Table S7. Fractional atomic coordinates (×10^4) and equivalent isotropic displacement parameters (Å^2×10^3) for exp_5693 (IV-1 hemiacetal). U_{eq} is defined as 1/3 of of the trace of the orthogonalised U_{IJ} tensor.

Atom	*x*	*y*	*z*	U(eq)
O1	-588.7(17)	-9634.0(13)	-8181.4(12)	13.8(3)
O2	-869.3(17)	-9873.2(13)	-6983.2(12)	16.2(3)
O5	-3487.0(17)	-8750.4(13)	-8745.5(15)	22.1(4)
O4	-4239.6(18)	-10706.4(15)	-9246.7(13)	21.7(4)
C9	-3308(2)	-10009.9(18)	-8550.7(17)	13.5(4)
C4	-1307(2)	-11704.8(17)	-8632.1(17)	12.8(4)
O3	2718.4(18)	-11045.0(17)	-8732.1(16)	28.0(4)
C5	330(3)	-12036.9(19)	-8985.5(18)	17.3(5)
C2	-405(2)	-11108.5(19)	-6753.8(17)	15.3(4)
C6	1534(2)	-11399.2(18)	-8300.0(19)	16.0(4)
C3	-1457(2)	-11973.7(17)	-7374.5(18)	14.6(4)
C11	-4870(3)	-8285(2)	-8294(3)	28.9(6)
C8	-1649(2)	-10343.2(18)	-8846.4(17)	11.8(4)
C7	2338(3)	-10408(2)	-6470(2)	25.2(5)
C10	-1282(3)	-9944(2)	-10041.8(18)	19.2(5)

Table S8. PL intensity of three probes incubated with different conditions for 2 h

Condition	Hemin (1mM) (μL)	SA (10mM) (μL)	GSH (10mM) (μL)	X-1a (1mM) (μL)	X-2a (1mM) (μL)	X-2b (1mM) (μL)	PBS (1×) (μL)	total (μL)
1	10	0	0	0	0	0	190	200
2	0	10	0	0	0	0	190	200
3	0	0	10	0	0	0	190	200
4	10	10	0	0	0	0	180	200
5	10	0	10	0	0	0	180	200
6	0	10	10	0	0	0	180	200
7	10	10	10	0	0	0	170	200
8	0	0	0	10	0	0	190	200
9	0	0	10	10	0	0	180	200
10	0	10	0	10	0	0	180	200
11	0	0	0	0	10	0	190	200
12	0	0	10	0	10	0	180	200
13	0	10	0	0	10	0	180	200
14	0	0	0	0	0	10	190	200
15	0	0	10	0	0	10	180	200
16	0	10	0	0	0	10	180	200
17	10	10	0	10	0	0	170	200
18	10	10	10	10	0	0	160	200

19	10	0	0	10	0	0	180	200
20	10	0	10	10	0	0	170	200
21	10	10	0	0	10	0	170	200
22	10	10	10	0	10	0	160	200
23	10	0	0	0	10	0	180	200
24	10	0	10	0	10	0	170	200
25	10	10	0	0	0	10	170	200
26	10	10	10	0	0	10	160	200
27	10	0	0	0	0	10	180	200
28	10	0	10	0	0	10	170	200

Table S9. PL intensity of three probes incubated with different metal ions for 2 h

Condition[a]	Fe^{2+} (μL)	Fe^{3+} (μL)	Zn^{2+} (μL)	Co^{2+} (μL)	Mg^{2+} (μL)	Ni^{2+} (μL)	Cu^{2+} (μL)	SA (μL)	Ca^{2+} (μL)	Mn^{2+} (μL)	K^{+} (μL)	Na^{+} (μL)	DMSO (μL)	X-1a (μL)	X-2a (μL)	X-2b (μL)	PBS (1×) (μL)	total (μL)
1													10				190	200
2														10			190	200
3	10													10			180	200
4		10												10			180	200
5			10											10			180	200
6				10										10			180	200
7					10									10			180	200
8						10								10			180	200
9							10							10			180	200
10							10	10						10			170	200
11								10						10			180	200
12									10					10			180	200
13										10				10			180	200
14											10			10			180	200
15												10		10			180	200
16															10		190	200
17	10														10		180	200
18		10													10		180	200
19			10												10		180	200
20				10											10		180	200
21					10										10		180	200
22						10									10		180	200
23							10								10		180	200
24							10	10							10		170	200
25								10							10		180	200
26									10						10		180	200
27										10					10		180	200
28											10				10		180	200
29												10			10		180	200
30																10	190	200
31	10															10	180	200
32		10														10	180	200
33			10													10	180	200
34				10												10	180	200

35	10								10	180	200
36		10							10	180	200
37			10						10	180	200
38			10	10					10	170	200
39				10					10	180	200
40					10				10	180	200
41						10			10	180	200
42							10		10	180	200
43								10	10	180	200

a: Fe^{2+}: 0.55 mM, other metal ions: 1 mM; probes: 10 μM; SA: 10 mM.

Table S10 The compound name and CAS No. of screened library of natural products

No	Name	Cas No	No	Name	Cas No	No	Name	Cas No
1	Phorbol	17673-25-5	35	Curcumol	4871-97-0	69	Cephaeline hydrochloride	3738-70-3
2	Vanitiolide	17692-71-6	36	Adenosine 5'-monophosphate	61-19-8	70	Anhydroicaritin	38226-86-7
3	Digitoxin	71-63-6	37	Valepotriate	18296-44-1	71	Resibufogenin	465-39-4
4	Octopamine hydrochloride	770-05-8	38	Monocrotaline	315-22-0	72	Calcium Levofolinate	80433-71-2
5	Benzyladenine	1214-39-7	39	Triptolide	38748-32-2	73	Topotecan hydrochloride	119413-54-6
6	D-Biotin	58-85-5	40	Betulin	473-98-3	74	Nootkatone	4674-50-4
7	Artemether	71963-77-4	41	Harmol	487-03-6	75	alpha-Cyperone	473-08-5
8	Ampicillin	69-53-4	42	Mangostin	6147-11-1	76	Chelidonine	476-32-4
9	Epiandrosterone	481-29-8	43	Cephalomannine	71610-00-9	77	(-)-Licarin B	51020-87-2
10	Actidione	66-81-9	44	Oridonin	28957-04-2	78	Scopolamine	51-34-3
11	Gibberellic acid	1977-6-5	45	Etoposide	33419-42-0	79	Corydaline	518-69-4
12	Praeruptorin A	73069-27-9	46	Decitabine	2353-33-5	80	Noricaritin	5240-95-9
13	Darutoside	59219-65-7	47	Brefeldin A	20350-15-6	81	Coixol	532-91-2
14	Glycocholic acid	475-31-0	48	Schisantherin A	58546-56-8	82	cystine	56-89-3
15	Caryophyllene oxide	1139-30-6	49	Ellagic acid	476-66-4	83	Thiocolchicoside	602-41-5

16	Cortisone	1953-6-5	50	Sophoricoside	152-95-4	84	Calcium Levofolinate	80433-71-2
17	Xanthurenic Acid	59-00-7	51	(S)-(+)-Carvone	2244-16-8	85	Oxytetracycline	79-57-2
18	Mangiferin	4773-96-0	52	Lycorine	476-28-8	86	Dehydroevodiamine	67909-49-3
19	Rheochrysidin	521-61-9	53	Veratramine	60-70-8	87	Phellodendrine	6873-13-8
20	Ftaxilide	19368-18-4	54	Triptophenolide	74285-86-2	88	Ganoderic acid A	81907-62-2
21	D-Camphor	464-49-3	55	Curdione	13657-68-6	89	Skimmianin	83-95-4
22	Isoalantolactone	470-17-7	56	Bardoxolone	218600-44-3	90	rel-(8R,8'R)-dimethyl-(7S,7'R)-bis(3,4-m	178740-32-4
23	(+)-Matrine	519-02-8	57	Saikosaponin D	20874-52-6	91	Sibiricaxanthone B	241125-81-5
24	Rutaecarpine	84-26-4	58	(20R)-Ginsenoside Rh1	80952-71-2	92	Hypaphorine	487-58-1
25	Solasodine	126-17-0	59	Oleuropein	32619-42-4	93	Isoschaftoside	52012-29-0
26	Spectinomycin dihydrochloride	21736-83-4	60	Wedelolactone	524-12-9	94	Piperlongumine	20069-09-4
27	Dicumarol	66-76-2	61	Cinchonine	118-10-5	95	Bullatine A	1354-84-3
28	L(+)-Ascorbic acid	50-81-7	62	Ajugol	52949-83-4	96	Pristimerin	1258-84-0
29	4-Methylumbelliferone	90-33-5	63	Irigenin	548-76-5	97	Aristololactam I	13395-02-3
30	Ethynyl estradiol	57-63-6	64	Atractyloside A	126054-77-1	98	Demethyleneberberine	25459-91-0
31	Tacrolimus	104987-11-3	65	Heteroclitin D	140369-76-2	99	Liensinine	2586-96-1
32	Stevioside	57817-89-7	66	Peimisine	19773-24-1	100	Deguelin	522-17-8
33	Costunolide	553-21-9	67	Xanthotoxol	2009-24-7			
34	Catalpol	2415-24-9	68	Peimine	23496-41-5			
